# AS01_E_-adjuvanted RSV pre-F protein vaccine in adults with lung or kidney transplant: a phase 2b randomized trial

**DOI:** 10.1038/s41467-026-78584-9

**Published:** 2026-11-13

**Authors:** Catherine Small, Deepali Kumar, Anum Abbas, Ana Sánchez Fructuoso, Shigeyoshi Yamanaga, Virginia Luz Pérez González, Hristos Karakizlis, Michael Nissen, Pyoeng Gyun Choe, Vera Paloschi, Steven Chadban, Hiwot Amare Hailemariam, Catherine Gerard, Marie-Pierre David, Dominique Descamps, Corinne Vandermeulen, Kemi Oluwayi, Veronica Hulstrøm

**Affiliations:** 1 Weill Cornell Medicine, New York, NY USA; 2 University Health Network, Toronto, ON Canada; 3 University of Nebraska Medical Center, Omaha, NE USA; 4 Hospital Clínico San Carlos and Universidad Complutense de Madrid, Madrid, Spain; 5 Japanese Red Cross Kumamoto Hospital, Kumamoto, Japan; 6 Hospital Universitario 12 de Octubre, Madrid, Spain; 7 Justus Liebig University Giessen, Giessen, Germany; 8 The Prince Charles Hospital, Metro North Health, Herston, Australia; 9 Royal Brisbane & Women’s Hospital, Metro North Health, Brisbane, Australia; 10 Frazer Institute, Faculty of Medicine, University of Queensland, Brisbane, Australia; 11 Seoul National University Hospital, Seoul, South Korea; 12 IRCCS San Raffaele Hospital, Milan, Italy; 13 Royal Prince Alfred Hospital and University of Sydney, Sydney, Australia; 14 GSK, Wavre, Belgium; 15 GSK, Rixensart, Belgium; 16 GSK, London, UK

**Keywords:** Peptide vaccines, Viral infection, Transplant immunology

## Abstract

Immunocompromised adults are at increased risk of severe respiratory syncytial virus (RSV) disease. This multicountry, open-label phase 2b trial (NCT05921903) evaluated the immunogenicity and safety of the AS01_E_-adjuvanted RSV prefusion F protein vaccine (adjuvanted RSVPreF3) over 12 months in kidney or lung transplant recipients ≥18 years, randomized 1:1 to receive one (*n *= 131) or two doses 30–60 days apart (*n *= 130). A control group of non-immunocompromised adults ≥50 years (*n *= 125) received a single dose. The primary outcome was the mean geometric increase in RSV-A and RSV-B neutralizing titers one month following a second adjuvanted RSVPreF3 dose relative to the first dose in transplant recipients. Secondary outcomes included assessment of the safety and immunogenicity of adjuvanted RSVPreF3 in transplant recipients compared with the control group. In transplant recipients receiving two doses, neutralizing titers against RSV-A and RSV-B were numerically higher one month post-dose 2 than post-dose 1 (mean geometric increases: 1.34 [95% CI: 1.20, 1.48] for RSV-A [*n *= 111] and 1.26 [95% CI: 1.14, 1.40] for RSV-B [*n *= 111]). A single dose was immunogenic in transplant recipients, but showed slower and generally lower initial neutralizing responses than in non-immunocompromised controls, specifically in individuals receiving mycophenolate. RSVPreF3-specific CD4 + T-cell frequencies increased after vaccination versus baseline in all groups. Humoral and cell-mediated immune responses remained above baseline for 12 months post-vaccination. Both doses of adjuvanted RSVPreF3 were well tolerated and had a similar and acceptable safety profile. Overall, these results support a favorable benefit-risk profile of adjuvanted RSVPreF3 in kidney and lung transplant recipients.

## Abstract (web summary)

The authors report results from a randomized phase 2 trial showing that the adjuvanted RSVPreF3 vaccine was immunogenic and had an acceptable safety profile in lung and kidney transplant recipients, supporting a favorable benefit-risk profile.

## Introduction

Common respiratory viruses, including respiratory syncytial virus (RSV), impose a substantial morbidity and mortality risk to individuals with immunocompromising conditions^1–4^. These conditions, including primary immunodeficiencies, long-term (post-transplant) immunosuppressive therapy, hematological malignancies, or human immunodeficiency virus infection, among others, increase susceptibility to prolonged and more severe RSV disease^4–7^. Pneumonia and hospitalization affect over one third of immunocompromised individuals with RSV^7^. Moreover, affected individuals frequently experience long-term sequelae; a systematic review reported that nearly half of RSV-infected lung transplant recipients experience declines in lung function^8^. This group also remains at high risk of RSV-associated graft complications (acute cellular rejection, graft dysfunction, or chronic lung allograft dysfunction)^8^. RSV-related mortality is considerable; among hematopoietic stem cell transplant recipients hospitalized for RSV, median all-cause mortality reached 17.5%^6^, and among adult bone marrow transplant recipients, the pooled RSV-related case fatality rate was 6.0%^9^.

Therapeutic options for RSV disease in adults remain largely supportive, underscoring the importance of preventive strategies such as vaccination for at-risk groups^10^. However, vaccine responses are often diminished in immunocompromised populations, as observed with severe acute respiratory syndrome coronavirus 2 (SARS-CoV-2), influenza, and other vaccines^11–17^. In transplant recipients, specific immunosuppressive regimens, particularly mycophenolate compounds, are key contributors to reduced immunogenicity^13–17^. Additional vaccine doses may then help improve and sustain protection^18–23^.

Prefusion F protein-based vaccines represent a major advance in RSV prevention. The RSV F protein, the antigenic target of these vaccines, is highly conserved across the two distinct RSV subtypes, RSV-A and RSV-B^24,25^, both of which may co-circulate, often shifting in predominance across locations and seasons^26^. Because RSV-A and RSV-B share this antigen, a monovalent F protein-based vaccine can confer subtype-spanning protection. Moreover, the prefusion form of the F protein is responsible for most of the neutralizing antibody activity^25^. The AS01_E_-adjuvanted RSV prefusion F protein vaccine (adjuvanted RSVPreF3, Arexvy, GSK) protects against disease caused by the RSV-A and RSV-B subtypes in adults^27^. In the phase 1/2 dose-selection study in non-immunocompromised older adults, adjuvanted RSVPreF3 elicited robust immune responses after a single dose, with no additional immunogenic benefit observed after a second dose administered two months later^28^, supporting a single-dose primary regimen in this population. Adjuvanted RSVPreF3 was initially approved in the United States (US) and Europe as a single-dose vaccine for ≥60-year-olds and for 50–59-year-olds at increased risk of severe RSV disease^29,30^. The indication has since been expanded to include adults aged ≥18 years at increased risk of severe RSV disease in the US (March 2026)^29^, and all adults aged ≥18 years in Europe (January 2026)^30^. In ≥60-year-olds, cumulative efficacy of one adjuvanted RSVPreF3 dose against RSV-related lower respiratory tract disease was 62.9% (97.5% confidence interval [CI]: 46.7, 74.8) over three RSV seasons^31^, with recent real-world effectiveness studies corroborating these findings by showing reduced RSV-related hospitalizations after a single RSV vaccine dose^32–37^. Vaccine efficacy remained high in ≥60-year-olds with chronic conditions that increase the risk of severe RSV disease (e.g., lung disease, cardiovascular disease, diabetes), and immunogenicity was comparable to that observed in individuals without such conditions^38,39^. In 50–59-year-olds with or without such chronic conditions^40^, and in 18–49-year-olds with similar conditions^41^, immunogenicity of adjuvanted RSVPreF3 was non-inferior to that in ≥60-year-olds for both RSV-A and RSV-B neutralizing titers. The vaccine demonstrated an acceptable safety profile in these age groups^31,40–42^.

For individuals with immunocompromising conditions, emerging real-world data and immunogenicity studies suggest prefusion F protein-based RSV vaccines can reduce RSV-related hospitalization and elicit immune responses^32–37,43–48^. However, available evidence in this population remains limited, often combines heterogeneous patient groups, and is observational in nature.

Here, we present the results of a multicountry, multicenter trial (NCT05921903) that evaluated the immunogenicity, reactogenicity, and safety of adjuvanted RSVPreF3 in ≥18-year-old lung or kidney transplant recipients receiving maintenance immunosuppressive regimens, following either a single-dose or two-dose regiment administered 30–60 days apart. Non-immunocompromised individuals aged ≥50 years served as single-dose controls. Given the potential suboptimal immune responses in immunocompromised individuals, the primary study objective was to assess whether immune responses in transplant recipients were higher after a second dose than after the first dose. Secondary objectives were to describe the safety and immunogenicity of adjuvanted RSVPreF3 in transplant recipients compared with non-immunocompromised controls. The study also evaluated the immunogenicity of the vaccine in transplant recipients receiving mycophenolate-containing versus mycophenolate-free regimens. Participants were followed for 12 months post-vaccination to assess long-term immunogenicity and safety.

**Fig. 1:**
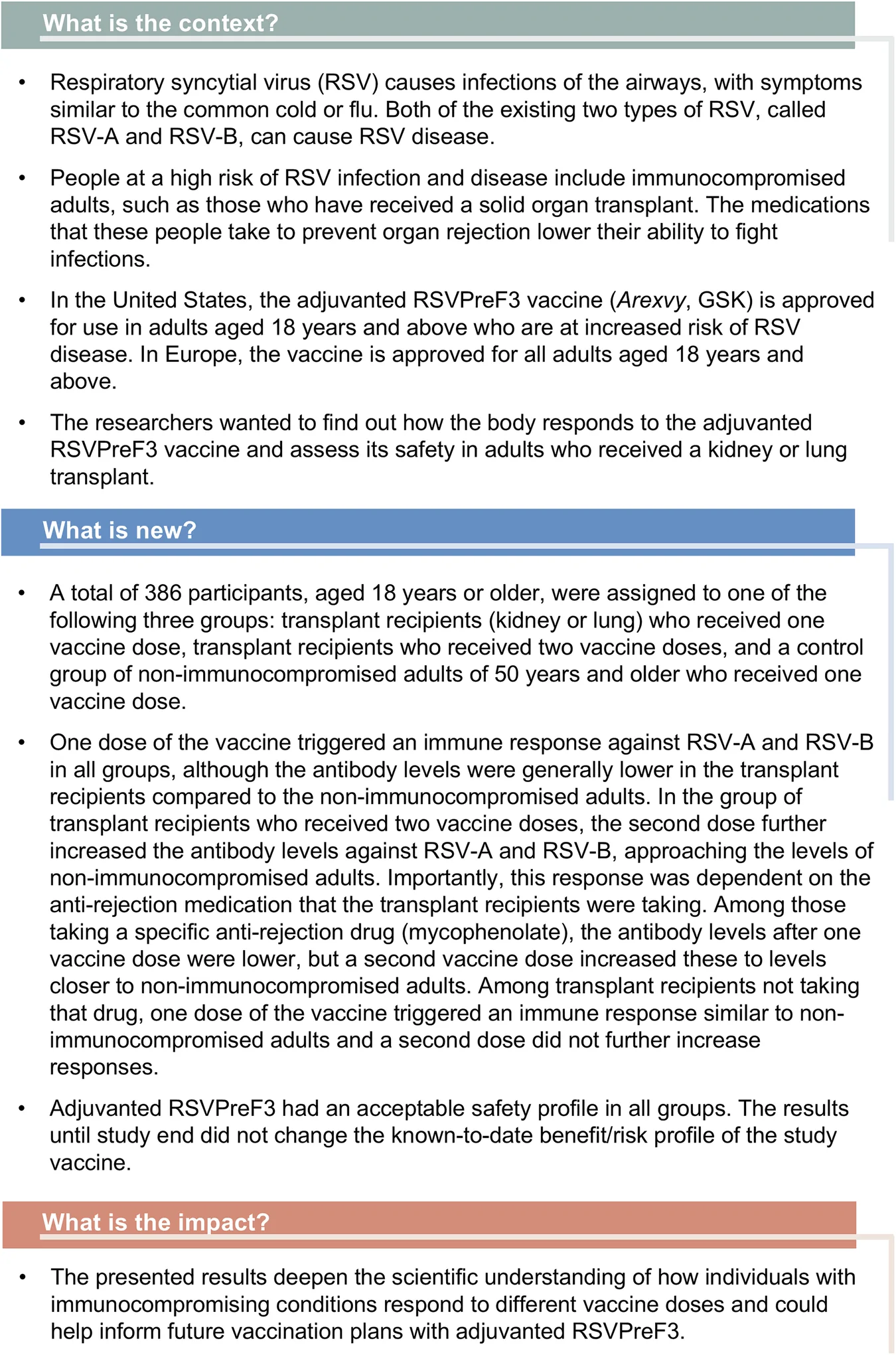
Plain language summary. Overview of the study and its main findings in plain language.

A plain language summary is provided in Fig. 1.

## Results

### Study participants

**Fig. 2:**
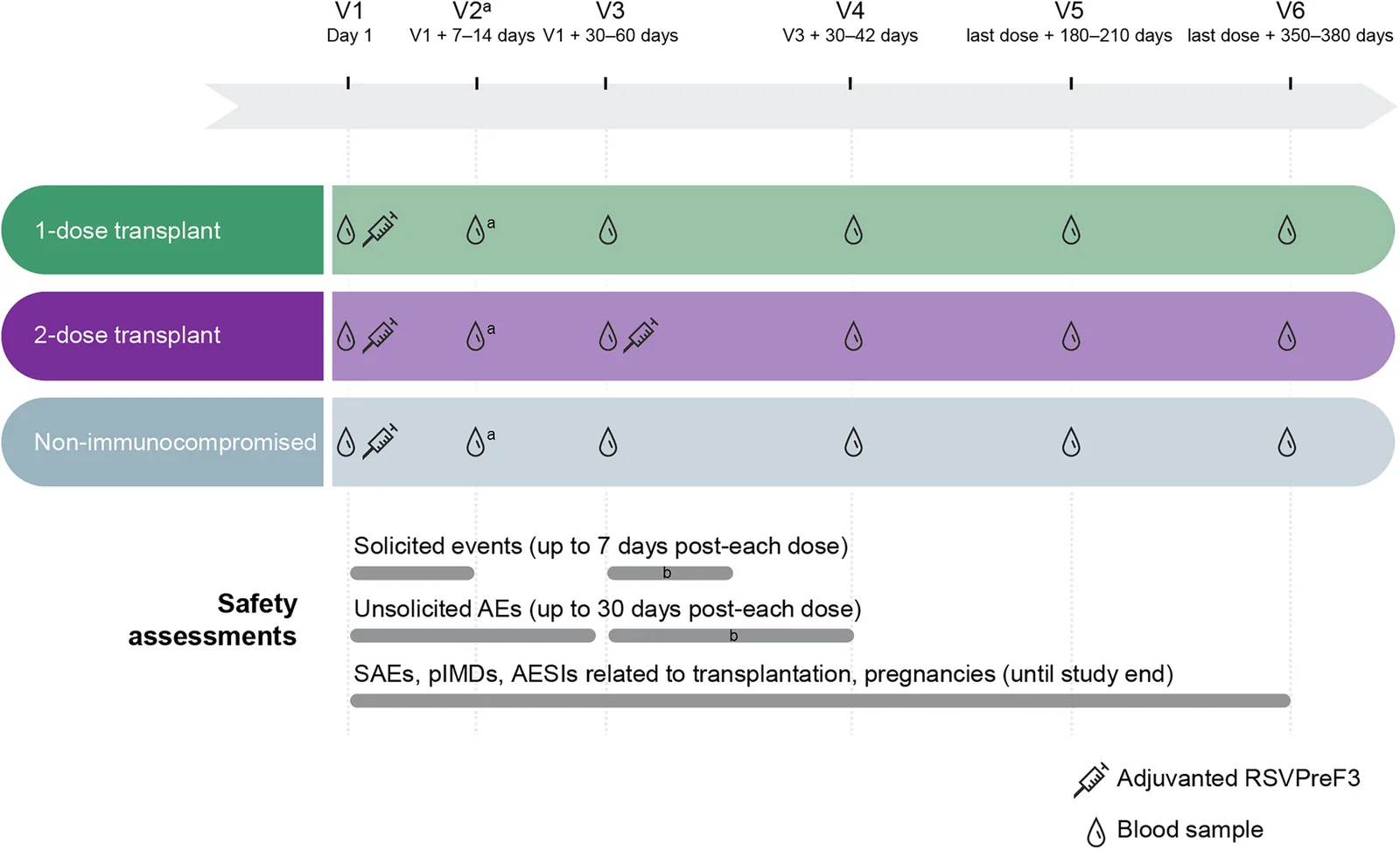
Study design. Schematic representation of the clinical trial design showing the study groups, time intervals, treatment schedules, immunogenicity blood sampling, and safety assessments. V, visit; 1-dose transplant group, immunocompromised kidney or lung transplantation recipients aged ≥18 years who received one dose of AS01_E_-adjuvanted respiratory syncytial virus prefusion F protein vaccine (adjuvanted RSVPreF3); 2-dose transplant group, immunocompromised kidney or lung transplantation recipients aged ≥18 years who received two doses of adjuvanted RSVPreF3 one to two months apart; non-immunocompromised group, non-immunocompromised individuals aged ≥50 years who received one dose of adjuvanted RSVPreF3; AE, adverse event; SAE, serious AE; pIMD, potential immune-mediated disease; AESI, AE of special interest. ^a^Only applicable to a subset of participants in each group (cell-mediated immunogenicity subset). ^b^Only applicable to the 2-dose transplant group.

**Fig. 3:**
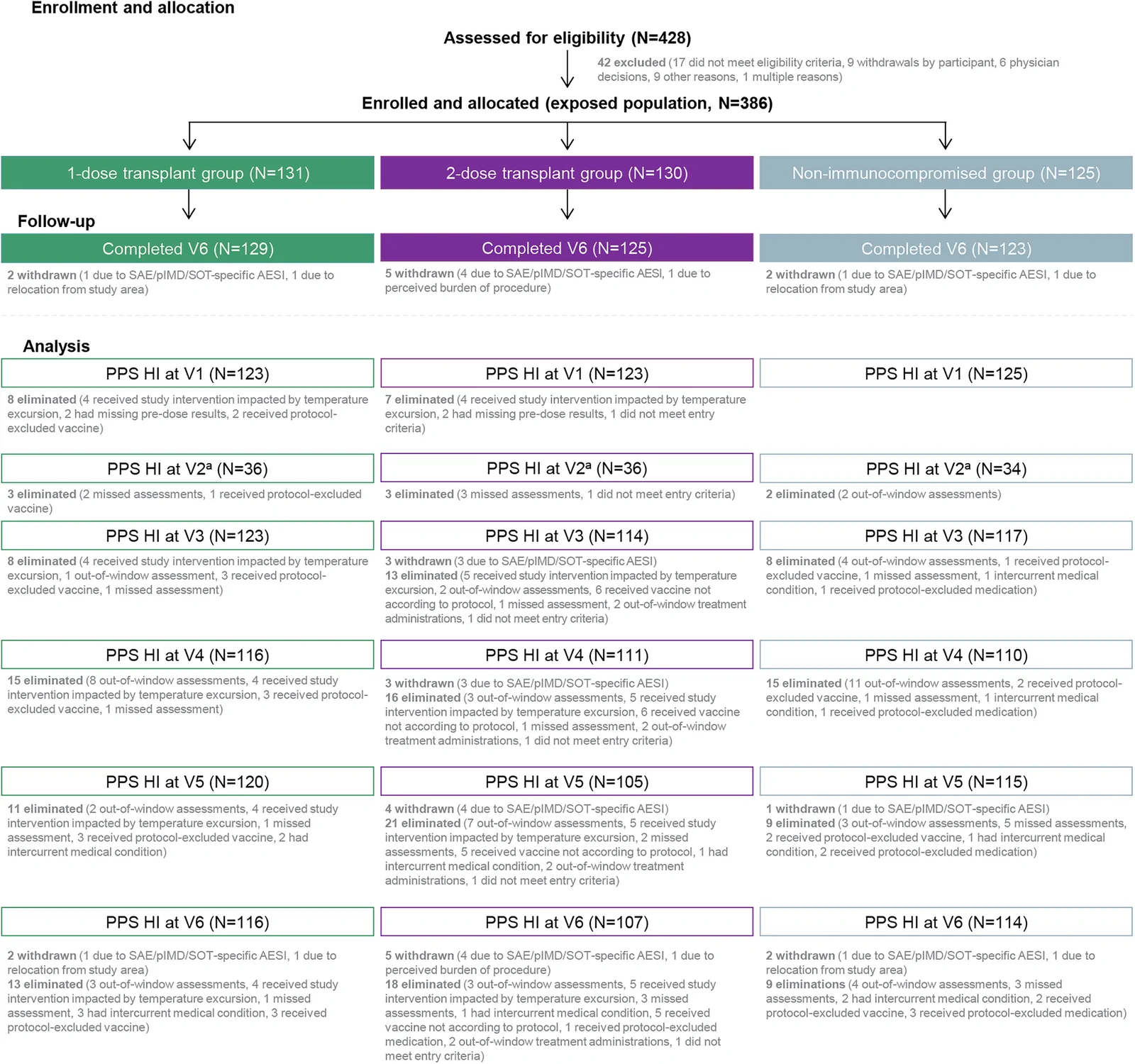
Participant flow. Enrollment and follow-up of the study participants. Multiple reasons for eliminations could apply. The exposed population included all the participants who received at least one vaccine dose. The per-protocol set for humoral immunogenicity (PPS HI) included all participants who received the study intervention administration as per protocol, had pre- and post-dose immunogenicity results, complied with blood draw intervals, had no intercurrent conditions that may interfere with immunogenicity (other than those inherent to the transplant population), and did not receive prohibited medication/vaccination. By study end, six participants were withdrawn from the study due to SAEs; none of these events were considered vaccine-related by the investigator. N, number of participants in the indicated analysis set, group, and timepoint; 1-dose transplant group, immunocompromised kidney or lung transplantation recipients aged ≥18 years who received one dose of AS01_E_-adjuvanted respiratory syncytial virus prefusion F protein vaccine (adjuvanted RSVPreF3); 2-dose transplant group, immunocompromised kidney or lung transplantation recipients aged ≥18 years who received two doses of adjuvanted RSVPreF3 one to two months apart; non-immunocompromised group, non-immunocompromised individuals aged ≥50 years who received one dose of adjuvanted RSVPreF3; V, visit; AE, adverse event; SAE, serious AE; pIMD, potential immune-mediated disease; SOT-specific AESI, AE of special interest specific for solid organ transplant recipients. ^a^Only applicable for a subset of participants in each group (cell-mediated immunogenicity subset).

The study enrolled two populations: immunocompromised individuals and non-immunocompromised individuals, with follow-up until 12 months after (last) vaccination across five to six predefined visits to assess immunogenicity and safety (Fig. 2). The immunocompromised cohort comprised lung or kidney transplant recipients aged ≥18 years who were at least one-year post-transplant and received maintenance therapy for prevention of allograft rejection. These participants were randomized 1:1 to receive one dose at Visit 1 (1-dose transplant group, *n *= 131) or two doses 30–60 days apart at Visit 1 and Visit 3 (2-dose transplant group, *n *= 130) of adjuvanted RSVPreF3. Non-immunocompromised adults ≥50 years served as controls and received one dose at Visit 1 (non-immunocompromised group, *n *= 125). A subset of participants ( ~ 30%) in each group, recruited from a selected number of countries and clinical sites, contributed to cell-mediated immunogenicity assessments and had an additional early post-dose 1 visit (Visit 2) (Fig. 2). In total, 386 participants received at least one vaccine dose, and 377 (97.7%) participants completed the study (Fig. 3).

The baseline characteristics in the exposed population (all participants who received at least one dose) were generally similar across study groups, except for medical conditions, as per eligibility criteria (Table 1). The median age was 63.0 years in the 1-dose transplant group, 62.0 years in the 2-dose transplant group, and 64.0 years in the non-immunocompromised group. In the 1-dose and 2-dose transplant groups, 70.2% and 72.3% of participants, respectively, had a kidney transplant; the remainder had a lung transplant. The median interval between last transplantation and Visit 1 was balanced between the transplant groups (1-dose transplant group: 67 months [min–max: 12–386], and 2-dose transplant group: 60 months [14–450]). Overall, 59.4% of transplant recipients were on immunosuppressive regimens that included a combination of mycophenolate compounds, corticosteroids, and calcineurin inhibitors (and/or mechanistic target of rapamycin [mTOR] inhibitors); 36.8% were on regimens containing any combination of two among mycophenolate compounds, corticosteroids, and calcineurin inhibitors (and/or mTOR inhibitors), while 3.8% were on a regimen containing a single component (mycophenolate compounds, corticosteroids, or calcineurin inhibitors [and/or mTOR inhibitors]). Nearly all transplant recipients (99.6%) were on regimens containing calcineurin inhibitors (and/or mTOR inhibitors); 79.3% were on corticosteroid-containing regimens, and 76.6% on mycophenolate-containing regimens. For purposes of this study, a small subset of participants on mTOR inhibitors were co-classified with those on calcineurin inhibitors.

**Table 1:** Baseline characteristics of the study participants (exposed population)

Characteristic	1-dose transplant *N* = 131	2-dose transplant *N* = 130	Non-immunocompromised *N* = 125
Age at vaccination^a^, yr			
Median (IQR) age^a^, yr	63.0 (55.5–68.0)	62.0 (56.0–66.8)	64.0 (57.0–70.0)
18–49 yr, *n* (%)	9 (6.9)	8 (6.2)	NA
Median (IQR) age^a^, yr	45.0 (42.0–45.0)	45.5 (38.8–48.0)	NA
50–59 yr, *n* (%)	36 (27.5)	41 (31.5)	44 (35.2)
Median (IQR) age^a^, yr	54.0 (51.0–57.0)	55.0 (53.0–57.0)	55.5 (53.0–57.0)
≥60 yr, *n* (%)	86 (65.6)	81 (62.3)	81 (64.8)
Median (IQR) age^a^, yr	67.0 (63.0–72.0)	65.0 (62.0–70.0)	69.0 (64.0–72.0)
Female sex, *n* (%)	54 (41.2)	50 (38.5)	66 (52.8)
Race^b^, *n* (%)			
White	89 (67.9)	79 (60.8)	95 (76.0)
Other	42 (32.1)	51 (39.2)	30 (24.0)
Mean (SD) BMI^c^, kg/m^2^	27.2 (5.4)	27.5 (6.4)	27.5 (5.4)
Country, *n* (%)			
United States	39 (29.8)	33 (25.4)	32 (25.6)
Spain	33 (25.2)	19 (14.6)	30 (24.0)
Japan	23 (17.6)	29 (22.3)	9 (7.2)
Canada	10 (7.6)	16 (12.3)	21 (16.8)
Italy	12 (9.2)	13 (10.0)	3 (2.4)
Australia	7 (5.3)	10 (7.7)	9 (7.2)
South Korea	3 (2.3)	6 (4.6)	15 (12.0)
Germany	4 (3.1)	4 (3.1)	6 (4.8)
Transplant type, *n* (%)			
Kidney transplant	92 (70.2)	94 (72.3)	NA
Lung transplant	39 (29.8)	36 (27.7)	NA
Median (min–max) interval between last transplant and Visit 1 (months)	67 (12–386)	60 (14–450)	NA
Number of transplantations, *n* (%)			
1	124 (94.7)	121 (93.1)	NA
2	7 (5.3)	6 (4.6)	NA
3	0 (0.0)	3 (2.3)	NA
Number of rejections, *n* (%)			
0	117 (89.3)	108 (83.1)	NA
1	14 (10.7)	22 (16.9)	NA
Immunosuppressive regimen, *n* (%)			
CS + MC + CNI/mTORi	75 (57.3)	80 (61.5)	NA
Any 2 among CS, MC, CNI/mTORi	52 (39.7)	44 (33.8)	NA
CS or MC or CNI/mTORi	4 (3.1)	6 (4.6)	NA
CS^d^	103 (78.6)	104 (80.0)	NA
No CS^d^	28 (21.4)	26 (20.0)	NA
MC	100 (76.3)	100 (76.9)	NA
No MC	31 (23.7)	30 (23.1)	NA
CNI/mTORi^d^	130 (99.2)	130 (100)	NA
No CNI/mTORi^d^	1 (0.8)	0 (0.0)	NA

1-dose transplant group, immunocompromised kidney or lung transplantation recipients aged ≥18 years who received one dose of AS01_E_-adjuvanted respiratory syncytial virus prefusion F protein vaccine (adjuvanted RSVPreF3); 2-dose transplant group, immunocompromised kidney or lung transplantation recipients aged ≥18 years who received two doses of adjuvanted RSVPreF3 one to two months apart; non-immunocompromised group, non-immunocompromised individuals aged ≥50 years who received one dose of adjuvanted RSVPreF3.

*N* number of participants in the exposed population, *yr* years, *IQR* interquartile range, *n (%)* number (percentage) of participants in a given category, *SD* standard deviation, *BMI* body mass index, *NA* not applicable, *CS* corticosteroids, *MC* mycophenolate compounds, *CNI/mTORi* calcineurin inhibitors and/or mTOR inhibitors (pooled for analysis purposes).

^a^Age was computed based on incomplete date of birth (only year was available). Median age and IQR age by age category were calculated following a post-hoc analysis.

^b^Race was self-reported by the participants. ‘Other’ includes American Indian or Alaska Native, Asian, Black or African American, Multiple, Not Reported, and Unknown.

^c^BMI data from four participants in the 2-dose transplant group were not available.

^d^The number of participants who received CS and CNI and/or mTORi were retained following a post-hoc analysis.

At each Visit, more than 80% of participants across groups were included in the per-protocol set for humoral immunogenicity (all participants who received the study intervention administration as per protocol, had pre- and post-dose immunogenicity results, complied with blood draw intervals, had no intercurrent conditions that may interfere with immunogenicity (other than those inherent to the transplant population), or did not receive prohibited medication/vaccination) (Fig. 3). Baseline characteristics of the per-protocol set for humoral immunogenicity at Visit 1 were generally similar to those of the exposed population. Within the subgroups defined by mycophenolate compound use (with vs without), characteristics were generally balanced (Supplementary Table 1). The cell-mediated immunogenicity subset at Visit 1 was broadly comparable to the overall immunogenicity cohort (per-protocol set for humoral immunogenicity) in terms of key baseline characteristics, despite its smaller sample size.

All transplant recipients had additional underlying medical conditions, compared with 75.2% of non-immunocompromised participants, based on Medical Dictionary for Regulatory Activities (MedDRA) classification. These comorbidities were diverse and spanned multiple MedDRA System Organ Classes (SOCs) in both groups. Specifically, in the transplant groups, Metabolism and nutrition disorders was the most common SOC (83.2%–84.6%), and in the non-immunocompromised group, Vascular disorders was the most prevalent SOC (38.4%). A complete distribution of comorbidities across main SOCs by study group is available in Supplementary Table 2.

### Primary descriptive endpoint: RSV-A and RSV-B serum neutralizing titers expressed as mean geometric increases (MGIs) following a second dose relative to the first dose in transplant recipients

MGIs in RSV-A and RSV-B neutralizing titers (i.e., geometric mean of the within-participant ratios of neutralizing titers) approximately one month post-dose 2 (Visit 4) relative to approximately one-month post-dose 1 (Visit 3) were calculated for the 2-dose transplant group.

**Fig. 4:**
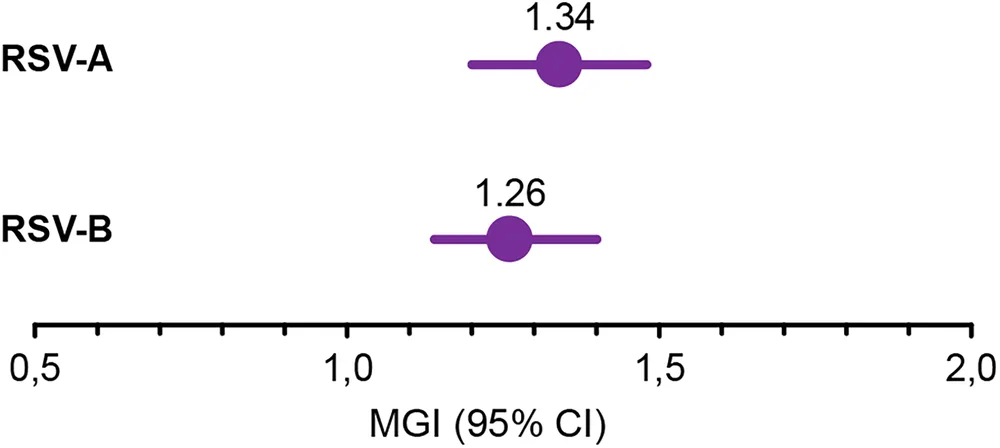
Mean geometric increases of RSV-A and RSV-B neutralizing titers 1-month post-dose 2 over 1-month post-dose 1 in transplant recipients (2-dose transplant group per-protocol set for humoral immunogenicity at Visit 3 and Visit 4). Mean geometric increases (MGIs) were calculated as Visit 4 titers divided by Visit 3 titers from the 2-dose transplant group. Visit 3 corresponds to approximately 1–2 months post-dose 1; Visit 4 corresponds to approximately 1 month post-dose 2. For both analyses (RSV-A and RSV-B), 111 participants in the 2-dose transplant group had immunogenicity data available at both Visit 3 and Visit 4. RSV, respiratory syncytial virus; CI, confidence interval; 2-dose transplant group, immunocompromised kidney or lung transplantation recipients aged ≥18 years who received two doses of AS01_E_-adjuvanted RSV prefusion F protein vaccine (adjuvanted RSVPreF3) 1–2 months apart.

MGIs post-dose 2 over post-dose 1 were 1.34 (95% CI: 1.20, 1.48) for RSV-A, and 1.26 (1.14, 1.40) for RSV-B, indicating that in transplant recipients, a second dose induced higher neutralizing titers than the first dose (*n *= 111 in the RSV-A and RSV-B per-protocol sets with immunogenicity data available at both timepoints) (Fig. 4). Results in the exposed population were similar.

### Humoral immunogenicity kinetics

**Fig. 5:**
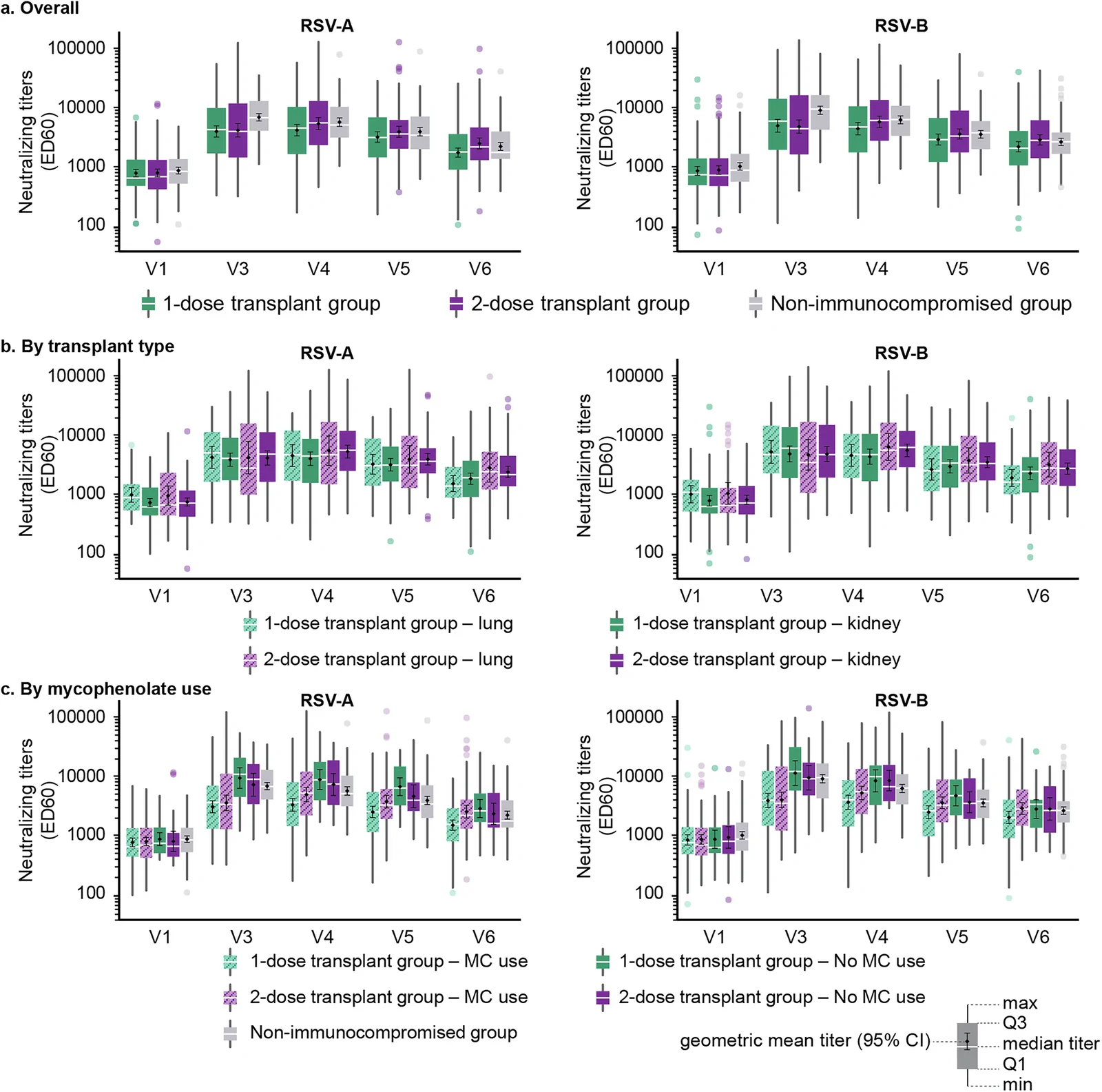
RSV-A and RSV-B neutralizing titers (per-protocol set for humoral immunogenicity). **a** RSV-A and RSV-B neutralizing titers in the three study groups before (first) vaccination (V1) and up to 12 months after (last) vaccination (V3, 4, 5, and 6). In the 2-dose transplant group, the second vaccine dose was administered at V3. V3 titers were measured before dose 2. The 1-dose transplant group is shown in green, the 2-dose transplant group in purple, and the non-immunocompromised control group in grey. Sample sizes at each visit (V1/V3/V4/V5/V6) were as follows: 1-dose transplant group (123/123/116/119/116 [RSV-A] or 123/123/116/120/116 [RSV-B]); 2-dose transplant group (123/114/111/105/107 [RSV-A/RSV-B]); and non-immunocompromised controls (125/117/110/115/114 [RSV-A/RSV-B]). **b** RSV-A and RSV-B neutralizing titers in the two transplant groups, stratified by transplant type. **c** RSV-A and RSV-B neutralizing titers in the three study groups, with transplant groups stratified by mycophenolate compound (MC) use. Neutralizing titers are expressed as ED60. The lower limits of quantification were 18 ED60 for RSV-A and 30 ED60 for RSV-B neutralizing titers. Geometric mean titers and 95% CIs at each Visit were assessed as part of the study secondary objectives; median neutralizing titers and interquartile ranges (Q1–Q3) at each Visit were calculated following a post-hoc analysis. RSV, respiratory syncytial virus; ED60, estimated dilution 60; 1-dose transplant group, immunocompromised kidney or lung transplantation recipients aged ≥18 years who received one dose of AS01_E_-adjuvanted RSV prefusion F protein vaccine (adjuvanted RSVPreF3); 2-dose transplant group, immunocompromised kidney or lung transplantation recipients aged ≥18 years who received two doses of adjuvanted RSVPreF3 one to two months apart; non-immunocompromised group, non-immunocompromised individuals aged ≥50 years who received one dose of adjuvanted RSVPreF3; MC, mycophenolate compound; CI, confidence interval; Q1, 25^th^ percentile; Q3, 75^th^ percentile. Source data are provided as a Source Data file.

At baseline (Visit 1, pre-vaccination), all participants had RSV-A and RSV-B neutralizing titers above assay cut-offs (18 estimated dilution [ED60] for RSV-A and 30 ED60 for RSV-B), reflecting prior RSV exposure. RSV-A and RSV-B geometric mean titers (GMTs) were within a similar range across groups, ranging 807.0–889.0 ED60 for RSV-A and 864.2–1027.3 ED60 for RSV-B (Fig. 5a and Table 2).

**Table 2:** RSV-A and RSV-B neutralizing titers (per-protocol set for humoral immunogenicity)

		1-dose transplant		2-dose transplant		Non-immunocompromised	
Timepoint	Variable	*N*	Value or % (95% CI)	*N*	Value or % (95% CI)	*N*	Value or % (95% CI)
**RSV-A neutralizing titers**							
Visit 1	GMT	123	807.0 (700.9–929.2)	123	814.1 (701.3–945.1)	125	889.0 (781.9–1010.6)
Visit 2	GMT	36	1775.8 (1201.0–2625.6)	36	2031.1 (1283.6–3213.9)	34	6716.8 (4907.6–9193.0)
	MGI	36	2.14 (1.44–3.18)	36	2.20 (1.45–3.34)	34	7.59 (5.44–10.58)
	SRR	9	25.0 (12.1–42.2)	9	25.0 (12.1–42.2)	21	61.8 (43.6–77.8)
Visit 3	GMT	123	3990.8 (3219.4–4947.0)	114	4174.0 (3230.7–5392.6)	117	6881.3 (5976.2–7923.6)
	MGI	122	4.92 (3.95–6.12)	113	5.01 (4.00–6.27)	117	7.90 (6.82–9.15)
	SRR	66	54.1 (44.8–63.2)	60	53.1 (43.5–62.5)	96	82.1 (73.9–88.5)
Visit 4	GMT	116	4184.7 (3360.1–5211.7)	111	5377.9 (4265.4–6780.6)	110	5707.0 (4853.6–6710.3)
	MGI	114	5.29 (4.24–6.60)	110	6.53 (5.32–8.01)	110	6.55 (5.60–7.66)
	SRR	64	56.1 (46.5–65.4)	72	65.5 (55.8–74.3)	80	72.7 (63.4–80.8)
Visit 5	GMT	119	3220.8 (2637.4–3933.3)	105	3954.5 (3205.4–4878.6)	115	3958.4 (3372.2–4646.6)
	MGI	118	4.16 (3.42–5.06)	104	4.71 (3.87–5.72)	115	4.46 (3.86–5.15)
	SRR	58	49.2 (39.8–58.5)	56	53.8 (43.8–63.7)	65	56.5 (47.0–65.7)
Visit 6	GMT	116	1773.4 (1486.2–2116.2)	107	2517.4 (2039.3–3107.5)	114	2243.6 (1925.0–2614.9)
	MGI	115	2.24 (1.87–2.69)	106	3.01 (2.54–3.56)	114	2.58 (2.24–2.97)
	SRR	32	27.8 (19.9–37.0)	40	37.7 (28.5–47.7)	30	26.3 (18.5–35.4)
**RSV-B neutralizing titers**							
Visit 1	GMT	123	864.2 (727.8–1026.2)	123	892.9 (754.1–1057.3)	125	1027.3 (889.6–1186.3)
Visit 2	GMT	36	2325.3 (1592.9–3394.5)	36	2603.1 (1714.9–3951.3)	34	8113.6 (5699.9–11549.5)
	MGI	36	2.51 (1.78–3.55)	36	2.54 (1.75–3.67)	34	8.59 (6.59–11.20)
	SRR	12	33.3 (18.6–51.0)	11	30.6 (16.3–48.1)	29	85.3 (68.9–95.0)
Visit 3	GMT	123	5002.9 (3901.7–6414.8)	114	4834.7 (3736.6–6255.6)	117	9125.2 (7781.9–10700.5)
	MGI	122	5.72 (4.53–7.21)	113	5.34 (4.25–6.72)	117	8.95 (7.81–10.25)
	SRR	71	58.2 (48.9–67.1)	60	53.1 (43.5–62.5)	102	87.2 (79.7–92.6)
Visit 4	GMT	116	4461.7 (3519.0–5657.0)	111	5820.8 (4658.2–7273.5)	110	6281.6 (5377.6–7337.7)
	MGI	114	5.22 (4.20–6.49)	110	6.52 (5.34–7.97)	110	6.35 (5.50–7.33)
	SRR	68	59.6 (50.1–68.7)	71	64.5 (54.9–73.4)	79	71.8 (62.4–80.0)
Visit 5	GMT	120	2926.9 (2364.0–3623.8)	105	3620.7 (2974.9–4406.7)	115	3584.5 (3093.6–4153.3)
	MGI	119	3.52 (2.92–4.25)	104	3.95 (3.38–4.61)	115	3.59 (3.13–4.10)
	SRR	58	48.7 (39.5–58.1)	48	46.2 (36.3–56.2)	52	45.2 (35.9–54.8)
Visit 6	GMT	116	2206.7 (1811.3–2688.5)	107	2886.5 (2368.0–3518.4)	114	2665.1 (2310.9–3073.5)
	MGI	115	2.58 (2.20–3.04)	106	3.17 (2.73–3.68)	114	2.69 (2.35–3.08)
	SRR	35	30.4 (22.2–39.7)	39	36.8 (27.6–46.7)	34	29.8 (21.6–39.1)

RSV-A and RSV-B neutralizing titers in the three study groups before (first) vaccination (V1) and up to 12 months after (last) vaccination (V2, 3, 4, 5, and 6). In the 2-dose transplant group, the second vaccine dose was administered at V3. V3 titers were measured before dose 2. 1-dose transplant group, immunocompromised kidney or lung transplantation recipients aged ≥18 years who received one dose of AS01_E_-adjuvanted RSV prefusion F protein vaccine (adjuvanted RSVPreF3); 2-dose transplant group, immunocompromised kidney or lung transplantation recipients aged ≥18 years who received two doses of adjuvanted RSVPreF3 one to two months apart; non-immunocompromised group, non-immunocompromised individuals aged ≥50 years who received one dose of adjuvanted RSVPreF3. Source data are provided as a Source Data file.

*N* number of participants with available results at the given timepoint (GMT), or with available results at both the given timepoint and at baseline (MGI), or with a ≥4-fold increase in neutralizing titers compared to baseline (SRR), *CI* confidence interval, *GMT* geometric mean titer, *MGI* mean geometric increase (post-dose titer compared to baseline titer), *RSV*, respiratory syncytial virus, *SRR* seroresponse rate.

At Visit 2 (i.e., 7–14 days post-dose 1), in the cell-mediated immunogenicity subset, RSV-A and RSV-B neutralizing GMT point estimates were higher than at baseline in all groups, but were numerically lower in both transplant groups compared to the non-immunocompromised group (Table 2).

At Visit 3 (i.e., 30–60 days post-dose 1), higher GMT values were observed compared to Visit 2 across the transplant groups, while those in the non-immunocompromised group were within a similar range to Visit 2 (Fig. 5a and Table 2). GMT point estimates in the transplant groups remained numerically lower than those in the non-immunocompromised group. For RSV-A, GMTs were 3990.8 ED60 (1-dose transplant), 4174.0 ED60 (2-dose transplant), and 6881.3 ED60 (non-immunocompromised); for RSV-B, 5002.9 ED60 (1-dose transplant), 4834.7 ED60 (2-dose transplant), and 9125.2 ED60 (non-immunocompromised).

At Visit 4 (i.e., 30–42 days post-dose 2 in the 2-dose transplant group, or two to three months post-dose 1 in the other groups), GMT point estimates in the non-immunocompromised group were lower than at Visit 3 but remained above baseline (RSV-A: 5707.0 ED60; RSV-B: 6281.6 ED60) (Fig. 5a and Table 2). In the 1-dose transplant group, GMT point estimates were in a similar range to those observed at Visit 3 (RSV-A: 4184.7 ED60; RSV-B: 4461.7 ED60). In the 2-dose transplant group, numerically higher GMTs were observed following the second dose relative to Visit 3, with values in a similar range to those in the non-immunocompromised group (RSV-A: 5377.9 ED60; RSV-B: 5820.8 ED60).

In all groups, the RSV-A and RSV-B neutralizing GMT point estimates were lower at later visits relative to Visit 4, but remained above baseline up to 12 months post-last dose (Visit 6) (Fig. 5a and Table 2). At Visit 6, GMTs ranged 1773.4–2517.4 ED60 for RSV-A and 2206.7–2886.5 ED60 for RSV-B across groups.

Similar observations were made in terms of MGIs of post-vaccination RSV-A and RSV-B neutralizing titers compared to baseline (Table 2) and for seroresponse rates (SRRs), defined as a ≥ 4-fold increase in neutralizing titers compared to baseline (Table 2 and Supplementary Fig. 1A). Between-group comparisons on humoral immunogenicity

Between-group comparisons of RSV-A and RSV-B neutralizing titers were assessed using adjusted GMT ratios for prespecified group comparisons at each Visit (Supplementary Table 3). One to two months after dose 1 (Visit 3), adjusted GMT ratios (non-immunocompromised over combined transplant group) were 1.63 (95% CI: 1.29, 2.06) for RSV-A and 1.69 (1.33, 2.15) for RSV-B. These observations suggest lower initial neutralizing responses in transplant recipients compared to the non-immunocompromised group following a single vaccine dose.

At Visit 4 (30–42 days after Visit 3), adjusted GMT ratios (non-immunocompromised over 2-dose transplant group) were 1.02 (0.80, 1.30) for RSV-A, and 1.01 (0.80, 1.26) for RSV-B, indicating that the GMT point estimates in the 2-dose transplant group were in a similar range to those in the non-immunocompromised group at this timepoint. In contrast, at the same timepoint, the adjusted GMT ratio (non-immunocompromised over 1-dose transplant group) was 1.31 (1.01, 1.68) for RSV-A and 1.29 (1.00, 1.65) for RSV-B, indicating that GMT point estimates were lower in the 1-dose transplant group compared to the non-immunocompromised group. This pattern was also reflected in the adjusted GMT ratio (2-dose transplant over 1-dose transplant group), which was 1.26 (0.94, 1.69) for RSV-A and 1.28 (0.97, 1.70) for RSV-B at Visit 4.

Over time, between-group differences attenuated, with most ratios (non-immunocompromised over any transplant group) around 1.0 or with CIs including 1.0 across Visits 5 to 6. Similar patterns were observed for between-group assessments in terms of SRR differences (Supplementary Table 3).

### Subgroup analyses of humoral immunogenicity

When the transplant recipients were stratified by transplant subtype, RSV-A and RSV-B GMT point estimates after one and two doses were within a similar range among lung and kidney transplant recipients (Fig. 5b).

When stratified by immunosuppressive regimen, neutralizing responses among transplant recipients showed distinct patterns. Those on regimens that did not include mycophenolate compounds (23.4% of transplant recipients) had initial post-dose 1 RSV-A and RSV-B GMTs within similar ranges to those observed in non-immunocompromised participants (Visit 3), with GMT point estimates after a second dose (Visit 4) in similar ranges to those post-dose 1. In contrast, transplant recipients on mycophenolate compounds (76.6% of transplant recipients) had lower initial post-dose 1 GMT point estimates than those observed in both non-immunocompromised participants and transplant recipients not on mycophenolate compounds. However, GMTs in this subgroup were higher post-dose 2 compared to post-dose 1 in the 2-dose transplant group, and were within similar ranges to those observed in non-immunocompromised participants post-dose 1 (Fig. 5c and Table 3). SRR analyses showed consistent patterns (Supplementary Fig. 1B).

**Table 3:** RSV-A and RSV-B neutralizing titers, with participants in the transplant groups stratified by mycophenolate compound use (per-protocol set for humoral immunogenicity)

		1-dose transplant				2-dose transplant				Non-immunocompromised	
		MC		No MC		MC		No MC			
Timepoint	Variable	*N*	Value or % (95% CI)	*N*	Value or % (95% CI)	*N*	Value or % (95% CI)	*N*	Value or % (95% CI)	*N*	Value or % (95% CI)
**RSV-A neutralizing titers**											
Visit 1	GMT	95	784.6 (662.6–929.0)	28	887.9 (691.9–1139.3)	94	813.1 (694.1–952.5)	29	817.5 (552.5–1209.6)	125	889.0 (781.9–1010.6)
Visit 2	GMT	28	1442.0 (957.2–2172.5)	8	3680.0 (1282.1–10,562.6)	29	1678.9 (1048.5–2688.2)	7	4470.9 (983.3–20,328.3)	34	6716.8 (4907.6–9193.0)
	MGI	28	1.90 (1.25–2.87)	8	3.27 (0.95–11.19)	29	1.79 (1.15–2.78)	7	5.10 (1.56–16.73)	34	7.59 (5.44–10.58)
	SRR	6	21.4 (8.3–41.0)	3	37.5 (8.5–75.5)	5	17.2 (5.8–35.8)	4	57.1 (18.4–90.1)	21	61.8 (43.6–77.8)
Visit 3	GMT	95	3101.4 (2458.6–3912.3)	28	9387.9 (6328.5–13,926.3)	90	3601.8 (2671.9–4855.4)	24	7255.2 (4667.7–11,277.0)	117	6881.3 (5976.2–7923.6)
	MGI	94	3.91 (3.08–4.97)	28	10.57 (6.89–16.24)	89	4.36 (3.39–5.60)	24	8.39 (5.16–13.67)	117	7.90 (6.82–9.15)
	SRR	44	46.8 (36.4–57.4)	22	78.6 (59.0–91.7)	42	47.2 (36.5–58.1)	18	75.0 (53.3–90.2)	96	82.1 (73.9–88.5)
Visit 4	GMT	89	3335.8 (2609.3–4264.6)	27	8835.6 (6020.2–12,967.6)	88	4960.3 (3778.9–6511.0)	23	7327.2 (4811.0–11,159.4)	110	5707.0 (4853.6–6710.3)
	MGI	88	4.35 (3.41–5.55)	26	10.27 (6.57–16.06)	87	6.09 (4.83–7.67)	23	8.50 (5.40–13.38)	110	6.55 (5.60–7.66)
	SRR	42	47.7 (37.0–58.6)	22	84.6 (65.1–95.6)	54	62.1 (51.0–72.3)	18	78.3 (56.3–92.5)	80	72.7 (63.4–80.8)
Visit 5	GMT	90	2539.3 (2034.8–3168.9)	29	6736.0 (4785.6–9481.2)	83	3789.0 (2973.9–4827.5)	22	4646.5 (2987.1–7227.7)	115	3958.4 (3372.2–4646.6)
	MGI	90	3.40 (2.74–4.23)	28	7.91 (5.47–11.43)	82	4.55 (3.68–5.63)	22	5.32 (3.23–8.77)	115	4.46 (3.86–5.15)
	SRR	37	41.1 (30.8–52.0)	21	75.0 (55.1–89.3)	41	50.0 (38.7–61.3)	15	68.2 (45.1–86.1)	65	56.5 (47.0–65.7)
Visit 6	GMT	89	1527.9 (1253.9–1861.7)	27	2898.5 (2044.4–4109.5)	83	2563.9 (2000.0–3286.7)	24	2363.0 (1567.1–3563.0)	114	2243.6 (1925.0–2614.9)
	MGI	89	1.99 (1.62–2.43)	26	3.40 (2.31–5.02)	82	3.09 (2.55–3.75)	24	2.73 (1.89–3.96)	114	2.58 (2.24–2.97)
	SRR	22	24.7 (16.2–35.0)	10	38.5 (20.2–59.4)	29	35.4 (25.1–46.7)	11	45.8 (25.6–67.2)	30	26.3 (18.5–35.4)
**RSV-B neutralizing titers**											
Visit 1	GMT	95	859.0 (703.1–1049.5)	28	882.1 (621.0–1253.1)	94	877.0 (728.9–1055.3)	29	946.3 (624.8–1433.1)	125	1027.3 (889.6–1186.3)
Visit 2	GMT	28	2117.6 (1382.1–3244.3)	8	3226.4 (1189.7–8749.6)	29	2050.1 (1330.5–3158.8)	7	7001.8 (2317.9–21,151.0)	34	8113.6 (5699.9–11,549.5)
	MGI	28	2.26 (1.52–3.36)	8	3.65 (1.65–8.09)	29	2.04 (1.39–2.98)	7	6.32 (2.48–16.14)	34	8.59 (6.59–11.20)
	SRR	10	35.7 (18.6–55.9)	2	25.0 (3.2–65.1)	7	24.1 (10.3–43.5)	4	57.1 (18.4–90.1)	29	85.3 (68.9–95.0)
Visit 3	GMT	95	3931.1 (2985.1–5177.0)	28	11,335.9 (7041.6–18,249.2)	90	4041.4 (3012.1–5422.3)	24	9468.3 (5899.8–15,195.3)	117	9125.2 (7781.9–10,700.5)
	MGI	94	4.49 (3.50–5.76)	28	12.85 (7.97–20.72)	89	4.57 (3.57–5.87)	24	9.52 (5.64–16.05)	117	8.95 (7.81–10.25)
	SRR	50	53.2 (42.6–63.6)	21	75.0 (55.1–89.3)	43	48.3 (37.6–59.2)	17	70.8 (48.9–87.4)	102	87.2 (79.7–92.6)
Visit 4	GMT	89	3677.2 (2800.4–4828.6)	27	8439.9 (5514.7–12,916.9)	88	5274.4 (4062.3–6848.1)	23	8487.5 (5736.0–12,558.8)	110	6281.6 (5377.6–7337.7)
	MGI	88	4.29 (3.37–5.46)	26	10.14 (6.61–15.57)	87	5.99 (4.81–7.46)	23	9.02 (5.60–14.54)	110	6.35 (5.50–7.33)
	SRR	47	53.4 (42.5–64.1)	21	80.8 (60.6–93.4)	53	60.9 (49.9–71.2)	18	78.3 (56.3–92.5)	79	71.8 (62.4–80.0)
Visit 5	GMT	91	2511.8 (1959.7–3219.3)	29	4730.0 (3201.0–6989.4)	83	3606.5 (2869.8–4532.3)	22	3674.9 (2455.6–5499.6)	115	3584.5 (3093.6–4153.3)
	MGI	91	3.03 (2.45–3.74)	28	5.76 (3.95–8.41)	82	3.95 (3.32–4.71)	22	3.93 (2.71–5.70)	115	3.59 (3.13–4.10)
	SRR	40	44.0 (33.6–54.8)	18	64.3 (44.1–81.4)	39	47.6 (36.4–58.9)	9	40.9 (20.7–63.6)	52	45.2 (35.9–54.8)
Visit 6	GMT	89	2048.0 (1620.2–2588.7)	27	2822.3 (1968.2–4047.2)	83	2898.1 (2308.4–3638.4)	24	2846.6 (1847.9–4385.1)	114	2665.1 (2310.9–3073.5)
	MGI	89	2.38 (1.99–2.85)	26	3.40 (2.35–4.91)	82	3.27 (2.76–3.87)	24	2.86 (2.04–4.02)	114	2.69 (2.35–3.08)
	SRR	24	27.0 (18.1–37.4)	11	42.3 (23.4–63.1)	33	40.2 (29.6–51.7)	6	25.0 (9.8–46.7)	34	29.8 (21.6–39.1)

RSV-A and RSV-B neutralizing titers in the three study groups, with participants in the transplant groups stratified by mycophenolate compound (MC) use, before (first) vaccination (V1) and up to 12 months after (last) vaccination (V2, 3, 4, 5, and 6). In the 2-dose transplant group, the second vaccine dose was administered at V3. V3 titers were measured before dose 2. 1-dose transplant group, immunocompromised kidney or lung transplantation recipients aged ≥18 years who received one dose of AS01_E_-adjuvanted RSV prefusion F protein vaccine (adjuvanted RSVPreF3); 2-dose transplant group, immunocompromised kidney or lung transplantation recipients aged ≥18 years who received two doses of adjuvanted RSVPreF3 one to two months apart; non-immunocompromised group, non-immunocompromised individuals aged ≥50 years who received one dose of adjuvanted RSVPreF3. Source data are provided as a Source Data file.

*N* number of participants with available results at the given timepoint (GMT), or with available results at both the given timepoint and at baseline (MGI), or with a ≥4-fold increase in neutralizing titers compared to baseline (SRR), *CI* confidence interval, *GMT* geometric mean titer, *MGI* mean geometric increase (post-dose titer compared to baseline titer), *RSV*, respiratory syncytial virus, *SRR* seroresponse rate.

Age-stratified analyses were limited by small subgroup sizes, precluding firm conclusions.

### Cell-mediated immunogenicity kinetics

**Fig. 6:**
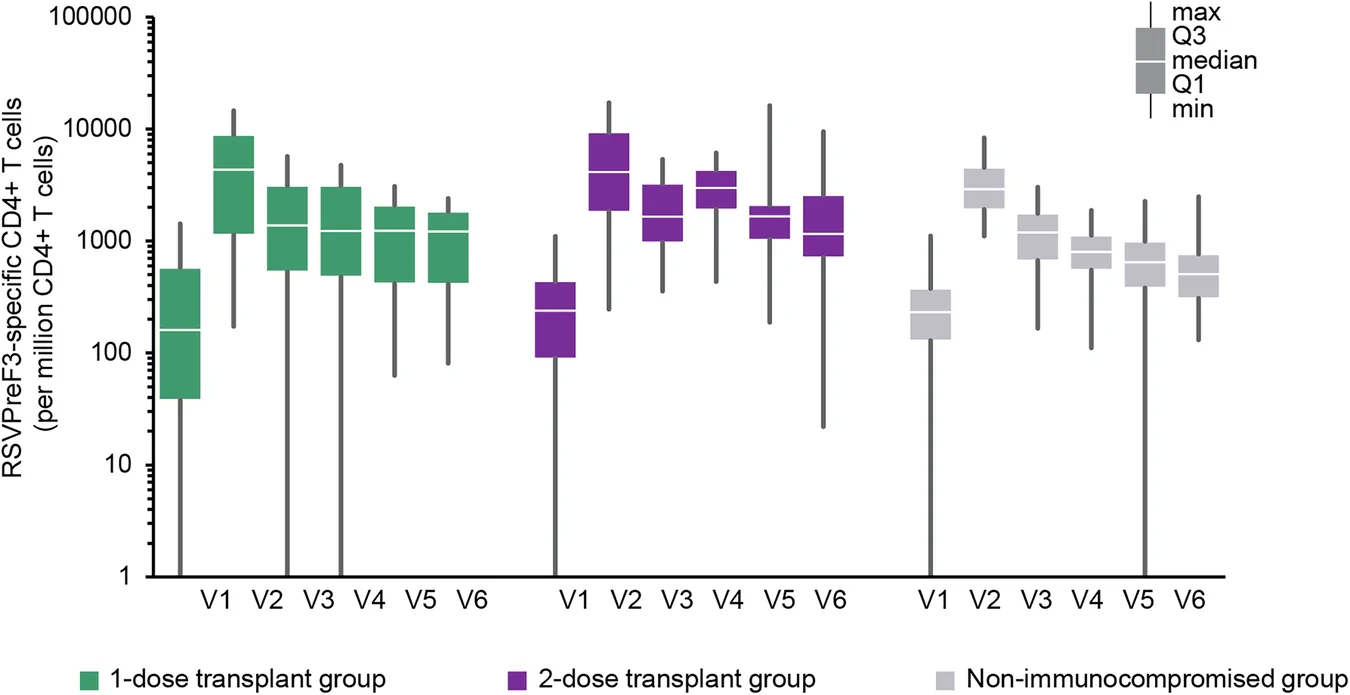
Median frequency of RSVPreF3-specific CD4+ T cells expressing ≥2 activation markers including ≥1 cytokine among CD40L, 4-1BB, interleukin-2, tumor necrosis factor-α, interferon-ɣ, interleukin-13, and interleukin-17, per million CD4+ T cells (per-protocol set for cell-mediated immunogenicity). RSVPreF3-specific CD4+ T-cell responses in the three study groups before (first) vaccination (V1) and up to 12 months after (last) vaccination (V2, 3, 4, 5, and 6). In the 2-dose transplant group, the second vaccine dose was administered at V3. V3 titers were measured before dose 2. The 1-dose transplant group is shown in green, the 2-dose transplant group in purple, and the non-immunocompromised control group in grey. Sample sizes at each visit (V1/V2/V3/V4/V5/V6) were as follows: 1-dose transplant group (23/24/22/23/22/17); 2-dose transplant group (28/27/27/26/25/25); and non-immunocompromised controls (31/28/24/28/28/23). RSVPreF3, respiratory syncytial virus prefusion F protein 3; 1-dose transplant group, immunocompromised kidney or lung transplantation recipients aged ≥18 years who received one dose of AS01_E_-adjuvanted RSVPreF3 vaccine (adjuvanted RSVPreF3); 2-dose transplant group, immunocompromised kidney or lung transplantation recipients aged ≥18 years who received two doses of adjuvanted RSVPreF3 approximately one month apart; non-immunocompromised group, non-immunocompromised individuals aged ≥50 years who received one dose of adjuvanted RSVPreF3; Q1, 25^th^ percentile; Q3, 75^th^ percentile. Source data are provided as a Source Data file.

In the cell-mediated immunogenicity subset, baseline median frequencies of RSVPreF3-specific CD4 + T cells expressing ≥2 activation markers were within the same range across groups, spanning 161.0 to 239.0 per million CD4 + T cells (Fig. 6).

Following vaccination, frequencies were higher than at baseline in all groups. Responses remained well above baseline up to 12 months post-last dose, with Visit 6 values ranging from 507.0 to 1220.0 per million CD4 + T cells across groups.

Stratification by transplant type showed RSVPreF3-specific CD4 + T-cell frequencies that were within a similar range among lung and kidney transplant recipients (Supplementary Table 4). RSVPreF3-specific CD4 + T-cell responses appeared preserved in transplant participants receiving mycophenolate compounds (Supplementary Table 5).

No RSVPreF3-specific CD8 + T-cell response was detected.

### Reactogenicity and safety

Within seven days post-dose 1, at least one solicited event was reported by 80.2% of participants in the 1-dose transplant group, 81.3% in the 2-dose transplant group, and 84.8% in the non-immunocompromised group; grade 3 solicited events were reported by 0.8%, 3.9%, and 1.6% of participants, respectively (Table 4). Post-dose 1, at least one solicited administration-site event was reported by 73.3% of participants in the 1-dose transplant group, 71.9% in the 2-dose transplant group, and 76.8% in the non-immunocompromised group, most commonly pain (72.5%, 71.9%, and 76.0%, respectively) (Table 4). Two participants in the 2-dose transplant group reported grade 3 solicited administration-site events (one pain and one swelling). Post-dose 1, at least one solicited systemic event was reported by 55.0% (1-dose transplant group), 51.6% (2-dose transplant group), and 59.2% (non-immunocompromised group) of participants (Table 4). The most frequent solicited systemic event was fatigue in the transplant groups (31.3% in 1-dose and 35.2% in 2-dose group), and myalgia in the non-immunocompromised group (39.2%) (Table 4). Grade 3 solicited systemic events were reported by one participant in the 1-dose transplant group, three in the 2-dose transplant group, and two in the non-immunocompromised group, predominantly fatigue, arthralgia, and headache.

**Table 4:** Solicited events, unsolicited adverse events, serious adverse events, and adverse events of special interest (exposed population)

	1-dose transplant	2-dose transplant		Non-immunocompromised
Event	*n* (%)	*n* (%)	*n* (%)	*n* (%)
	Post-dose 1	Post-dose 1	Post-dose 2	Post-dose 1
**Solicited events within 7 days following vaccination**	***N*** = **131**	***N*** = **128**	***N*** = **121**	***N*** = **125**
Any grade solicited event	105 (80.2)	104 (81.3)	94 (77.7)	106 (84.8)
Grade 3 solicited event	1 (0.8)	5 (3.9)	3 (2.5)	2 (1.6)
Administration-site event	96 (73.3)	92 (71.9)	83 (68.6)	96 (76.8)
Erythema	8 (6.1)	14 (10.9)	10 (8.3)	11 (8.8)
Pain	95 (72.5)	92 (71.9)	82 (67.8)	95 (76.0)
Swelling	8 (6.1)	11 (8.6)	6 (5.0)	3 (2.4)
Systemic event	72 (55.0)	66 (51.6)	66 (54.5)	74 (59.2)
Arthralgia	19 (14.5)	15 (11.7)	19 (15.7)	15 (12.0)
Fatigue	41 (31.3)	45 (35.2)	47 (38.8)	48 (38.4)
Fever^a^	2 (1.5)	1 (0.8)	2 (1.7)	1 (0.8)
Headache	31 (23.7)	33 (25.8)	30 (24.8)	44 (35.2)
Myalgia	37 (28.2)	38 (29.7)	38 (31.4)	49 (39.2)
**Unsolicited AEs within 30 days following vaccination**	***N*** = **131**	***N*** = **130**	***N*** = **121**	***N*** = **125**
Any grade unsolicited AE	23 (17.6)	33 (25.4)	39 (32.2)	27 (21.6)
Any grade unsolicited AE considered related to vaccine^b^	4 (3.1)	12 (9.2)	9 (7.4)	6 (4.8)
Grade 3 unsolicited AE	2 (1.5)	3 (2.3)	8 (6.6)	0 (0.0)
Grade 3 unsolicited AE considered related to vaccine^b^	0 (0.0)	0 (0.0)	0 (0.0)	0 (0.0)
**SAEs up to study end (Visit 6)**	***N*** = **131**	***N*** = **130**		***N*** = **125**
Any SAE	30 (22.9)	39 (30.0)		4 (3.2)
SAE considered related to vaccine^b^	1 (0.8)^c^	0 (0.0)		0 (0.0)
Fatal SAE	3 (2.3)	3 (2.3)		0 (0.0)
**AESIs up to study end (Visit 6)**	***N*** = **131**	***N*** = **130**		***N*** = **125**
Any pIMD	2 (1.5)	1 (0.8)		1 (0.8)
pIMD considered related to vaccine^b^	0 (0.0)	0 (0.0)		0 (0.0)
Any AF	0 (0.0)	1 (0.8)		1 (0.8)
AF considered related to vaccine^b^	0 (0.0)	0 (0.0)		0 (0.0)
Any AESI related to organ transplant	0 (0.0)	1 (0.8)		NA
Any AESI related to organ transplant considered related to vaccine	0 (0.0)	0 (0.0)		NA

1-dose transplant group, immunocompromised kidney or lung transplantation recipients aged ≥18 years who received one dose of AS01_E_-adjuvanted respiratory syncytial virus prefusion F protein vaccine (adjuvanted RSVPreF3); 2-dose transplant group, immunocompromised kidney or lung transplantation recipients aged ≥18 years who received two doses of adjuvanted RSVPreF3 one to two months apart; non-immunocompromised group, non-immunocompromised individuals aged ≥50 years who received one dose of adjuvanted RSVPreF3; N, for solicited events: number of participants in the exposed population with available diary card data; n (%), number (percentage) of participants presenting ≥1 type of AE in a given category.

*N*, for unsolicited AEs, SAEs, and AESIs, number of participants in the exposed population; AE, adverse event; SAE, serious AE; AESI, AE of special interest; pIMD, potential immune-mediated disease; AF, atrial fibrillation. ^a^Fever was defined by a temperature ≥38.0 °C. ^b^By investigator assessment. ^c^Case of gastroenteritis. In this participant, there is indirect evidence suggesting a bacterial infectious etiology, as there was an increased white blood cell count at time of admission, which normalized after two days of antibiotics treatment with azithromycin. Although stool culture was negative for bacteria, the yield of conventional stool cultures is known to be low in both immunocompetent and transplant populations. Additionally, the participant received mycophenolate mofetil, tacrolimus, and sirolimus, all of which could be associated with diarrhea and represent plausible alternative contributors to the clinical presentation.

Within seven days post-dose 2 in the 2-dose transplant group, 77.7% of participants reported at least one solicited event, and 2.5% reported at least one grade 3 solicited event (Table 4). At least one solicited administration-site event was reported by 68.6% of participants, mostly pain (67.8%) (Table 4); two participants reported grade 3 solicited administration-site pain. At least one solicited systemic event was reported by 54.5% of participants, most commonly fatigue (38.8%) (Table 4); three participants reported grade 3 solicited systemic events post-dose 2 (myalgia, arthralgia, fatigue).

After any dose, the median duration for each solicited event type was ≤3 days for any grade, except for a single episode of fever post-dose 1 in the 2-dose transplant group lasting 6 days.

Rates and intensity of unsolicited adverse events (AEs) reported within 30 days after vaccination were broadly comparable across groups and doses: 17.6% of 1-dose transplant recipients, 25.4% of 2-dose transplant recipients post-dose 1, 32.2% of 2-dose transplant recipients post-dose 2, and 21.6% of non-immunocompromised participants reported at least one unsolicited AE (Table 4). Among transplant recipients, Infections and infestations was the most frequent SOC (8.4% of 1-dose transplant recipients and 16.2% of 2-dose transplant recipients after any dose). Within this SOC, the most frequent unsolicited AE by preferred term (PT) was Nasopharyngitis in the transplant recipients (no 1-dose transplant recipients, 3.1% of 2-dose transplant recipients after any dose). Among non-immunocompromised participants, the most frequent SOC was Respiratory, thoracic and mediastinal disorders (8.0%), with Oropharyngeal pain (3.2%) as the leading PT.

Post-dose 1, at least one unsolicited AE considered vaccine-related by the investigator was reported by 3.1% of 1-dose transplant recipients, 9.2% of 2-dose transplant recipients, and 4.8% of non-immunocompromised participants. Post-dose 2, 7.4% of 2-dose transplant recipients reported at least one unsolicited AE that was considered vaccine-related (Table 4). The most frequently reported unsolicited AE considered related by PT was Administration-site pruritus across groups (none in 1-dose transplant group, 3.8% in 2-dose transplant group after any dose, and 0.8% in non-immunocompromised group). Grade 3 unsolicited AEs were reported by 1.5% of 1-dose transplant recipients, 2.3% (post-dose 1) and 6.6% (post-dose 2) of 2-dose transplant recipients, and no participant in the non-immunocompromised group; none of the reported grade 3 unsolicited AEs were considered vaccine-related.

Up to study end, serious AEs (SAEs) were more frequent in the transplant groups (22.9% in 1-dose, 30.0% in 2-dose group) than in the non-immunocompromised group (3.2%) (Table 4). Pneumonia was the most common SAE by PT for the transplant groups (2.3% in 1-dose and 3.8% in 2-dose group). One SAE, a case of gastroenteritis in the 1-dose transplant group, was considered vaccine-related by the investigator. It occurred two days post-vaccination in a lung transplant recipient on mycophenolate, tacrolimus, and sirolimus (Supplementary Table 6). Inflammatory markers (white blood cell count and C-reactive protein) were elevated at hospital admission, stool culture was negative for bacteria, and no viral and parasite stool testing was performed. After two days of antibiotic therapy with azithromycin, white blood cell counts normalized, and the participant was discharged. A grade 3 SAE of anaphylactic shock was reported in the 2-dose transplant group but was not considered vaccine-related by the investigator. It occurred three days post-dose 2, resolved after three days and was attributed to administration of prophylactic clavulanic acid in a participant with a known clavulanic acid allergy. Six fatalities were reported (all in transplant groups), which were not considered vaccine-related; three (out of the six) occurred after the respective participants’ study completion (Supplementary Table 6).

Up to study end, at least one AE of special interest (AESI) was reported in two 1-dose transplant recipients (1.5%; immune thrombocytopenia and scleroderma, both classified as potential immune-mediated diseases [pIMDs]), three 2-dose transplant recipients (2.3%; optic ischemic neuropathy classified as a pIMD, kidney impairment classified as a transplant-related AESI, and atrial fibrillation [AF]), and two non-immunocompromised participants (1.6%; one exacerbation of psoriasis classified as pIMD and one AF) (Table 4 and Supplementary Table 6). None of these AESIs were considered vaccine-related.

No pregnancies were reported during the study.

In total, six participants were withdrawn from the study due to unsolicited AEs or SAEs: one participant in the 1-dose transplant group (fatal SAE), four in the 2-dose transplant group (four SAEs, including two fatal), and one in the non-immunocompromised group (SAE). None of these SAEs were considered vaccine-related by the investigator.

The independent data monitoring committee did not raise concerns.

## Discussion

Individuals with immunocompromising conditions are at increased risk of severe RSV outcomes and represent an important priority population for vaccination. Data on the immunogenicity of RSV vaccines^44–48^ and their real-world effectiveness against severe outcomes^32–37,43^ in immunocompromised individuals are accumulating, although most evidence to date comes from broad and heterogeneous cohorts that include diverse immunocompromised subgroups.

Here, we present the immunogenicity, reactogenicity, and safety of adjuvanted RSVPreF3 in lung and kidney transplant recipients on maintenance immunosuppressive regimens, among whom 76.6% received mycophenolate compounds.

Our descriptive analysis of immunogenicity showed that a single dose of adjuvanted RSVPreF3 increased humoral and cell-mediated immune responses in lung and kidney transplant recipients, with responses that persisted for up to 12 months after vaccination. Strong neutralizing antibody responses were generated against both RSV-A and RSV-B subtypes, with similar responses observed for the two subtypes across timepoints, demonstrating a consistent and broadly reactive immune profile across RSV subtypes. However, compared with non-immunocompromised controls, transplant recipients generally exhibited a slower induction and lower RSV-A and RSV-B neutralizing titers after a single dose. Following a second dose administered one to two months later, titers in transplant recipients further increased and were within a similar range to those observed in non-immunocompromised individuals. This response pattern was predominantly observed among transplant recipients receiving mycophenolate, for whom initial titers after a single dose were generally lower than those observed in the non-immunocompromised population but, after a second dose, reached a similar range. Among recipients not on mycophenolate, RSV-A/RSV-B titers after a single dose were within a similar range to those of the non-immunocompromised group at all timepoints, and a second dose did not result in further increases. These findings align with prior studies with other vaccines (influenza, SARS-CoV-2, pneumococcal, and others) in immunocompromised populations, which identified mycophenolate as a strong predictor of reduced humoral immunogenicity^13–17^.

Cell-mediated immunogenicity was assessed in a small subset of participants in each group; overall, the RSVPreF3-specific CD4 + T-cell responses that were induced by one or two doses of adjuvanted RSVPreF3 remained above baseline up to study end in all groups.

The robust and sustained humoral and cellular immune responses observed with adjuvanted RSVPreF3 in this immunocompromised population are noteworthy. The AS01_E_ adjuvant, which has been associated with strong and durable CD4 + T-cell responses alongside high neutralizing titers^49^, may contribute to this observed immunogenicity, even in the context of immunosuppression.

Both dosing regimens of adjuvanted RSVPreF3 were well tolerated and had an acceptable safety profile. Most solicited events were mild to moderate and short-lived, and unsolicited AEs were overall comparable across groups. SAEs were reported more frequently in transplant recipients than in non-immunocompromised individuals; Infections and infestations was the leading SAE by SOC in the transplant groups, and Pneumonia the most common SAE by PT, aligning with the known susceptibility of this population to severe respiratory infections^1^. One SAE was assessed as vaccine-related by the investigator (gastroenteritis in the 1-dose transplant group). Due to the patient’s inflammatory status, potential confounders, and insufficient evidence of causality, the Sponsor assessed the event to be unrelated to the study intervention.

Data on RSV vaccines in immunocompromised populations are so far limited and largely reflect single-dose assessments. Available immunogenicity data vary considerably across studies^44–48^, likely reflecting differences in study size, design, underlying conditions, and immunosuppression regimens. In a small study in immunocompromised adults (predominantly solid organ transplant recipients), a single dose of either of two RSV vaccines (including adjuvanted RSVPreF3) elicited neutralizing antibody responses, though similar to our observations, reduced responses were noted compared to historical data from non-immunocompromised populations^44^. A single-center interventional cohort study in allogeneic hematopoietic stem cell transplant and lung transplant recipients also noted modest increases in humoral immune responses in both conditions approximately one month after a single dose of adjuvanted RSVPreF3^45^. Another single-center retrospective cohort study in lung transplant recipients reported that a single dose of adjuvanted RSVPreF3 induced sustained antibody responses above baseline up to six months post-vaccination^46^.

Clinical studies evaluating two-dose RSV vaccination strategies in immunocompromised populations have shown variable results. In a phase 3 study of the mRNA-1345 RSV vaccine (mRESVIA, Moderna) in liver, kidney, and lung transplant recipients, one dose was immunogenic, and administration of a second dose resulted in modestly higher neutralizing titers one month post-vaccination^47^. The added benefit of the second dose was more apparent among participants with a kidney or lung transplant, those <2-year post-transplant, and those receiving mycophenolate, consistent with our findings. In a trial evaluating another RSV vaccine (Abrysvo, Pfizer) in a broad immunocompromised cohort that included solid organ transplant recipients, individuals with autoimmune inflammatory disorders receiving immunomodulator therapy, with advanced non-small-cell lung cancer, or on hemodialysis for end-stage renal disease, variable but immunogenic neutralizing antibody responses were reported one month after a single vaccine dose. In this heterogeneous population, a second dose did not further increase titers at one month post-vaccination^48^.

Emerging real-world effectiveness data suggest that a single RSV vaccine dose can reduce RSV-related illness and hospitalization in immunocompromised populations^32–37,43^, although estimates are often drawn from broader, heterogeneous immunocompromised cohorts and vary by outcome, care setting, and population characteristics. Across studies, findings suggest that effectiveness in immunocompromised populations is generally comparable to that in non-immunocompromised groups, with some estimates indicating modestly reduced protection^32,34^. A recent large US retrospective cohort study using administrative claims data (CLEAR-VE RSV) reported adjuvanted RSVPreF3 effectiveness against RSV-related hospitalization of 71.4% (95% CI: 57.0, 81.1) among adults aged ≥60 years with immunocompromising conditions, comparable to the estimate observed in the overall study population (75.6% [69.8, 80.2])^37^. Similarly, a smaller single-center US test-negative study found high effectiveness of adjuvanted RSVPreF3 against hospitalization for RSV-associated lower respiratory tract infections in immunocompromised populations (which comprised 16.2% of the overall assessed population), with estimates that were very similar to the overall and non-immunocompromised populations (overall population: 82.3% [95% CI: 61.5, 91.9]; non-immunocompromised population: 81.1% [55.8, 91.9]; immunocompromised population: 90.0% [11.6, 98.9])^35^. Another recent target-trial emulation using Veterans Health Administration data assessed the effectiveness of a single RSV vaccine dose against RSV infections over two RSV seasons, and found that a single RSV vaccine dose provided initial strong protection in adults ≥60 years, but noted that effectiveness declined over 18 months, particularly among immunocompromised populations^36^. Evidence from more specific immunocompromised populations is also emerging; a large US target-trial emulation using the nationwide Epic Cosmos database found that RSV vaccination among solid organ transplant recipients (with follow-up during October 2023–March 2025) was associated with lower risks of RSV infection, hospitalization, and all-cause mortality, with generally consistent findings across transplant types^43^.

Taken together, the findings of the current study align with previous RSV vaccine research in immunocompromised populations, indicating strong immunogenicity, though the magnitude of the responses varies across subgroups, reflecting the heterogeneity of this population^32–36,44–48^. A key strength of the present study is its focus on moderately to severely immunocompromised solid organ transplant recipients, providing robust immunogenicity and safety data that can help to support interpretation across other immunocompromised populations. Moreover, the study is among the first to evaluate a two-dose RSV vaccine regimen in an immunocompromised population. The study design enabled important subgroup analyses—particularly distinguishing between patients on immunosuppressive regimens with versus without mycophenolate. Immunocompromised patients generally experience more rapid waning of vaccine‑induced immunity than immunocompetent individuals^50–52^; our long-term follow‑up until 12 months post-vaccination therefore provides important data on durability of both safety and immune responses in this high‑risk population. The contemporaneous non-immunocompromised group included as a comparator helped ensure similar RSV seasonal exposures between groups across regions and time, thereby enhancing the homogeneity of the comparison. Overall, the findings from this trial contribute valuable data to the evidence base for RSV vaccination in immunocompromised populations.

There are several limitations to this work. Although the study offers important insights into vaccine immunogenicity in a challenging patient population, the descriptive study design, the sample size, especially within specific subgroups, and incomplete control of confounding factors, may affect the strength of the conclusions. In addition, the cell-mediated immunogenicity subset was selected non-randomly, limiting comparability and interpretation of these analyses. More broadly, the study focuses on immunogenicity, recognizing that RSV-specific neutralizing titers and T-cell responses are considered key components of protective immunity, while no validated correlate of protection exists for RSV vaccines. Future work could explore whether specific combinations of immunosuppressant classes differentially affect vaccine responses, and whether varying dosing intervals could influence immunogenicity. Moreover, effectiveness assessments in specific immunocompromised subpopulations will support risk stratification and guide vaccination policies.

In conclusion, a single dose of adjuvanted RSVPreF3 was immunogenic in kidney or lung transplant recipients receiving maintenance immunosuppressant therapy. Among transplant recipients who received two doses of adjuvanted RSVPreF3, neutralizing titers were numerically higher approximately one month after the second dose than after the first dose, and were within a similar range to those observed in non-immunocompromised individuals. Subgroup analyses showed that responses after one dose were specifically hampered in individuals receiving mycophenolate; however, after a second dose, titers in this subgroup were within a similar range to those observed in non-immunocompromised controls. Both dosing schedules elicited RSVPreF3-specific cell-mediated immune responses in transplant recipients that were broadly in line with those observed in the controls. Humoral and cell-mediated immune responses declined but persisted up to 12 months following completion of the single- or two-dose schedules. The safety profile was acceptable for both doses. Overall, the findings support a favorable benefit-risk profile of adjuvanted RSVPreF3 in immunocompromised populations.

## Methods

### Study design, participants, and intervention

This phase 2b, randomized, open-label study (NCT05921903) was conducted across 48 centers in Australia, Canada, Germany, Italy, Japan, Republic of Korea, Spain, and the US from July 28, 2023, to May 16, 2025. The protocol and protocol amendments were approved by the relevant independent ethics committees/institutional review boards (Supplementary Notes). The study adhered to the protocol, statistical analysis plan, and consensus ethical principles derived from international guidelines, including the Declaration of Helsinki, CIOMS International Ethical Guidelines, ICH guidelines for good clinical practice, and applicable laws and regulations. Participants provided written informed consent before any study-specific procedures. The study protocol is available in the Supplementary Information and the statistical analysis plan can be consulted on https://www.gsk-studyregister.com/en/trial-details/?id=219900.

The study enrolled two populations: immunocompromised individuals and non-immunocompromised individuals, serving as a control (Fig. 2).

The immunocompromised population comprised individuals aged ≥18 years who had received an ABO-compatible allogeneic kidney or lung transplant more than 12 months prior to first study vaccination, were on maintenance immunosuppressant therapy, and had stable kidney or lung function, as assessed by the investigator. Transplant-type distribution within the population was targeted at minimum 65% kidney and 20% lung transplant recipients. Exclusions included allograft rejections in the previous three months prior to the first study intervention administration, use of anti-CD20 or other B-cell-depleting monoclonal antibodies as immunosuppressant therapy within nine months prior to first vaccination, and malignancies or other events that could increase chronic allograft dysfunction risk.

The non-immunocompromised population consisted of adults aged ≥50 years, including those with chronic stable medical conditions with or without specific treatment (e.g., diabetes, hypertension, or cardiac disease). Individuals with suspected immunosuppressive or immunodeficient conditions were excluded. Enrollment in the non-immunocompromised population was organized into three age categories, with minimum 30% of participants aged 50–59 years, 30% aged 60–69 years, and 20% aged 70 years or older, while the remaining 20% could be freely allocated across these age categories.

For both populations, individuals who had previously received any RSV vaccine were ineligible. The study included both males and females. Sex was collected and recorded for each participant, as determined and documented by the investigator in the electronic case report form. Participants received compensation in accordance with GSK’s policy: https://www.gsk.com/media/r1xojpsj/gsk-position-on-approach-to-clinical-trials.pdf. Detailed eligibility criteria are provided in the Supplementary Notes.

Transplant recipients were randomized 1:1 to receive either one dose (1-dose transplant group) or two doses administered one to two months apart (range: 30–60 days) (2-dose transplant group) of adjuvanted RSVPreF3 (Fig. 2). The non-immunocompromised group received a single dose. Each 0.5-ml dose of adjuvanted RSVPreF3 contained 120 µg of RSVPreF3 antigen and the liposome-based AS01_E_ adjuvant system, containing 25 µg of 3-O-desacyl-4′-monophosphoryl lipid A and 25 µg of *Quillaja saponaria* Molina, fraction 21 (QS-21) (QS-21 is licensed by GSK from Antigenics LLC, a wholly owned subsidiary of Agenus Inc., a Delaware, USA corporation). The vaccine was administered intramuscularly in the deltoid region of the non-dominant arm.

Approximately 30% of participants in each group (non-immunocompromised group, 1-dose transplant group, and 2-dose transplant group) were allocated to the cell-mediated immunogenicity subset at Visit 1. These participants were recruited from selected countries and sites. At these sites, investigators allocated the first participants enrolled in each group, if possible, to the cell-mediated immunogenicity subset at Visit 1, until the target of approximately 38 participants per group was reached.

Most participants in all groups had five visits: Day 1 (Visit 1; day of first vaccination in all groups), 30–60 days after Visit 1 (Visit 3; day of second vaccination in the 2-dose transplant group), 30–42 days after Visit 3 (Visit 4), 180–210 days after the last dose (Visit 5), and 350–380 days after the last dose (Visit 6). The participants allocated to the cell-mediated immunogenicity subset had an additional visit 7–14 days after Day 1 (Visit 2). The total study duration was approximately 12 months for participants in the non-immunocompromised group and 1-dose transplant group, and 13–15 months for those in the 2-dose transplant group.

### Randomization

The randomization strategy in the transplant recipients in two dosing groups in a 1:1 ratio employed a permuted block randomization method, with stratification by transplant type and cell-mediated immunogenicity subset, using a GSK Randomization and Trial Supply Management system. This system managed recruitment caps for the transplant types to maintain balance.

### Study objectives and endpoints

The primary study objective was to assess the humoral immune response in terms of RSV-A and RSV-B neutralizing titers following the first and second dose of adjuvanted RSVPreF3 within the group of kidney and lung transplant participants who received two doses, specifically assessed by the MGI of RSV-A/RSV-B neutralizing titers one-month post-dose 2 over one-month post-dose 1 in the 2-dose transplant group.

Secondary endpoints included evaluating the humoral immune response in terms of RSV-A and RSV-B neutralizing titers, the cell-mediated immune response measured by the frequency of RSVPreF3-specific positive CD4 + /CD8 + T cells per million CD4 + /CD8 + T cells, and assessing reactogenicity and safety up to approximately 12 months after the last dose.

A detailed list of study objectives is presented in Supplementary Table 7.

### Immunogenicity evaluations

Blood samples were collected from all participants at baseline (Visit 1, pre-vaccination), 30–60 days post-dose 1 (Visit 3), 30–42 days after Visit 3 (Visit 4), and approximately 6 months (Visit 5) and 12 months (Visit 6) post-last dose (Fig. 2). In participants assigned to the cell-mediated immunogenicity subset, additional blood was collected for cell-mediated immunogenicity analysis at the same timepoints. Moreover, blood samples in this subset were collected at an additional visit 7–14 days post-dose 1 (Visit 2) for humoral and cell-mediated immunogenicity analysis.

All immunogenicity blood samples of the study were analyzed at a single, centralized laboratory. RSV-A and RSV-B neutralizing titers were assessed from the same samples using a serum neutralization assay and expressed in ED60, with lower limits of quantification set at 18 ED60 for RSV-A and 30 ED60 for RSV-B.

Cell-mediated immunogenicity, assessed as RSVPreF3-specific T-cell responses, was evaluated by intracellular cytokine staining of peripheral blood mononuclear cells followed by flow cytometry analysis, using an adaptation of methods previously described^53,54^. Briefly, peripheral blood mononuclear cells were stimulated in vitro either with a pool of 15-mer peptides overlapping by 11 amino acids and spanning the RSVPreF3 protein sequence, or with medium alone as negative control (background condition). Culture medium contained anti-CD28/anti-CD49d costimulatory antibodies (Pharmingen, cat. no. 555725 and 555501, respectively). In peptide-stimulated wells, the final dimethyl sulfoxide dilution was 1/1600. After an initial 2-hour incubation at 37 °C, Brefeldin A (GolgiPlug, Pharmingen) was added to inhibit cytokine secretion for additional 18 hours (total incubation time: 20 hours). Cells were subsequently harvested, stained for surface markers (CD4 and CD8) together with a viability dye (*L&D Aqua*, Invitrogen), and then fixed and permeabilized (CellFix/CellPerm, Pharmingen). Permeabilized cells were stained with labelled antibodies specific for CD3, CD40 ligand, 4-1BB, interleukin-2, tumor necrosis factor-α, interferon-ɣ, interleukin-13, and interleukin-17. Supplementary Fig. 2 illustrates the gating strategy of flow cytometry. A detailed list of antibodies and their validation is provided in the Supplementary Notes. Data acquisition was performed on flow cytometers (Fortessa X20, Symphony A3 or A5, BD Biosciences).

Data were analyzed using FlowJo v09 (BD Biosciences). Boolean gating analysis was used to identify and sum all T-cell populations expressing at least two activation markers (including at least one cytokine), to calculate polypositive counts. These counts were subsequently expressed as frequencies of polypositive cells per million CD4+ or CD8 + T cells. Polypositive frequencies obtained under background conditions were then subtracted from those obtained under stimulated conditions to calculate RSVPreF3-specific polypositive CD4+ and/or CD8 + T-cell frequencies, expressed per million CD4 + /CD8 + T cells.

### Reactogenicity and safety evaluations

All participants recorded solicited administration-site and systemic events that started within seven days after each dose using paper diary cards. Unsolicited AEs were recorded by the investigator up to 30 days after each dose. SAEs, AEs leading to discontinuation, and AESIs, including pIMDs, AF, and transplant rejections (specific to kidney and lung transplant patients), were recorded up to study end.

The intensity of each AE was graded from mild (grade 1) to severe (grade 3); solicited events were graded by the participants, and other AEs were graded by the investigators (Supplementary Notes). The investigator evaluated the relationship between the vaccine and each AE and reported its outcome.

All reported AEs were proactively followed up by the investigator at subsequent visits or contacts. All SAEs and AESIs were followed until resolution, stabilization, otherwise explained, or participant lost to follow-up.

An independent data monitoring committee including clinicians with expertise in lung and kidney transplant monitored safety on a regular basis throughout the study and made recommendations.

### Statistics

The target enrollment was 375 participants: 250 adult transplant recipients and 125 non-immunocompromised adults. The sample size was determined to adequately detect relevant increases in RSV-A and RSV-B GMTs post-dose 2 compared to post-dose 1 (Supplementary Notes). All objectives were evaluated descriptively without hypothesis testing.

Humoral and cell-mediated immunogenicity was assessed on the respective per-protocol set.

The primary objective was addressed using an estimation approach without hypothesis testing. The MGIs of serum neutralizing titers for RSV-A and RSV-B (i.e., the geometric means of the within-participant ratios of neutralizing titers one-month post-dose 2 [Visit 4] over one-month post-dose 1 [Visit 3]) were computed in the 2-dose transplant group, with 95% CIs derived from back transformation of the CI for the mean difference between log_10_-transformed values, assuming normal distribution with unknown variance. Missing data were not imputed.

RSV-A and RSV-B neutralizing GMTs, MGIs over baseline (i.e., geometric means of the within-participant ratios of each post-vaccination titer over the pre-vaccination titer), and SRRs were calculated with 95% CIs at each timepoint.

For between-group comparisons of humoral responses, aimed to characterize the difference in immunogenicity between groups, group GMT ratios with 95% CIs were calculated at each Visit, derived from an ANCOVA model on log_10_-transformed titers for each neutralization assay (non-immunocompromised over 1-dose transplant, non-immunocompromised over 2-dose transplant, 2-dose transplant over 1-dose transplant, except for Visits 2 and 3, where 1-dose transplant and 2-dose transplant groups were combined, i.e., non-immunocompromised over combined transplant). The model included the group and baseline log_10_-transformed titer as covariates. The group GMT ratios were based on a back transformation of group contrast in the ANCOVA model applied to the logarithmically transformed titers. In addition, 2-sided 95% CI for the difference in SRRs between groups were calculated (non-immunocompromised minus 1-dose transplant, non-immunocompromised minus 2-dose transplant, 2-dose transplant minus 1-dose transplant, except for Visits 2 and 3, where 1-dose transplant and 2-dose transplant were combined, i.e., non-immunocompromised minus combined transplant).

Subgroup analyses on humoral immunogenicity were performed on the per-protocol set, stratified by transplant subtype, age group, and immunosuppressive therapy. The latter was categorized as any single agent or combination regimen (two or more agents) from among three immunosuppressant groups: corticosteroids, mycophenolate compounds, and calcineurin inhibitors and/or mTOR inhibitors (a small subset of participants on mTOR inhibitors were pooled with calcineurin inhibitors for analysis purposes). In addition, subgroup analyses were conducted specifically based on the presence/absence of mycophenolate compounds within the immunosuppressant regimen.

The cell-mediated immunogenicity response expressed as the median frequency of RSVPreF3-specific CD4 + /CD8 + T cells (per million CD4 + /CD8 + T cells) was summarized for each study group at each timepoint using descriptive statistics in the cell-mediated immunogenicity subset.

Demographic characteristics and safety endpoints were assessed on the exposed population. Number and percentages of participants with AEs were calculated with exact 95% CIs.

Sex-stratified analyses were not prespecified in the study protocol or statistical analysis plan. The proportion of male and female participants is reported as part of the demographic summary.

Statistical analyses were performed using SAS Viya 3.5 software (SAS Institute Inc., Cary, NC, US).

### Reporting summary

Further information on research design is available in the Nature Portfolio Reporting Summary linked to this article.

## Supplementary information

Supplementary Information

Reporting Summary

Transparent Peer Review file

## Source data

Source Data

## Data Availability

Not all participant-level data generated or analyzed during this study are publicly available because they contain information that could compromise participant privacy or consent. Where appropriate, data underlying the figures and tables are provided in the Source Data file; however, data rows have been randomized to minimize the risk of participant re-identification. Additional de-identified participant data generated and analyzed during this study can be requested via Vivli - Center for Global Clinical Research Data. Requests are reviewed by an independent panel within 90 calendar days of receipt of a complete proposal, and if approved, researchers will be granted access to anonymized data in a secure environment after signing a Data Sharing Agreement (DSA). Additional guidance is available via https://www.gsk-studyregister.com/gsk-patient-level-data-sharing-july2025.pdf. Source data are provided with this paper.
