# Supplementary material for: AS01E-adjuvanted RSV pre-F protein vaccine in adults with lung or kidney transplant: a phase 2b randomized trial
Source: Nat Commun. 2026 Nov 13;17:11678. doi: 10.1038/s41467-026-78584-9 (PMC13768339; doi:10.1038/s41467-026-78584-9)
Supplement: Supplementary file 1 [file 41467_2026_78584_MOESM1_ESM.pdf]

AS01$_E$-adjuvanted RSV pre-F protein vaccine in adults with lung or kidney transplant: a phase 2b randomized trial

Authors: Catherine Small, Deepali Kumar, Anum Abbas, Ana Sánchez Fructuoso, Shigeyoshi Yamanaga, Virginia Luz Pérez González, Hristos Karakizlis, Michael Nissen, Pyoeng Gyun Choe, Vera Paloschi, Steven Chadban, Hiwot Amare Hailemariam, Catherine Gerard, Marie-Pierre David, Dominique Descamps, Corinne Vandermeulen, Kemi Oluwayi, Veronica Hulstrøm, on behalf of the RSV OA=ADJ-023 Study Group

Supplementary information

Supplementary Table 1. Baseline characteristics of the study participants in the transplant groups, stratified by mycophenolate compound use (exposed population)........................ 4

Supplementary Table 2. Baseline comorbidities based on MedDRA System Organ Classes (exposed population). 5

Supplementary Table 3. Between-group assessments of RSV-A and RSV-B neutralizing titers in terms of adjusted GMT ratios and SRR differences (per-protocol set for humoral immunogenicity).. 8

Supplementary Table 4. Median frequency of RSVPreF3-specific CD4+ T cells expressing ≥2 activation markers including ≥1 cytokine among CD40 ligand, 4-1BB, interleukin-2, tumor necrosis factor-α, interferon-ɣ, interleukin-13, and interleukin-17, per million CD4+ T cells, with participants in the transplant groups stratified by transplant type (per-protocol set for cell-mediated immunogenicity). 10

Supplementary Table 5. Median frequency of RSVPreF3-specific CD4+ T cells expressing ≥2 activation markers including ≥1 cytokine among CD40 ligand, 4-1BB, interleukin-2, tumor necrosis factor-α, interferon-ɣ, interleukin-13, and interleukin-17, per million CD4+ T cells, with participants in the transplant groups stratified by mycophenolate compound use (per-protocol set for cell-mediated immunogenicity). 12

Supplementary Table 6. Serious adverse events considered vaccine-related, fatal serious adverse events, and adverse events of special interest reported from vaccination until study end (exposed population). 14

Supplementary Table 7. Study objectives. 19

Supplementary Figure 1. RSV-A and RSV-B neutralizing titers in terms of seroresponse rates (per-protocol set for humoral immunogenicity). 22

Supplementary Figure 2. Gating strategy of flow cytometry for assessment of cell-mediated immunogenicity. 24

Supplementary Notes 26
Eligibility criteria 26
Antibodies used for flow cytometry 33
Intensity grading of solicited events and adverse events 33
Sample size determination 35
Overview of ethics committees 36
CONSORT 2025 checklist 38
Study Protocol 41

Supplementary Table 1. Baseline characteristics of the study participants in the transplant groups, stratified by mycophenolate compound use (exposed population).

Characteristic	1-dose transplant		2-dose transplant	
	MC<br>N=100	No MC<br>N=31	MC<br>N=100	No MC<br>N=30
Mean (SD) age at vaccination[a], y	62.1 (9.2)	62.1 (9.9)	60.9 (8.4)	63.0 (8.4)
Mean (SD) BMI[b], kg/m^2	27.5 (5.9)	26.2 (3.4)	27.6 (6.9)	27.1 (4.2)
Transplant type, n (%)				
Kidney transplant	74 (74.0)	18 (58.1)	73 (73.0)	21 (70.0)
Lung transplant	26 (26.0)	13 (41.9)	27 (27.0)	9 (30.0)
Median (min, max) interval between last transplant and Visit 1 (months)	66 (13, 319)	68 (12, 386)	58 (14, 450)	70 (15, 325)

1-dose transplant group, immunocompromised kidney or lung transplantation recipients aged ≥18 years who received one dose of $AS01_E$-adjuvanted respiratory syncytial virus prefusion F protein vaccine (adjuvanted RSVPreF3); 2-dose transplant group, immunocompromised kidney or lung transplantation recipients aged ≥18 years who received two doses of adjuvanted RSVPreF3 one to two months apart; MC, mycophenolate compound use; N, number of participants in the exposed population; n (%), number (percentage) of participants in a given category; SD, standard deviation; y, years; BMI, body mass index. [a]Age was computed based on incomplete date of birth (only year was available). [b]BMI data from four participants in the 2-dose transplant group were not available (two in the MC subgroup and two in the No-MC subgroup).

Supplementary Table 2. Baseline comorbidities based on MedDRA System Organ Classes (exposed population).

Pre-existing condition, n (%)	1-dose transplant<br>N=131	2-dose transplant<br>N=130	Non-immunocompromised<br>N=125
Any	131 (100)	130 (100)	94 (75.2)
**SOC: Metabolism and nutrition disorders**	**109 (83.2)**	**110 (84.6)**	**43 (34.4)**
HLT: Diabetes mellitus	46 (35.1)	51 (39.2)	16 (12.8)
PT: Type 2 diabetes mellitus	35 (26.7)	38 (29.2)	16 (12.8)
HLT: Lipid metabolism and deposit disorders NEC	40 (30.5)	46 (35.4)	12 (9.6)
PT: Dyslipidaemia	40 (30.5)	45 (34.6)	12 (9.6)
HLT: Disorders of purine metabolism	30 (22.9)	31 (23.8)	3 (2.4)
PT: Hyperuricaemia	22 (16.8)	22 (16.9)	0 (0)
HLT: Hyperlipidaemias NEC	22 (16.8)	24 (18.5)	10 (8.0)
PT: Hyperlimidaemia	22 (16.8)	24 (18.5)	10 (8.0)
HLT: Elevated cholesterol	26 (19.8)	12 (9.2)	7 (5.6)
PT: Hypercholesterolaemia	26 (19.8)	12 (9.2)	7 (5.6)
HLT: Fat soluble vitamin deficiencies and disorders	19 (14.5)	6 (4.6)	3 (2.4)
PT: Vitamin D deficiency	19 (14.5)	6 (4.6)	3 (2.4)
**SOC: Vascular disorders**	**102 (77.9)**	**105 (80.8)**	**48 (38.4)**
HLT: Vascular hypertensive disorders NEC	98 (74.8)	102 (78.5)	46 (36.8)
PT: Hypertension	96 (73.3)	100 (76.9)	45 (36.0)
**SOC: Gastrointestinal disorders**	**70 (53.4)**	**62 (47.7)**	**23 (18.4)**
HLT: Gastrointestinal atonic and hypomotility disorders NEC	44 (33.6)	39 (30.0)	11 (8.8)
PT: Gastrooesophageal reflux disease	41 (31.3)	32 (24.6)	10 (8.0)

**SOC: Musculoskeletal and connective tissue disorders**	**56 (42.7)**	**65 (50.0)**	**21 (16.8)**
HLT: Metabolic bone disorders	25 (19.1)	37 (28.5)	9 (7.2)
PT: Osteoporosis	15 (11.5)	26 (20.0)	5 (4.0)
**SOC: Renal and urinary disorders**	**68 (51.9)**	**64 (49.2)**	**8 (6.4)**
HLT: Renal failure and impairment	51 (38.9)	43 (33.1)	0 (0)
PT: Chronic kidney disease	37 (28.2)	35 (26.9)	0 (0)
**SOC: Infections and infestations**	**58 (44.3)**	**56 (43.1)**	**15 (12.0)**
HLT: Coronavirus infections	18 (13.7)	17 (13.1)	2 (1.6)
PT: COVID-19	15 (11.5)	13 (10.0)	2 (1.6)
**SOC: Respiratory, thoracic and mediastinal disorders**	**50 (38.2)**	**43 (33.1)**	**17 (13.6)**
HLT: Bronchospasm and obstruction	12 (9.2)	20 (15.4)	8 (6.4)
HLT: Breathing abnormalities	15 (11.5)	8 (6.2)	5 (4.0)
HLT: Parenchymal lung disorders NEC	14 (10.7)	9 (6.9)	0 (0)
**SOC: Surgical and medical procedures**	**48 (36.6)**	**35 (26.9)**	**22 (17.6)**
**SOC: Nervous system disorders**	**33 (25.2)**	**38 (29.2)**	**16 (12.8)**
**SOC: Cardiac disorders**	**39 (29.8)**	**37 (28.5)**	**7 (5.6)**
**SOC: Neoplasms benign, malignant and unspecified**	**34 (26.0)**	**32 (24.6)**	**10 (8.0)**
**SOC: Reproductive system and breast disorders**	**38 (29.0)**	**26 (20.0)**	**12 (9.6)**
HLT: Prostatic neoplasms and hypertrophy	17 (13.0)	14 (10.8)	7 (5.6)
PT: Benign prostatic hyperplasia	17 (13.0)	14 (10.8)	7 (5.6)
**SOC: Endocrine disorders**	**32 (24.4)**	**31 (23.8)**	**12 (9.6)**
HLT: Thyroid hypofunction disorders	13 (9.9)	17 (13.1)	12 (9.6)
PT: Hypothyroidism	12 (9.2)	17 (13.1)	12 (9.6)
HLT: Hyperparathyroid disorders	17 (13.0)	13 (10.0)	0 (0)
**SOC: Blood and lymphatic system disorders**	**33 (25.2)**	**32 (24.6)**	**0 (0)**

HLT: Anemias NEC	15 (11.5)	9 (6.9)	0 (0)
PT: Anemia	15 (11.5)	8 (6.2)	0 (0)
**SOC: Psychiatric disorders**	**22 (16.8)**	**26 (20.0)**	**16 (12.8)**
**SOC: Immune system disorders**	**23 (17.6)**	**22 (16.9)**	**18 (14.4)**
**SOC: Congenital, familial and genetic disorders**	**23 (17.6)**	**19 (14.6)**	**2 (1.6)**
HLT: Renal disorders congenital	17 (13.0)	10 (7.7)	0 (0)
PT: Congenital cystic kidney disease	16 (12.2)	9 (6.9)	0 (0)
**SOC: Eye disorders**	**19 (14.5)**	**11 (8.5)**	**8 (6.4)**
**SOC: Injury, poisoning and procedural complications**	**15 (11.5)**	**12 (9.2)**	**5 (4.0)**

Pre-existing relevant medical conditions were obtained by interviewing the participant and/or reviewing the participant's medical records. Classification was done according to Medical Dictionary for Regulatory Activities (MedDRA) Terminology: System Organ Class (SOC), high-level term (HLT), and preferred term (PT). n (%), number (percentage) of participants in the indicated category; N, number of participants in the exposed population; 1-dose transplant group, immunocompromised kidney or lung transplantation recipients aged ≥18 years who received one dose of $AS01_E$-adjuvanted respiratory syncytial virus prefusion F protein vaccine (adjuvanted RSVPreF3); 2-dose transplant group, immunocompromised kidney or lung transplantation recipients aged ≥18 years who received two doses of adjuvanted RSVPreF3 one to two months apart; non-immunocompromised group, non-immunocompromised individuals aged ≥50 years who received one dose of adjuvanted RSVPreF3; NEC, not elsewhere classified.

Supplementary Table 3. Between-group assessments of RSV-A and RSV-B neutralizing titers in terms of adjusted GMT ratios and SRR differences (per-protocol set for humoral immunogenicity).

Timepoint	Groups (N)	Adjusted GMT ratio (95% CI)	SRR difference, % (95% CI)
**RSV-A neutralizing titers**			
Visit 2	Non-immunocompromised (34) / pooled transplant (72)	3.52 (2.26–5.48)	36.76 (16.79–54.21)
Visit 3	Non-immunocompromised (117) / pooled transplant (235)	1.63 (1.29–2.06)	28.43 (18.43–37.36)
Visit 4	Non-immunocompromised (110) / 1-dose transplant (114)	1.31 (1.01–1.68)	16.59 (4.04–28.64)
	Non-immunocompromised (110) / 2-dose transplant (110)	1.02 (0.80–1.30)	7.27 (-4.98–19.35)
	2-dose transplant (110) / 1-dose transplant (114)	1.26 (0.94–1.69)	9.31 (-3.51–21.83)
Visit 5	Non-immunocompromised (115) / 1-dose transplant (118)	1.13 (0.90–1.43)	7.37 (-5.46–19.96)
	Non-immunocompromised (115) / 2-dose transplant (104)	0.97 (0.77–1.21)	2.68 (-10.46–15.75)
	2-dose transplant (104) / 1-dose transplant (118)	1.16 (0.90–1.52)	4.69 (-8.47–17.68)
Visit 6	Non-immunocompromised (114) / 1-dose transplant (115)	1.20 (0.97–1.48)	-1.51 (-13.03–10.04)
	Non-immunocompromised (114) / 2-dose transplant (106)	0.87 (0.70–1.07)	-11.42 (-23.56–0.93)
	2-dose transplant (106) / 1-dose transplant (115)	1.36 (1.07–1.72)	9.91 (-2.48–22.14)
**RSV-B neutralizing titers**			
Visit 2	Non-immunocompromised (34) / pooled transplant (72)	3.37 (2.28–4.98)	53.35 (34.63–67.03)
Visit 3	Non-immunocompromised (117) / pooled transplant (235)	1.69 (1.33–2.15)	31.43 (22.06–39.75)
Visit 4	Non-immunocompromised (110) / 1-dose transplant (114)	1.29 (1.00–1.65)	12.17 (-0.30–24.28)
	Non-immunocompromised (110) / 2-dose transplant (110)	1.01 (0.80–1.26)	7.27 (-5.07–19.44)
	2-dose transplant (110) / 1-dose transplant (114)	1.28 (0.97–1.70)	4.90 (-7.83–17.44)
Visit 5	Non-immunocompromised (115) / 1-dose transplant (119)	1.08 (0.87–1.35)	-3.52 (-16.17–9.24)
	Non-immunocompromised (115) / 2-dose transplant (104)	0.93 (0.77–1.12)	-0.94 (-14.06–12.19)
	2-dose transplant (104) / 1-dose transplant (119)	1.15 (0.91–1.45)	-2.59 (-15.58–10.52)
Visit 6	Non-immunocompromised (114) / 1-dose transplant (115)	1.09 (0.90–1.33)	-0.61 (-12.48–11.28)
	Non-immunocompromised (114) / 2-dose transplant (106)	0.87 (0.72–1.04)	-6.97 (-19.33–5.50)

2-dose transplant (106) / 1-dose transplant (115)	1.25 (1.01–1.53)	6.36 (-6.10–18.73)

Between-group adjusted GMT ratios and SRR differences at each timepoint up to 12 months after (last) vaccination (V2, 3, 4, 5, and 6). In the 2-dose transplant group, the second vaccine dose was administered at V3. V3 titers were measured before dose 2.RSV, respiratory syncytial virus; GMT, geometric mean titer; SRR, seroresponse rate was defined as a ≥4-fold increase in titers compared to baseline; N, number of participants with available results at the given timepoint; CI, confidence interval; 1-dose transplant group, immunocompromised kidney or lung transplantation recipients aged ≥18 years who received one dose of $AS01_E$-adjuvanted RSV prefusion F protein vaccine (adjuvanted RSVPreF3); 2-dose transplant group, immunocompromised kidney or lung transplantation recipients aged ≥18 years who received two doses of adjuvanted RSVPreF3 one to two months apart; pooled transplant, combined 1-dose and 2-dose transplant groups; non-immunocompromised group, non-immunocompromised individuals aged ≥50 years who received one dose of adjuvanted RSVPreF3.

Supplementary Table 4. Median frequency of RSVPreF3-specific CD4+ T cells expressing ≥2 activation markers including ≥1 cytokine among CD40 ligand, 4-1BB, interleukin-2, tumor necrosis factor-α, interferon-ɣ, interleukin-13, and interleukin-17, per million CD4+ T cells, with participants in the transplant groups stratified by transplant type (per-protocol set for cell-mediated immunogenicity).

	1-dose transplant				2-dose transplant			
	Lung		Kidney		Lung		Kidney	
**Timepoint**	**N**	**Median frequency (IQR)**	**N**	**Median frequency (IQR)**	**N**	**Median frequency (IQR)**	**N**	**Median frequency (IQR)**
Visit 1	5	77.0 (1.0–161.0)	18	191.0 (65.0–647.0)	6	104.0 (1.0–277.0)	22	252.0 (89.0–469.0)
Visit 2	5	680.0 (447.0–1136.0)	19	7090.0 (1780.0–9688.0)	7	2811.0 (419.0–4134.0)	20	4609.0 (2837.5–9508.5)
Visit 3	6	651.5 (472.0–820.0)	16	2893.5 (1011.5–3468.5)	7	1477.0 (360.0–4177.0)	20	1848.5 (1435.0–2956.0)
Visit 4	5	480.0 (471.0–556.0)	18	2029.5 (896.0–3707.0)	6	3624.5 (1263.0–5741.0)	20	2983.0 (2060.0–3874.0)
Visit 5	3	447.0 (143.0–799.0)	19	1301.0 (416.0–2129.0)	5	993.0 (734.0–1308.0)	20	1703.5 (1155.5–2110.5)
Visit 6	2	1240.0 (81.0–2399.0)	15	1220.0 (413.0–1834.0)	4	739.5 (421.0–2013.5)	21	1219.0 (850.0–2584.0)

RSVPreF3-specific CD4+ T-cell responses in the two transplant groups, stratified by transplant type, before (first) vaccination (V1) and up to 12 months after (last) vaccination (V2, 3, 4, 5, and 6). In the 2-dose transplant group, the second vaccine dose was administered at V3. V3 titers were measured before dose 2. RSVPreF3, respiratory syncytial virus prefusion F protein 3; 1-dose transplant group, immunocompromised kidney or lung transplantation recipients aged ≥18 years who received one dose of $AS01_E$-adjuvanted RSVPreF3 vaccine (adjuvanted

RSVPreF3); 2-dose transplant group, immunocompromised kidney or lung transplantation recipients aged ≥18 years who received two doses of adjuvanted RSVPreF3 one to two months apart; N, number of participants with available results at the given timepoint; IQR, interquartile range.

Supplementary Table 5. Median frequency of RSVPreF3-specific CD4+ T cells expressing ≥2 activation markers including ≥1 cytokine among CD40 ligand, 4-1BB, interleukin-2, tumor necrosis factor-α, interferon-ɣ, interleukin-13, and interleukin-17, per million CD4+ T cells, with participants in the transplant groups stratified by mycophenolate compound use (per-protocol set for cell-mediated immunogenicity).

	1-dose transplant				2-dose transplant			
	MC		No MC		MC		No MC	
**Timepoint**	**N**	**Median frequency (IQR)**	**N**	**Median frequency (IQR)**	**N**	**Median frequency (IQR)**	**N**	**Median frequency (IQR)**
Visit 1	17	162.0 (65.0–647.0)	6	119.0 (1.0–568.0)	24	243.0 (89.0–440.0)	4	239.0 (116.0–674.0)
Visit 2	17	6144.0 (1771.0–8925.0)	7	2827.0 (447.0–7090.0)	22	4076.5 (1818.0–9638.0)	5	4164.0 (2939.0–4348.0)
Visit 3	15	1482.0 (532.0–3412.0)	7	820.0 (334.0–3124.0)	22	1766.5 (970.0–3256.0)	5	1652.0 (1627.0–1805.0)
Visit 4	16	1702.5 (516.5–3096.5)	7	827.0 (226.0–3774.0)	22	3465.5 (2216.0–4474.0)	4	1902.0 (1086.0–2549.0)
Visit 5	17	1258.0 (449.0–1834.0)	5	447.0 (319.0–2079.0)	21	1673.0 (1166.0–2102.0)	4	810.5 (393.5–1926.0)
Visit 6	13	1220.0 (418.0–1834.0)	4	900.5 (112.0–2028.5)	20	1297.5 (825.5–2600.5)	5	691.0 (666.0–850.0)

RSVPreF3-specific CD4+ T-cell responses in the two transplant groups, with participants stratified by mycophenolate compound (MC) use, before (first) vaccination (V1) and up to 12 months after (last) vaccination (V2, 3, 4, 5, and 6). In the 2-dose transplant group, the second vaccine dose was administered at V3. V3 titers were measured before dose 2. RSVPreF3, respiratory syncytial virus prefusion F protein 3; 1-

dose transplant group, immunocompromised kidney or lung transplantation recipients aged ≥18 years who received one dose of $AS01_E$-adjuvanted RSVPreF3 vaccine (adjuvanted RSVPreF3); 2-dose transplant group, immunocompromised kidney or lung transplantation recipients aged ≥18 years who received two doses of adjuvanted RSVPreF3 one to two months apart; N, number of participants with available results at the given timepoint; IQR, interquartile range.

Supplementary Table 6. Serious adverse events considered vaccine-related, fatal serious adverse events, and adverse events of special interest reported from vaccination until study end (exposed population).

Event	Age category onset (y)	Sex	Onset	Resolved	Study group	Vaccine-related[a]	SAE	Intensity grading[a]	Additional notes
SAE considered vaccine-related[a]									
Gastroenteritis	50–59	Female	2 days post-dose 1	Yes (24 days)	1-dose transplant	Yes	Yes	2	Lung transplant recipient. In this participant, there is indirect evidence suggesting a bacterial infectious etiology, as there was an increased white blood cell count at time of admission, which normalized after 2 days of antibiotics treatment with azithromycin. Although stool culture was negative for bacteria, the yield of conventional stool cultures is known to be low in both immunocompetent and transplant populations. Additionally, the participant received mycophenolate mofetil, tacrolimus and sirolimus,

									all of which could be associated with diarrhea and represent plausible alternative contributors to the clinical presentation.
Fatal SAEs									
Post-transplant lymphoproliferative disorder[b]	≥60	Female	318 days post-dose 1	No, fatal	1-dose transplant	No	Yes	3	Lung transplant recipient.
Myelodysplastic syndrome with excess blasts[b]	≥60	Male	Approx. 1 month post-dose 1	No, fatal	1-dose transplant	No	Yes	3	Lung transplant recipient.
Acute respiratory failure, *Aspergillus* infection, Pneumonia Staphylococcal[b]	≥60	Female	355 days post-dose 2	No, fatal	2-dose transplant	No	Yes	3	Lung transplant recipient.
Nocardiosis	≥60	Female	323 days post-dose 1	No, fatal	1-dose transplant	No	Yes	3	Lung transplant recipient.
Acute respiratory failure	≥60	Male	83 days post-dose 1	No, fatal	2-dose transplant	No	Yes	3	Lung transplant recipient.

Pneumonia	50–59	Female	31 days post-dose 1	No, fatal	2-dose transplant	No	Yes	3	Lung transplant recipient.
AESIs									
Immune thrombocytopenia	50–59	Male	169 days post-dose 1	Yes (47 days)	1-dose transplant	No	Yes	3	Kidney transplant recipient.
Scleroderma	18–49	Female	368 days post-dose 1	No (Recovering/ resolving)	1-dose transplant	No	No	2	Lung transplant recipient.
Optic ischemic neuropathy	≥60	Female	324 days post-dose 2	No	2-dose transplant	No	Yes	1	Kidney transplant recipient.
Exacerbated psoriasis	≥60	Male	17 days post-dose 1	Yes (163 days)	Non-immunocompromised	No	No	2	Exacerbation of pre-existing condition.
Renal impairment	≥60	Male	148 days post-dose 2	Yes (64 days)	2-dose transplant	No	Yes	2	Kidney transplant recipient. The participant had a kidney biopsy performed one day after the onset of the event, which was suspicious of rejection of the kidney graft (reported as borderline

									[suspicious] for acute T-cell-mediated rejection). Relevant medical history included diabetic nephropathy, BK virus nephropathy and acute rejection (approximately 6 months post-transplant).
Atrial fibrillation	≥60	Male	337 days post-dose 2	No	2-dose transplant	No	No	2	Kidney transplant recipient. Detected during a routine medical checkup. The participant had a relevant medical history of type 2 diabetes mellitus, chronic kidney disease, atrial tachycardia, cardiac murmur, thrombosis, hypertension, hyperlipidemia, and congenital cystic kidney disease.
Atrial fibrillation	≥60	Male	365 days post-dose 1	Yes (7 days)	Non-immunocompromised	No	Yes	3	Reported during a hospitalization for *Mycoplasma* pneumonia. Diagnosed by a multiparameter monitor. The participant had a relevant medical history of alcohol consumption and tobacco smoking/consumption, and a

			medical history of colon cancer that was resolved prior to study screening. The participant received anticoagulant medication and was on rate and rhythm control medications.

y, year; SAE, serious adverse event; AESI, adverse event of special interest; 1-dose transplant group, immunocompromised kidney or lung transplantation recipients aged ≥18 years who received one dose of $AS01_E$-adjuvanted respiratory syncytial virus prefusion F protein vaccine (adjuvanted RSVPreF3); 2-dose transplant group, immunocompromised kidney or lung transplantation recipients aged ≥18 years who received two doses of adjuvanted RSVPreF3 one to two months apart; non-immunocompromised group, non-immunocompromised individuals aged ≥50 years who received one dose of adjuvanted RSVPreF3. [a]By investigator assessment. [b]Occurred post-study conclusion.

Supplementary Table 7. Study objectives.

Objectives	Endpoints and estimands
**Primary**	
To evaluate the humoral immune response against RSV-A following the first and the second dose of adjuvanted RSVPreF3 within 2-dose group in kidney or lung solid organ transplant patients.	RSV-A serum neutralizing titers expressed as MGI post-dose 2 (Visit 4) over post-dose 1 (Visit 3) in kidney or lung solid organ transplant patients in the 2-dose transplant group.
To evaluate the humoral immune response against RSV-B following the first and the second dose of adjuvanted RSVPreF3 within 2-dose group in kidney and lung solid organ transplant patients.	RSV-B serum neutralizing titers expressed as MGI post-dose 2 (Visit 4) over post-dose 1 (Visit 3) in kidney and lung solid organ transplant patients in the 2-dose transplant group.
**Secondary – immunogenicity**	
To evaluate the humoral immune response to adjuvanted RSVPreF3 until 12 months post-study intervention administration in all groups.	RSV-A and RSV-B serum neutralizing titers expressed as GMT, at pre-study intervention administration (Visit 1), Visit 2 (in a subset of participants), Visit 3, Visit 4, Visit 5, and Visit 6 in kidney and lung solid organ transplant patients (1-dose and 2-dose transplant group), and non-immunocompromised adults (non-immunocompromised group).<br><br>RSV-A and RSV-B serum neutralizing titers expressed as group GMT ratio (non-immunocompromised over combined transplant groups [pooled 1-dose and 2-dose transplant groups]) at Visit 2 (in a subset of participants) and Visit 3, 2-dose transplant over 1-dose transplant, non-immunocompromised over 1-dose transplant, and non-immunocompromised over 2-dose transplant at Visit 4, Visit 5, and Visit 6.<br><br>RSV-A and RSV-B serum neutralizing titers expressed as MGI at Visit 2 (in a subset of participants) over Visit 1 and Visit 3 over Visit 1 in non-

	immunocompromised and combined transplant groups, and at Visit 4, Visit 5, and Visit 6 over Visit 1 in 1-dose transplant, 2-dose transplant, and non-immunocompromised groups.
To evaluate the cell-mediated immune response following adjuvanted RSVPreF3 administration in a subset of participants in all groups.	Cell-mediated immune response expressed as group geometric mean of the frequency of RSVPreF3-specific CD4+ and/or CD8+ T cells expressing at least two activation markers including at least one cytokine among CD40L, 4-1BB, IL-2, TNF-α, IFN-γ, IL-13, and IL-17 at pre-study intervention administration, Visit 2, Visit 3, Visit 4, Visit 5, and Visit 6, in a subset of 1-dose transplant, 2-dose transplant, and non-immunocompromised participants.
**Secondary – safety**	
To evaluate the safety and reactogenicity following adjuvanted RSVPreF3 administration in all groups.	Percentage of participants reporting each solicited administration-site event with onset within 7 days post-study intervention administration (i.e., the day of vaccination and 6 subsequent days).<br>Percentage of participants reporting each solicited systemic event with onset within 7 days post-study intervention administration (i.e., the day of vaccination and 6 subsequent days).<br>Percentage of participants reporting unsolicited AEs within 30 days post-study intervention administration (i.e., the day of vaccination and 29 subsequent days).<br>Percentage of participants reporting SAEs after study intervention administration (Day 1) up to study end (Visit 6).<br>Percentage of participants reporting pIMDs after study intervention administration (Day 1) up to study end (Visit 6).<br>Percentage of participants reporting SAEs related to study intervention administration after study intervention administration (Day 1) up to study end (Visit 6).

	Percentage of participants reporting pIMDs related to study intervention administration after study intervention administration (Day 1) up to study end (Visit 6).<br>Percentage of participants reporting fatal SAEs after study intervention administration (Day 1) up to study end (Visit 6).<br>Percentage of participants reporting AESIs (specific to kidney and lung solid organ transplant patients) after first study intervention administration (Day 1) up to study end (Visit 6).

RSV, respiratory syncytial virus; 1-dose transplant group, immunocompromised kidney or lung transplantation recipients aged ≥18 years who received one dose of $AS01_E$-adjuvanted RSV prefusion F protein vaccine (adjuvanted RSVPreF3); 2-dose transplant group, immunocompromised kidney or lung transplantation recipients aged ≥18 years who received two doses of adjuvanted RSVPreF3 one to two months apart; non-immunocompromised group, non-immunocompromised individuals aged ≥50 years who received one dose of adjuvanted RSVPreF3; MGI, mean geometric increase; GMT, geometric mean titer; CD40L, CD40 ligand; IL, interleukin; TNF-α, tumor necrosis factor alpha; IFN-γ, interferon gamma; AE, adverse event; SAE, serious AE; pIMD, potential immune-mediated disease; AESI, AE of special interest.

Supplementary Figure 1. RSV-A and RSV-B neutralizing titers in terms of seroresponse rates (per-protocol set for humoral immunogenicity).

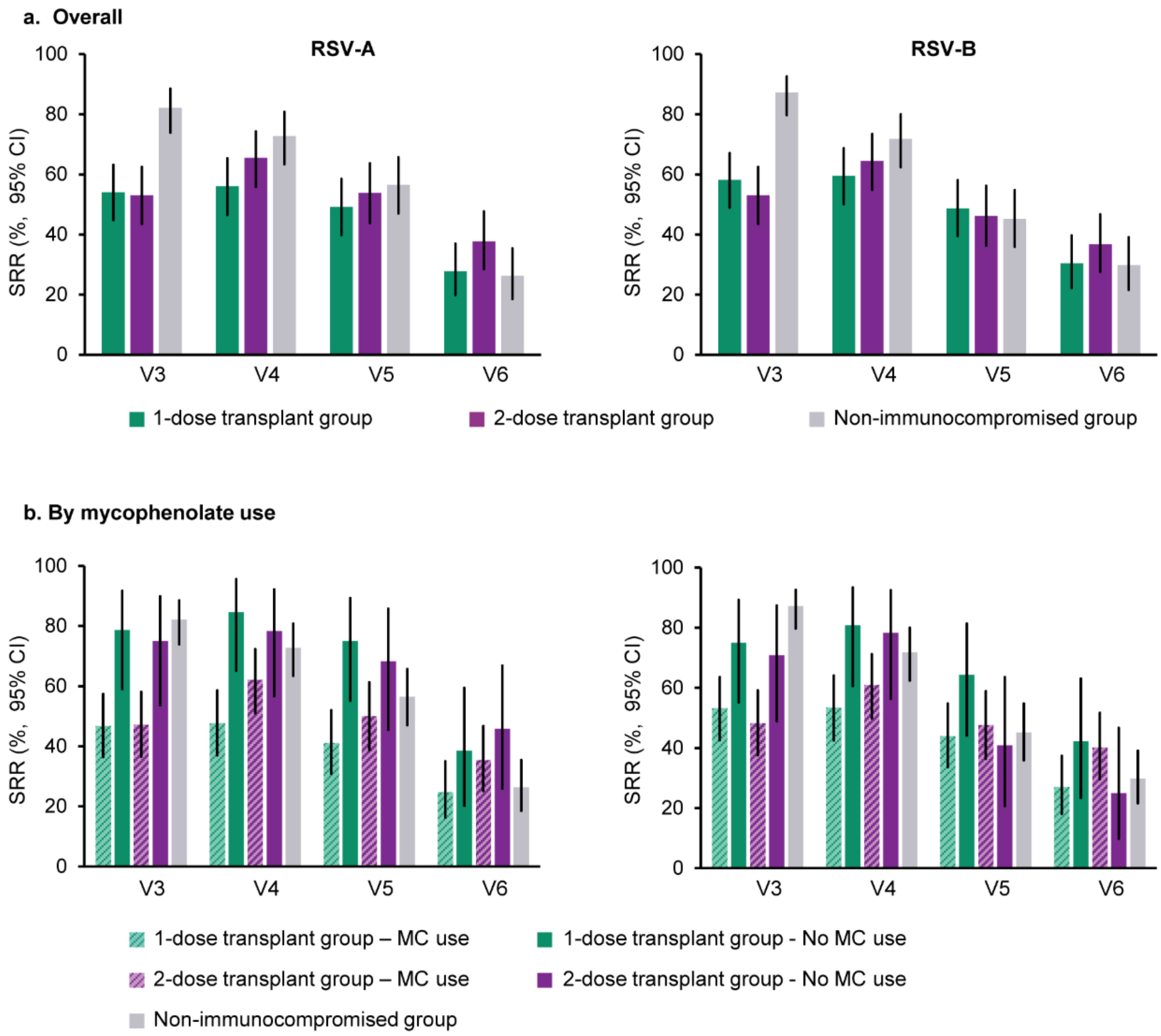

a) RSV-A and RSV-B neutralizing titers in terms of seroresponse rates (SRRs) in the three study groups before (first) vaccination (V1) and up to 12 months after (last) vaccination (V3, 4, 5, and 6). In the 2-dose transplant group, the second vaccine dose was administered at V3. V3 titers were measured before dose 2. b) RSV-A and RSV-B neutralizing titers in terms of SRRs in the three study groups, with participants in the transplant groups stratified by mycophenolate compound (MC) use. SRR was defined as a ≥4-fold increase in neutralizing titers compared to baseline. The 1-dose transplant group is shown in green, the 2-dose transplant group in purple, and the non-immunocompromised control group in grey. Sample sizes at each visit (V3/V4/V5/V6) were as follows: 1-dose transplant group (123/116/119/116 [RSV-A] or 123/116/120/116 [RSV-B]); 2-dose transplant group (114/111/105/107 [RSV-

A/RSV-B]); and non-immunocompromised controls (117/110/115/114 [RSV-A/RSV-B]). RSV, respiratory syncytial virus; CI, confidence interval; 1-dose transplant group, immunocompromised kidney or lung transplantation recipients aged ≥18 years who received one dose of $AS01_E$-adjuvanted RSV prefusion F protein vaccine (adjuvanted RSVPreF3); 2-dose transplant group, immunocompromised kidney or lung transplantation recipients aged ≥18 years who received two doses of adjuvanted RSVPreF3 one to two months apart; non-immunocompromised group, non-immunocompromised individuals aged ≥50 years who received one dose of adjuvanted RSVPreF3. Source data are provided as a Source Data file.

Supplementary Figure 2. Gating strategy of flow cytometry for assessment of cell-mediated immunogenicity.

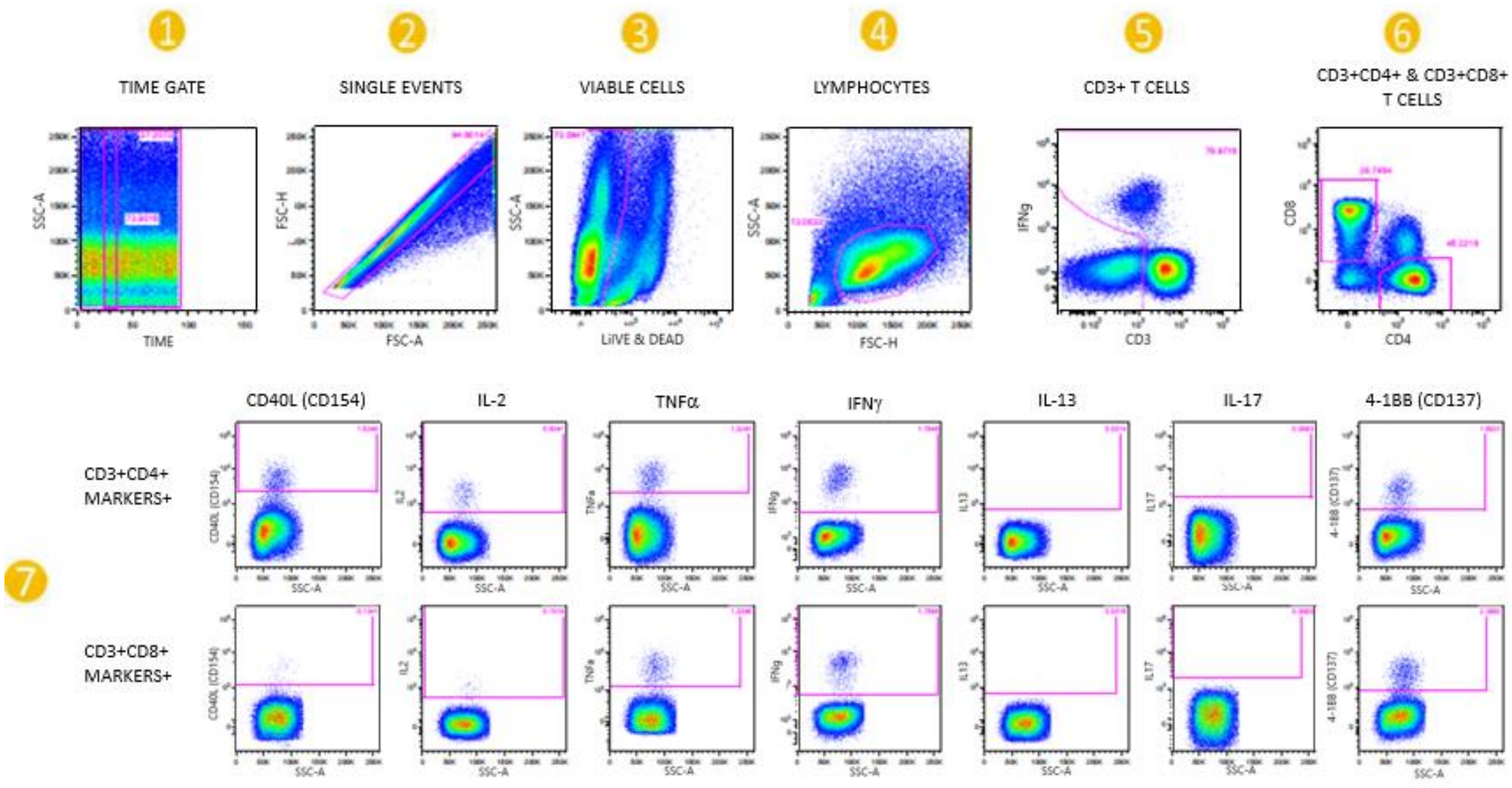

1 = time gate; 2 = exclusion of doublets; 3 = exclusion of dead cells; 4 = lymphocyte gate; 5 = gating on T cells (CD3+); 6 = CD4+ vs CD8+ T cells; 7 = analysis of activation markers

Events were sequentially gated as follows: (1) a time gate was created to ensure that the events were captured once the flow stream was stabilized SSC-A versus time density plot); (2) a gate was made on singlets (doublets were excluded [FSC-H versus FSC-A plot]); (3) a gate was made on live cells (dead cells were excluded [live-dead dye parameter versus SSC-A plot]); (4) a lymphocyte gate was created based on size and granularity (FSC-H [size] versus SSC-A [granularity] plot); (5) CD3+ T cells were gated (CD3 versus IFN-γ plot; as the CD3 marker expression is downregulated after stimulation, a polygon gate was used); (6) CD4+ (CD4+CD8-) and CD8+ (CD8+CD4-) T cells were then gated (CD4 versus CD8 plot); and finally (7) activation markers (SSC-A versus CD40L, IL-2, IL-13, IL-17, TNF-α, IFN-γ and 4-1BB, respectively) were assessed in both CD4+CD8- T cells and CD8+CD4- T cells. A Boolean gating analysis was used to identify all combinations of T cells expressing at least two activation markers, including at least one cytokine, which were summed to generate polypositive T-cell counts. These counts were subsequently expressed as frequencies of polypositive T cells per million CD4+ or CD8+ T cells. Polypositive T-cell frequencies obtained under background conditions were subtracted from those obtained under stimulated

conditions, to calculate RSVPreF3-specific polypositive T-cell frequencies per million CD4+ or CD8+ T cells. A detailed list of the antibodies that were used is provided in the Supplementary Notes. CD40L, CD40 ligand; IL, interleukin; TNF-α, tumor necrosis factor alpha; IFN-γ, interferon gamma; FSC-A/H, forward scatter-area/height; SSC-A, side scatter-area.

Supplementary Notes

Eligibility criteria

Inclusion criteria

Participants were eligible to be included in the study only if all of the following criteria applied:

- For all participants:
  - Participants and/or participants parent(s)/legally authorized representative(s) (LAR[s]) who, in the opinion of the investigator, could and would comply with the requirements of the protocol (e.g., completion of the paper diary cards, return for follow-up Visits, ability to access and utilize a phone or other electronic communications, have regular contact to allow evaluation during the study).
  - Participants living in the general community or in an assisted-living facility that provides minimal assistance could be enrolled, such that the participant was primarily responsible for self-care and activities of daily living.
  - Written or witnessed informed consent obtained from the participant/participant's parent(s)/LAR(s) (participant had to be able to understand the informed consent) prior to performance of any study-specific procedure.
  - Female participants of nonchildbearing potential could be enrolled in the study.
  - Female participants of childbearing potential could be enrolled in the study if the participant had practiced adequate contraception from one month prior to study intervention administration, agreed to continue adequate contraception until study end, and had a negative pregnancy test on the day of and prior to study intervention administration.
- For immunocompromised participants:

- A male or female participant, ≥18 years of age at the time of signing the informed consent form or informed assent form.
- Written informed assent obtained from the participant (participant must be able to understand the informed assent) if he/she was less than legal age of consent, or written informed consent obtained from the participant if the participant had achieved legal age of consent.
- Participant who had received an ABO-compatible allogeneic kidney or lung transplant (allograft) more than 12 months (365 days) prior to the first study intervention administration.
- Participant receiving maintenance immunosuppressive therapy for the prevention of allograft rejection.
- For kidney transplant recipients: Participant with stable kidney function, stability defined as less than 20% variability between last two results of estimated glomerular filtration rate (eGFR) or in the opinion of the investigator after investigator review of more than the last two results of eGFRs and based on medical history.
- For lung transplant recipients: Participant with stable lung function, with stability defined as the stability in the forced expiratory volume in one second (FEV1) compared to post-transplant baseline FEV1 and based on medical history of the last three months, in the opinion of the investigator.

- For non-immunocompromised participants:
    - A male or female, ≥50 years of age at the time of signing the informed consent form.
    - Healthy participants as established by medical history and clinical examination before entering the study.
    - Participants who were medically stable in the opinion of the investigator at the time of first study intervention administration.

- Participants with chronic stable medical conditions with or without specific treatment, such as diabetes mellitus, hypertension, or cardiac disease, were allowed to participate in this study if considered by the investigator as medically stable.

Exclusion criteria

Participants were excluded from the study if any of the following criteria applied:

- Medical conditions:
    - History of any reaction or hypersensitivity likely to be exacerbated by any component of the study intervention.
    - Acute or chronic clinically significant cardiovascular or hepatic functional abnormality as determined by physical examination or laboratory screening tests.
    - Recurrent or uncontrolled neurological disorders or seizures. Participants with medically controlled chronic neurological diseases could be enrolled in the study as per investigator assessment, provided that their condition would allow them to comply with the requirements of the protocol (e.g., completion of the diary cards, attend study site Visits). Study participants could decide to assign a caregiver to help them complete some of the study procedures.
    - Any history of dementia or any medical condition that moderately or severely impaired cognition.
    - Any condition which, in the judgment of the investigator, would have made intramuscular injection unsafe.
    - Significant underlying illness that in the opinion of the investigator would have expected to prevent completion of the study (e.g., life-threatening disease likely to limit survival up to study end).
    - Acute disease and/or fever at the time of study intervention administration. Fever was defined as temperature ≥38°C/100.4°F determined by oral or axillary route. However,

participants with a minor illness (such as mild diarrhea, mild upper respiratory infection) without fever could be enrolled at the discretion of the investigator.

- Bedridden participants.
- Any other clinical condition that, in the opinion of the investigator, might have posed additional risk to the participant due to participation in the study.

- Prior/concomitant therapy:
  - Use of any investigational or non-registered product (drug, vaccine, or medical device) other than the study intervention administration during the period beginning 30 days before the first dose of study intervention administration (Day -30 to Day 1), or their planned use during the study period (up to Visit 6).
  - Previous vaccination with the same antigen (RSV) containing vaccine as that of the study intervention, including investigational RSV vaccines.
  - Planned or actual administration of a vaccine not foreseen by the study protocol in the period starting 30 days before the first dose of study intervention administration and ending 30 days after the last dose of study intervention administration. In the case of COVID-19 and inactivated/subunit/split influenza vaccines, this time window could be decreased to 14 days before and after each study intervention administration.
- Prior/concurrent clinical study experience:
  - Concurrently participating in another clinical study, at any time during the study period, in which the participant was or would be exposed to an investigational or a non-investigational intervention (drug/invasive medical device).
- Other exclusion criteria:
  - Pregnant or lactating female participant.

- Female participant planning to become pregnant or planning to discontinue contraceptive precautions.
- History of chronic alcohol consumption and/or drug abuse as deemed by the investigator to render the potential participant unable/unlikely to provide accurate safety reports or comply with study procedures.
- Participation of any study personnel or their immediate dependents, family, or household members.
- Planned move during the study period that would prohibit participating in the study until study end.

- Specific exclusion criteria for kidney/lung transplant recipients:
    - More than one organ transplanted (i.e., kidney-liver or kidney-other organ[s] transplanted). Dual organ transplant was allowed (double kidney or double lung).
    - History of events that, in the opinion of the investigator, may have put the participant at increased risk for chronic allograft dysfunction.
    - Participant with an episode of allograft rejection over the three months (90 days) prior to the first study intervention administration.
    - Histologic evidence of chronic allograft injury.
    - Active treatment for acute rejection.
    - Current diagnosis of malignancy (except non-melanoma skin cancer that did not require systemic therapy).
    - Any autoimmune conditions or potential immune-mediated diseases (pIMDs) that in the opinion of the investigator may have put the participant at increased risk.

- Any confirmed or suspected human immunodeficiency virus (HIV) infection or primary immunodeficiency disease or ongoing cytomegalovirus (CMV) infection with a viremia >200 IU/mL.
- Use of anti-CD20 or other B-cell monoclonal antibody agents (e.g., rituximab) as induction, maintenance and/or therapeutic immunosuppressive therapy for the prevention of allograft rejection within nine months (274 days) of first dose of study.
- Use of investigational and non-registered immunosuppressants at the local/country level, unless specifically prescribed for the prevention of allograft rejection, which are non-registered, and:
    - available locally through compassionate use programs,
    - submitted for and pending local/country registration,
    - approved and registered for use in other countries with well-documented Summary of Product Characteristics or Prescribing Information. The name of the active component(s) of these immunosuppressants had to be provided in the concomitant medication listing.
- Evidence or high suspicion, in the opinion of the investigator, of noncompliance or nonadherence to use of induction and/or maintenance immunosuppressant therapies.
- Any clinically significant hematologic (hemoglobin level, white blood cell, lymphocyte, neutrophil, eosinophil, platelet, red blood cell count, erythrocyte mean corpuscular volume) and/or biochemical (alanine transaminase, aspartate transaminase, creatinine, blood urea nitrogen) laboratory abnormality.
- For kidney transplant recipients:
    - Previous allograft loss secondary to recurrent primary kidney disease. Multiple consecutive kidney transplants were allowed if the reason for a previous allograft loss was not recurrent primary kidney disease.

    - Evidence of significant proteinuria/albuminuria in the opinion of the investigator.
  - For lung transplant recipients:
    - At study intervention administration visit, diagnosis of documented acute pulmonary infection within the two prior weeks, based on the following: clinical, radiological, and physiological deterioration; or isolation of an organism from a clinically relevant bronchoalveolar lavage fluid culture.
    - Patients with diagnosis of chronic lung allograft dysfunction, defined as a decrement of 20% or more in FEV1 compared to post-transplant baseline FEV1.
- Specific exclusion criteria for non-immunocompromised participants:
  - Any confirmed or suspected immunosuppressive or immunodeficient condition resulting from disease (e.g., current malignancy, HIV) or immunosuppressive/cytotoxic therapy (e.g., medication used during cancer chemotherapy, organ transplantation, or to treat autoimmune disorders), based on medical history and physical examination (no laboratory testing required).
  - Unstable serious chronic illness.
  - Chronic administration of immune-modifying drugs (defined as more than 14 consecutive days in total) and/or administration of long-acting immune-modifying treatments or planned administration at any time up to the end of the study.
    - Up to three months prior to the study intervention administration:
      - For corticosteroids, this meant prednisone equivalent ≥20 mg/day, or equivalent. Inhaled, topical and intra-articular steroids were allowed.
      - Administration of immunoglobulins and/or any blood products or plasma derivatives.

- Up to six months prior to study intervention administration: long-acting immune-modifying drugs including among others, immunotherapy (e.g., TNF-inhibitors), monoclonal antibodies, antitumoral medication.

Antibodies used for flow cytometry

- CD3, fluorochrome BV605 (BioLegend, cat. no.: 317322, clone: OKT3)
- CD4, fluorochrome BB700 (BD, cat. no.: 566392, clone: SK3)
- CD8, fluorochrome APC-H7 (BD, cat. no.: 641400, clone: SK1)
- IL-2, fluorochrome APC-R700 (Pharmingen, cat. no.: 565136, clone: MQ1-17H12)
- TNFα, fluorochrome PeCy7 (Pharmingen, cat. no.: 557647, clone: Mab11)
- IFNγ, fluorochrome FITC (Pharmingen, cat. no.: 554551, clone: 4S.B3)
- IL-13, fluorochrome APC (BioLegend, cat. no.: 501907, clone: JES10-5A2)
- IL-17, fluorochrome BV785 (BioLegend, cat. no.: 512338, clone: BL168)
- CD40L, fluorochrome PE (Miltenyi Biotech, cat. no.: 130-113-613, clone: REA238)
- 4-1BB, fluorochrome BV421 (BioLegend, cat. no.: 309820, clone: 4B4-1)

Specificity of each antibody used for flow cytometry was confirmed during assay development through a Fluorescence Minus One experiment, ensuring the signal detected in the dedicated channel was only present when the antibody was present and that it did not impact the fluorescence in the other channels. In addition, each antibody lot was titrated to ensure the use at optimal dilution, identified as providing the best signal-to-noise ratio and stain index.

Intensity grading of solicited events and adverse events

The investigator made an assessment of the intensity of each solicited event, AE, AESI, and SAE reported during the study and assigned it to one of three grades:

Event	Grade	Parameter
**Administration-site solicited events**		

Pain	1	Mild: any pain neither interfering with nor preventing normal everyday activities
	2	Moderate: painful when limb is moved and interferes with normal everyday activities
	3	Severe: significant pain at rest that prevents normal everyday activities
Swelling/redness	1	Mild: greatest surface diameter >20 – ≤50 mm
	2	Moderate: greatest surface diameter >50 – ≤100 mm
	3	Severe: greatest surface diameter >100 mm
**Systemic solicited events**		
Fever	1	Mild: temperature ≥38.0°C (100.4°F) – ≤38.5°C (101.3°F)
	2	Moderate: temperature >38.5°C (101.3°F) – ≤39.0°C (102.2°F)
	3	Severe: temperature >39.0°C (102.2°F)
Fatigue	1	Mild: easily tolerated
	2	Moderate: interferes with normal activity
	3	Severe: prevents normal activity
Myalgia/arthralgia	1	Mild: present but does not interfere with activity
	2	Moderate: interferes with normal activity
	3	Severe: prevents normal activity
**Unsolicited AE/SAE/AESI**		
Any	1	Mild: usually transient and may require only minimal treatment or therapeutic intervention. The event does not generally interfere with usual activities of daily living
	2	Moderate: usually alleviated with additional specific therapeutic intervention. The event interferes with usual activities of daily living, causing discomfort but poses no significant or permanent risk of harm to the research participant
	3	Severe: interrupts usual activities of daily living, or significantly affects clinical status, or may require intensive therapeutic intervention

AE, adverse event; SAE, serious AE; AESI, AE of special interest.

Sample size determination

The study targeted approximately 375 eligible enrolled participants, with a solid organ transplant population of approximately 250 participants to be randomized (1:1) into two transplant groups at Visit 1: 125 per group. For the non-immunocompromised population, a sample size of 125 participants was planned. An attrition rate of 20% was anticipated from enrolled to evaluable participants. In each group, approximately 38 participants were targeted for inclusion in the cell-mediated immunogenicity subset.

The descriptive nature of the study and prior immunogenicity results with adjuvanted RSVPreF3 informed the overall sample size. A power calculation was performed during the study design to inform sample size determination and to ensure that meaningful differences in immune responses for the primary objective could be detected. The primary study objective (i.e., comparing the response post-dose 2 to post-dose 1 in the 2-dose transplant group) was specified with one hundred evaluable participants. Analyzing a paired t-test with 1-sided alpha of 2.5% and a standard deviation of differences of 0.5 (inflated from 0.4 to account for the immunocompromising conditions), the detectable fold-increases were 1.39 with 80% power and 1.46 with 90% power. With a standard deviation of differences of 0.4, the detectable fold-increases were 1.30 with 80% power and 1.35 with 90% power. These calculations were intended to support study design, rather than to define formal success thresholds. Given the descriptive nature of the study, no multiplicity adjustment was applied.

Overview of ethics committees

1. Metro North Health Human Research Ethics Committee, Herston, Australia
2. University of British Columbia Clinical Research Ethics Board, Vancouver, Canada
3. University of Alberta Human Research Ethics Board, Edmonton, Canada
4. Advarra, Aurora, Canada
5. University Health Network Research Ethics Board, Toronto, Canada
6. Western Health Sciences Research Ethics Board, London, Canada
7. Ethikkommission der Universität Duisburg-Essen, Langen, Germany
8. CET Interaziendale AOU Maggiore della Carità di Novara, Novara, Italy
9. Nagoya Daini Red Cross Hospital Institutional Review Board, Nagoya, Japan
10. Tokyo Medical University Hachioji Medical Center Institutional Review Board, Tokyo, Japan
11. The Central Institutional Review Board for the Fujita Health University Hospitals, Toyoake, Japan
12. Hyogo Prefectural Nishinomiya Hospital Institutional Review Board, Nishinomiya, Japan
13. Fukuoka University Hospital Institutional Review Board, Fukuoka, Japan
14. Okayama University Hospital Institutional Review Board, Okayama, Japan
15. Medical Corporation Shinanokai Shinanosaka Clinic Institutional Review Board, Tokyo, Japan
16. Japanese Red Cross Kumamoto Hospital Institutional Review Board, Kumamoto, Japan
17. Seoul National University Hospital Institutional Review Board, Seoul, Republic of Korea
18. Yonsei University Health System, Severance Hospital, Institutional Review Board, Seoul, Republic of Korea
19. Asan Medical Center Institutional Review Board, Seoul, Republic of Korea
20. CEIm Hospital Clínico San Carlos, Madrid, Spain
21. WCG IRB, Princeton, United States
22. University of Kentucky Office of Research Integrity, Lexington, United States

23. Baylor Scott & White Research Institute, Dallas, United States

24. Columbia University Institutional Review Board, New York, United States

25. University of Minnesota Institutional Review Board, Minneapolis, United States

CONSORT 2025 checklist

Section / Topic	No	CONSORT 2025 checklist item description	Reported on page no.
**Title and abstract**			
Title and structured abstract	1a	Identification as a randomised trial	1
	1b	Structured summary of the trial design, methods, results, and conclusions	3
**Open science**			
Trial registration	2	Name of trial registry, identifying number (with URL) and date of registration	3, 22
Protocol and statistical analysis plan	3	Where the trial protocol and statistical analysis plan can be accessed	22
Data sharing	4	Where and how the individual de-identified participant data (including data dictionary), statistical code and any other materials can be accessed	28
Funding and conflicts of interest	5a	Sources of funding and other support (e.g., supply of drugs), and role of funders in the design, conduct, analysis and reporting of the trial	35-36
	5b	Financial and other conflicts of interest of the manuscript authors	36
**Introduction**			
Background and rationale	6	Scientific background and rationale	4-6
Objectives	7	Specific objectives related to benefits and harms	5-6
**Methods**			
Patient and public involvement	8	Details of patient or public involvement in the design, conduct and reporting of the trial	NA
Trial design	9	Description of trial design including type of trial (e.g., parallel group, crossover), allocation ratio, and framework (e.g., superiority, equivalence, non-inferiority, exploratory)	22
Changes to trial protocol	10	Important changes to the trial after it commenced including any outcomes or analyses that were not prespecified, with reason	NA
Trial setting	11	Settings (e.g., community, hospital) and locations (e.g., countries, sites) where the trial was conducted	22
Eligibility criteria	12a	Eligibility criteria for participants	22-23
	12b	If applicable, eligibility criteria for sites and for individuals delivering the interventions (e.g., surgeons, physiotherapists)	NA

Intervention and comparator	13	Intervention and comparator with sufficient details to allow replication. If relevant, where additional materials describing the intervention and comparator (e.g., intervention manual) can be accessed	23
Outcomes	14	Pre-specified primary and secondary outcomes, including the specific measurement variable (e.g., systolic blood pressure), analysis metric (e.g., change from baseline, final value, time to event), method of aggregation (e.g., median, proportion), and time point for each outcome	24 and Supplement
Harms	15	How harms were defined and assessed (e.g., systematically, non-systematically)	26
Sample size	16a	How sample size was determined, including all assumptions supporting the sample size calculation	26 and Supplement
	16b	Explanation of any interim analyses and stopping guidelines	NA
Randomisation:			
Sequence generation	17a	Who generated the random allocation sequence and the method used	24
	17b	Type of randomisation and details of any restriction (e.g., stratification, blocking and block size)	24
Allocation concealment mechanism	18	Mechanism used to implement the random allocation sequence (e.g., central computer/telephone; sequentially numbered, opaque, sealed containers), describing any steps to conceal the sequence until interventions were assigned	24
Implementation	19	Whether the personnel who enrolled and those who assigned participants to the interventions had access to the random allocation sequence	NA – open-label study
Blinding	20a	Who was blinded after assignment to interventions (e.g., participants, care providers, outcome assessors, data analysts)	NA – open-label study
	20b	If blinded, how blinding was achieved and description of the similarity of interventions	NA
Statistical methods	21a	Statistical methods used to compare groups for primary and secondary outcomes, including harms	26-27
	21b	Definition of who is included in each analysis (e.g., all randomised participants), and in which group	7-8
	21c	How missing data were handled in the analysis	27
	21d	Methods for any additional analyses (e.g., subgroup and sensitivity analyses), distinguishing prespecified from post-hoc	27-28, Figure and table footnotes, where applicable
**Results**			
Participant flow, including flow diagram	22a	For each group, the numbers of participants who were randomly assigned, received intended intervention, and were analysed for the primary outcome	Fig3
	22b	For each group, losses and exclusions after randomisation, together with reasons	Fig3

Recruitment	23a	Dates defining the periods of recruitment and follow-up for outcomes of benefits and harms	22
	23b	If relevant, why the trial ended or was stopped	NA
Intervention and comparator delivery	24a	Intervention and comparator as they were actually administered (e.g., where appropriate, who delivered the intervention/comparator, how participants adhered, whether they were delivered as intended [fidelity])	Fig3
	24b	Concomitant care received during the trial for each group	NA
Baseline data	25	A table showing baseline demographic and clinical characteristics for each group	Table 1
Numbers analysed, outcomes and estimation	26	For each primary and secondary outcome, by group:<br>● the number of participants included in the analysis<br>● the number of participants with available data at the outcome time point<br>● result for each group, and the estimated effect size and its precision (such as 95% confidence interval)<br>● for binary outcomes, presentation of both absolute and relative effect size	Fig3
Harms	27	All harms or unintended events in each group	12-15, Table 4
Ancillary analyses	28	Any other analyses performed, including subgroup and sensitivity analyses, distinguishing pre-specified from post-hoc	11-12, Table 3, Fig5b/c
**Discussion**			
Interpretation	29	Interpretation consistent with results, balancing benefits and harms, and considering other relevant evidence	15-21
Limitations	30	Trial limitations, addressing sources of potential bias, imprecision, generalisability, and, if relevant, multiplicity of analyses	20

Study Protocol

Clinical Study Protocol

**Primary Study Intervention**	GSK's investigational respiratory syncytial virus (RSV) vaccine BIO RSV OA=ADJ (GSK3844766A)
**Study Identifier**	219900 (RSV OA=ADJ-023)
**EU CT Number**	2023-503951-81-00
**Approval Date**	11 Oct 2023
**Title**	A Phase 2b, randomized, controlled, open-label study to evaluate the immune response and safety of the RSVPreF3 OA investigational vaccine in adults (≥18 years of age) when administered to lung and renal transplant recipients comparing 1 versus 2 doses and compared to healthy controls (≥50 years of age) receiving 1 dose
**Brief Title**	A study on the immune response and safety of an RSV vaccine when given to adults 18 years of age and above who received lung or kidney transplant and are at an increased risk of respiratory syncytial virus lower respiratory tract disease and compared to healthy adults 50 years of age and above
**Sponsor**	GlaxoSmithKline Biologicals S.A.<br>Rue De L'Institut 89<br>1330 Rixensart<br>Belgium
**Sponsor signatory**	Veronica Hulstrøm, MD, PhD<br>Clinical Project Lead<br>RSV Older Adults
**Qualified Person for Pharmacovigilance/Delegate**	PPD, MD, MBA<br>PPD

Medical monitor name and contact can be found in local study contact information document

©2023 GSK group of companies or its licensor.

PROTOCOL AMENDMENT 2 INVESTIGATOR AGREEMENT

I agree:

- To conduct the study in compliance with this protocol, any future protocol amendments, with the terms of the clinical trial agreement and with any other study conduct procedures and/or study conduct documents provided by GSK.
- To assume responsibility for the proper conduct of the study at this site.
- That I am aware of and will comply with GCP and all applicable regulatory requirements.
- That I will comply with the terms of the site agreement.
- To comply with local bio-safety legislation.
- To ensure that all persons assisting me with the study are adequately informed about the GSK study intervention and other study-related duties and functions as described in the protocol.
- To supervise any individual or party to whom I have delegated study-related duties and functions conducted at the study site.
- To ensure that any individual or party to whom I have delegated study-related duties and functions conducted at the study site are qualified to perform those study-related duties and functions.
- To acquire the reference ranges for laboratory tests performed locally and, if required by local regulations, obtain the laboratory's current certification or Quality Assurance procedure manual.
- To ensure that no clinical samples (including serum samples) are retained on-site or elsewhere without the approval of GSK and the express physical informed consent of the participant and/or the participant's LAR.
- To perform no biological assays on the clinical samples other than those described in the protocol or its amendment(s).
- To co-operate with representative(s) of GSK in the monitoring and data management processes of the study with respect to data entry and resolution of queries about the data.
- To have control of all essential documents and records generated under my responsibility before, during, and after the study.
- That I have been informed that certain regulatory authorities require the sponsor to obtain and supply, as necessary, details about the investigator(s)' ownership interest in the sponsor or the study intervention, and more generally about their financial ties with the sponsor. GSK will use and disclose the information solely for the purpose of complying with regulatory requirements.

Hence, I:

- Agree to supply GSK with any necessary information regarding ownership interest and financial ties (including those of my spouse and dependent children).
- Agree to promptly update this information if any relevant changes occur during the study and for 1 year following completion of the study.
- Agree that GSK may disclose any information about such ownership interests and financial ties to regulatory authorities.
- Agree to provide GSK with an updated Curriculum Vitae and all other documents required by regulatory agencies for this study.

**Study identifier**	219900 (RSV OA=ADJ-023)
**EU CT number**	2023-503951-81-00
**Approval date**	11 Oct 2023
**Title**	A Phase 2b, randomized, controlled, open-label study to evaluate the immune response and safety of the RSVPreF3 OA investigational vaccine in adults (≥18 years of age) when administered to lung and renal transplant recipients comparing 1 versus 2 doses and compared to healthy controls (≥50 years of age) receiving 1 dose
**Investigator name**	______________________________
**Signature**	______________________________
**Date of signature** (DD Month YYYY)	______________________________

PROTOCOL AMENDMENT SUMMARY OF CHANGES TABLE

DOCUMENT HISTORY	
**Document**	**Date of Issue**
Amendment 2	11 Oct 2023
Amendment 1 GER, ITA, SPN-1	04 September 2023
Original Protocol	25 April 2023

Type of Protocol Amendment	Numbering	Type of Changes
Global	Amendment 2 Final	New changes to allow the inclusion of lung or renal transplant patients as of 18 YoA and above
Country-specific	Amendment 1 GER, ITA, SPN-1 Final	New changes to address health authority feedback

Amendment 2 (11 Oct 2023)

This amendment is considered substantial based on the criteria defined in EU Clinical Trial Regulation No 536/2014 of the European Parliament and the Council of the European Union because it significantly impacts the scientific value of the study.

Overall rationale for the current Amendment:

To allow the inclusion of renal and lung solid organ transplant patients as of 18 years of age (YoA) and above. Renal and lung transplant recipient patients 18 YoA and above are at high risk of RSV-related LRTD, due to the life-long immunosuppressive therapy received for allograft rejection prevention regardless of their age [Dropulic, 2016; Fishman, 1998; Reichenberger, 2001].

Because of the safety signal of preterm birth observed in the RSV MAT-009 study, the initial RSV OA=ADJ-023 study protocol allowed only for the inclusion of participants as of 50 YoA (refer to Section 2.3.1).

Further evaluation of the preterm birth signal based on different analyses of the RSV MAT-009 study data, research on plausible biological mechanisms and recommendations by external experts led to the following conclusions:

- There is an identified risk for preterm birth in pregnant women vaccinated during the late second and third trimester with the RSV MAT vaccine candidate (refer to Section 2.3.1) and therefore pregnant women should be excluded from participation in the current study.

- The biological mechanism behind the identified risk of preterm birth in pregnant women who received the RSV MAT vaccine candidate is not known. Nevertheless, there is no theoretical mechanism to suggest a risk to future pregnancy for women vaccinated prior to pregnancy.
- Precautionary measures to mitigate the risk of exposure to the RSVPreF3 OA vaccine at any time during pregnancy, such as not enrolling pregnant individuals, pregnancy testing before each administration of the study vaccine and adequate contraception for at least 1 month before the study intervention administration until the study end are included in the inclusion criteria in this study. (Refer to Section 10.4.1 for definitions of women of childbearing potential, menopause and Section 10.4.2 on adequate contraception).

Given the high observed efficacy of the RSVPreF3 OA vaccine in clinical studies in individuals 60 YoA and older, and taking into account the precautionary measures to be implemented for this study and the unmet medical need in the renal and lung solid organ transplant patient population as of 18 YoA, the anticipated benefit-risk of the RSVPreF3 OA vaccine supports the continued clinical development in less than 50-year-old non-pregnant adults at increased risk, especially the immunocompromised (IC) population.

Since the burden of RSV-related LRTD in healthy adults 18-49 YoA is low, the age range for the RSV_HA group was not adjusted.

List of main changes in the protocol and their rationale:

Section # and title	Description of change	Brief rationale
Title page<br>1.1 Synopsis<br>1.2 Schema, Figure 1<br>2.1 Study rationale<br>4 Study design<br>5.1 Inclusion Criteria<br>6.3.2 Randomization to study intervention<br>9.5 Pre-dose sample determination	Inclusion of adults ≥18 YoA in the IC population (participants with lung or renal transplant).	To include at risk population as of 18 YoA and improve the feasibilty of recruitment of IC population.
2.1 Study rationale	Added market approval information for RSV OA vaccine	New information.
2.2 Background	Updated information on BoD of RSV in transplant patients.	Correction.
2.3 Benefit/risk assessment	Updated risk information for study vaccine in adults < 60 YoA.	Consistency update for risk information in population <60 YoA, as the age was lowered to include ≥18 YoA and above.

Section # and title	Description of change	Brief rationale
	Correction and clarifications provided for the mitigation strategy for potential risks.	
3 Objectives endpoints and estimands<br>9.3.2 Secondary endpoints/estimands analyses	Secondary immunogenicity endpoints and their estimands analyses were added.	Added based on health authority feedback from Korea.
Investigator agreement page, sections 5.1, 8.1.2, 8.1.5, 8.2, 8.4.2, 8.4.8, 10.1.3, 10.1.5, 10.3.3, 10.3.4 and 10.3.5	Inclusion of LAR in the study throughout relevant sections in the protocol. Information pertaining to informed assent for the minor added.	Wording included for assent/consent of minor participants in certain countries where legal age for consent is ≥18 YoA.
6.9 Prior and concomitant therapy Table 10	Updated the list of prohibited medication and washout period for SOT patients.	Clarification.
7.2. Participant discontinuation/withdrawal from the study	Revised text related to sample handling and its documentation upon partcipant's withdrawal from the study.	Updated to allow sample handling as per local regulations, and in response to Health authority feedback.
CCI [redacted]	CCI [redacted].	Correction.
8.4.1 Time period and frequency for collecting AE, SAE, and other safety information	Removal of study intervention from collection of study participation related safety events.	Correction.
10.3.5 Recording, assessment and follow-up of AE, SAE, AESIs and pregnancies<br>Table 24	Update in description of intensity scales for solicited events.	Clarification to site/investigator.
10.4.2 Contraception guidance<br>Table 26	Footnote for recommendation for oral contraception added.	Recommendation is suggested for population of ≥18 YoA as compared to ≥50 YoA earlier.

Section # and title	Description of change	Brief rationale
10.6 Appendix 6: Country-specific requirements<br>10.6.3 South Korea	Exclusion criteria updated.	Country-specific age requirement for participants to be 19 years of age to be included in study.
10.7 Appendix 7: Protocol amendment history	Appendix created to capture the summary of prior amendments. Information for Amendment 1 (amended based on European health authority feedback) moved to Appendix 7 and replaced with Amendment 2 (current global amendment) information.	Editorial changes to align with the Sponsor's standard protocol template.
Throughout	Updated 'RSV OA Vaccine' to 'RSVPreF3 OA vaccine'.	Consistency update in vaccine nomenclature across clinical and regulatory documents.
List of abbreviations and definition of terms	Updated the definition for Investigational product and defined concomitant medications, placebo, and standard of care.	Updates to align with GSK standards for protocol template
Throughout protocol	Administrative and editorial changes were made to update sponsor signatory on cover page, align table numbers, formatting, and crossreferences.	To maintain consistency with template guidance.

TABLE OF CONTENTS

PAGE

PROTOCOL AMENDMENT SUMMARY OF CHANGES TABLE 5

LIST OF ABBREVIATIONS AND DEFINITIONS OF TERMS 16

1. PROTOCOL SUMMARY 26
 1.1. Synopsis 26
 1.2. Schema 27
 1.3. Schedule of Activities (SoA) 28

2. INTRODUCTION 39
 2.1. Study rationale 39
 2.2. Background 40
 2.3. Benefit/risk assessment 41
  2.3.1. Risk assessment 41
  2.3.2. Benefit assessment 43
  2.3.3. Overall benefit-risk conclusion 43

3. OBJECTIVES, ENDPOINTS AND ESTIMANDS 44

4. STUDY DESIGN 45
 4.1. Overall design 45
 4.2. Scientific rationale for study design 46
  4.2.1. Participant input into design 47
 4.3. Justification for dose 47
 4.4. End of study definition 47

5. STUDY POPULATION 48
 5.1. Inclusion criteria 48
  5.1.1. Inclusion criteria for all participants 48
  5.1.2. Specific inclusion criteria for renal/lung transplant patients 49
  5.1.3. Specific inclusion criteria for renal transplant (RTx) patients 49
  5.1.4. Specific inclusion criteria for lung transplant (LTx) patients 49
  5.1.5. Specific inclusion criteria for healthy participants 49
 5.2. Exclusion criteria 50
  5.2.1. Medical conditions 50
  5.2.2. Prior/Concomitant therapy 51
  5.2.3. Prior/Concurrent clinical study experience 51
  5.2.4. Other exclusion criteria 51
   5.2.4.1. Specific exclusion criteria for renal/lung transplant patients: 52
   5.2.4.2. Specific exclusion criteria for RTx patients: 53
   5.2.4.3. Specific exclusion criteria for LTx patients: 53
   5.2.4.4. Specific exclusion criteria for healthy participants: 53
 5.3. Lifestyle considerations 53
 5.4. Screen failures 54
 5.5. Criteria for temporarily delaying enrollment/ administration of study intervention 54

6. STUDY INTERVENTION AND CONCOMITANT THERAPY ...55
6.1. Study intervention administered ...55
6.1.1. Medical devices ...55
6.2. Preparation, handling, storage, and accountability ...56
6.3. Assignment to study intervention ...56
6.3.1. Participant identification ...56
6.3.2. Randomization to study intervention ...56
6.3.3. Intervention allocation to the participant ...57
6.3.4. Allocation of participants to CMI assay subsets ...57
6.4. Blinding ...58
6.5. Study intervention compliance ...58
6.6. Dose modification ...58
6.7. Continued access to study intervention after the end of the study ...58
6.8. Treatment of overdose ...58
6.9. Prior and concomitant therapy ...58

7. DISCONTINUATION OF STUDY INTERVENTION AND PARTICIPANT DISCONTINUATION/WITHDRAWAL ...60
7.1. Discontinuation of study intervention ...60
7.1.1. Contraindications to subsequent study intervention administration ...61
7.2. Participant discontinuation/withdrawal from the study ...62
7.3. Lost to follow-up ...63

8. STUDY ASSESSMENTS AND PROCEDURES ...63
8.1. Administrative and baseline procedures ...65
8.1.1. Collection of demographic data ...65
8.1.2. Medical/vaccination history ...65
8.1.3. Record specific participant transplant information ...66
8.1.4. Record height and weight ...66
8.1.5. Targeted physical examination/history-directed physical examination ...67
8.1.6. Vital signs ...67
8.1.7. Allograft rejection ...68
8.1.7.1. Record estimated glomerular filtration rate (eGFR) and proteinuria/albuminuria (only in RTx patients) ...68
8.1.7.2. Record bronchoscopy and/or transbronchial lung biopsy (TBBx) (only in LTx patients) ...69
8.1.7.3. Record FEV1 (only in LTx patients) ...69
8.1.7.4. Imaging results (RTx and LTx patients) ...69
8.1.7.5. Biopsy results (RTx and LTx patients) ...70
8.2. Immunogenicity assessments ...70
8.2.1. Biological samples ...70
8.2.2. Laboratory assays ...71
8.2.3. Immunological read-outs ...72
8.2.4. Cytology ...73
8.2.5. Immunological correlates of protection ...73
8.3. Safety assessments ...73
8.3.1. Clinical safety laboratory tests ...73
CCI [redacted]
8.3.3. Pregnancy testing ...74

8.3.4. Safety monitoring and Committee 74
8.4. Adverse events (AEs), serious adverse events (SAEs), and other safety reporting 74
8.4.1. Time period and frequency for collecting AE, SAE, and other safety information 75
8.4.2. Method of detecting AEs and SAEs 78
8.4.3. Follow-up of AEs and SAEs 78
8.4.4. AESIs 78
8.4.4.1. Potential immune-mediated diseases 78
8.4.4.2. Atrial fibrillation (AF) 83
8.4.4.3. AESI for Transplant patients 84
8.4.5. Regulatory reporting requirements for SAEs/pregnancies/AESIs 84
8.4.6. Pregnancy 85
8.4.7. Contact information for reporting SAEs, AESIs and pregnancies 86
8.4.8. Participant card 86
8.5. Pharmacokinetics 86
8.6. Pharmacodynamics 86
8.7. Genetics 86
8.8. Biomarkers 86
8.9. Immunogenicity assessments 86
8.10. Health economics or medical resource utilization and health economics 86

9. STATISTICAL CONSIDERATIONS 87
9.1. Statistical Hypotheses 87
9.2. Analysis sets 87
9.2.1. Criteria for elimination from analysis 87
9.3. Statistical analyses 88
9.3.1. Primary endpoints/estimands analyses 88
9.3.2. Secondary endpoints/estimands analyses 88
9.4. Interim analysis 90
9.4.1. Sequence of analyses 90
9.4.2. Statistical considerations for interim analysis 90
9.5. Pre-dose sample size determination 90
9.5.1. Sample size justification 91

10. SUPPORTING DOCUMENTATION AND OPERATIONAL CONSIDERATIONS 92
10.1. Appendix 1: Regulatory, ethical, and study oversight considerations 92
10.1.1. Regulatory and ethical considerations 92
10.1.2. Financial disclosure 93
10.1.3. Informed consent process 93
10.1.4. Recruitment strategy 94
10.1.5. Data protection 94
10.1.6. Committees structure 95
10.1.7. Dissemination of Clinical Study Data 95
10.1.8. Data quality assurance 96
10.1.9. Source documents 97
10.1.10. Study and site start and closure 97
10.1.11. Publication policy 98

10.2. Appendix 2: Clinical laboratory tests ... 98
10.2.1. RSV-A and RSV-B neutralization assays ... 98
10.2.2. Intracellular cytokine staining ... 99
10.3. Appendix 3: AEs and SAEs: Definitions and procedures for recording, evaluating, follow-up, and reporting ... 100
10.3.1. Definition of AE ... 100
10.3.2. Definition of SAE ... 101
10.3.3. Solicited events ... 102
10.3.4. Unsolicited AE ... 103
10.3.5. Recording, assessment and follow-up of AE, SAE, AESIs and pregnancies ... 103
10.3.5.1. AE and SAE recording ... 103
10.3.5.2. Assessment of intensity ... 104
10.3.5.3. Assessment of causality ... 106
10.3.5.4. Assessment of outcomes ... 107
10.3.5.5. Follow-up of AEs, SAEs, AESIs and pregnancies ... 107
10.3.5.6. Updating of SAE, AESI and pregnancy information after removal of write access to the participant's eCRF ... 108
10.3.5.7. Reporting of SAEs, AESIs and pregnancies ... 108
10.4. Appendix 4: Guidance on Contraception, Women not considered as WOCBP and collection of pregnancy information ... 109
10.4.1. Definitions ... 109
10.4.1.1. Woman of childbearing potential (WOCBP) ... 109
10.4.1.2. Woman of Nonchildbearing potential (WONCBP) ... 109
10.4.2. Contraception guidance ... 110
10.4.2.1. Woman of childbearing potential (WOCBP) ... 110
10.5. Appendix 5: Grading scales in scope of the SOT population ... 111
10.5.1. Chronic kidney disease (CKD) classification in RTx patients ... 111
10.5.1.1. Revised equations for eGFR from serum creatinine in Japan ... 111
10.5.2. Proteinuria/albuminuria categories in CKD in RTx patients ... 112
10.5.3. Staging of bronchoscopy – acute cellular rejection (ACR) in LTx patients ... 113
10.5.4. Grading of bronchoscopy – antibody-mediated rejection (AMR) in LTx patients ... 113
10.5.5. CLAD staging in LTx patients ... 113
10.6. Appendix 6: Country-specific requirements ... 113
10.6.1. Germany ... 113
10.6.2. Japan ... 116
10.6.3. South Korea ... 117
10.7. Appendix 7: Protocol amendment history ... 117

11. REFERENCES ... 121

LIST OF TABLES

PAGE

Table 1 Schedule of Activities for the IC 1-dose group (RSV_IC_1) 28
Table 2 Schedule of Activities for the IC 2-dose group (RSV_IC_2) 32
Table 3 Schedule of Activities for the healthy participants group (RSV_HA) 36
Table 4 Intervals between study visits for IC 1-dose group (RSV_IC_1) 39
Table 5 Intervals between study visits for IC 2-dose group (RSV_IC_2) 39
Table 6 Intervals between study visits for healthy participant group (RSV_HA) 39
Table 7 Objectives, endpoints and estimands 44
Table 8 Study Intervention Administered 55
Table 9 Allocation to CMI subset 57
Table 10 Prohibited medications and washout period 59
Table 11 Timing of collection of concomitant medication to be recorded 60
Table 12 Biological samples 70
Table 13 Laboratory assays 71
Table 14 Immunological read-outs 72
Table 15 Timeframes for collecting and reporting of safety information in IC 1-dose group and healthy participant group 76
Table 16 Timeframes for collecting and reporting of safety information in IC 2-dose group 77
Table 17 List of potential immune-mediated diseases (pIMDs) 79
Table 18 Timeframes for submitting SAEs, pregnancies and AESIs to GSK 85
Table 19 Contact information for reporting SAEs, AESIs and pregnancies 86
Table 20 Analysis sets 87
Table 21 Estimate of the fold-increase that can be detected by power 91
Table 22 Solicited administration site events 102
Table 23 Solicited systemic events 102

Table 24 Intensity scales for solicited events in participants ≥18 YoA (IC group) and ≥50 YoA (HA group) 105

Table 25 Intensity scales of administration site redness/swelling, and fever 105

Table 26 Highly effective contraceptive methods 110

Table 27 Proteinuria/albuminuria categories in CKD 112

Table 28 Relationship among categories for albuminuria and proteinuria 112

Table 29 Classification of ACR 113

Table 30 Classification of AMR 113

Table 31 Stages of CLAD 113

LIST OF FIGURES

PAGE

Figure 1 Study design overview 27

LIST OF ABBREVIATIONS AND DEFINITIONS OF TERMS

List of Abbreviations

Abbreviation	Definition
ACR	Acute cellular rejection
AE	Adverse event
AESI	Adverse event of special interest
AF	Atrial fibrillation
ALT	Alanine aminotransferase
AMR	Antibody-mediated rejection
ANCOVA	Analysis of covariance
AST	Aspartate aminotransferase
BAL	Bronchoalveolar lavage
BoD	Burden of disease
CCI	Commercially confidential information
CD	Cluster of differentiation
CI	Confidence interval
CIOMS	Council for International Organizations of Medical Sciences
CKD	Chronic kidney disease
CLAD	Chronic lung allograft dysfunction
CLS	Clinical laboratory sciences
CMI	Cell-mediated immunity
CMV	Cytomegalovirus
CONSORT	Consolidated Standards of Reporting Trials
COPD	Chronic obstructive pulmonary disease
COVID-19	Coronavirus disease 2019

Abbreviation	Definition
CRA	Clinical research associate
CRO	Contract research organization
CSR	Clinical study report
DFP	Direct-from-participant
DRC	Data review committee
CCI [redacted]	
DTP	Direct-to-participant
ECG	Electrocardiogram
eCRF	Electronic case report form
ED	Early discontinuation
eGFR	Estimated glomerular filtration rate
EMA	European Medicines Agency
EoS	End of study
ES	Exposed set
EU	European Union
EUA	Emergency use authorization
FAS	Full analysis set
FEV1	Forced expiratory volume in 1 second
FSFV	First subject first visit
FSH	Follicle stimulating hormone
FVC	Forced vital capacity
GCP	Good clinical practices
GFR	Glomerular filtration rate
GMT	Geometric mean titer

Abbreviation	Definition
GSK	GlaxoSmithKline Biologicals S.A.
HHS	Home healthcare services
HI	Humoral immunity
CCI [redacted]	
HLT	High Level Term
HRT	Hormonal replacement therapy
IAF	Informed assent form
IB	Investigator’s brochure
IC	Immunocompromised
ICF	Informed consent form
ICH	International Council on Harmonisation
ICMJE	International Committee of Medical Journal Editors
ICS	Intracellular cytokine staining
IDMC	Independent data monitoring committee
IEC	Independent ethics committee
IFN	Interferon
IgA	Immunoglobulin A
IL	Interleukin
IM	Intramuscular
IMP	Investigational medicinal product
IRB	Institutional review board
ISHLT	International Society for Heart and Lung Transplantation
IUD	Intrauterine device
IUS	Intrauterine hormone-releasing system

Abbreviation	Definition
LAR	Legally acceptable representative
LRTD	Lower respiratory tract disease
LSLV	Last Subject Last Visit
LTx	Lung translplant
MDRD	Modification of Diet in Renal Disease
MedDRA	Medical Dictionary for Regulatory Activities
MGI	Mean geometric increase
CCI [redacted]	
OA	Older adults
PBMC	Peripheral blood mononuclear cells
PD	Pharmacodynamic
pDiary	Paper diary
pIMD	Potential immune-mediated disease
PK	Pharmacokinetic
PPS	Per protocol set
PRB	Protocol review board
PT	Preferred term
PTB	Preterm birth
QS-21:	*Quillaja saponaria* Molina, fraction 21 (Licensed by GSK from Antigenics LLC, a wholly owned subsidiary of Agenus Inc., a Delaware, USA corporation)
QTL	Quality tolerance limit
RNA	Ribonucleic acid
rSDV	Remote source data verification
RSV	Respiratory Syncitial Virus

Abbreviation	Definition
RSV-LRTD	RSV-lower respiratory tract disease
RTI	Respiratory tract infections
RTSM	Randomization and Trial Supply Management
RTx	Renal transplant
RZV	Recombinant Zoster vaccine
SAE	Serious adverse event
SAP	Statistical analysis plan
SAR	Serious adverse reaction
SDV	Source data verification
SmPC	Summary of product characteristics
SoA	Schedule of activities
SoC	Standard of care
SOC	System organ class
SOT	Solid organ transplant
SRT	Safety Review Team
SUSAR	Suspected unexpected serious adverse reaction
TLC	Total lung capacity
TNF	Tumor necrosis factor
TOC	Table of contents
Tx	Transplant
UK	United Kingdom
US/USA	United States of America
WBC	White blood cell
WOCBP	Woman of childbearing potential

Abbreviation	Definition
WONCBP	Woman of nonchildbearing potential
YoA	Years of age

Definition of Terms

Term	Definition
Adverse event	Any untoward medical occurrence in a patient or clinical investigation participant, temporally associated with the use of a medicinal product, whether or not considered related to the medicinal product.<br>An AE can therefore be any unfavorable and unintended sign (including an abnormal laboratory finding), symptom, or disease (new or exacerbated) temporally associated with the use of a medicinal product. For marketed medicinal products, this also includes failure to produce expected benefits (i.e., lack of efficacy), abuse or misuse.
Adverse event of special interest	An adverse event of special interest (serious or non-serious) is one of scientific and medical concern specific to the sponsor's product or program, for which ongoing monitoring and rapid communication by the investigator to the sponsor can be appropriate. Such an event might warrant further investigation in order to characterize and understand it. Depending on the nature of the event, rapid communication by the trial sponsor to other parties (e.g., regulators) might also be warranted.
Blinding	A procedure in which 1 or more parties to the study are kept unaware of the intervention assignment in order to reduce the risk of biased study outcomes. The level of blinding is maintained throughout the conduct of the study, and only when the data are cleaned to an acceptable level of quality will appropriate personnel be unblinded or when required in case of a SAE.<br>In an open-label study, no blind is used. Both the investigator and the participant know the identity of the intervention assigned.
Caregiver	A 'caregiver' is someone who<br>• lives in the close surroundings of a participant and has a continuous caring role or<br>• has substantial periods of contact with a participant and is engaged in their daily health care (e.g., a relative of the participant, a nurse who helps with daily activities in case of residence in a nursing home).

Term	Definition
	In the context of a clinical study, a caregiver could include an individual appointed to oversee and support the participant's compliance with protocol-specified procedures(such as transcribing responses to diaries, receiving phone calls, planning study visits, etc.). However, at no time, the caregiver should evaluate the participant's health status while answering diaries or make decisions on behalf of the participant.
Certified copy	A copy (irrespective of the type of media used) of the original record that has been verified (i.e., by a dated signature or by generation through a validated process) to have the same information, including data that describe the context, content, and structure, as the original.
Co-administered (concomitant) products	A product given to clinical trial participants as required in the protocol as part of their standard care for a condition which is not the indication for which the IMP is being tested and is therefore not part of the objective of the study.
Direct-from-Participant Shipments	Home pickup of collected biological specimens, or pickup and return of unused/partially used/expired trial materials for return to investigator site.
Direct-to-Participant Shipments	Shipping of lab kits, devices, etc., to the participant's residence under secure and controlled conditions.
Eligible	Qualified for enrollment into the study based upon strict adherence to inclusion/exclusion criteria.
Enrolled participant	All participants who entered the study (who were randomized or received study intervention administration or underwent a post-screening study procedure).<br><br>NOTE: screening failures (who never passed screening even if rescreened) and participants screened (met eligibility) but never enrolled into the study are excluded from the Enrolled Set as they did not enter the study.<br><br>Refer to the Section 9.2 for the definition of 'Enrolled Set' applicable to the study.
Essential documents	Documents which individually and collectively permit evaluation of the conduct of a study and the quality of the data produced.
Evaluable	Meeting all eligibility criteria, complying with the procedures defined in the protocol, and, therefore, included in the per protocol analysis.
Home Healthcare Services	Deployment of mobile health care professional(s) (nurses or phlebotomists) to perform study activities remotely.

Term	Definition
Intercurrent medical condition	A condition that has the capability of altering the immune response to the study vaccine or is confirmed to have an alteration of the participant's initial immune status.
Intervention number	A number identifying an intervention to a participant, according to intervention allocation.
Invasive medical device	A device which, in whole or in part, penetrates inside the body, either through a body orifice or through the surface of the body.
Investigational product	A pharmaceutical form of an active substance or placebo being tested or used as a reference in a clinical trial, including products already with a marketing authorization but used or assembled (formulated or packaged) in a way different from the authorised form, or when used for an unauthorised indication, or when used to gain further information about the authorised form.
Investigator	A person responsible for the conduct of the clinical study at a study site. If a study is conducted by a team of individuals at a study site, the investigator is the responsible leader of the team and may be called the principal investigator.<br>The investigator can delegate study-related duties and functions conducted at the study site to qualified individual or party to perform those study-related duties and functions.
Participant	Term used throughout the protocol to denote an individual who has been contacted to participate or who participates in the clinical study as a recipient of the study intervention (vaccine(s)/product(s)/control).<br>Synonym: subject.
Participant number	A unique identification number assigned to each participant who consents to participate in the study.
Placebo	An inactive substance or treatment that looks the same as, and is given in the same way as, an active drug or intervention/treatment being studied.
Primary Completion Date	The date on which the last participant in a clinical study was examined or received an intervention to collect final data for the primary outcome measure.<br>Whether the clinical study ended according to the protocol or was terminated does not affect this date. For clinical studies with more than one primary outcome measure with different completion dates, this term refers to the date on which data collection is completed for all the primary outcome measures.

Term	Definition
Protocol amendment	The ICH defines a protocol amendment as: 'A written description of a change(s) to or formal clarification of a protocol.' GSK further details this to include a change to an approved protocol that affects the safety of participants, scope of the investigation, study design, or scientific integrity of the study.
Randomization	Process of random attribution of intervention to participants to reduce selection bias.
Screened participant	All participants who were screened for eligibility.
Self-contained study	Study with objectives not linked to the data of another study.
Serious Adverse Reaction	All noxious and unintended responses to an IMP related to any dose administered that result in death, are life-threatening, require patient hospitalization or prolongation of existing hospitalization, result in persistent or significant disability or incapacity, or are a congenital anomaly or birth defect.
Solicited event	Events to be recorded as endpoints in the clinical study. The presence/occurrence/intensity of these events is actively solicited from the participant or an observer during a specified follow-up period following study intervention administration.
Source data	All information in original records and certified copies of original records of clinical findings, observations, or other activities in a clinical study necessary for the reconstruction and evaluation of the study. Source data are contained in source documents (original records or certified copies).
Source documents	Original legible documents, data, and records (e.g., hospital records, clinical and office charts, laboratory notes, memoranda, participants' diaries or evaluation checklists, pharmacy dispensing records, recorded data from automated instruments, copies or transcriptions certified after verification as being accurate copies, microfiches, photographic negatives, microfilm or magnetic media, x-rays, participant files, and records kept at the pharmacy, laboratories and at medico-technical departments involved in the clinical study).
Standard of Care	Medicine(s) for a specific indication, or a component of the standard care for a particular medical indication, based on national and/or international consensus; there is no regulatory significance to this term.<br><br>Products/regimens considered standard of care may differ country to country, depending on consensus in individual countries.

Term	Definition
Study completion date	The date on which the last participant in a clinical study was examined or received an intervention/treatment to collect final data for the primary outcome measures, secondary outcome measures, and AEs (that is, the last participant's last visit or LSLV).
Study intervention	Term used throughout the clinical study to denote a set of investigational product(s) or marketed product(s) or placebo intended to be administered to a participant.
Study monitor	An individual assigned by the sponsor and responsible for assuring proper conduct of clinical studies at 1 or more investigational sites.
Sub-cohort	A group of participants for whom specific study procedures are planned as compared to other participants or a group of participants who share a common characteristic (e.g., ages, vaccination schedule, etc.) at the time of enrollment.<br>Synonym: subset.
Suspected Unexpected Serious Adverse Reaction	A Suspected Unexpected Serious Adverse Reaction is a Serious Adverse Reaction whose nature, severity or outcome is not consistent with the reference safety information.
Unsolicited adverse event	Any AE reported in addition to those solicited during the clinical study. Also, any ‘solicited’ symptom with onset outside the specified period of follow-up for solicited symptoms will be reported as an unsolicited adverse event.

1. PROTOCOL SUMMARY

1.1. Synopsis

Protocol Title: A Phase 2b, randomized, controlled, open-label study to evaluate the immune response and safety of the RSVPreF3 OA investigational vaccine in adults (≥18 years of age) when administered to lung and renal transplant recipients comparing 1 versus 2 doses and compared to healthy controls (≥50 years of age) receiving 1 dose.

Brief Title: A study on the immune response and safety of an RSV vaccine when given to adults 18 years of age and above who received a lung or kidney transplant and are at an increased risk of respiratory syncytial virus lower respiratory tract disease and compared to healthy adults 50 years of age and above.

Rationale: Refer to Section 2.1.

Objectives, Endpoints, and Estimands: Refer to Section 3.

Overall Design: Refer to Sections 1.2 and 4.1.

Number of Participants: Refer to Section 9.5.

Data Monitoring/Other Committee: Refer to Section 10.1.6.

1.2. Schema

Figure 1 Study design overview

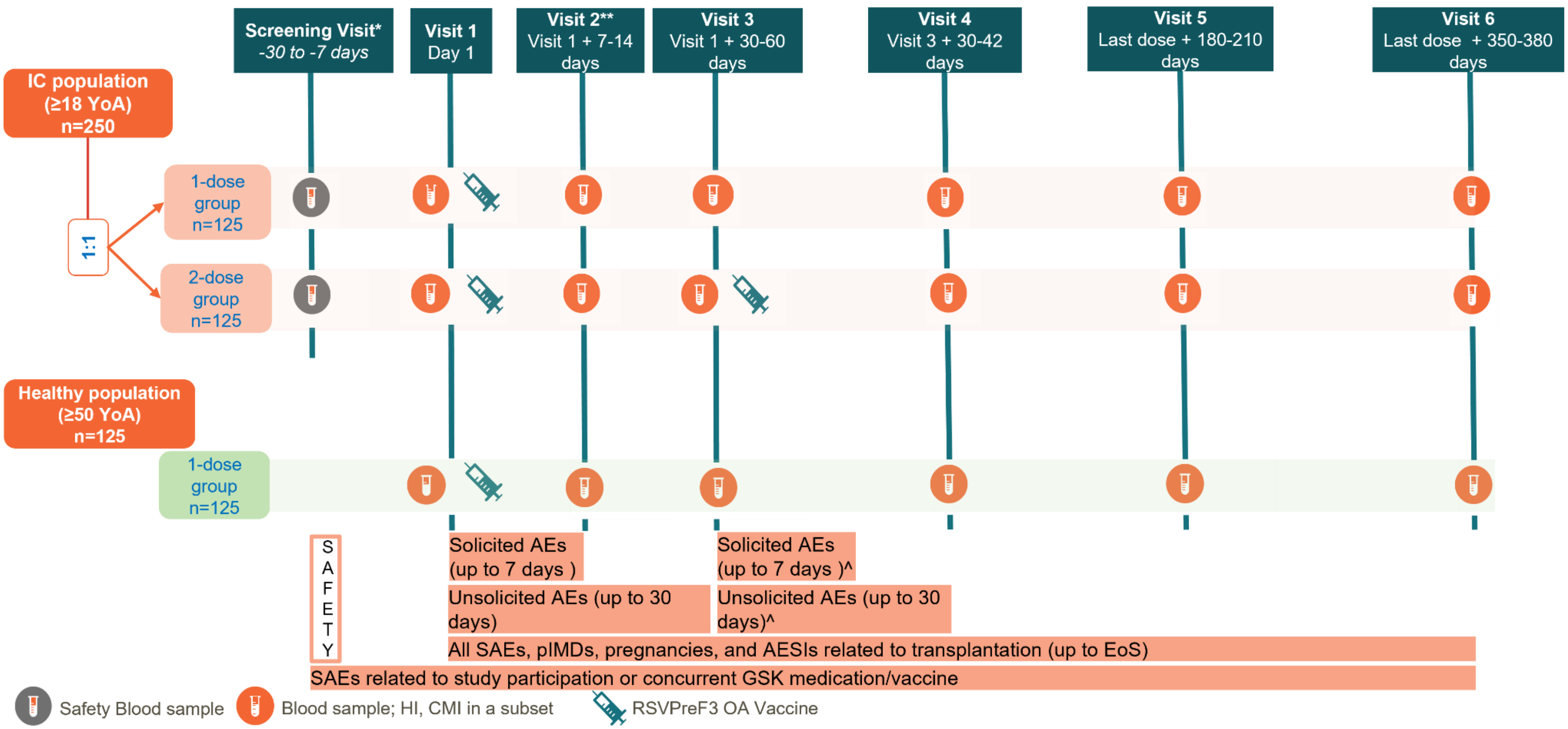

*Screening Visit: Only in IC population. Blood sample would be collected at screening visit to evaluate participant eligibility.

**Visit 2: Only in a subset of the study population, ~30% of the participants (Visit can occur 7-14 days after first study intervention administration).

^ Solicited and unsolicited AEs after second study intervention administration, only in the IC 2-dose group.

AE = Adverse event; AESI = Adverse event of special interest; CMI = Cell-mediated immunity; EoS = End of study; HI = Humoral immunity; IC = Immunocompromised; pIMD = Potential immune-mediated disease; SAE = Serious adverse event; YoA = Years of age.

1.3. Schedule of Activities (SoA)

Table 1 Schedule of Activities for the IC 1-dose group (RSV_IC_1)

Type of contact	Screening Visit	Visit 1	Visit 2[a]	Visit 3	Visit 4	Visit 5	Visit 6	Notes
Time points	-30 to -7 days	Day 1	Visit 1 + 7-14 days	Visit 1 + 30-60 days	Visit 3 + 30-42 days	Visit 1 + 180-210 days	Visit 1 + 350-380 days	
**Baseline and demography assessments**								
Informed consent	●							See Section 10.1.3 for details
Assignment/recording of participant number	●							See Section 6.3.1 for details
Check inclusion and exclusion criteria	●	●						See Sections 5.1 and 5.2 for details
Check with participants if he/she will appoint a caregiver, and distribute caregiver information letter to caregiver, when applicable	○	○	○	○	○	○	○	See Section 8 for details
Collect demographic data	●							See Section 8.1.1 for details
Record relevant medical history	●							See Section 8.1.2 for details
Record vaccination history	●							See Section 8.1.2 for details
Record specific transplant information	●							See Section 8.1.3 for details
Record height and weight	●							See Section 8.1.4 for details
History directed physical examination	○							See Section 8.1.5 for details
Record vital signs		●[b]						See Section 8.1.6 for details
Screening conclusion	●							
**Laboratory assessments**								
Safety blood sample (Hematology & Biochemistry for liver and renal function; ~ 6 mL)	●							See Section 8.3.1 for details
Urine pregnancy test (if applicable, WOCBP only)	●	●[b]						See Section 8.3.3 for details
Blood sampling (~ 8.5 mL) for RSV neutralizing titers		●[b]	●	●	●	●	●	See Section 8.2 for details

Type of contact	Screening Visit	Visit 1	Visit 2[a]	Visit 3	Visit 4	Visit 5	Visit 6	Notes
**Time points**	**-30 to -7 days**	**Day 1**	**Visit 1 + 7-14 days**	**Visit 1 + 30-60 days**	**Visit 3 + 30-42 days**	**Visit 1 + 180-210 days**	**Visit 1 + 350-380 days**	
Blood sampling (~ 30 mL) for CMI response (in CMI subset only)		●[b]	●	●	●	●	●	See Section 8.2 for details
CCI [redacted]								
**Study intervention administration**								
Distribution of 'Participant card'		○						See Section 8.4.8 for details
Check contraindications, warnings, and precautions to vaccination		○						See Section 7.1.1 for details
Check criteria for temporary delay for enrollment and study intervention administration		○						See Section 5.5 for details
Body temperature before study intervention administration		●						The location for measuring temperature will be oral or axillary (See Section 8.1.6). Fever is defined as temperature ≥38.0°C/100.4°F, regardless of the location of measurement.
Randomization, study group and intervention number allocation		○						See Section 6.3 for details
Study intervention administration (including 30-minutes post-study intervention administration observation)		●						See Section 6.1 for details
Recording of administered study intervention number		●						See Section 6.3.3 for details
**Safety assessments**								
Provide post-vaccination diary cards		○						See Section 10.3.5.1 for details
Train participants on completion of diary cards		○						See Section 10.3.5.1 for details

Type of contact	Screening Visit	Visit 1	Visit 2[a]	Visit 3	Visit 4	Visit 5	Visit 6	Notes
Time points	-30 to -7 days	Day 1	Visit 1 + 7-14 days	Visit 1 + 30-60 days	Visit 3 + 30-42 days	Visit 1 + 180-210 days	Visit 1 + 350-380 days	
Return of post-vaccination diary cards			○[c]	○				See Section 10.3.5.1 for details
Verify and transcribe post-vaccination diary cards				●				See Section 10.3.5.1 for details
Record SoC results of eGFR and proteinuria/albuminuria in RTx patients [d]	●	●	●	●	●	●	●	See Section 8.1.7.1 for details
Record SoC results of bronchoscopy and/or TBBx in LTx patients, if available	●	●	●	●	●	●	●	See Section 8.1.7.2 for details
Record SoC results of FEV1 in LTx patients, if available [e]	●	●	●	●	●	●	●	See Section 8.1.7.3 for details
Record SoC results of imaging in RTx and LTx patients, if available	●	●	●	●	●	●	●	See Section 8.1.7.4 for details
Targeted physical exam (as needed, SoC)		○	○	○	○	○	○	See Section 8.1.5 for details
Record concomitant medication/vaccination	●	●	●	●	●	●	●	See Section 6.9 for details
Record solicited administration site and systemic events (within 7 days post-study intervention administration)		●	●	●				See Section 10.3.5 for details
Record unsolicited AEs (within 30 days post-study intervention administration) [f]		●	●	●				See Section 10.3.5 for details
Record SAEs related to study participation or concurrent GSK medication/vaccine	●	●	●	●	●	●	●	See Section 10.3.5 for details

Type of contact	Screening Visit	Visit 1	Visit 2[a]	Visit 3	Visit 4	Visit 5	Visit 6	Notes
Time points	-30 to -7 days	Day 1	Visit 1 + 7-14 days	Visit 1 + 30-60 days	Visit 3 + 30-42 days	Visit 1 + 180-210 days	Visit 1 + 350-380 days	
Record:<br>- All SAEs [f]<br>- All pIMDs<br>- Pregnancies<br>- AESIs specific for renal/lung transplant<br>- (S)AEs leading to withdrawal from the study<br>- Intercurrent medical conditions		●	●	●	●	●	●	See Section 10.3.5 for details
Study conclusion							●	See Section 4.4 for details

Note: The double-line borders indicate the analyses which will be performed on all data (i.e., data that are as clean as possible) obtained up to these timepoints.

AE = Adverse event; AESI = Adverse event of specific interest; AF = Atrial fibrillation; CMI = Cell-mediated immunity; eCRF = electronic case report form; eGFR = estimated glomerular filtration rate; FEV1 = forced expiratory volume in 1 second; LTx = Lung transplant; CCI [redacted]; pIMDs = Potential immune-mediated diseases; RSV = Respiratory Syncytial Virus; RTx = Renal transplant; SAE = Serious adverse event; SoC = Standard of care; TBBx = Transbronchial Bioscopy; WOCBP = Women of childbearing potential.

● is used to indicate a study procedure that requires documentation in the individual eCRF.

○ is used to indicate a study procedure that does not require documentation in the individual eCRF.

[a] Visit 2 is planned in the CMI subset of the study population (30% of the group population i.e., 38 participants in the group, at the designated CMI sites).

[b] Vital signs, urine sample for pregnancy test and blood sampling for RSV neutralizing titers, CMI and CCI [redacted] must be taken on the same day, prior to study intervention administration. If the study intervention administration is delayed by any reason, the vital signs and samples (both blood and urine) will need to be collected again on the day of study intervention administration, urine pregnancy test needs to be repeated, and the first blood sample will be destroyed.

[c] Participants must bring their diary cards at this visit for review and the same would be re-dispensed to them.

[d] eGFR measurements are performed in RTx patients at the central lab at screening visit. At subsequent visits, it is recorded if performed as SoC. Proteinuria/albuminuria is recorded, if performed as SoC in RTx patients.

[e] FEV1 results recorded only at health facility (hospital, clinic, site) will be used.

[f] AF will be considered as AESI in this study and will be additionally reported in the AF follow-up questionnaire (electronic or paper) in eCRF. The collection of AF will be performed following the AE/SAE reporting periods. The reporting of non-serious AF will be performed according to the unsolicited AE reporting period. The reporting of AF meeting the SAE definition (serious AF) will be performed according to the SAE reporting period.

Under special circumstances, home visits may be conducted at the discretion of the investigator (Refer to Section 8 for details).

Table 2 Schedule of Activities for the IC 2-dose group (RSV_IC_2)

Type of contact	Screening Visit	Visit 1	Visit 2[a]	Visit 3	Visit 4	Visit 5	Visit 6	Notes
**Time points**	**-30 to -7 days**	**Day 1**	**Visit 1 + 7-14 days**	**Visit 1 + 30-60 days**	**Visit 3 + 30-42 days**	**Visit 3 + 180-210 days**	**Visit 3 + 350-380 days**	
**Baseline and demography assessments**								
Informed consent	●							See Section 10.1.3 for details
Assignment/recording of participant number	●							See Section 6.3.1 for details
Check inclusion and exclusion criteria	●	●						See Sections 5.1 and 5.2 for details
Check with participants if he/she will appoint a caregiver, and distribute caregiver information letter to caregiver, when applicable	○	○	○	○	○	○	○	See Section 8 for details
Collect demographic data	●							See Section 8.1.1 for details
Record relevant medical history	●							See Section 8.1.2 for details
Record vaccination history	●							See Section 8.1.2 for details
Record specific transplant information	●							See Section 8.1.3 for details
Record height and weight	●							See Section 8.1.4 for details
History directed physical examination	○							See Section 8.1.5 for details
Record vital signs		●[b]		●[b]				See Section 8.1.6 for details
Screening conclusion	●							
**Laboratory assessments**								
Safety blood sample (Hematology & Biochemistry for liver and renal function; ~6 mL)	●							See Section 8.3.1 for details
Urine pregnancy test (if applicable, WOCBP only)	●	●[b]		●[b]				See Section 8.3.3 for details
Blood sampling (~ 8.5 mL) for RSV neutralizing titers		●[b]	●	●[b]	●	●	●	See Section 8.2 for details
Blood sampling (~ 30 mL) for CMI response (in CMI subset only)		●[b]	●	●[b]	●	●	●	See Section 8.2 for details
CCI [redacted]								

Type of contact	Screening Visit	Visit 1	Visit 2[a]	Visit 3	Visit 4	Visit 5	Visit 6	Notes
**Time points**	**-30 to -7 days**	**Day 1**	**Visit 1 + 7-14 days**	**Visit 1 + 30-60 days**	**Visit 3 + 30-42 days**	**Visit 3 + 180-210 days**	**Visit 3 + 350-380 days**	
**Study intervention administration**								
Distribution of 'Participant card'		○						See Section 8.4.8 for details
Check contraindications, warnings, and precautions to vaccination		○		○				See Section 7.1.1 for details
Check criteria for temporary delay for enrollment		○						See Section 5.5 for details
Check criteria for temporary delay of study intervention administration		○		○				See Section 5.5 for details
Body temperature before study intervention administration		●		●				The location for measuring temperature will be oral or axillary (See Section 8.1.6). Fever is defined as temperature ≥38.0°C/100.4°F, regardless of the location of measurement.
Randomization, study group allocation		○						See Section 6.3 for details
Intervention number allocation		○		○				See Section 6.3 for details
Study intervention administration (including 30-minutes post-study intervention administration observation)		●		●				See Section 6.1 for details
Recording of administered study intervention number		●		●				See Section 6.3.3 for details
**Safety assessments**								
Provide post-vaccination diary cards		○		○				See Section 10.3.5.1 for details
Train participants on completion of diary cards		○						See Section 10.3.5.1 for details
Return of post-vaccination diary cards			○[c]	○	○			See Section 10.3.5.1 for details
Verify and transcribe post-vaccination diary cards				●	●			See Section 10.3.5.1 for details

Type of contact	Screening Visit	Visit 1	Visit 2[a]	Visit 3	Visit 4	Visit 5	Visit 6	Notes
Time points	-30 to -7 days	Day 1	Visit 1 + 7-14 days	Visit 1 + 30-60 days	Visit 3 + 30-42 days	Visit 3 + 180-210 days	Visit 3 + 350-380 days	
Record SoC results of eGFR and proteinuria/albuminuria in RTx patients [d]	●	●	●	●	●	●	●	See Section 8.1.7.1 for details
Record SoC results of bronchoscopy and/or TBBx in LTx patients, if available	●	●	●	●	●	●	●	See Section 8.1.7.2 for details
Record SoC results of FEV1 in LTx patients, if available [e]	●	●	●	●	●	●	●	See Section 8.1.7.3 for details
Record SoC results of imaging in RTx and LTx patients, if available	●	●	●	●	●	●	●	See Section 8.1.7.4 for details
Targeted physical exam (as needed, SoC)		○	○	○	○	○	○	See Section 8.1.5 for details
Record concomitant medication/vaccination	●	●	●	●	●	●	●	See Section 6.9 for details
Record solicited administration site and systemic events (within 7 days post-study intervention administration)		●	●	●	●			See Section 10.3.5 for details
Record unsolicited AEs (within 30 days post-study intervention administration) [f]		●	●	●	●			See Section 10.3.5 for details
Record SAEs related to study participation or concurrent GSK medication/vaccine	●	●	●	●	●	●	●	See Section 10.3.5 for details
Record:<br>- All SAEs [f]<br>- All pIMDs<br>- Pregnancies<br>- AESIs specific for renal/lung transplant<br>- (S)AEs leading to withdrawal from the study<br>- Intercurrent medical conditions		●	●	●	●	●	●	See Section 10.3.5 for details
Study conclusion							●	See Section 4.4 for details

Note: The double-line borders indicate the analyses which will be performed on all data (i.e., data that are as clean as possible) obtained up to these timepoints.

AE = Adverse event; AESI = Adverse event of specific interest; AF = Atrial fibrillation; CMI = Cell-mediated immunity; eCRF = electronic case report form; eGFR = estimated glomerular filtration rate; FEV1 = forced expiratory volume in 1 second; LTx = Lung transplant; [redacted]; pIMDs = Potential immune-mediated diseases; RSV = Respiratory Syncytial Virus; RTx = Renal transplant; SAE = Serious adverse event; SoC = Standard of care; TBBx = Transbronchial Bioscopy; WOCBP = Women of childbearing potential.

● is used to indicate a study procedure that requires documentation in the individual eCRF.

○ is used to indicate a study procedure that does not require documentation in the individual eCRF.

[a] Visit 2 is planned in the CMI subset of the study population (30% of the group population i.e., 38 participants in the group, at the designated CMI sites).

[b] Vital signs, urine sample for pregnancy test and blood sampling for RSV neutralizing titers, CMI and [redacted] must be taken on the same day, prior to study intervention administration. If the study intervention administration is delayed by any reason, the vital signs and samples (both blood and urine) will need to be collected again on the day of study intervention administration, urine pregnancy test needs to be repeated, and the first blood sample will be destroyed.

[c] Participants must bring their diary cards at this visit for review, and the same would be re-dispensed to them.

[d] eGFR measurements are performed in RTx patients at the central lab at screening visit. At subsequent visits, it is recorded if performed as SoC. Proteinuria/albuminuria is recorded, if performed as SoC in RTx patients.

[e] FEV1 results recorded only at health facility (hospital, clinic, site) will be used.

[f] AF will be considered as AESI in this study and will be additionally reported in the AF follow-up questionnaire (electronic or paper) in eCRF. The collection of AF will be performed following the AE/SAE reporting periods. The reporting of non-serious AF will be performed according to the unsolicited AE reporting period. The reporting of AF meeting the SAE definition (serious AF) will be performed according to the SAE reporting period.

Under special circumstances, home visits may be conducted at the discretion of the investigator (Refer to Section 8 for details).

Table 3 Schedule of Activities for the healthy participants group (RSV_HA)

Type of contact	Visit 1	Visit 2[a]	Visit 3	Visit 4	Visit 5	Visit 6	Notes
Time points	Day 1	Visit 1 + 7-14 days	Visit 1 + 30-60 days	Visit 3 + 30-42 days	Visit 1 + 180-210 days	Visit 1 + 350-380 days	
**Baseline and demography assessments**							
Informed consent	●						See Section 10.1.3 for details
Assignment/recording of participant number	●						See Section 6.3.1 for details
Check inclusion and exclusion criteria	●						See Sections 5.1 and 5.2 for details
Check with participants if he/she will appoint a caregiver, and distribute caregiver information letter to caregiver, when applicable	○	○	○	○	○	○	See Section 8 for details
Collect demographic data	●						See Section 8.1.1 for details
Record relevant medical history	●						See Section 8.1.2 for details
Record vaccination history	●						See Section 8.1.2 for details
Record height and weight	●						See Section 8.1.4 for details
History directed physical examination	○						See Section 8.1.5 for details
Record vital signs	●[b]						See Section 8.1.6 for details
Screening conclusion	●						
**Laboratory assessments**							
Urine pregnancy test (if applicable, WOCBP only)	●[b]						See Section 8.3.3 for details
Blood sampling (~ 8.5 mL) for RSV neutralizing titers	●[b]	●	●	●	●	●	See Section 8.2 for details
Blood sampling (~ 30 mL) for CMI response (in CMI subset only)	●[b]	●	●	●	●	●	See Section 8.2 for details
**Study intervention administration**							
Distribution of 'Participant card'	○						See Section 8.4.8 for details
Check contraindications, warnings, and precautions to vaccination	○						See Section 7.1.1 for details
Check criteria for temporary delay for enrollment and study intervention administration	○						See Section 5.5 for details

Type of contact	Visit 1	Visit 2[a]	Visit 3	Visit 4	Visit 5	Visit 6	Notes
**Time points**	**Day 1**	**Visit 1 + 7-14 days**	**Visit 1 + 30-60 days**	**Visit 3 + 30-42 days**	**Visit 1 + 180-210 days**	**Visit 1 + 350-380 days**	
Body temperature before study intervention administration	●						The location for measuring temperature will be oral or axillary (See Section 8.1.6). Fever is defined as temperature ≥38.0°C/100.4°F, regardless of the location of measurement.
Study group and intervention number allocation	○						See Section 6.3 for details
Study intervention administration (including 30-minutes post-study intervention administration observation)	●						See Section 6.1 for details
Recording of administered study intervention number	●						See Section 6.3.3 for details
**Safety assessments**							
Provide post-vaccination diary cards	○						See Section 10.3.5.1 for details
Train participants on completion of diary cards	○						See Section 10.3.5.1 for details
Return of post-vaccination diary cards		○[c]	○				See Section 10.3.5.1 for details
Verify and transcribe post-vaccination diary cards			●				See Section 10.3.5.1 for details
Targeted physical examination (as needed, SoC)		○	○	○	○	○	See Section 8.1.5 for details
Record concomitant medication/vaccination	●	●	●	●	●	●	See Section 6.9 for details
Record solicited administration site and systemic events (within 7 days post-study intervention administration)	●	●	●				See Section 10.3.5 for details
Record unsolicited AEs (within 30 days post-study intervention administration) [d]	●	●	●				See Section 10.3.5 for details
Record SAEs related to study participation or concurrent GSK medication/vaccine	●	●	●	●	●	●	See Section 10.3.5 for details

Type of contact	Visit 1	Visit 2[a]	Visit 3	Visit 4	Visit 5	Visit 6	Notes
**Time points**	**Day 1**	**Visit 1 + 7-14 days**	**Visit 1 + 30-60 days**	**Visit 3 + 30-42 days**	**Visit 1 + 180-210 days**	**Visit 1 + 350-380 days**	
Record:<br>- All SAEs [d]<br>- All pIMDs<br>- Pregnancies<br>- (S)AEs leading to withdrawal from the study<br>- Intercurrent medical conditions	●	●	●	●	●	●	See Section 10.3.5 for details
Study conclusion						●	See Section 4.4 for details

Note: The double-line borders indicate the analyses which will be performed on all data (i.e., data that are as clean as possible) obtained up to these timepoints.

AE = Adverse event; AESI = Adverse event of specific interest; AF = Atrial fibrillation; CMI = Cell-mediated immunity; eCRF = electronic case report form; pIMDs = Potential immune-mediated diseases; RSV = Respiratory Syncytial Virus; SAE = Serious adverse event; SoC = Standard of care; WOCBP = Women of childbearing potential.

● is used to indicate a study procedure that requires documentation in the individual eCRF.

○ is used to indicate a study procedure that does not require documentation in the individual eCRF.

[a] Visit 2 is planned in the CMI subset of the study population (30% of the group population i.e., 38 participants in the group, at the designated CMI sites).

[b] Vital signs, urine sample for pregnancy test and blood sampling for RSV neutralizing titers, CMI and [redacted] must be taken on the same day, prior to study intervention administration. If the study intervention administration is delayed by any reason, the vital signs and samples (both blood and urine) will need to be collected again on the day of study intervention administration, urine pregnancy test needs to be repeated, and the first blood sample will be destroyed.

[c] Participants must bring their diary cards at this visit for review, and the same would be re-dispensed to them.

[d] AF will be considered as AESI in this study and will be additionally reported in the AF follow-up questionnaire (electronic or paper) in eCRF. The collection of AF will be performed following the AE/SAE reporting periods. The reporting of non-serious AF will be performed according to the unsolicited AE reporting period. The reporting of AF meeting the SAE definition (serious AF) will be performed according to the SAE reporting period.

Under special circumstances, home visits may be conducted at the discretion of the investigator (Refer to Section 8 for details).

Table 4 Intervals between study visits for IC 1-dose group (RSV_IC_1)

Interval	Planned visit interval	Allowed interval range
Screening Visit → Visit 1	-7 days	-30 to -7 days
Visit 1 → Visit 2*	7 days	7 - 14 days
Visit 1 → Visit 3	30 days	30 - 60 days
Visit 3 → Visit 4	30 days	30 - 42 days
Visit 1 → Visit 5	180 days	180 - 210 days
Visit 1 → Visit 6	365 days	350 - 380 days

* Only in the CMI subset.

Table 5 Intervals between study visits for IC 2-dose group (RSV_IC_2)

Interval	Planned visit interval	Allowed interval range
Screening Visit → Visit 1	-7 days	-30 to -7 days
Visit 1 → Visit 2*	7 days	7 - 14 days
Visit 1 → Visit 3	30 days	30 - 60 days
Visit 3 → Visit 4	30 days	30 - 42 days
Visit 3 → Visit 5	180 days	180 - 210 days
Visit 3 → Visit 6	365 days	350 - 380 days

* Only in the CMI subset.

Table 6 Intervals between study visits for healthy participant group (RSV_HA)

Interval	Planned visit interval	Allowed interval range
Visit 1 → Visit 2*	7 days	7 - 14 days
Visit 1 → Visit 3	30 days	30 - 60 days
Visit 3 → Visit 4	30 days	30 - 42 days
Visit 1 → Visit 5	180 days	180 - 210 days
Visit 1 → Visit 6	365 days	350 - 380 days

* Only in the CMI subset.

2. INTRODUCTION

2.1. Study rationale

GSK has developed a RSVPreF3 OA vaccine against RSV-associated (subtypes A and B) disease in adults ≥60 YoA at an increased risk of RSV-LRTD. The vaccine was first approved for use in adults ≥60 YoA in the US on 03 May 2023, EU on 06 June 2023, UK on 07 July 2023, Canada on 04 Aug 2023, and Japan on 25 September 2023, under the tradename *Arexvy*. Additionally, immunocompromised patients such as SOT recipients, who are under chronic immunosuppressive therapy required to prevent organ rejection, are at high risk of severe RSV-LRTD. The aim of this open-label study is to evaluate the immunogenicity, safety, and reactogenicity of the RSVPreF3 OA investigational vaccine in an immunocompromised (lung and renal transplant recipients, 18 YoA and above) population and assess whether a second dose of the vaccine increases the immune response.

2.2. Background

RSV is an RNA virus of which 2 antigenically distinct subtypes exist, referred to as RSV-A and RSV-B [Borchers, 2013]. RSV causes RTI in people of all ages. In addition to the high BoD in children and older adults (≥60 YoA), RSV infection is an important cause of severe lower respiratory disease in the IC population [Pochon, 2019]. Currently, there are 2 vaccines available for prevention of RSV infection in adults ≥60 YoA; *Arexvy* (manufactured by GSK) and *Abrysvo* (manufactured by Pfizer Inc).

GSK is developing the adjuvanted RSVPreF3 OA vaccine (*Arexvy*) for patient populations at increased risk of RSV-LRTD.

Recent in-house results from a Phase 3 clinical trial in older adults (RSV OA=ADJ-004) demonstrated that one dose of the RSVPreF3 OA investigational vaccine-induced strong humoral and cell-mediated immune responses, which remained above pre-vaccination levels up to at least 13 months post-vaccination. In another large Phase 3 vaccine clinical trial (RSV OA=ADJ-006) in adults aged 60 years and above, the vaccine candidate demonstrated an overall vaccine efficacy of 82.6% (96.95% CI, 57.9–94.1) against RSV-LRTD up to 6 months post-vaccination. The vaccine was well tolerated with an acceptable safety profile [Papi, 2023]. The study is still ongoing to assess other endpoints. However, all studies done until now included only non-IC participants.

In general, IC patients are at increased risk of severe disease following an infection and tend to have lower immune responses following vaccination [Dropulic, 2016]. The IC population is a heterogenous group of patients which include patients with SOT, cancer patients (hematologic and solid organ tumors) on active treatment, patients on immunotherapy for autoimmune diseases, patients with congenital immuno-deficiencies, and patients living with HIV with low CD4 T-cell counts. Within the IC population, the SOT patients are under chronic immunosuppressive therapy required to prevent organ rejection, which places them at high risk of severe RSV-LRTD associated with a high morbidity and mortality [Dropulic, 2016; Fishman, 1998; Reichenberger, 2001].

The BoD of RSV infections is well-documented in lung transplant recipients, with mortality attributable to RSV-LRTD in this population reported from 10 to 20% [Neemann, 2015]. Although RSV burden in renal transplant recipients has been less thoroughly documented, those patients like other IC patients remain at increased risk for respiratory viral infections in general and RSV-LRTDs in particular, due to the IC status and immunosuppressive therapy. In general, IC patients exhibit lower immune responses following vaccination. Nevertheless, a recent clinical trial demonstrated that an adjuvanted RZV was immunogenic in chronically immunosuppressed renal transplant recipients ≥18 YoA [Vink, 2020].

Please refer to the IB for information regarding pre-clinical studies, and clinical studies of RSVPreF3 OA investigational vaccine.

2.3. Benefit/risk assessment

2.3.1. Risk assessment

Detailed information about the known and expected benefits, potential risks, and reasonably expected AEs of RSVPreF3 OA investigational vaccine can be found in the IB.

Potential Risk of Clinical Significance	Rationale for Risk	Mitigation Strategy
**RSVPreF3 OA investigational vaccine**		
pIMDs	pIMDs are considered a potential risk for this vaccine.	Refer to Section 8.4.4 for details.
Hypersensitivity reactions (including anaphylaxis)	Previous exposure to components of the vaccine might have induced an immune response that results in an exaggerated or inappropriate reaction.	All participants will remain under observation at the clinical center for at least 30 minutes after study intervention administration or longer if deemed necessary by site personnel.<br>Appropriate medical care must be readily available during this period.<br>Participants with a history of hypersensitivity or severe allergic reaction to any component of the vaccine are excluded from study enrollment.
Syncope (fainting)	Syncope (fainting) can occur following or even before study intervention administration as a psychogenic response to the needle insertion.	Participants who mention experiencing previous episodes of fainting or dizziness before, during or after vaccination, will be asked to lie down during the intervention and remain under observation at the clinical center for at least 30 minutes after study intervention administration or longer if deemed necessary by site personnel.<br>Appropriate medical care must be readily available during this period.
Allograft rejection	Allograft rejection is a theoretical concern with adjuvanted vaccines in solid organ transplant participants. To date, there is no evidence of an increased risk of allograft rejection as demonstrated in ZOSTER-041 study [Vink, 2020], nor with other vaccines.	During the informed consent process for vaccination, the participants will be informed of this potential risk and the need to inform the clinic/site and seek medical attention if unwell after vaccination. Clinical management of allograft rejection will be per local standard of care and recorded as AE/SAE, as appropriate. The occurrence of allograft rejection during the study will be described.
**Study procedures**		
Local reactions at the injection site	Intramuscular vaccination commonly precipitates a transient and self-limiting local inflammatory reaction. This may typically include pain at injection site, erythema/redness, and swelling.	Physician can implement the measures that they consider necessary.<br>Solicited local adverse events (AE) will be collected and reviewed up to Day 7 following vaccination.
Local reactions at site of blood draw	Pain, redness, irritation, and bruising may occur at the site where blood is drawn.	Physician can implement the measures that they consider necessary.

Potential Risk of Clinical Significance	Rationale for Risk	Mitigation Strategy
Syncope (fainting)	Syncope (fainting) can occur following or even before any blood draw as a psychogenic response to the needle insertion.	Participants who mention experiencing previous episodes of fainting or dizziness before, during or after a blood draw, will be asked to lie down during the intervention and remain under observation at the clinical center for at least 30 minutes after blood draw or longer if deemed necessary by site personnel.<br>Appropriate medical care must be readily available during this period.

For details of study procedures, dose, and study design justification, refer to Section 1.3 and Section 4.2.

In parallel with the RSVPreF3 OA clinical development program, another RSV vaccine development program was initiated by GSK, with the objective to prevent RSV-associated lower respiratory tract illness in neonates and infants during their first 6 months of life, through immunization of pregnant women with a single dose of the investigational RSV maternal vaccine candidate.

In 2020, GSK initiated a Phase 3, double-blind, 2:1-randomized, placebo-controlled study (RSV MAT-009; NCT04605159) in 24 countries to assess the safety and efficacy of the maternal vaccine candidate (RSVPreF3 Mat) administered to 18–49-year-old women in the late second or third trimester of pregnancy.

In February 2022, GSK decided to stop enrollment and vaccination in RSV maternal vaccine studies involving pregnant women. This decision was taken because of an observed imbalance in the proportions of both preterm births and neonatal deaths (death of an infant within the first 28 days of life) in the treatment group vs. the placebo group in the RSV MAT-009 study. Subsequently, the enrollment and vaccination in all studies of the RSV maternal vaccine candidate involving women of childbearing potential were also stopped.

Following the Day 43 post-birth interim analysis (DLP 04 October 2022) of the RSV MAT-009 study, GSK concluded that preterm birth is an identified risk for the pregnant women population, for the RSV maternal vaccine candidate. The observed numerical imbalance in neonatal deaths is not an independent safety signal but a consequence of the imbalance in preterm births. GSK has discontinued the further development of this RSV maternal vaccine candidate.

The safety concern is specific to women who received the RSV maternal vaccine candidate during the late second or third trimester of pregnancy. To date, analyses of the available safety data have not established what caused the observed imbalance in preterm births.

The vaccine candidate for older adults (RSV PreF3 OA vaccine), contains the same RSV antigen as the RSV maternal vaccine candidate but the RSVPreF3 OA vaccine is combined with GSK's established AS01E adjuvant to boost the immune response in the OA population.

CCI [redacted]. As a precautionary measure, no pregnant women will be included and all WOCBP will be required to use adequate contraception and have a negative pregnancy test prior to each vaccination in this study. Study participants will be adequately informed of the risks associated with pregnancy as the informed consent contains specific information regarding the RSV Maternal study.

The RSVPreF3 OA vaccine trials in participants ≥60 YoA are closely monitored for safety with all available safety data reviewed internally. In addition, the Phase 3 RSV OA=ADJ-006 clinical study is monitored by an IDMC on an ongoing basis. The IDMC has not raised any concern for safety in the OA population. The RSVPreF3 OA vaccine has not been studied in pregnant women to date.

2.3.2. Benefit assessment

The participants may or may not benefit directly from participating in this study. By receiving the RSVPreF3 OA vaccine, the participant may have the benefit of being protected against RSV-associated disease. In a prespecified efficacy interim analysis of an ongoing Phase 3 trial (RSV OA=ADJ-006) in participants ≥60 YoA, for those receiving a single dose of the RSVPreF3 OA investigational vaccine, the primary endpoint was met with a high vaccine efficacy during the first RSV season and no unexpected safety concerns were observed (refer to IB).

However, patients under immune suppression often respond less well to vaccines, and therefore, the information obtained in this study, namely whether a second dose can increase the immune response in IC patients, will further aid the development of the RSVPreF3 OA vaccine. It will be the first time the vaccine will be administered in IC participants ≥18 YoA, who are generally at an increased risk of developing severe RSV-LRTD.

Another benefit for all study participants may include gaining information about their general health status through the medical evaluations/assessments associated with this study (i.e., extra physical examination).

2.3.3. Overall benefit-risk conclusion

The RSVPreF3 OA investigational vaccine is in clinical development. Considering the measures taken to minimize the risk to participants in this study, the potential risks are justified by the potential benefits linked to the development of this vaccine.

3. OBJECTIVES, ENDPOINTS AND ESTIMANDS

Table 7 Objectives, endpoints and estimands

Objectives	Endpoints and estimands
**Primary**	
• To evaluate the humoral immune response against RSV-A following the first and the second dose of RSVPreF3 OA investigational vaccine within 2-dose group in renal and lung SOT patients.	• RSV-A serum neutralizing titers expressed as MGI post-Dose 2 (Visit 4*) over post-Dose 1 (Visit 3*) in renal and lung SOT patients in the 2-dose group.
• To evaluate the humoral immune response against RSV-B following the first and the second dose of RSVPreF3 OA investigational vaccine within 2-dose group in renal and lung SOT patients.	• RSV-B serum neutralizing titers expressed as MGI post-Dose 2 (Visit 4*) over post-Dose 1 (Visit 3*) in renal and lung SOT patients in the 2-dose group.
**Secondary-Immunogenicity**	
• To evaluate the humoral immune response to the RSVPreF3 OA investigational vaccine until 12 months post-study intervention administration in all groups.	• RSV-A and RSV-B serum neutralizing titers expressed as GMT, at pre-study intervention administration, Visit 2* (in a subset of participants), Visit 3*, Visit 4*, Visit 5* and Visit 6* in renal and lung SOT patients (1-dose and 2-dose group), and healthy participants.<br>• RSV-A and RSV-B serum neutralizing titers expressed as group GMT ratio RSV_HA over RSV_IC (pooled RSV_IC_1 and RSV_IC_2 groups) at Visit 2* (in a subset of participants) and Visit 3* and RSV_IC_2 over RSV_IC_1, RSV_HA over RSV_IC_1, and RSV_HA over RSV_IC_2) at Visit 4*, Visit 5* and Visit 6*.<br>• RSV-A and RSV-B serum neutralizing titers expressed as MGI at Visit 2* (in a subset of participants) over Visit 1, and Visit 3* over Visit 1* in RSV_HA and RSV_IC (pooled RSV_IC_1 and RSV_IC_2 groups) and at Visit 4*, Visit 5* and Visit 6* over Visit 1* in RSV_IC_1, RSV_2, RSV_HA groups.
• To evaluate the CMI response following RSVPreF3 OA investigational vaccine administration in a subset of participants in all groups.	• CMI response expressed as group geometric mean of the frequency of RSVPreF3-specific CD4+ and/or CD8+ T cells expressing at least 2 activation markers including at least one cytokine among CD40L, 4-1BB, IL-2, TNF-α, IFN-γ, IL-13, and IL-17 at pre-study intervention administration, Visit 2*, Visit 3*, Visit 4*, Visit 5* and Visit 6*, in a subset of participants (renal and lung SOT patients (1-dose and 2-dose group), and healthy participants).
**Secondary-Safety**	
• To evaluate the safety and reactogenicity following RSVPreF3 OA investigational vaccine administration in all groups.	• Percentage of participants reporting each solicited administration site event with onset within 7 days post-study intervention administration (i.e., the day of vaccination and 6 subsequent days).<br>• Percentage of participants reporting each solicited systemic event with onset within 7 days post-study intervention administration (i.e., the day of vaccination and 6 subsequent days).<br>• Percentage of participants reporting unsolicited AEs within 30 days post-study intervention administration (i.e., the day of vaccination and 29 subsequent days).

Objectives	Endpoints and estimands
	• Percentage of participants reporting SAEs after study intervention administration (Day 1) up to study end (Visit 6).<br>• Percentage of participants reporting pIMDs after study intervention administration (Day 1) up to study end (Visit 6).<br>• Percentage of participants reporting SAEs related to study intervention administration after study intervention administration (Day 1) up to study end (Visit 6).<br>• Percentage of participants reporting pIMDs related to study intervention administration after study intervention administration (Day 1) up to study end (Visit 6).<br>• Percentage of participants reporting fatal SAEs after study intervention administration (Day 1) up to study end (Visit 6).<br>• Percentage of participants reporting AESIs (specific to renal and lung SOT patients) after first study intervention administration (Day 1) up to study end (Visit 6).
**Tertiary**<br>*(Note that the tertiary objective, endpoints, and estimands are optional and will only be assessed if needed; therefore, not all testing might be performed and reported)*	
CCI [redacted]	

AE = Adverse event; AESI = Adverse event of special interest; CD = Cluster of differentiation; CMI = Cell-mediated immunity; CCI [redacted]; CCI [redacted]; IFN = Interferon; IL = Interleukin; GMT = Geometric mean titer; MGI = Mean geometric increase; CCI [redacted]; OA = Older adult; pIMD = Potential immune-mediated disease; RSV = Respiratory Syncytial Virus; SAE = Serious adverse event; SOT = Solid organ transplant; TNF = Tumor necrosis factor.
MGI is defined as the geometric mean of the within-participant ratios of antibody titers.
*Refer to Table 1, Table 2, and Table 3 for details on visits and intervals.

Details related to attributes of estimands covering intercurrent events, population and treatment definition are provided in the Section 9.

4. STUDY DESIGN

4.1. Overall design

The study design overview is presented in Figure 1.

Experimental design: Phase 2b, randomized, multi-center, open-label study with three parallel groups (see Figure 1).

Study groups and Randomization and enrollment rules: Approximately 375 eligible participants will be enrolled in the study, under the following groups.

- Approximately 250 IC adults ≥18 YoA who received a renal or lung SOT, will be randomly assigned (1:1) to the following 2 IC groups (1 dose versus 2 doses; 125 participants each) at Visit 1 (Day 1). Each group may include approximately 65% renal transplant patients and approximately 20% lung transplant patients, and the remaining patients can be freely distributed across the 2 groups.
  - **RSV_IC_1 group:** IC patients receiving 1 dose of RSVPreF3 OA investigational vaccine at Visit 1 (Day 1).
  - **RSV_IC_2 group**: IC patients receiving 2 doses of RSVPreF3 OA investigational vaccine at Visit 1 (Day 1) and Visit 3 (Visit 1 + 30-60 days).
- Approximately 125 healthy adults ≥50 YoA (distributed among age categories of 50-59 YoA (30 %), 60-69 YoA (30%), ≥70 YoA (20%), and 20% to be freely distributed over the different age categories) will be assigned to the healthy participants group.
  - **RSV_HA group**: Healthy participants receiving 1 dose of RSVPreF3 OA investigational vaccine at Visit 1 (Day 1).

Primary completion date: Visit 4 (1 month after the last study intervention administration).

Duration of the study: The total duration of the study, per participant, will be approximately 13-15 months for the IC patients and 12 months for the healthy participants.

Type of study: self-contained.

Data collection: A standardized eCRF will be used. Solicited events will be collected as of the day of each study intervention administration and for 6 subsequent days. Unsolicited AEs, new concomitant medications/vaccinations will be collected as of the day of each study intervention administration and for 29 subsequent days. As of Day 30 following each study intervention administration, the details are collected in source documents prior to entering them in the eCRF until study end.

Safety monitoring: The study will be monitored by an IDMC as well as the project SRT (Refer Section 8.3.4 and Section 10.1.6).

4.2. Scientific rationale for study design

RSV is associated with serious illness in OA and high-risk adults, including those who are immunocompromised that may lead to increased risk of RSV-LRTD. The efficacy of a single dose of the RSVPreF3 OA investigational vaccine in the prevention of RSV-LRTD in OA ≥60 YoA has been shown in the Phase 3 clinical study RSV OA=ADJ-006 (refer to IB).

Since the immune response in IC patients is often lower compared to healthy persons, the current study is designed as a Phase 2, open-label study to evaluate the immune response, safety, and reactogenicity of 1 versus 2 doses of RSVPreF3 OA investigational vaccine in

an IC population (renal and lung SOT patients) ≥18 YoA and additionally comparing it to 1 dose in healthy participants ≥50 YoA.

The inclusion of the 1-dose group in the SOT patient groups will provide more robust information and will help to make an informed decision on the need to use 1 dose versus 2 doses by comparing the immune response (in terms of GMT ratios in neutralization titers against RSV-A and RSV-B, as well as cell-mediated immunity) in the 1-dose group at Visit 3 and Visit 4 with the immune response in the 2-dose group at Visit 4.

In addition, a comparison on humoral and cell-mediated immunity at all timepoints up to 12 months post-last dose (including Month 6 and Month 12) between both groups of SOT patients as well as comparison to healthy participants will provide important information on waning of the immune response. In this study, the inclusion of a group of healthy adults ≥50 YoA will help to better elucidate the potential differences in immunogenicity and safety in the SOT patient groups versus healthy participants as of 50 YoA. Having head-to-head comparison with the healthy group will aid in the characterization of the immune response (both humoral and cell-mediated) of the IC population. Specifically for RSV, it is important that the healthy group is included in the same study since the primed status of the healthy adults and therefore the immune response to the vaccine, might differ between studies, as RSV seasonality can be different across countries/regions and years. Moreover, a head-to-head comparison with the healthy participants group will also enable further characterization of the reactogenicity of RSVPreF3 OA vaccine in IC population.

4.2.1. Participant input into design

Not applicable.

4.3. Justification for dose

A single dose (0.5 mL) or 2 doses of the licensed formulation (120 µg RSVPreF3/AS01E) will be used in this study.

Since the immune response in immunocompromised patients is often lower compared to healthy persons, this study will investigate the impact of a second dose given 1-2 months apart from the first dose. Therefore, the current study is designed as a Phase 2, open-label study to evaluate the immune response, safety, and reactogenicity of RSVPreF3 OA investigational vaccine in IC patients ≥18 YoA following the first and the second dose and additionally comparing it to 1 dose in healthy adults ≥50 YoA.

4.4. End of study definition

A participant is considered to have completed the study if the participant has completed all periods of the study including the last visit.

EoS: LSLV (Visit 6) or Date of the last testing/reading released of the Human Biological Samples, related to primary and secondary endpoints, whichever occurs later. EoS must be achieved no later than 8 months after LSLV. EoS cannot be before LSLV.

5. STUDY POPULATION

Prospective approval of protocol deviations to recruitment and enrollment criteria, also known as protocol waivers or exemptions, is not permitted.

Adherence to the inclusion and exclusion criteria specified in the protocol is essential.

5.1. Inclusion criteria

Participants are eligible to be included in the study only if all of the following criteria apply:

5.1.1. Inclusion criteria for all participants

All participants must satisfy ALL the following criteria at study entry:

- Participants and/or participant's parent(s)/LAR(s) who, in the opinion of the investigator, can and will comply with the requirements of the protocol (e.g., completion of the paper diary cards, return for follow-up visits, ability to access and utilize a phone or other electronic communications, have regular contact to allow evaluation during the study).

  Note: In case of physical incapacity that would preclude the self-completion of the diary cards, either site staff can assist the participant (for activities performed during site visits) or the participant may assign a caregiver to assist him/her with this activity (for activities performed at home). However, at no time will the site staff or caregiver evaluate the participant's health status while answering diary cards or make decisions on behalf of the participant. Refer to Definition of Terms for the definition of caregiver.

- Participants living in the general community or in an assisted-living facility that provides minimal assistance can be enrolled, such that the participant is primarily responsible for self-care and activities of daily living.
- Written or witnessed informed consent obtained from the participant/participant's parent(s)/LARs (participant must be able to understand the informed consent) prior to performance of any study-specific procedure.
- Female participants of nonchildbearing potential may be enrolled in the study. Non-childbearing potential is defined as hysterectomy, bilateral oophorectomy, bilateral salpingectomy, and post-menopause.
- Female participants of childbearing potential may be enrolled in the study if the participant
  - has practiced adequate contraception from 1 month prior to study intervention administration and agreed to continue adequate contraception until study end for this study, and

- has a negative pregnancy test on the day of and prior to study intervention administration.

  Refer to Section 10.4.1 for definitions of women of childbearing potential, non-childbearing potential, menarche and menopause and Section 10.4.2 on adequate contraception.

5.1.2. Specific inclusion criteria for renal/lung transplant patients

- A male or female participant, ≥18 YoA at the time of signing the Informed consent form (ICF) or Informed assent form (IAF).
- Written informed assent obtained from the participant (participant must be able to understand the informed assent) if he/she is less than legal age of consent*, or written informed consent obtained from the participant if the participant has achieved legal age of consent.

  **The legal age of consent is determined according to local regulations in each participating country. Assent must be obtained from the minor participant in addition to the consent provided by the participants' parent(s)/LAR(s) when a minor can assent to participate in a study. If the legal age is achieved during the conduct of the study, an additional written informed consent from the participant should be obtained at that time.*
- Participant who has received an ABO compatible allogeneic renal or lung transplant (allograft) more than 12 months (365 days) prior to the first study intervention administration.
- Participant receiving maintenance immunosuppressive therapy for the prevention of allograft rejection.

5.1.3. Specific inclusion criteria for renal transplant (RTx) patients

- Participant with stable renal function, stability defined as less than 20% variability between last two results of eGFR or in the opinion of the investigator after investigator review of more than the last two results of eGFRs and based on medical history.

5.1.4. Specific inclusion criteria for lung transplant (LTx) patients

- Participant with stable lung function, with stability defined as the stability in the FEV1 compared to post-transplant baseline FEV1 and based on medical history of the last 3 months, in the opinion of the investigator.

5.1.5. Specific inclusion criteria for healthy participants

- A male or female, ≥50 YoA at the time of signing the ICF.
- Healthy participants as established by medical history and clinical examination before entering the study.

- Participants who are medically stable in the opinion of the investigator at the time of first study intervention administration.
- Participants with chronic stable medical conditions with or without specific treatment, such as diabetes mellitus, hypertension, or cardiac disease, are allowed to participate in this study if considered by the investigator as medically stable.

5.2. Exclusion criteria

Participants are excluded from the study if any of the following criteria apply:

5.2.1. Medical conditions

- History of any reaction or hypersensitivity likely to be exacerbated by any component of the study intervention (For details on components of study intervention administered, refer to Table 8 and *Arexvy* SmPC/Prescribing information [Arexvy Summary of Product Characteristics, 2023; Arexvy Prescribing Information, 2023].
- Acute or chronic clinically significant cardiovascular or hepatic functional abnormality as determined by physical examination or laboratory screening tests.
- Recurrent or uncontrolled neurological disorders or seizures. Participants with medically controlled chronic neurological diseases can be enrolled in the study as per investigator assessment, provided that their condition will allow them to comply with the requirements of the protocol (e.g., completion of the diary cards, attend study site visits). Study participants may decide to assign a caregiver to help them complete some of the study procedures (Refer to Section 8).
- Any history of dementia or any medical condition that moderately or severely impairs cognition.

  Note: If deemed necessary for clinical evaluation, the investigator can use tools such as Mini-Mental State Exam, Mini-Cog or Montreal Cognitive Assessment (to determine cognition levels of the participant).
- Any condition which, in the judgment of the investigator, would make IM injection unsafe.
- Significant underlying illness that in the opinion of the investigator would be expected to prevent completion of the study (e.g., life-threatening disease likely to limit survival up to study end).
- Acute disease and/or fever at the time of study intervention administration. Fever is defined as temperature ≥ 38°C /100.4°F determined by oral or axillary route. However, participants with a minor illness (such as mild diarrhea, mild upper respiratory infection) without fever may be enrolled at the discretion of the investigator.
- Bedridden participants.

- Any other clinical condition that, in the opinion of the investigator, might pose additional risk to the participant due to participation in the study.

5.2.2. Prior/Concomitant therapy

- Use of any investigational or non-registered product (drug, vaccine, or medical device) other than the study intervention administration during the period beginning 30 days before the first dose of study intervention administration (Day -30 to Day 1), or their planned use during the study period (up to Visit 6).
- Previous vaccination with the same antigen (RSV) containing vaccine as that of the study intervention, including investigational RSV vaccines.
- Planned or actual administration of a vaccine not foreseen by the study protocol in the period starting 30 days before the first dose of study intervention administration and ending 30 days after the last dose of study intervention administration*. In the case of COVID-19 and inactivated/subunit/split influenza vaccines, this time window can be decreased to 14 days before and after each study intervention administration.

  **If emergency mass vaccination for an unforeseen public health threat (e.g., a pandemic) is recommended and/or organized by the public health authorities outside the routine immunization program, the time period of 30 days described above can be reduced, if necessary for that vaccine, provided it is used according to the local governmental recommendations and that the Sponsor is notified.*

5.2.3. Prior/Concurrent clinical study experience

- Concurrently participating in another clinical study, at any time during the study period, in which the participant has been or will be exposed to an investigational or a non-investigational intervention (drug/invasive medical device).

5.2.4. Other exclusion criteria

- Pregnant or lactating female participant.
- Female participant planning to become pregnant or planning to discontinue contraceptive precautions.
- History of chronic alcohol consumption and/or drug abuse as deemed by the investigator to render the potential participant unable/unlikely to provide accurate safety reports or comply with study procedures.
- Participation of any study personnel or their immediate dependents, family, or household members.
- Planned move during the study period that will prohibit participating in the study until study end.

5.2.4.1. Specific exclusion criteria for renal/lung transplant patients:

- More than one organ transplanted (i.e., kidney-liver or kidney-other organ(s) transplanted). Dual organ is allowed (double kidney or double lung).
- History of events that, in the opinion of the investigator, may put the participant at increased risk for chronic allograft dysfunction.
- Participant with an episode of allograft rejection over the previous 3 months (90 days) prior to the first study intervention administration.
- Histologic evidence of chronic allograft injury.
- Active treatment for acute rejection.
- Current diagnosis of malignancy (except non-melanoma skin cancer that does not require systemic therapy).
- Any autoimmune conditions or pIMDs that in the opinion of the investigator may put the participant at increased risk.
- Any confirmed or suspected HIV infection or primary immunodeficiency disease or ongoing CMV infection with a viremia > 200 IU/mL.
- Use of anti-CD20 or other B-cell monoclonal antibody agents (e.g., rituximab) as induction, maintenance and/or therapeutic immunosuppressive therapy for the prevention of allograft rejection within 9 months (274 days) of first dose of study.
- Use of investigational and non-registered immunosuppressants at the local/country level, unless specifically prescribed for the prevention of allograft rejection, and which are non-registered and:
  - available locally through compassionate use programs,
  - submitted for and pending local/country registration,
  - approved and registered for use in other countries with well-documented SmPC or Prescribing Information. The name of the active component(s) of these immunosuppressants must be provided in the concomitant medication listing.
- Evidence or high suspicion, in the opinion of the investigator, of noncompliance or nonadherence to use of induction and/or maintenance immunosuppressive therapies.
- Any clinically significant* hematologic (hemoglobin level, white blood cell, lymphocyte, neutrophil, eosinophil, platelet red blood cell count and erythrocyte mean corpuscular volume) and/or biochemical (ALT, AST, creatinine, blood urea nitrogen) laboratory abnormality.

  ** The investigator should use their clinical judgment to decide which abnormalities are clinically significant.*

5.2.4.2. Specific exclusion criteria for RTx patients:

- Previous allograft loss secondary to recurrent primary kidney disease. Multiple consecutive kidney transplants are allowed if the reason for a previous allograft loss is not recurrent primary kidney disease.
- Evidence of significant proteinuria/albuminuria in the opinion of the investigator.

5.2.4.3. Specific exclusion criteria for LTx patients:

- At study intervention administration visit, diagnosis of documented acute pulmonary infection within the 2 prior weeks, based on the following: clinical, radiological, and physiological deterioration; OR isolation of an organism from a clinically relevant BAL fluid culture.
- Patients with diagnosis of chronic lung allograft dysfunction, defined as a decrement of 20% or more in FEV1 compared to post-transplant baseline FEV1.

5.2.4.4. Specific exclusion criteria for healthy participants:

- Any confirmed or suspected immunosuppressive or immunodeficient condition resulting from disease (e.g., current malignancy, HIV) or immunosuppressive / cytotoxic therapy (e.g., medication used during cancer chemotherapy, organ transplantation, or to treat autoimmune disorders), based on medical history and physical examination (no laboratory testing required).
- Unstable serious chronic illness.
- Chronic administration of immune-modifying drugs (defined as more than 14 consecutive days in total) and/or administration of long-acting immune-modifying treatments or planned administration at any time up to the end of the study.
  - Up to 3 months prior to the study intervention administration:
    - For corticosteroids, this will mean prednisone equivalent ≥20 mg/day, or equivalent. Inhaled, topical and intra-articular steroids are allowed.
    - Administration of immunoglobulins and/or any blood products or plasma derivatives.
  - Up to 6 months prior to study intervention administration: long-acting immune-modifying drugs including among others, immunotherapy (e.g., TNF-inhibitors), monoclonal antibodies, antitumoral medication.

5.3. Lifestyle considerations

No restrictions are required.

5.4. Screen failures

A screen failure occurs when a participant who has consented to participate in the clinical study is not subsequently entered in the study. A minimal set of screen failure information is required to ensure transparent reporting of screen failure participants to meet the CONSORT publishing requirements and to respond to queries from regulatory authorities. Minimal information includes demography, screen failure details, eligibility criteria, and any SAE.

Individuals who do not meet the criteria for participation in this study (screen failure) may be rescreened once. Rescreened participants should be assigned a new participant number at the rescreening event. Previously assigned participant number will be recorded in the participants' eCRF.

5.5. Criteria for temporarily delaying enrollment/ administration of study intervention

Enrollment/study intervention administration may be postponed within the permitted visit interval until transient conditions cited below are resolved and before the end of recruitment period:

- Participants (SOT patients) with any clinically significant (in the opinion of the investigator) hematologic/biochemical values out of normal range which are expected to be temporary may be retested once at a later date within the allowed visit interval.
- Acute disease and/or fever at the time of study intervention administration. Refer to the SoA (Section 1.3) for definition of fever and preferred location for measuring temperature in this study.
- Participants with a minor illness (such as mild upper respiratory infection, mild diarrhea) without fever may be enrolled and/or dosed at the discretion of the investigator.
- In case of administration of vaccines, study intervention administration should be postponed, within given protocol timelines and prior to the end of the study enrollment period, to allow respect of at least 30 days interval between other vaccination and study intervention administration.
- In case of administration of inactivated, subunit and split influenza vaccines or COVID-19 vaccines (fully licensed or with EUA): postponement of study intervention administration within given protocol timelines and prior to the end of the study enrollment period, to allow respect of at least 14 days interval between flu/COVID-19 vaccination and study intervention administration.
- Participants with symptoms suggestive of active COVID-19 infection (e.g., fever, cough, etc.). The return of the participant to the site will follow the specific guidance from local public health and other competent authorities (e.g., free of symptoms, COVID-19 negative testing, etc.).

- Participants with known COVID-19 positive contacts may be dosed at least 14 days after the exposure, provided that the participant remains symptom-free, and at the discretion of the investigator.

6. STUDY INTERVENTION AND CONCOMITANT THERAPY

The definition of study intervention is provided in the Definition of Terms.

6.1. Study intervention administered

Study intervention administered is mentioned in Table 8 below. Refer to Section 4.1 for study intervention administration schedule.

Table 8 Study Intervention Administered

**Study intervention name:**	**RSVPreF3 OA Investigational Vaccine**	
**Vaccines/ product formulation**	RSVPreF3 (120 µg)	AS01E: QS-21* (25 µg), MPL (25 µg), liposomes; Water for injections
**Presentation**	Powder for suspension for injection (Vial)	Suspension for suspension for injection (Vial)
**Type**	Study	
**Product category**	Biologic	
**Route of administration**	IM	
**Administration site**		
**Location**	Deltoid	
**Directionality**	Upper	
**Laterality****	Non-dominant	
**Number of doses to be Administered**	1 dose (RSV_IC_1 and RSV_HA groups)<br>2 doses (RSV_IC_2 group)	
**Volume to be administered by dose*****	0.5 mL	
**Packaging and labeling**	Refer to Pharmacy Manual for details	
**Manufacturer**	GSK	

AS01E = Adjuvant System 01; QS-21 = *Quillaja saponaria* Molina, fraction 21; MPL = Monophosphoryl lipid A.
* Licensed by GSK from Antigenics Inc, a wholly owned subsidiary of Agenus Inc., a Delaware, USA corporation.
** The non-dominant arm is the preferred arm of injection. In case it is not possible to administer the study intervention in the non-dominant arm, an injection in the dominant arm may be performed.
*** Refer to the Pharmacy Manual for the volume after reconstitution.

Study participants must be observed closely for at least 30 minutes after the administration of the study intervention. Appropriate medical treatment and equipment must be readily available during the observation period in case of anaphylaxis or syncope.

6.1.1. Medical devices

- There are no GSK manufactured medical devices (or devices manufactured for GSK by a third party) provided for use in this study. Other medical devices (not manufactured by or for GSK) provided for use in this study are thermometer for body temperature measurement, ruler for skin reaction measurement, materials for study intervention administration, syringes, blood collection kits, and cup for urine collection.

- Instructions for medical device use are provided in Laboratory Manual and Pharmacy Manual.
- All device deficiencies (including malfunction, use error and inadequate labeling) shall be documented and reported by the investigator throughout the clinical investigation (see Section 8.4.7) and appropriately managed by GSK.

6.2. Preparation, handling, storage, and accountability

The investigator or designee must confirm appropriate conditions (e.g., temperature) have been maintained during transit for all study intervention received, and any discrepancies are reported and resolved before use of the study intervention.

Only participants enrolled in the study may receive study intervention, and only authorized site staff may supply, prepare, or administer study intervention. If allowed by country regulation/ethics, study intervention may be administered by a HHS professional.

All study intervention must be stored in a secure, environmentally controlled, and monitored (manual or automated) area in accordance with the labeled storage conditions with access limited to the investigator and authorized site staff.

The investigator, institution, the head of the medical institution (where applicable), or authorized site staff is responsible for study intervention accountability, reconciliation, and record maintenance (i.e., receipt, reconciliation, and final disposition records).

Further guidance and information for the final disposition of unused study interventions are provided in the pharmacy manual.

6.3. Assignment to study intervention

6.3.1. Participant identification

Participant identification numbers will be assigned sequentially to the individuals who have consented to participate in the study. Each study center will be allocated a range of participant identification numbers.

6.3.2. Randomization to study intervention

Immunocompromised adults ≥18 YoA who received a renal or lung SOT will be randomly assigned in a 1:1 ratio at Visit 1 (Day 1) to RSV_IC_1 group or RSV_IC_2 group to receive either 1 dose or 2 doses of the RSVPreF3 OA investigational vaccine, respectively.

All participants in RSV_HA group (Healthy adults ≥50 YoA) will receive 1 dose of the RSVPreF3 OA investigational vaccine at Visit 1 (Day 1).

The assignment of clinical trial supplies will be performed using a GSK Randomization and Trial Supply Management (RTSM) system.

6.3.3. Intervention allocation to the participant

The order of study intervention assignment is generated (permuted block randomization) and stratified by SOT type and CMI subset using a GSK RTSM system. The subject level study intervention assignment activity is conducted using a GSK RTSM system.

Each IC group may include approximately 65% renal transplant patients and approximately 20% lung transplant patients, and the remaining patients can be freely distributed across the 2 groups. Recruitment caps for patient transplant populations are managed by the RTSM system.

For the healthy participant group, participants will be enrolled in 3 age categories, with approximately 30% of participants 50-59 YoA, approximately 30% of participants 60-69 YoA, and approximately 20% of participants ≥ 70 YoA. The remaining 20% can be distributed freely across the 3 age categories. Recruitment caps for age group populations are managed by the RTSM system.

Within each group 30% participants will be assigned to CMI subset (See Section 6.3.4 for details).

The RTSM system will assign randomisation number and corresponding study group at time of randomisation as well as the study intervention number at time of each study intervention administration.

When RTSM system is not available, please refer to the Pharmacy Manual for instructions.

Refer to the Pharmacy Manual for additional information about the study intervention number allocation.

6.3.4. Allocation of participants to CMI assay subsets

Participants contributing to the CMI subset will be recruited from a selected number of countries and selected number of clinical sites. In the selected sites, the investigator will allocate if possible, at Visit 1, the first participants in each group to the CMI subset until the allocated target is reached. The subsets are detailed in Table 9 below.

Table 9 Allocation to CMI subset

Group	Subset	Number of participants
RSV_IC_1	CMI	~38
	Non-CMI	~87
RSV_IC_2	CMI	~38
	Non-CMI	~87
RSV_HA	CMI	~38
	Non-CMI	~87
**Total**		**~375**

CMI = Cell-mediated immunity; HA = Healthy adults; IC = immunocompromised; RSV = Respiratory Syncytial Virus.

6.4. Blinding

This is an open-label study; potential bias will be reduced by central randomization.

6.5. Study intervention compliance

Participants will receive study intervention at the site, directly from the investigator or designee, under medical supervision. The date and time of each dose administered in the clinic will be recorded in the source documents.

A record of the quantity of RSVPreF3 OA Investigational Vaccine administered to each participant must be maintained and reconciled with study intervention and compliance records.

6.6. Dose modification

Not applicable.

6.7. Continued access to study intervention after the end of the study

There is no plan to provide continued access to the study intervention following the end of the study.

6.8. Treatment of overdose

Not applicable.

6.9. Prior and concomitant therapy

The following concomitant medication(s)/product(s)/vaccine(s) must be recorded in the eCRF:

- All concomitant medication leading to elimination from the analysis, including products/vaccines. Please refer to the Section 5.2.2 for further details.
- All concomitant medication which may explain/cause/be used to treat an SAE/pIMD/AESI including vaccines/products, as defined in Section 8.4 and Section 10.3.5.7. These must also be recorded in the Expedited AEs Report.
- For all AF AESIs (including serious and non-serious), concomitant drugs which could be associated with development or worsening of AF must be reported in the AF follow-up questionnaire.
- Any prophylactic medication (e.g., analgesics, antipyretics) administered on the day of study intervention administration (Day 1) in the absence of any symptom and in anticipation of a reaction to the study intervention administration.

- Any medication or vaccine (including over-the-counter or prescription medicines) or other specific categories of interest (immunosuppresive medication for SOT patients) that the participant is receiving at the time of enrollment or up to 30 days post-last study intervention administration must be recorded along with:
  - Reason for use (indication)
  - Dates of administration including start and end dates
  - Dosage information including dose and frequency
- Any antipyretic administered in the period starting 6 hours before vaccination and ending 12 hours after vaccination needs to be recorded on the eCRF.

The Medical monitor should be contacted if there are any questions regarding concomitant or prior therapy.

Medications that are contraindicated for use in this study are stipulated in the exclusion criteria in Section 5.2.2, Section 5.2.4.1 and Section 5.2.4.4.

Table 10 describes prohibited medications (that could impact evaluation of participants baseline status, safety and efficacy assessments) and the required washout period prior to study intervention administration. Generally, these medications are prohibited until after Visit 6, unless otherwise stated below.

Medications that are not stipulated in the exclusion criteria or listed in Table 10 may be used based on the discretion of the investigator (after careful evaluation whether or not the medication could impact evaluation of the clinical trial objective or the safety of the participant).

Table 10 Prohibited medications and washout period

Prohibited medications	Washout period
Investigational or non-registered product	30 days prior to first dose until Visit 6[1]
Vaccine	30 days prior to first dose until 30 days after last dose[2]
Immune-modifying drugs >14 consecutive days in total[3]	3 months prior to first dose until Visit 6[4]
Long-acting immune-modifying treatments (e.g., immunotherapy (e.g., TNF-inhibitors), monoclonal antibodies, antitumoral medication)[3]	6 months prior to first dose until Visit 6
Immunoglobulins and/or any blood products or plasma derivatives[3]	3 months prior to first dose until Visit 6
Anti-CD20 or other B-cell monoclonal antibody agents (e.g., rituximab) as induction, maintenance and/or therapeutic immunosuppressive therapy for the prevention of allograft rejection[5]	9 months (274 days) prior to first dose until Visit 6

[1] For renal/lung transplant patients, the use of investigational and non-registered immunosuppressants at the local/country level are contraindicated, unless specifically prescribed for the prevention of allograft rejection, and which are non-registered and: available locally through compassionate use programs; submitted for and pending local/country registration; approved and registered for use in other countries with well-documented SmPC or Prescribing Information.

[2] Any vaccine. However, for COVID-19 and inactivated/subunit/split influenza vaccines (fully licensed or with emergency use authorisation [EUA]), this time window can be decreased to 14 days before and after each dose. If emergency mass vaccination for an unforeseen public health threat (e.g., a pandemic) is recommended and/or organized by the public health authorities outside the routine immunization program, the time period of 30 days

described above can be reduced, if necessary for that vaccine, provided it is used according to the local governmental recommendations and that the Sponsor is notified. Live attenuated vaccines should not be administered throughout the study for SOT patients as a part of SoC.

[3] Applicable only for healthy participants.

[4] For corticosteroids, this will mean prednisone equivalent ≥20 mg/day, or equivalent. Please note: Inhaled, topical and intra-articular steroids are allowed.

[5] Applicable only for SOT patients.

Table 11 Timing of collection of concomitant medication to be recorded

	Dose 1<br>Day 1	Day 30	Study conclusion<br>(Visit 6 at Month 12-15)
All concomitant medication including vaccines/products, except vitamins and dietary supplements#			
All concomitant medication including products/vaccines leading to elimination from the applicable analysis			
All concomitant medication including vaccines/products which may explain/cause/be used to treat an SAE/pIMD/AESI*			
Any prophylactic medication#			

AESI = Adverse event of special interest; AF = Atrial Fibrillation; pIMD = Potential immune-mediated disease; SAE = Serious adverse event.

Note: The collection period for the concomitant medications to be recorded in eCRF is indicated in gray.

# For the RSV_IC_2 group, the time period corresponds to both dose 1 and dose 2 of study intervention administration.

* For all AF AESIs (including serious and non-serious), concomitant drugs which could be associated with development or worsening of AF must be reported in the AF follow-up questionnaire.

7. DISCONTINUATION OF STUDY INTERVENTION AND PARTICIPANT DISCONTINUATION/WITHDRAWAL

7.1. Discontinuation of study intervention

Discontinuation of study intervention refers to any participant who has not received all planned doses of the study intervention. In rare instances, it may be necessary for a participant to permanently discontinue study intervention. If study intervention is permanently discontinued, the participant should, if possible, continue other study procedures (e.g., safety or immunogenicity), planned in the study protocol at the discretion of the investigator.

The primary reason for premature discontinuation of the study intervention will be documented in the eCRF based on the list below:

Reasons	Additional items/Sub-reasons
AE	Unsolicited AE
	Solicited AE
	SAEs / pIMDs /AESIs
Lost to follow-up	
Withdrawal by Participant	Burden of Procedure
	Participant Relocated
	COVID-19

Reasons	Additional items/Sub-reasons
	Other, Specify
Investigator Decision	Specify
Protocol Deviation	Specify
Site Terminated by Sponsor	
Study Terminated by Sponsor	
Pregnancy	
Death	
Other	Specify

AE = Adverse event; AESI = Adverse event of special interest; COVID-19 = Coronavirus disease; pIMD = Potential immune-mediated disease; SAE = Serious adverse event.

7.1.1. Contraindications to subsequent study intervention administration

The eligibility for subsequent study intervention administration must be confirmed before administering any additional dose.

Participants who meet any of the criteria listed below or criteria listed in Sections 5.2.1 and 5.2.2 should not receive additional doses of study intervention. Such participants should be encouraged to continue other study procedures, at the investigator's discretion. All relevant criteria for discontinuation of study intervention administration must be recorded in the eCRF.

- Participants who experience any SAE judged to be possibly or probably related to study intervention or non-study concomitant vaccine/product, including hypersensitivity reactions.
- Participants who develop any new condition which, in the opinion of the investigator, may pose additional risk to the participants if they continue to participate in the study.
- Anaphylaxis following the administration of study intervention.
- Condition that in the judgment of the investigator would make intramuscular injection unsafe.
- Occurrence of a new pIMD or the exacerbation of an existing pIMD that, in the opinion of the investigator, expose the participant to unacceptable risk from subsequent vaccination. In such cases, the investigator should use their clinical judgment prior to administering the next dose of the study intervention. Refer to Section 8.4.4.1 for the definition of pIMD.
- Occurrence of a new AESI or the exacerbation of an existing AESI that, in the opinion of the investigator, exposes the participant to unacceptable risk from subsequent doses of study intervention. Refer to Section 8.4.4 for the definition of AESI.

- Pregnant or lactating female participant (Refer to Section 8.4.6).

7.2. Participant discontinuation/withdrawal from the study

A participant may withdraw from the study at any time at the participant's own request for any reason (or without providing any reason).

A participant may be withdrawn at any time at the discretion of the investigator for safety, behavioral, or compliance reasons.

Investigators will attempt to contact participants who do not return for scheduled visits or follow-up.

All data and samples collected up to and including the date of withdrawal of/last contact with the participant will be included in the study analyses. If the participant withdraws consent for disclosure of future information, the sponsor may retain and continue to use any data collected before such a withdrawal of consent.

A participant can request for the destruction of their samples any time during the study. The investigator must document the request in the site study records. If a participant withdraws from the study, any samples taken and not tested, should not be tested, and should be destroyed, if applicable, as per local regulations.

The primary reason for participant discontinuation/ withdrawal from the study will be documented in the eCRF based on the list below:

Reasons	Additional items/Sub-reasons
AE	Unsolicited AE
	Solicited AE
	SAEs / pIMDs /AESIs
Lost to follow-up	
Withdrawal by Participant	Burden of Procedure
	Participant Relocated
	COVID-19
	Other, Specify
Investigator Decision	Specify
Protocol Deviation	Specify
Site Terminated by Sponsor	
Study Terminated by Sponsor	
Pregnancy	
Death	
Other	Specify

AE = Adverse event; AESI = Adverse event of special interest; COVID-19 = Coronavirus disease; pIMD = Potential immune-mediated disease; SAE = Serious adverse event.

Participants who are withdrawn from the study because of AEs/SAEs must be clearly distinguished from participants who are withdrawn for other reasons. Investigator will follow participants who are withdrawn from the study due to an AE/SAE/AESI until the event is resolved, stabilized, otherwise explained, or the participant is lost to follow-up (see Section 10.3.5.5).

7.3. Lost to follow-up

A participant will be considered lost to follow-up if the participant repeatedly fails to return for scheduled visits and is unable to be contacted by the study site.

The following actions must be taken if a participant fails to return to the clinic for a required study visit:

- The site must attempt to contact the participant/caregiver and reschedule the missed visit as soon as possible, within the allowed visit interval, counsel the participant on the importance of maintaining the assigned visit schedule and ascertain whether the participant wishes to and/or should continue in the study.
- Before a participant is deemed lost to follow-up, the investigator or designee must make every effort to regain contact with the participant/caregiver (where possible, 3 telephone calls, and if necessary, a certified letter to the participant’s last known mailing address or local equivalent methods). These contact attempts should be documented in the participant’s medical record.
- Should the participant continue to be unreachable, the participant will be considered to have withdrawn from the study.
- Site personnel, or an independent third party, will attempt to collect the vital status of the participant within legal and ethical boundaries for all participants randomized, including those who did not get study intervention. Public sources may be searched for vital status information. If vital status of the participant is determined as deceased, this will be documented and the participant will not be considered lost to follow-up. Sponsor personnel will not be involved in any attempts to collect vital status information.

8. STUDY ASSESSMENTS AND PROCEDURES

- Study procedures and their timing are summarized in the SoA (Section 1.3). Protocol waivers or exemptions are not allowed.
- Adherence to the study design requirements, including those specified in the SoA, is essential and required for study conduct.
- Immediate safety concerns should be discussed with the sponsor as soon as they occur or when the study team becomes aware of them. In case of doubts of immediate safety concerns regarding the inclusion of a possible participant, inclusion should be postponed until a decision can be made.

- All screening evaluations must be completed and reviewed to confirm that potential participants meet all eligibility criteria. Subjects who have signed informed consent but are not eligible to proceed should be recorded in the eCRF with a status of 'screen failure'. The investigator will maintain a log of all participants screened. All relevant information, such as confirmation of eligibility and reasons for screening failure will be mentioned in this screening log.
- Procedures conducted as part of the participant's routine clinical management (e.g., blood count) and obtained before signing of the ICF, and those conducted during the entire duration of the study may be utilized for screening or baseline purposes, as well as for safety follow-up (e.g., SoC tests to assess occurrence of allograft rejection in SOT patients) provided the procedures met the protocol-specified criteria and were performed within the timeframe defined in the SoA.
- Study participants may decide to assign a caregiver to help them complete the study procedures. Please refer to the Definition of Terms for the definition of a caregiver.
  - A caregiver can be appointed by the participant at any time during the study, when the participant feels it is necessary. Caregiver should receive the caregiver information letter before providing support to the study participant. Ideally, a single caregiver should be appointed by the participant but, in some situations, it may happen that several caregivers will support a study participant throughout the conduct of the study. This should be recorded in the source documents. However, every effort should be done to ensure that only one caregiver enters the data into the paper diary to allow for timely completion.
  - Caregivers may help the study participants with performing some practical study procedures such as receiving or making phone calls to site staff, planning study visits, transcribing responses to diaries, transportation to and from the study site etc. However, at no time, the caregiver should evaluate the participant's health status while answering diaries or make decisions on behalf of the participant. At the first study visit, the site staff should inform the participant of the possibility to appoint a caregiver. Then at subsequent study visit(s), the site staff should check again with the participants if they wish to appoint a caregiver or if there were or will be changes of caregiver.
- In the event of a significant study-continuity issue (e.g., caused by a pandemic), alternate strategies for participant visits, assessments, study intervention distribution and monitoring may be implemented by the sponsor or the investigator, as per local health authority/ethics requirements.
- It is preferred to have all visits on-site. However, during special circumstances (e.g., COVID-19 pandemic, SOT patients in whom health has deteriorated during the study, or for whom the transfer to the clinic has become very difficult), the specific guidance from local public health, if applicable, and other competent authorities regarding the protection of individuals' welfare must be applied and consulted with the sponsor. For the duration of such special circumstances, the following measures may be implemented for enrolled participants:

- Follow-up visits (Visit 3 in HA and IC 1-dose groups, and Visits 2, 4, 5, and 6 for all groups) may be conducted at the participant's home (by the site staff or by a home care service system), if appropriate. If home visits are not possible, telephone calls or other means of virtual contacts may be used.
- Diary cards may be transmitted from and to the site by electronic means or courier service if allowed by local regulations and or conventional mail or collected at home. Biological samples may be collected at participant's home. Biological samples should not be collected if they cannot be processed in a timely manner or appropriately stored until the intended use. Home Visits are not allowed for participants in the CMI subset (due to time-sensitivity of CMI samples) except in special circumstances and with the prior approval of the GSK central study team.

- Impact of visits performed outside of the site's location on the PPS for immunogenicity will be determined on a case-by-case basis.
- The maximum amount of blood collected from each participant over the duration of the study, related to procedures in this study, will not exceed 288 mL. Repeat or unscheduled samples may be taken for safety reasons or for technical issues with the samples.

8.1. Administrative and baseline procedures

8.1.1. Collection of demographic data

Record demographic data such as year of birth, age at the time of study intervention administration, sex, race*, and ethnicity* in the participant's eCRF.

Collection of sex, race and ethnicity data is necessary to assess and monitor the diversity of the trial participants, and to determine if the trial participants are truly representative of the impacted population.

*Differences in the safety and efficacy of certain medical products, including vaccines [Haralambieva, 2013; Pérez-Losada, 2009; Kollmann, 2013] have been observed in racially and ethnically distinct subgroups. These differences may be attributable to intrinsic factors (e.g., genetics, metabolism, elimination), extrinsic factors (e.g., diet, environmental exposure, sociocultural issues), or interactions between these factors. Therefore, both geographic ancestry (race) and ethnicity will be collected for all study participants.

Age is collected to stratify the population and determine the impact of the study intervention by age.

8.1.2. Medical/vaccination history

Obtain the participant's relevant medical/vaccination history by interviewing the participant/participant's parent(s)/LAR(s) and/or review of the participant's medical/vaccination records. Record any relevant pre-existing conditions, signs and/or symptoms present prior to the study intervention in the eCRF.

Any vaccine administered up to 1 year before study vaccine administration should be recorded in the eCRF with date of vaccination. For history of influenza vaccination, information about the vaccine formulation (e.g., adjuvanted or non-adjuvanted or highdose) should be recorded.

Administration of *Shingrix* at any timepoint (even if longer than 1 year before the study vaccine administration) should be recorded in the eCRF. The date of vaccinations should be collected and recorded in the eCRF.

Specifically additional data will be collected for transplant participants as listed in Section 8.1.3.

8.1.3. Record specific participant transplant information

The following information will be recorded (if available in medical history of the lung/renal transplant recipient patient) and entered into the eCRF at screening visit:

Current transplant history

Information will be collected and entered eCRF about the current transplant, including:

- Type of transplant (one or two kidneys, one or two lungs)
- Date of transplantation
- Biopsies since transplantation (Yes/No), and if yes,
  - collect the results indicating rejection and type of rejection (Acute or Chronic)
- Current allograft function with options (stable for 3 to ≤6 months, stable for 6 to ≤12 months, or stable for >12 months)

Previous transplant

Information will be collected and entered eCRF about the previous transplants (if any before the current), including:

- Type of transplant (one or two kidneys, one or two lungs)
- Date of transplantation
- Data about allograft loss (Yes/No), and if yes,
  - collect the reasons for allograft loss

In case of multiple previous transplantations, all previous transplantations should be recorded with the information as above.

8.1.4. Record height and weight

Measure the participant’s height and weight and record the values in the eCRF.

8.1.5. Targeted physical examination/history-directed physical examination

A history-directed physical examination will be performed for each participant at the screening visit in SOT patients and Visit 1 in healthy participants. At each subsequent visit, a targeted physical examination will be performed, as needed.

If the investigator determines that the participant's health on the day of study intervention administration temporarily precludes dosing, the visit will be rescheduled. Refer to Section 5.5 for the list of criteria for temporary delay of study intervention administration. Treatment of any abnormality observed during this examination has to be performed according to local medical practice outside this study or by referral to an appropriate health care provider.

Collected information needs to be recorded in the eCRF.

A targeted physical examination at each study visit after the first study intervention administration will be performed if part of the SoC or only if the participant/participant's parent(s)/LAR(s) indicates during questioning that there might be some underlying pathology(ies) or if deemed necessary by the investigator or delegate.

8.1.6. Vital signs

At minimum, temperature, vital signs (e.g., heart rate, respiratory rate, and blood pressure) will be recorded.

Vital signs are to be taken after at least 10 minutes of rest and before blood collection for laboratory tests (if applicable) and will consist of systolic/diastolic blood pressure, heart rate and respiratory rate by counting the number of breaths for 1 minute before vaccination at Visit 1 and Visit 3.

Blood pressure and pulse measurements will be assessed with a completely automated device. Manual techniques will be used only if an automated device is not available.

In case of increased blood pressure (i.e., systolic BP ≥ 140 mm Hg, and /or diastolic BP ≥90 mm Hg) the measurements of 3 consecutive blood pressure readings will be recorded at intervals of at least 1 minute. The average of the 3 blood pressure readings will be recorded [Smith, 2005].

Collected information needs to be recorded in the eCRF.

If the investigator determines that the participant's health on the day of study intervention administration temporarily precludes dosing, the visit will be rescheduled. Vital signs will be re-taken in case of delayed vaccination. Refer to the Section 5.5 for the list of criteria for temporary delay of study intervention administration.

The body temperature of each participant needs to be measured prior to study intervention administration and recorded in the eCRF. The location for measuring temperature will be oral or axillary. If the participant has fever (defined as temperature ≥38.0°C/100.4°F regardless of the location of measurement) on the day of study intervention administration, the visit will be rescheduled (refer to Section 5.5 for details).

8.1.7. Allograft rejection

Participants will be instructed to contact the study site or their health care provider if they develop signs or symptoms perceived as allograft dysfunction, rejection, or failure, as explained by their transplant physician(s).

Participants, after notifying the study site of possible allograft dysfunction, rejection, or failure, will undergo the study site's standard clinical procedures for the diagnosis of possible rejection. These allograft diagnostic tests, including any biopsies, will be considered "clinically indicated".

In addition to clinically indicated testing and as part of the management of the transplant, allograft function testing and biopsies may be routinely performed, as per center-defined protocol and timetable for surveillance. These tests will be considered as performed per "surveillance protocol" and recorded into eCRF screen.

Allograft rejection will be recorded as an AESI in the Expedited AEs Report. The following information on allograft rejection is to be included in eCRF screens:

- Start date as based on:
  - Date of first appearance of clinical symptoms indicative of rejection, resulting in a "clinically indicated" biopsy or
  - Date of "surveillance protocol" biopsy.
- Pertinent medical evaluations with results, including:
  - Allograft biopsy - date, histological results
  - Clinical laboratories
  - Imaging
- Medical management including but not limited to:
  - procedures
  - changes in immunosuppressive medications
- End date of the rejection event.
- Outcome: not recovered/not resolved (ongoing, stabilized, progressing [e.g., allograft rejection progressing to failure]), recovering/resolving/disappearing, recovered/resolved.

8.1.7.1. Record estimated glomerular filtration rate (eGFR) and proteinuria/albuminuria (only in RTx patients)

Local laboratory ranges on eGFR and proteinuria/albuminuria including equations used to calculate eGFR or conversions used for proteinuria/albuminuria should be provided to the sponsor prior to study start.

eGFR and proteinuria/albuminuria measures (as obtained per SoC "surveillance protocols" and/or for "clinical indications") will be recorded:

- Record all available eGFR from SoC, as of screening visit, so that at least 2 measurements are recorded prior to the first study intervention administration, and continue to record during the entire study and,
- Record all available results on proteinuria/albuminuria from SoC, starting from the day of first study intervention administration, backward so that at least 2 measurements are recorded prior to the first study intervention administration and continue to record during the entire study.

The eGFR results will be collected as well as their corresponding dates and their interpretation (staging), see Section 10.5.1.

The proteinuria/albuminuria results will be collected in mg/g units as well as their corresponding dates and their interpretation (staging), see Section 10.5.2.

8.1.7.2. Record bronchoscopy and/or transbronchial lung biopsy (TBBx) (only in LTx patients)

In some countries bronchoscopy is the SoC test to assess possible signs of rejection in LTx patients. In case the bronchoscopy results (measures as obtained per SoC "surveillance protocols" and/or for "clinical indications") are available, they will be recorded. If TBBx have been requested by SoC or by indication, the results will be also recorded.

The results of bronchoscopy will be collected as well as their corresponding dates and their interpretation (classification), see Sections 10.5.3 and 10.5.4.

8.1.7.3. Record FEV1 (only in LTx patients)

In LTx patients, the FEV1 results (measures as obtained at site per SoC "surveillance protocols" and/or for "clinical indications") will be recorded as well as their interpretation in staging the CLAD. The FEV1 results will be collected as well as their corresponding dates and their interpretation (classification), see Section 10.5.5.

8.1.7.4. Imaging results (RTx and LTx patients)

In some countries, imaging such as CT-Scan, MRI, X-Ray, ultrasound or other, is a SoC test to assess possible signs of rejection in Tx patients. In case the imaging results (measured as obtained per SoC "surveillance protocols" and/or for "clinical indications") are available, they will be recorded in the eCRF, including their timestamp and summary of the results.

8.1.7.5. Biopsy results (RTx and LTx patients)

In case biopsies are taken to assess the possible onset of rejection (either acute or chronic), a summary of the results as well as indication of showing rejection, will be recorded in the eCRF.

8.2. Immunogenicity assessments

Biological samples will be used for research planned in the protocol and for purposes related to the improvement, development and quality assurance of the laboratory tests described in this protocol.

Findings in this or future studies may make it desirable to use samples acquired in this study for research not planned in this protocol. In this case, all participants in countries where this is allowed will be asked to give consent to allow GSK or a contracted partner, to use the samples for further research. The further research will be subject to prior IEC/IRB approval, if required by local legislation.

Information on further research and its rationale can be obtained from GSK.

Sample testing will be done in accordance with the recorded consent of the individual participant/participant's parent(s)/LAR(s).

By default, collected samples will be stored for a maximum of 20 years. This storage period begins when the last participant performs the last study visit. This timeline can be adapted based on local laws, regulations or guidelines requiring different timeframes or procedures. In all cases, the storage period should be aligned with participant's consent. These additional requirements must be formally communicated to, discussed and agreed with GSK.

Planned timepoints for all immunogenicity assessments are provided in the SoA (Section 1.3).

8.2.1. Biological samples

An overall maximal volume of 288 mL of blood will be collected per participant during the entire study period (12-15 months). Refer to Table 12 and SoA (Section 1.3) for information on volumes collected for different assessments.

Table 12 Biological samples

Sample type	Quantity	Unit	Timepoint	Subset name
**Blood for Hematology and Biochemistry**	~6	mL	Screening Visit	IC population
**Blood for HI (neutralizing titers against RSV-A, RSV-B)**	~8.5	mL	Visit 1<br>Visit 2*<br>Visit 3<br>Visit 4<br>Visit 5	All Participants

Sample type	Quantity	Unit	Timepoint	Subset name
Blood for Hematology and Biochemistry	~6	mL	Screening Visit	IC population
			Visit 6	
Blood for CMI	~30	mL	Visit 1<br>Visit 2<br>Visit 3<br>Visit 4<br>Visit 5<br>Visit 6	CMI subset
CCI [redacted]				
Urine for pregnancy test	-	-	Screening Visit**<br>Visit 1<br>Visit 3***	WOCBP

CMI = Cell-mediated immunity; HI = Humoral Immunity; IC = Immunocompromised; CCI [redacted]; RSV = Respiratory Syncytial Virus. WOCBP = Women of Childbearing Potential

* Only for CMI subset (Refer Section 6.3.4)

** Only for IC population.

*** Only for IC-2 dose group.

8.2.2. Laboratory assays

Table 13 Laboratory assays

Test Classification	System	Component	Challenge	Method	Laboratory
**CMI**	PBMC	CD4+ and CD8+ T cells expressing immune or activation markers among CD40L, 4-1BB, IL-2, TNF-α, IFN-γ, IL-13, and IL-17	Peptide pool covering the RSVPreF3 protein sequence	ICS	GSK*
**Humoral immunity (antibody determination)**	Serum	RSV-A neutralizing titers		Neutralization	GSK*
		RSV-B neutralizing titers			
CCI [redacted]					

Test Classification	System	Component	Challenge	Method	Laboratory
**Hematology**	Blood	Leukocytes (white blood cells)<br>Neutrophils<br>Lymphocytes<br>Basophils<br>Monocytes<br>Eosinophils<br>Hemoglobin<br>Platelets<br>Erythrocytes (red blood cells)		As per central laboratory procedure	Central Lab
**Biochemistry**	Serum	Alanine Aminotransferase (ALT)<br>Aspartate Aminotransferase (AST)<br>Creatinine<br>eGFR (Refer 10.5.1 for definition)<br>Blood Urea Nitrogen		As per central laboratory procedure	Central Lab

CD = Cluster of differentiation; CD40L = CD40 ligand; CMI = Cell-mediated immunity; [redacted]; eGFR = estimated glomerular filtration rate; GSK = GlaxoSmithKline Biologicals S.A.; [redacted]; ICS = Intracellular cytokine staining; IFN = Interferon; IL = Interleukin; [redacted]; PBMC = Peripheral blood mononuclear cell; RSV = Respiratory Syncytial Virus; TNF = Tumor necrosis factor.

* GSK laboratory refers to the clinical laboratory sciences (CLS) in Rixensart, Belgium; Wavre, Belgium. CLS may delegate testing to GSK Research laboratories in Rixensart, Belgium; Rockville, USA; Siena, Italy or to a contracted CRO.

Please refer to Section 10.2 for a brief description of the assays performed in the study.

The addresses of clinical laboratories used for sample analysis are provided in a separate document accompanying this study protocol.

GSK clinical laboratories have established a Quality System supported by procedures. The activities of GSK clinical laboratories are audited regularly for quality assessment by an internal (sponsor-dependent) but laboratory-independent Quality Department.

8.2.3. Immunological read-outs

Table 14 Immunological read-outs

Blood sampling time point		Subset name	No. of participants	Component
**Type of contact and time point**	**Sampling time point**			
Humoral immunity (on serum samples)				
Visit 1 (Day 1)	Pre-dose-1	All participants	375	RSV-A neutralization<br>RSV-B neutralization
Visit 2 (V1 + 7-14 days)	Post-dose-1	CMI subset	114	RSV-A neutralization<br>RSV-B neutralization
Visit 3 (V1 + 30-60 days)	Post-dose-1	All participants	375	RSV-A neutralization<br>RSV-B neutralization
Visit 4 (V3 + 30-42 days)	Post-dose-1/-2	All participants	375	RSV-A neutralization<br>RSV-B neutralization
Visit 5 (V1/V3 + 180-210 days)	Post-dose-1/-2	All participants	375	RSV-A neutralization<br>RSV-B neutralization
Visit 6 (V1/V3 + 350-380 days)	Post-dose-1/-2	All participants	375	RSV-A neutralization<br>RSV-B neutralization

Blood sampling time point		Subset name	No. of participants	Component
Type of contact and time point	Sampling time point			
CMI (on PBMC samples)				
Visit 1 (Day 1)	Pre-dose-1	CMI subset	114	CD4+ and CD8+ T cells expressing immune or activation markers among CD40L, 4-1BB, IL-2, TNF-α, IFN-γ, IL-13, and IL-17
Visit 2 (V1 + 7-14 days)	Post-dose-1	CMI subset	114	CD4+ and CD8+ T cells expressing immune or activation markers among CD40L, 4-1BB, IL-2, TNF-α, IFN-γ, IL-13, and IL-17
Visit 3 (V1 + 30-60 days)	Post-dose-1	CMI subset	114	CD4+ and CD8+ T cells expressing immune or activation markers among CD40L, 4-1BB, IL-2, TNF-α, IFN-γ, IL-13, and IL-17
Visit 4 (V3 + 30-42 days)	Post-dose-1/-2	CMI subset	114	CD4+ and CD8+ T cells expressing immune or activation markers among CD40L, 4-1BB, IL-2, TNF-α, IFN-γ, IL-13, and IL-17
Visit 5 (V1/V3 + 180-210 days)	Post-dose-1/-2	CMI subset	114	CD4+ and CD8+ T cells expressing immune or activation markers among CD40L, 4-1BB, IL-2, TNF-α, IFN-γ, IL-13, and IL-17
Visit 6 (V1/V3 + 350-380 days)	Post-dose-1/-2	CMI subset	114	CD4+ and CD8+ T cells expressing immune or activation markers among CD40L, 4-1BB, IL-2, TNF-α, IFN-γ, IL-13, and IL-17

CD = Cluster of differentiation; CD40L = CD40 ligand; CMI = Cell-mediated immunity; IFN = Interferon; IL = Interleukin; RSV = Respiratory Syncytial Virus; TNF = Tumor necrosis factor.

8.2.4. Cytology

Not applicable.

8.2.5. Immunological correlates of protection

No generally accepted immunological correlate of protection has been demonstrated so far for the antigen used in the RSVPreF3 OA investigational vaccine.

8.3. Safety assessments

Planned timepoints for all safety assessments are provided in the SoA (Section 1.3).

8.3.1. Clinical safety laboratory tests

See Section 10.2 for the list of clinical laboratory tests to be performed in accordance with lab manual and the SoA (Section 1.3).

The investigator must review the laboratory results from screening and document this review. The laboratory results must be retained with source documents.

Abnormal laboratory findings associated with the underlying disease at screening are not considered clinically significant unless judged by the investigator to be more severe than expected for the participant's condition. In this case, the SOT patient can not be included in the study.

CCI [redacted]

8.3.3. Pregnancy testing

A urine pregnancy test must be performed for female participants of childbearing potential before the administration of any dose of study intervention. Pregnancy testing must be done even if the participant is menstruating at the time of the study visit. The study intervention may only be administered if the pregnancy test is negative.

Refer to Section 8.4.6 for the information on study continuation for participants who become pregnant during the study.

8.3.4. Safety monitoring and Committee

Participant safety will be continuously monitored by the Medical monitor and the designated Safety Lead (or delegate), Safety Review Team (SRT) and IDMC throughout the study. Pertinent findings and conclusions are shared with the product's SRT for review of the overall benefit-risk profile of the product.

This study will include lung and renal transplant recipient patients which are a special population within the group of IC patients because of the specific risk of organ rejection. Therefore, an IDMC which includes clinicians with expertise in lung and renal transplant will review safety data and provide recommendations to the team. The IDMC charter will describe the timing and the frequency of the meetings, the required data review and documentation of the safety evaluations.

Safety data will be summarized and reviewed by the IDMC, according to the IDMC charter, for agreement of next steps.

8.4. Adverse events (AEs), serious adverse events (SAEs), and other safety reporting

For definitions relating to safety information see Section 10.3.

The investigator and any qualified designees are responsible for detecting, documenting, and recording events that meet the definition of an AE or SAE and other safety information and remain responsible for following up all AEs. This includes events reported by the participant (or, when appropriate, by a caregiver).

Investigator safety reports must be prepared for suspected unexpected serious adverse reactions (SUSARs) according to local regulatory requirements and sponsor policy and forwarded to investigators as necessary.

The method of recording, evaluating, and assessing causality of AEs and SAEs and the procedures for completing and transmitting SAE reports are provided in Section 10.3.

8.4.1. Time period and frequency for collecting AE, SAE, and other safety information

All AEs and SAEs will be collected at the time points specified in the SoA (Section 1.3).

AF reporting will follow the same reporting periods as for AEs and SAEs. Non-serious AF with an onset during the 30-day period following each study vaccine administration will be collected. The reporting of AF meeting the SAE definition (serious AF) will be performed according to the SAE reporting period.

SAEs assessed as related to study participation (e.g., protocol-mandated procedures, invasive tests, or change in existing therapy) or related to a GSK product (non-IMP) will be recorded from the time a participant consents to participate in the study.

Medical occurrences that begin before the start of study intervention but after obtaining informed consent will be recorded as medical history/current medical conditions, not as AEs.

All SAEs will be recorded and reported to the sponsor or designee immediately and under no circumstance should this exceed 24 hours, as indicated in Section 10.3. The investigator will submit any updated SAE data to the sponsor within 24 hours of it being available. A post-study AE/SAE is defined as any event that occurs outside of the AE/SAE reporting period defined in Section 8.4.1/ Table 15 and Table 16.

Table 15 Timeframes for collecting and reporting of safety information in IC 1-dose group and healthy participant group

Type of contact	Screening Visit (for IC population only)*	Visit 1		Visit 2**	Visit 3	Visit 4	Visit 5	Visit 6
Allowed interval	-30 to -7 days	Day 1 (pre-vaccination) *	Day 1	Visit 1 + 7-14 days	Visit 1 + 30-60 days	Visit 3 + 30-42 days	Visit 1 + 180-210 days	Visit 1 + 350-380 days
Solicited administration site and systemic events (within 7 days after study intervention administration)								
Unsolicited AEs (within 30 days after study intervention administration)^								
SAEs related to study participation*** or concurrent GSK medication/vaccine								
All SAEs^, all pIMDs, pregnancies, and (S)AEs leading to withdrawal from the study								
Intercurrent medical conditions								
AESIs specific for renal/lung transplant^^								

AE = Adverse event; AESI = Adverse event of special interest; AF = Atrial fibrillation; IC = Immunocompromised; pIMD = Potential immune-mediated disease; SAE = Serious adverse event.

* Corresponds to the day when informed consent is obtained (Day 1, prior to study intervention administration in healthy participants, and at screening visit in IC population).

** Visit 2 is planned in the CMI subset of the study population (30% of the group population i.e., 38 participants in each group).

*** Any SAEs assessed as related to study participation (e.g., protocol-mandated procedures, invasive tests such as blood sampling) or related to a GSK product will be recorded from the time a participant consents to participate in the study.

^ AF will be considered as AESI in this study and will be additionally reported in the AF follow-up questionnaire in eCRF. The reporting of non-serious AF will be performed according to the unsolicited AE reporting period. The reporting of AF meeting the SAE definition (serious AF) will be performed according to the SAE reporting period. Fatal AF and Serious AF judged as related to study vaccination will be reported according to the fatal SAE and related SAE reporting period, respectively.

^^ Corresponds only to the IC population. These AESIs include signs for allograft rejection.

The shaded regions in the table indicate time period of data collection.

Table 16 Timeframes for collecting and reporting of safety information in IC 2-dose group

**Type of contact**	**Screening Visit***	**Visit 1**		**Visit 2****	**Visit 3**	**Visit 4**	**Visit 5**	**Visit 6**
**Allowed interval**	**-30 to -7 days**	**Day 1 (pre-vaccination)**	**Day 1**	**Visit 1 + 7-14 days**	**Visit 1 + 30-60 days**	**Visit 3 + 30-42 days**	**Visit 3 + 180-210 days**	**Visit 3 + 350-380 days**
Solicited administration site and systemic events (within 7 days after each study intervention administration)								
Unsolicited AEs (within 30 days after each study intervention administration)^								
SAEs related to study participation*** or concurrent GSK medication/vaccine								
All SAEs^, all pIMDs, pregnancies, and (S)AEs leading to withdrawal from the study								
Intercurrent medical conditions								
AESIs specific for renal/lung transplant^^								

AE = Adverse event; AESI = Adverse event of special interest; AF = Atrial fibrillation; IC = Immunocompromised; pIMD = Potential immune-mediated disease; SAE = Serious adverse event.

* Corresponds to the day when informed consent is obtained.

** Visit 2 is planned in the CMI subset of the study population (30% of the group population i.e., 38 participants).

*** Any SAEs assessed as related to study participation (e.g., protocol-mandated procedures, invasive tests such as blood sampling) or related to a GSK product will be recorded from the time a participant consents to participate in the study.

^ AF will be considered as AESI in this study and will be additionally reported in the AF follow-up questionnaire in eCRF. The reporting of non-serious AF will be performed according to the unsolicited AE reporting period. The reporting of AF meeting the SAE definition (serious AF) will be performed according to the SAE reporting period. Fatal AF and Serious AF judged as related to study vaccination will be reported according to the fatal SAE and related SAE reporting period, respectively.

^^ These AESIs include signs for allograft rejection.

The shaded regions in the table indicate time period of data collection.

Investigators are not obligated to actively seek information on AEs or SAEs after conclusion of the study participation. However, if the investigator learns of any SAE, including a death, after a participant has been discharged from the study, the investigator must record it in the medical records. If the investigator considers the event to be reasonably related to the study intervention or study participation, the investigator must promptly notify the sponsor.

8.4.2. Method of detecting AEs and SAEs

Care will be taken not to introduce bias when detecting AEs and SAEs. Open-ended and nonleading verbal questioning of the participant/participant's parent(s)/LAR(s) is the preferred method to inquire about AE occurrences.

8.4.3. Follow-up of AEs and SAEs

After the initial AE/SAE report, the investigator is required to proactively follow each participant at subsequent visits/contacts. All SAEs and AESIs (as defined in Section 8.4.4) will be followed until resolution, stabilization, the event is otherwise explained, or the participant is lost to follow-up (as defined in Section 7.3). For AF cases, the investigator will provide any new or updated relevant information on previously reported AF during the study to GSK using a paper/electronic Expedited AEs Report and the AF follow-up questionnaire as applicable. Further information on follow-up procedures is provided in Section 10.3.5.5.

8.4.4. AESIs

8.4.4.1. Potential immune-mediated diseases

Potential immune-mediated diseases (pIMDs) are a subset of AESIs that include autoimmune diseases and other inflammatory and/or neurologic disorders of interest which may or may not have an autoimmune etiology. AEs that need to be recorded and reported as pIMDs include those listed in Table 17.

In order to facilitate the documentation of pIMDs in the eCRF, a pIMD standard questionnaire and a list of preferred terms (PTs) and PT codes corresponding to the above diagnoses will be available to investigators at study start.

The investigator(s) must exercise their medical/scientific judgment to determine whether other diseases have an autoimmune origin (i.e., pathophysiology involving systemic or organ-specific pathogenic autoantibodies) and should also be recorded as a pIMD. In addition, the investigator should categorize each pIMD either as a new onset condition (if it started following vaccination) or as an exacerbation of a pre-existing chronic condition (if it exacerbated following vaccination) in the eCRF.

Table 17 List of potential immune-mediated diseases (pIMDs)

Medical Concept	Additional Notes
**Blood disorders and coagulopathies**	
Antiphospholipid syndrome	
Autoimmune aplastic anemia	
Autoimmune hemolytic anemia	• Includes warm antibody hemolytic anemia and cold antibody hemolytic anemia
Autoimmune lymphoproliferative syndrome (ALPS)	
Autoimmune neutropenia	
Autoimmune pancytopenia	
Autoimmune thrombocytopenia	• Frequently used related terms include: “autoimmune thrombocytopenic purpura”, “idiopathic thrombocytopenic purpura (ITP)”, “idiopathic immune thrombocytopenia”, “primary immune thrombocytopenia”.
Evans syndrome	
Pernicious anemia	
Thrombosis with thrombocytopenia syndrome (TTS)	
Thrombotic thrombocytopenic purpura	• Also known as “Moschcowitz-syndrome” or “microangiopathic hemolytic anemia”
**Cardio-pulmonary inflammatory disorders**	
Idiopathic Myocarditis/Pericarditis	Including but not limited to:<br>• Autoimmune / Immune-mediated myocarditis<br>• Autoimmune / Immune-mediated pericarditis<br>• Giant cell myocarditis
Idiopathic pulmonary fibrosis	Including but not limited to:<br>• Idiopathic interstitial pneumonia (frequently used related terms include “Interstitial lung disease”, “Pulmonary fibrosis”, “Immune-mediated pneumonitis”)<br>• Pleuroparenchymal fibroelastosis (PPFE)
Pulmonary alveolar proteinosis (PAP)	• Frequently used related terms include: “pulmonary alveolar lipoproteinosis”, “phospholipidosis”
**Endocrine disorders**	
Addison’s disease	
Autoimmune / Immune-mediated thyroiditis	Including but not limited to:<br>• Hashimoto thyroiditis (autoimmune hypothyroidism, lymphocytic thyroiditis)<br>• Atrophic thyroiditis<br>• Silent thyroiditis<br>• Thyrotoxicosis
Autoimmune diseases of the testis and ovary	• Includes autoimmune oophoritis, autoimmune ovarian failure and autoimmune orchitis
Autoimmune hyperlipidemia	
Autoimmune hypophysitis	
Diabetes mellitus type I	
Grave's or Basedow’s disease	• Includes Marine Lenhart syndrome and Graves’ ophthalmopathy, also known as thyroid eye disease (TED) or endocrine ophthalmopathy
Insulin autoimmune syndrome	
Polyglandular autoimmune syndrome	• Includes Polyglandular autoimmune syndrome type I, II and III

Medical Concept	Additional Notes
**Eye disorders**	
Ocular Autoimmune / Immune-mediated disorders	Including but not limited to:<br>• Acute macular neuroretinopathy (also known as acute macular outer retinopathy)<br>• Autoimmune / Immune-mediated retinopathy<br>• Autoimmune / Immune-mediated uveitis, including idiopathic uveitis and sympathetic ophthalmia<br>• Cogan's syndrome: an oculo-audiovestibular disease<br>• Ocular pemphigoid<br>• Ulcerative keratitis<br>• Vogt-Koyanagi-Harada disease
**Gastrointestinal disorders**	
Autoimmune / Immune-mediated pancreatitis	
Celiac disease	
Inflammatory Bowel disease	Including but not limited to:<br>• Crohn's disease<br>• Microscopic colitis<br>• Terminal ileitis<br>• Ulcerative colitis<br>• Ulcerative proctitis
**Hepatobiliary disorders**	
Autoimmune cholangitis	
Autoimmune hepatitis	
Primary biliary cirrhosis	
Primary sclerosing cholangitis	
**Musculoskeletal and connective tissue disorders**	
Gout	• Includes gouty arthritis
Idiopathic inflammatory myopathies	Including but not limited to:<br>• Dermatomyositis<br>• Inclusion body myositis<br>• Immune-mediated necrotizing myopathy<br>• Polymyositis
Mixed connective tissue disorder	
Polymyalgia rheumatica (PMR)	
Psoriatic arthritis (PsA)	
Relapsing polychondritis	
Rheumatoid arthritis	Including but not limited to:<br>• Rheumatoid arthritis associated conditions<br>• Juvenile idiopathic arthritis<br>• Palindromic rheumatism<br>• Still's disease<br>• Felty's syndrome
Sjögren's syndrome	
Spondyloarthritis	Including but not limited to:<br>• Ankylosing spondylitis<br>• Juvenile spondyloarthritis<br>• Keratoderma blenorrhagica<br>• Psoriatic spondylitis<br>• Reactive Arthritis (Reiter's Syndrome)<br>• Undifferentiated spondyloarthritis

Medical Concept	Additional Notes
Systemic lupus Erythematosus	• Includes Lupus associated conditions (e.g., Cutaneous lupus erythematosus, Lupus nephritis, etc.) or complications such as shrinking lung syndrome (SLS)
Systemic Scleroderma (systemic sclerosis)	• Includes Reynolds syndrome (RS), systemic sclerosis with diffuse scleroderma and systemic sclerosis with limited scleroderma (also known as CREST syndrome)
Neuroinflammatory/neuromuscular disorders	
Acute disseminated encephalomyelitis (ADEM) and other inflammatory demyelinating variants	Includes the following:<br>• Acute necrotizing myelitis<br>• Bickerstaff's brainstem encephalitis<br>• Disseminated necrotizing leukoencephalopathy (also known as Weston-Hurst syndrome, acute hemorrhagic leuko-encephalitis, or acute necrotizing hemorrhagic encephalomyelitis)<br>• Myelin oligodendrocyte glycoprotein antibody-associated disease<br>• Neuromyelitis optica (also known as Devic's disease)<br>• Noninfective encephalitis / encephalomyelitis / myelitis<br>• Postimmunization encephalomyelitis
Guillain-Barré syndrome (GBS)	• Includes variants such as Miller Fisher syndrome and the acute motor and sensory axonal neuropathy (AMSAN)
Idiopathic cranial nerve palsies/paresis and inflammations (neuritis)	Including but not limited to:<br>• Cranial nerve neuritis (e.g., Optic neuritis)<br>• Idiopathic nerve palsies/paresis (e.g., Bell's palsy)<br>• Melkersson-Rosenthal syndrome<br>• Multiple cranial nerve palsies/paresis
Multiple sclerosis (MS)	Includes the following:<br>• Clinically isolated syndrome (CIS)<br>• Malignant MS (the Marburg type of MS)<br>• Primary-progressive MS (PPMS)<br>• Radiologically isolated syndrome (RIS)<br>• Relapsing-remitting MS (RRMS)<br>• Secondary-progressive MS (SPMS)<br>• Uhthoff's phenomenon
Myasthenia gravis	• Includes ocular myasthenia and Lambert-Eaton myasthenic syndrome
Narcolepsy	• Includes narcolepsy with or without presence of unambiguous cataplexy
Peripheral inflammatory demyelinating neuropathies and plexopathies	Including but not limited to:<br>• Acute Brachial Radiculitis (also known as Parsonage-Turner Syndrome or neuralgic amyotrophy)<br>• Antibody-mediated demyelinating neuropathy<br>• Chronic idiopathic axonal polyneuropathy (CIAP)<br>• Chronic Inflammatory Demyelinating Polyradiculoneuropathy (CIDP), including atypical CIDP variants (e.g., multifocal acquired demyelinating sensory and motor neuropathy also known as Lewis-Sumner syndrome)<br>• Multifocal motor neuropathy (MMN)
Transverse myelitis I	• Includes acute partial transverse myelitis (APTM) and acute complete transverse myelitis (ACTM)

Medical Concept	Additional Notes
**Renal disorders**	
Autoimmune / immune-mediated glomerulonephritis	Including but not limited to:<br>• IgA nephropathy<br>• IgM nephropathy<br>• C1q nephropathy<br>• Fibrillary glomerulonephritis<br>• Glomerulonephritis rapidly progressive<br>• Membranoproliferative glomerulonephritis<br>• Membranous glomerulonephritis<br>• Mesangioproliferative glomerulonephritis<br>• Tubulointerstitial nephritis and uveitis syndrome
**Skin and subcutaneous tissue disorders**	
Alopecia areata	
Autoimmune / immune-mediated blistering dermatoses	Including but not limited to:<br>• Bullous Dermatitis<br>• Bullous Pemphigoid<br>• Dermatitis herpetiformis<br>• Epidermolysis bullosa acquisita (EBA)<br>• Linear IgA-mediated bullous dermatosis (LABD), also known as Linear IgA disease<br>• Pemphigus
Erythema multiforme	
Erythema nodosum	
Reactive granulomatous dermatitis	Including but not limited to<br>• Interstitial granulomatous dermatitis<br>• Palisaded neutrophilic granulomatous dermatitis
Lichen planus	• Includes liquen planopilaris
Localized Scleroderma (Morphoea)	• Includes Eosinophilic fasciitis (also called Shulman syndrome)
Psoriasis	
Pyoderma gangrenosum	
Stevens-Johnson syndrome (SJS)	Including but not limited to:<br>• Toxic Epidermal Necrolysis (TEN)<br>• SJS-TEN overlap
Sweet's syndrome	• Includes Acute febrile neutrophilic dermatosis
Vitiligo	
**Vasculitis**	
Large vessels vasculitis	Including but not limited to:<br>• Arteritic anterior ischemic optic neuropathy (AAION or arteritic AION)<br>• Giant cell arteritis (also called temporal arteritis)<br>• Taka'asu's arteritis
Medium sized and/or small vessels vasculitis	Including but not limited to:<br>• Anti-neutrophil cytoplasmic antibody (ANCA) positive vasculitis (type unspecified)<br>• Be'cet's syndrome<br>• Buerger's disease (thromboangiitis obliterans)<br>• Churg–Strauss syndrome (allergic granulomatous angiitis)<br>• Erythema induratum (also known as nodular vasculitis)<br>• Henoch-Schonlein purpura (also known as IgA vasculitis)<br>• Microscopic polyangiitis<br>• Necrotizing vasculitis<br>• Polyarteritis nodosa

Medical Concept	Additional Notes
	• Single organ cutaneous vasculitis, including leukocytoclastic vasculitis, hypersensitivity vasculitis and acute hemorrhagic edema of infancy (AHEI)<br>• Wegener's granulomatosis
Other (including multisystemic)	
Anti-synthetase syndrome	
Capillary leak syndrome	• Frequently used related terms include: "systemic capillary leak syndrome (SCLS)" or "Clarkson's Syndrome"
Goodpasture syndrome	• Frequently used related terms include: "pulmonary renal syndrome" and "anti-Glomerular Basement Membrane disease (anti-GBM disease)"
Immune-mediated enhancement of disease	• Includes vaccine associated enhanced disease (VAED and VAERD). Frequently used related terms include "vaccine-mediated enhanced disease (VMED)", "enhanced respiratory disease (ERD)", "vaccine-induced enhancement of infection", "disease enhancement", "immune enhancement", and "antibody-dependent enhancement (ADE)
Immunoglobulin G4 related disease	
Langerhans' cell histiocytosis	
Multisystem inflammatory syndromes	Including but not limited to:<br>• Kawasaki's disease<br>• Multisystem inflammatory syndrome in adults (MIS-A)<br>• Multisystem inflammatory syndrome in children (MIS-C)
Overlap syndrome	
Raynaud's phenomenon	
Sarcoidosis	• Includes Loefgren syndrome
Susac's syndrome	

8.4.4.2. Atrial fibrillation (AF)

AEs of AF are considered as AESI in this study.

In the efficacy study (RSV OA=ADJ-006), at the time of safety analysis (data lock point [DLP]) of 30 April 2022, a numerical imbalance in events of AF was observed within 30 days post-vaccination, with 10 events of AF (among which 7 [0.1%] were serious) in the RSVPreF3 group versus 4 (among which 1 [<0.1%] was serious) in the placebo group. No imbalance was observed for serious events of AF reported within 6 months post-vaccination. To further characterize events of AF, AF will be considered as an AESI.

When there is enough evidence to make the above diagnosis, the AE must be reported as AESI. Symptoms, signs or conditions which might (or might not) represent AF, should be recorded and reported as AEs but not as AESI until the final or definitive diagnosis has been determined, and alternative diagnoses have been eliminated or shown to be less likely.

For each case of AF reported in the AE or SAE section in the eCRF, additional information will be collected in a specific 'AF follow-up questionnaire' eCRF screen.

8.4.4.3. AESI for Transplant patients

Within the framework of the study, transplant rejection that occurs at any time during the study will be considered and recorded as an AESI.

Transplant rejection occurs when transplanted tissue is rejected by the recipient's immune system, which destroys the transplanted tissue. Diagnosis of rejection relies on patient signs and symptoms, laboratory data, imaging or other technical procedures and/or tissue biopsies. The investigator will take into consideration all available information, including biopsy results, to decide whether or not organ rejection occurs.

Clinically, management of allograft rejection will be as per local standard of care.

Any form of rejection should be recorded and reported to the sponsor or designee as indicated in Section 10.3.

When there is enough evidence to make any of the above diagnoses, the AE must be reported as AESI. Symptoms, signs or conditions which might (or might not) lead to one of the above diagnoses, should be recorded and reported as AEs but not as AESIs until the final or definitive diagnosis has been made, and alternative diagnoses eliminated or shown to be less likely.

8.4.5. Regulatory reporting requirements for SAEs/pregnancies/AESIs

Prompt notification by the investigator to the sponsor of an SAE/pregnancy/AESI is essential so that legal obligations and ethical responsibilities toward the safety of participants and the safety of a study intervention under clinical investigation are met. See Section 8.4.1 for reporting timeframes.

For SAEs/AESIs, the investigator must always provide an assessment of causality at the time of the initial report, as defined in Section 10.3.5.6.

An investigator who receives an investigator safety report describing an SAE or other specific safety information (e.g., summary or listing of SAEs) from the sponsor will review and then file it along with the IB and will notify the IRB/IEC, if appropriate according to local requirements.

Table 18 Timeframes for submitting SAEs, pregnancies and AESIs to GSK

Type of event	Initial reports		Follow-up of relevant information on a previous report	
	Timeframe	Documents	Timeframe	Documents
SAEs	24 hours*	electronic AEs Report	24 hours*	electronic AEs Report
Pregnancies	24 hours*	electronic pregnancy report	24 hours*	electronic pregnancy report
AESIs	24 hours**	electronic AEs Report	24 hours*	electronic AEs Report
Serious AF***	24 hours**	electronic AEs Report + AF follow-up questionnaire	24 hours*	electronic AEs Report + AF follow-up questionnaire

* Timeframe allowed after receipt or awareness of the information by the investigator/site staff.
** Timeframe allowed once the investigator determines that the event meets the protocol definition of an AESI.
*** Only AF meeting SAE definition will be reported in electronic AEs Report and in the specific AF follow-up questionnaire. Non-serious AF will be reported in the non-serious adverse event eCRF screen and in the AF follow-up questionnaire.

8.4.6. Pregnancy

Female participants who become pregnant after the first study intervention dose must not receive subsequent doses of the study intervention but may continue other study procedures at the discretion of the investigator.

Female participants who become pregnant after administration of the study intervention may continue other study procedures at the discretion of the investigator.

Details of all pregnancies in female participants will be collected after the start of study intervention and until end of study (refer Section 5.1 for details).

If a pregnancy is reported, the investigator will record pregnancy information on the appropriate form and submit it to the sponsor within 24 hours of learning of the female participant's pregnancy.

Any pregnancy complication or elective termination of a pregnancy for medical reasons will be reported as an AE or SAE.

Abnormal pregnancy outcomes (e.g., spontaneous abortion, fetal death, stillbirth, congenital anomalies, ectopic pregnancy) are considered SAEs and will be reported as such.The participant will be followed to determine the outcome of the pregnancy. The investigator will collect follow-up information on the participant and the neonate and the information will be forwarded to the sponsor. See Table 18 for reporting timeframes.

Any post-study pregnancy-related SAE considered reasonably related to the study intervention by the investigator will be reported to the sponsor as described in Section 8.4.4. While the investigator is not obligated to actively seek this information in former study participants, he or she may learn of an SAE through spontaneous reporting.

Any female participant who becomes pregnant while participating in the study will discontinue study intervention.

8.4.7. Contact information for reporting SAEs, AESIs and pregnancies

Table 19 Contact information for reporting SAEs, AESIs and pregnancies

**Study contact for questions regarding SAEs, AESIs, pregnancies and SAEs linked to device deficiencies**<br>Contact GSK's local and/or medical contacts
**Contacts for reporting SAEs, AESIs, pregnancies and SAEs linked to device deficiencies**<br>Available 24/24 hours and 7/7 days<br>ogm28723@gsk.com

8.4.8. Participant card

The investigator (or designee) must provide the participant/participant's parent(s)/LAR(s) with a "participant card" containing information about the clinical study. The participant/participant's parent(s)/LAR(s) must be instructed to always keep the participant card in their possession for the duration of the study. In an emergency, this card serves to inform the responsible attending physician/LAR/caregiver that the participant is in a clinical study and that relevant information may be obtained by contacting the investigator(s) or their back up.

8.5. Pharmacokinetics

PK is not evaluated in this study.

8.6. Pharmacodynamics

PD is not evaluated in this study.

8.7. Genetics

Genetics are not evaluated in this study.

8.8. Biomarkers

Biomarkers are not evaluated in this study.

8.9. Immunogenicity assessments

Immunogenicity is described in Section 8.2

8.10. Health economics or medical resource utilization and health economics

Not applicable for this study.

9. STATISTICAL CONSIDERATIONS

9.1. Statistical Hypotheses

The primary objective, as outlined in Section 3, will be addressed using an estimation approach with no hypothesis testing. For the primary estimand, descriptive analysis will be utilized.

9.2. Analysis sets

Analysis sets are presented in Table 20.

Table 20 Analysis sets

Analysis Set	Definition / Criteria	Analyses Evaluated
**Screened Set**	All participants who were screened for eligibility.	Study Population
**Enrolled Set**	All participants who entered the study (who were randomized or received study intervention administration or underwent a post-screening study procedure).<br>NOTE: screening failures (who never passed screening even if rescreened) and participants screened (met eligibility) but never enrolled into the study are excluded from the Enrolled Set as they did not enter the study.	Study Population
**Exposed Set (ES)**	All participants who received the study intervention administration. Analysis per group is based on medical condition (IC versus healthy adults) and according to the randomization (RSV_IC_1 versus RSV_IC_2 group)..	Study Population, Safety
**Per Protocol Set* (PPS)**	All eligible participants<br>• who received the study intervention administration as per protocol<br>• had immunogenicity results pre- and post-dose<br>• complied with blood draw intervals<br>• without intercurrent conditions that may interfere with immunogenicity<br>• without prohibited concomitant medication/vaccination.<br>Analysis per group is based on medical condition (IC versus healthy adults) and according to the randomization (RSV_IC_1 versus RSV_IC_2 group).	Immunogenicity

* Contribution of participants to PPS will be defined by timepoint.

9.2.1. Criteria for elimination from analysis

If the participant meets one of the criteria mentioned below or ones listed in the Section 7.1.1 (contraindication to subsequent vaccination) or 5.2.1 (medical conditions) or 5.2.2 (concomitant therapy), he/she may be eliminated from per protocol analysis.

In case of administration of any medication mentioned in Section 5.2.2 during the study, this will be considered as a protocol deviation and the participant may be eliminated from the PPS.

Participants may be eliminated from the PPS for immunogenicity if, during the study, they incur a condition that has the capability of altering their immune response (intercurrent medical condition) or are confirmed to have an alteration of their initial immune status. Refer to Definition of Terms for the definition of intercurrent medical conditions.

9.3. Statistical analyses

This section is a summary of the planned statistical analyses of the primary and secondary endpoints. Supportive analyses and demography summaries will be described in the SAP.

9.3.1. Primary endpoints/estimands analyses

The co-primary endpoints/estimands are described in Section 3. The primary analysis will be performed on the PPS.

MGI, i.e., the geometric mean of the within-participant ratios, of serum neutralizing titers against RSV-A and RSV-B post-Dose 2 (Visit 4) over post-Dose 1 (Visit 3) will be computed with their 95% CI in the RSV_IC_2 group.

CIs for MGI will be based on a back transformation of CI for the mean of the difference between log10-transformed values, assuming that log-transformed values are normally distributed with unknown variance.

Missing data will not be imputed.

9.3.2. Secondary endpoints/estimands analyses

Refer to Section 3 for the definition of secondary immunogenicity (humoral and CMI response) and safety endpoints. The immunogenicity analysis will be based on the PPS and safety analysis will be based on the ES.

For humoral immune response, the following analysis will be tabulated for each immunological assay (RSV-A and RSV-B neutralization titers):

- Percentage of participants above predefined threshold and GMT, with 95% CI, at pre-study intervention administration (Visit 1), Visit 2 (in a subset of participants), Visit 3, Visit 4, Visit 5, and Visit 6 in all groups.
- GMT ratio (RSV_HA group over RSV_IC group (pooled RSV_IC_1 and RSV_IC_2 group) with 95%CI, at Visit 2 (in a subset of participants) and Visit 3.
- GMT ratio (RSV_IC_2 group over RSV_IC_1 group) with 95% CI, at Visit 4, Visit 5, and Visit 6.
- GMT ratio (RSV_HA group over RSV_IC_1 group) with 95% CI, at Visit 4, Visit 5, and Visit 6.
- GMT ratio (RSV_HA group over RSV_IC_2 group) with 95%CI, at Visit 4, Visit 5, and Visit 6.
- MGI (RSV_IC group [pooled RSV_IC_1 and RSV_IC_2 group] and RSV_HA group) with 95% CI, at Visit 2 (in a subset of participants) over Visit 1, and at Visit 3 over Visit 1.

- MGI (RSV_IC_1, RSV_IC_2 and RSV_HA group) with 95% CI, at Visit 4, Visit 5 and Visit 6 over Visit 1.

The GMT ratios and their 95% CIs will be derived from an ANCOVA model on log10-transformed titers (details will be provided in the SAP).

The following analysis will be tabulated by group for CMI response in the CMI subset:

- Descriptive statistics (N, geometric mean, min, Q1, median, Q3, max) for the frequency of RSVPreF3-specific CD4+ and/or CD8+ T cells expressing at least 2 activation markers including at least one cytokine among CD40L, 4-1BB, IL-2, TNF-α, IFN-γ, IL-13, IL-17 at pre-study intervention administration (Visit 1), Visit 2, Visit 3, Visit 4, Visit 5, and Visit 6 in all groups.

Descriptive safety analysis by group will be summarized as follows:

- Percentage of participants reporting each solicited administration site event and solicited systemic event during a 7-day follow-up period (i.e., on the day of study intervention administration and 6 subsequent days) after each study intervention administration.
- Percentage of participants reporting unsolicited AEs during a 30-day follow-up period (i.e., on the day of study intervention administration and 29 subsequent days) after each study intervention administration by MedDRA Primary System Organ Class (SOC), High Level Term (HLT) and Preferred Term (PT).
- Percentage of participants reporting SAEs from study intervention administration (Day 1) up to study end (Visit 6) by MedDRA Primary SOC, HLT and PT.
- Percentage of participants reporting pIMDs from study intervention administration (Day 1) up to study end (Visit 6) by MedDRA Primary SOC, HLT and PT.
- Percentage of participants reporting SAEs related to study intervention administration from study intervention administration (Day 1) up to study end (Visit 6) by MedDRA Primary SOC, HLT and PT.
- Percentage of participants reporting pIMDs related to study intervention administration from study intervention administration (Day 1) up to study end (Visit 6) by MedDRA Primary SOC, HLT and PT.
- Percentage of participants reporting fatal SAEs from study intervention administration (Day 1) up to study end (Visit 6) by MedDRA Primary SOC, HLT and PT.
- Percentage of participants reporting AESIs (specific to renal and lung SOT patients) from study intervention administration (Day 1) up to study end (Visit 6) by MedDRA Primary SOC, HLT and PT.

- Percentage of participants reporting demyelinating disorder during a 30-day follow-up period (i.e., on the day of study intervention administration and 29 subsequent days) after each study intervention administration by MedDRA Primary SOC, HLT and PT.
- Percentage of participants reporting demyelinating disorder SAE from study intervention administration (Day 1) up to study end (Visit 6) by MedDRA Primary SOC, HLT and PT.

Exact 95% CIs around percentages will be computed using the method of Clopper and Pearson [Clopper, 1934].

9.4. Interim analysis

9.4.1. Sequence of analyses

The following analyses will be performed stepwise:

- **PCA** (**Primary completion analysis)**: A first analysis will be performed on all immunogenicity, reactogenicity and safety data available and as clean as possible, when data for at least primary and secondary endpoints up to Visit 4 are available. This analysis will be considered as final for those endpoints*.
- **Month 6 post-last dose**: A second analysis will be performed on all immunogenicity and safety data available and as clean as possible, when data for at least secondary endpoints up to Visit 5 are available. This analysis will be considered as final for those endpoints*.
- An **end of study** analysis will be performed when all data for at least secondary endpoints up to study conclusion (Visit 6) will be available.

  ** Each analysis can be considered as final, as it is based on data that is as clean as possible. However, the consecutive analysis of the same time point might slightly differ at the next analysis e.g., when Month 6 data are re-analyzed at the time of Month 12 analysis. If major changes are identified, they will be described in the clinical study report.*

9.4.2. Statistical considerations for interim analysis

This section is not applicable.

9.5. Pre-dose sample size determination

Approximately 375 eligible participants will be enrolled in the study, under the following groups:

- Approximately 250 IC adults ≥18 YoA who received a renal or lung SOT, will be randomly assigned (1:1) to the following 2 IC groups (1 dose versus 2 doses; 125 participants each) at Visit 1 (Day 1). Each group may include approximately

65% renal transplant patients and approximately 20% lung transplant patients, and the remaining patients can be freely distributed across the 2 groups.

- **RSV_IC_1 group**: N = 125; IC patients receiving 1 dose of vaccine at Visit 1.
- **RSV_IC_2 group**: N = 125; IC patients receiving 2 doses of vaccine; first dose at Visit 1 and second dose at Visit 3.

- Approximately 125 healthy adults ≥50 YoA (distributed among age categories of 50-59 YoA (30%), 60-69 YoA (30%), ≥70 YoA (20%), and 20% to be freely distributed over the different age categories) will be assigned to the healthy adults group.
  - **RSV_HA group**: N = 125; healthy adults ≥50 YoA receiving 1 dose of vaccine at Visit 1.

A 20% attrition rate is accounted from enrolled to evaluable participants.

9.5.1. Sample size justification

The study is aimed at describing the immune responses and exploring potential differences, between the immune response of IC patients (either after 1 or 2 doses of RSV vaccine) and the immune response of healthy participants. Given the descriptive nature of the study, no multiplicity adjustment is implemented. The sample size for this descriptive study is determined based on the rationale of adequately detecting relevant differences between immune responses:

- **Effect of the second vaccination in IC patients (within-participant comparison)**

In IC patients receiving 2 doses of RSV vaccine, this is evaluated by comparing the response post-Dose 2 (Visit 4) and post-Dose 1(Visit 3). The power and fold-increase that can be detected with a sample size of 100 evaluable participants are shown in Table 21 below.

Table 21 Estimate of the fold-increase that can be detected by power

SD	Power	Fold-increase
0.5	80%	1.39
0.5	90%	1.46
0.4	80%	1.30
0.4	90%	1.35

Paired T-test: alpha (1-sided) = 2.5%, SD of differences = 0.5 (based on RSV OA=ADJ-002 data, 120 ug/AS01E group, V3 vs V6, SD difference neutra RSV-A = 0.23, SD difference neutra RSV-B = 0.40 inflated from 0.4 to 0.5 to account for IC condition).

- **Difference in the humoral immune response of IC patients (1 and 2 doses) vs healthy participants (1 dose)**

One hundred evaluable participants in each group achieve 80% power to detect actual GMT fold differences of ≥1.55 at 1-sided α = 2.5% and 1:1 allocation ratio, assuming standard deviation of antibody log10-titers of 0.45 and 0.50 in the healthy and IC groups respectively (Two-sample T-test).

In addition, pooling of IC groups after 1 dose (200 evaluable participants) allows to detect actual GMT fold differences of ≥1.45 with 80% power compared to healthy participants (100 evaluable participants). Two-sample T-test allowing unequal variance: alpha (1-sided) = 2.5%, SD = 0.45 (healthy), 0.5 (IC).

10. SUPPORTING DOCUMENTATION AND OPERATIONAL CONSIDERATIONS

10.1. Appendix 1: Regulatory, ethical, and study oversight considerations

10.1.1. Regulatory and ethical considerations

- This study will be conducted in accordance with the protocol and with the following:
  - Consensus ethical principles derived from international guidelines including the Declaration of Helsinki and CIOMS international ethical guidelines
  - Applicable ICH GCP guidelines
  - Applicable laws and regulations
- The protocol, protocol amendments, ICF, IB, and other relevant documents (e.g., advertisements) must be submitted to an IRB/IEC by the investigator and reviewed and approved by the IRB/IEC before the study is initiated.
- Substantial amendments to the protocol will require IRB/IEC approval before implementation of changes made to the study design, except for changes necessary to eliminate an immediate hazard to study participants.
- Protocols and any substantial amendments to the protocol will require health authority approval prior to initiation except for changes necessary to eliminate an immediate hazard to study participants.
- The investigator will be responsible for the following, as applicable:
  - Providing written summaries of the status of the study to the IRB/IEC annually or more frequently in accordance with the requirements, policies, and procedures established by the IRB/IEC
  - Notifying the IRB/IEC of SAEs or other significant safety findings as required by IRB/IEC procedures
  - Providing oversight of the conduct of the study at the site and adherence to requirements of 21 CFR, ICH guidelines, the IRB/IEC, European regulation 536/2014 for clinical studies (if applicable), and all other applicable local regulations

10.1.2. Financial disclosure

- Investigators and sub-investigators will provide the sponsor with sufficient, accurate financial information as requested to allow the sponsor to submit complete and accurate financial certification or disclosure statements to the appropriate regulatory authorities. Investigators are responsible for providing information on financial interests during the course of the study and for 1 year after completion of the study.

10.1.3. Informed consent process

- The investigator or the investigator's representative will explain the nature of the study, including the risks and benefits, to the participants/participants' parent(s)/LAR(s) and answer all questions regarding the study.
- Potential participants/participant's parent(s)/LAR(s) must be informed that their/their child's participation is voluntary. They or their LAR(s) will be required to physically sign a statement of informed consent that meets the requirements of 21 CFR 50, local regulations, ICH guidelines, HIPAA requirements, privacy and data protection requirements, where applicable, and the IRB/IEC or study center.
- The investigator must obtain assent from the minor participant in addition to the consent provided by the participants' parent(s)/LAR(s) when a minor can assent to participate in a study. The investigator is also accountable for determining a minor's capacity to assent to participation in a research study according to the local laws and regulations.
- In accordance with local laws and regulations, participants who become legally emancipated during the study, i.e., reach the legal age of consent, must be reconsented.
- The medical record must include a statement that physical informed consent was obtained before the participant was enrolled in the study and the date the physical consent was obtained. The authorized person obtaining the informed consent must also sign the ICF.
- Participants must be reconsented to the most current version of the ICF(s) during their participation in the study.
- A physical copy of the ICF(s) must be provided to the participant/participant's parent(s)/LAR(s).
- The participant must provide consent by signing an ICF, which summarizes the study, includes a consent statement and provides documentation that the participant agrees to continue participating in the study. Participants who are rescreened are required to sign a new ICF.

- The ICF will contain a separate section that addresses the use of remaining mandatory samples for optional exploratory research. The investigator or authorized designee will explain to each participant the objectives of the exploratory research. Participants will be told that they are free to refuse to participate and may withdraw their consent at any time and for any reason during the storage period.
- In case of unexpected pregnancy, participant must be informed that personal information such as date of birth, sex of the baby will be collected as part of safety follow-up. Consent for the baby may be obtained from the participant and/or their partner as per local regulations.

10.1.4. Recruitment strategy

- The study is planned to be conducted at sites in multiple countries. The recruitment plan will be defined by each participating site. Recruitment will be tracked using RTSM system.
- The recruitment plan may be adapted based on the actual number of participants enrolled in each country. In case a site would fall behind in participant recruitment, a redistribution of the enrollment target per site in the participating countries may be made. This would allow the other participating sites to enroll additional participants to ensure full and timely enrollment of the overall targeted number of participants specified in this protocol.
- When the target number of participants is reached in a particular group (renal Tx, lung Tx or healthy) or age category (healthy), further enrollment of participants in that category will be stopped. If needed, the maximum number of participants in a particular disease or age category may be adapted during the study.
- The procedures for participants identification/recruitment (e.g., referral letters, advertisements etc.) must be approved by the IRB/IEC together with the material intended for participants identification/recruitment and participants use.

10.1.5. Data protection

- Participants will be assigned a unique identifier by the investigator. Any participant records or datasets that are transferred to the sponsor will contain the identifier only; participant names or any information which would make the participant identifiable will not be transferred.
- GSK will ensure protection of the personal data of the investigator and site staff which is collected within the framework of and for the purpose of the study.
- The participant/participants' parent(s)/LAR(s) must be informed that their/the minor participant's personal study-related data will be used by the sponsor in accordance with local data protection law. The level of disclosure must also be explained to the participant/participants' parent(s)/LAR(s), that their/the minor participant's data will be used as described in the informed consent.

- The participant/participants' parent(s)/LAR(s) must be informed that their/the minor participant's medical records may be examined by Clinical Quality Assurance auditors or other authorized personnel appointed by the sponsor, by appropriate IRB/IEC members, and by inspectors from regulatory authorities.
- The contract between sponsor and study sites specifies responsibilities of the parties related data protection, including handling of data security breaches and respective communication and cooperation of the parties.
- Information technology systems used to collect, process, and store study-related data are secured by technical and organizational security measures designed to protect such data against accidental or unlawful loss, alteration, or unauthorized disclosure or access. GSK and/or trusted third parties working on behalf of GSK and/or institutions working with GSK for the purposes of this study are contractually bound to protect participant coded data. GSK will protect participant coded data and will only share it as described in the ICF.

10.1.6. Committees structure

- A SRT is in place for each GSK product. It comprises of a global cross-functional team responsible for the ongoing assessment of benefit-risk for a product. The SRT contribute to the continual assessment of incoming new efficacy and safety information.
- Review and evaluation of safety data will be performed by an IDMC on a regular basis. Clinical experts on renal and lung transplantation will be included in the IDMC quorum. In preparation of the IDMC meetings, safety data will be summarized according to the IDMC charter. Only the outcomes and recommendations of the IDMC will be communicated to the study team. Operational details will be provided in the IDMC charter.

10.1.7. Dissemination of Clinical Study Data

- The key design elements of this protocol and results summaries will be posted on www.ClinicalTrials.gov and/or GSK Clinical Study Register in compliance with applicable regulations/GSK policy. GSK will aim to register protocols summaries prior to study start and target results summaries submission within 12 months of primary/ study completion date. Where external regulations require earlier disclosure, GSK will follow those timelines.
- Where required by regulation, summaries will also be posted on applicable national or regional clinical study registers.
- Where required by applicable regulatory requirements, an investigator signatory will be identified for the approval of the study report, and provided reasonable access to statistical tables, figures, and relevant reports. GSK will also provide the investigator with the full summary of the study results, including a summary of trial results understandable to laypersons. The investigator is encouraged to share the plain language summary with the study participants, as appropriate. The full study report will be made available upon request, after decision on marketing authorization by regulatory authorities.

- GSK will provide the investigator with the randomization codes and participant-level line listings for their site only after completion of the full statistical analysis.
- GSK intends to make anonymized participant-level data from this study available to external researchers for scientific analyses or to conduct further research that can help advance medical science or improve patient care. This helps ensure the data provided by study participants are used to maximum effect in the creation of knowledge and understanding.

10.1.8. Data quality assurance

- All participant data relating to the study will be recorded on printed or electronic CRFs unless transmitted to the sponsor or designee electronically (e.g., laboratory data). The investigator is responsible for verifying that data entries are accurate and correct by physically or electronically signing the CRF.
- Guidance on completion of eCRFs will be provided in eCRF completion guidelines.
- The investigator must permit study-related monitoring, audits, IRB/IEC review, and regulatory agency inspections and provide direct access to source documents.
- QTLs will be predefined in the Quality plan to identify systematic issues that can impact participant right, safety and/or reliability of study results. These predefined parameters will be monitored during the study, and important deviations from the QTLs and remedial actions taken will be summarized in the CSR.
- Monitoring details describing strategy, including definition of study critical data items and processes (e.g., risk-based initiatives in operations and quality such as risk management and mitigation strategies and analytical risk-based monitoring, involvement of central reading mechanism) methods, responsibilities, and requirements, including handling of noncompliance issues and monitoring techniques (central, remote, or on-site monitoring) are provided in the monitoring plan.
- The sponsor or designee is responsible for the data management of this study, including quality checking of the data.
- The sponsor assumes accountability for actions delegated to other individuals (e.g., contract research organizations).
- Records and documents, including signed ICF, pertaining to the conduct of this study must be retained by the investigator for 25 years from the issue of the final CSR/ equivalent summary unless local regulations or institutional policies require a different retention period. No records may be destroyed during the retention period without the written approval of the sponsor. No records may be transferred to another location or party without written notification to the sponsor.
- When copies of source documents are shared externally for review by a central reader mechanism (e.g., endpoint adjudication committee; expert reader), documents are stored by the external body for 25 years.

10.1.9. Source documents

- Source documents provide evidence for the existence of the participant and substantiate the integrity of the data collected. Source documents are filed at the investigator's site.
- Data reported on the eCRF or entered in the eCRF that are transcribed from source documents must be consistent with the source documents or the discrepancies must be explained. The investigator may need to request previous medical records or transfer records, depending on the study. Also, current medical records must be available.
- Definition of what constitutes source data and its origin can be found in Definition of Terms.
- The investigator must maintain accurate documentation (source data) that supports the information entered in the eCRF.
- The sponsor or designee will perform monitoring to confirm that data entered into the eCRF by authorized site personnel are accurate, complete, and verifiable from source documents; that the safety and rights of participants are being protected; and that the study is being conducted in accordance with the currently approved protocol and any other study agreements, ICH GCP, and all applicable regulatory requirements.
- Copies of documents are shared with external third parties contracted by GSK for review by a central reader mechanism (e.g., endpoint adjudication committee; expert reader). The non-exhaustive list of documents shared to inform the central reader may include, discharge summaries, imaging reports, ECG reports etc. Participant names or any information which would make the participant identifiable or is not essential for the central reader mechanism will be redacted by the investigator sites prior to transfer. Details of the list of documents and the redaction procedure are provided in the site manual or equivalent. These documents will be used by the third party solely for the purpose indicated within this protocol.

10.1.10. Study and site start and closure

Start of study and first act of recruitment

The start of study and the first act of recruitment are defined as FSFV (first ICF signature date).

Study/site termination

GSK or designee reserves the right to close the study site or terminate the study at any time for any reason at the sole discretion of GSK. Study sites will be closed upon study completion. A study site is considered closed when all required documents and study supplies have been collected and a study site closure visit has been performed.

The investigator may initiate study site closure at any time, provided there is reasonable cause and sufficient notice is given in advance of the intended termination.

Reasons for the early closure of a study site by the sponsor or investigator may include but are not limited to:

- For study termination:
  - Discontinuation of further study intervention development
- For site termination:
  - Failure of the investigator to comply with the protocol, the requirements of the IRB/IEC or local health authorities, the sponsor’s procedures, or GCP guidelines
  - Inadequate or no recruitment (evaluated after a reasonable amount of time) of participants by the investigator
  - Total number of participants included earlier than expected in the study

If the study is prematurely terminated or temporarily suspended, the sponsor shall promptly inform the investigators, the IECs/IRBs, the regulatory authorities, and any contract research organization(s) used in the study of the reason for termination or temporary suspension, as specified by the applicable regulatory requirements. The investigator shall promptly inform the participant and should assure appropriate participant therapy and/or follow-up.

10.1.11. Publication policy

The results of this study may be published in peer reviewed scientific literature and/or presented at scientific meetings. The sponsor will comply with the requirements for publication of study results in accordance with standard editorial and ethical practice and as per the sponsor’s internal policy. Authorship will be determined by mutual agreement and in line with ICMJE authorship requirements.

10.2. Appendix 2: Clinical laboratory tests

10.2.1. RSV-A and RSV-B neutralization assays

The serum neutralization assay is a functional assay that measures the ability of serum antibodies to neutralize RSV entry and replication in a host cell line.

Virus neutralization is performed by incubating a fixed amount of RSV-A strain (Long, ATCC No. VR-26) or RSV-B strain (18537, ATCC No. VR-1580) with serial dilutions of the test serum. The serum-virus mixture is then transferred onto a layer of Vero cells (African Green Monkey, kidney, *Cercopitheus aethiops*, ATCC CCL 81) and incubated for 2 days to allow infection of the Vero cells by non-neutralized virus and the formation of plaques in the cell layer. Following a fixation step, RSV-infected cells are detected using a primary antibody directed against RSV (Polyclonal anti-RSV-A/B IgG) and a secondary antibody conjugated to horse-radish peroxidase, allowing the visualization of plaques after coloration with *TrueBlue* peroxidase substrate. Viral plaques are counted using an automated microscope coupled to an image analyzer (Scanlab system with a Reading software or equivalent). For each serum dilution, a ratio, expressed as a

percentage, is calculated between the number of plaques at each serum dilution and the number of plaques in the virus control wells (no serum added). The serum neutralizing titer is expressed in Estimated Dilution 60 and corresponds to the inverse of the interpolated serum dilution that yields a 60% reduction in the number of plaques compared to the virus control wells, as described by others [Barbas, 1992; Bates, 2014]. Secondary standards calibrated against the international reference (NIBSC 16/284) [McDonald, 2018; McDonald, 2020] are included in every run to allow conversion into international units per milliliter.

10.2.2. Intracellular cytokine staining

The ICS is used to assess RSVPreF3-specific CD4+ and/or CD8+ T cells expressing at least 2 activation markers including at least 1 cytokine among CD40L, 4-1BB, IL-2, TNF-α, IFN-γ, IL-13, IL-17 (secondary endpoint).

As previously described [Moris, 2011], thawed PBMCs are stimulated in vitro in the presence of anti-CD28 and anti-CD49d antibodies either with pools of 15-mer peptides overlapping by 11 amino acids and spanning the sequence of the RSVPreF3 protein, or with medium. After 2 hours of incubation at 37ºC, Brefeldin A is added to inhibit cytokine secretion during an additional overnight incubation at 37ºC. Cells are subsequently harvested, stained for surface markers (CD4+ and CD8+) and then fixed. Fixed cells are then permeabilized and stained with labeled antibodies specific for the following immune markers:

- CD3+: phenotyping T cells;
- CD40L (CD154), expressed on activated CD4+ T cells, [Chattopadhyay, 2005; Frentsch, 2005; Samten, 2000; Stubbe, 2006];
- IL-2: key for the development, survival and function of T cells [Boyman, 2012];
- TNF-α: anti-viral/intracellular factor, pro-inflammatory cytokine, cytotoxicity [Sedger, 2014];
- IFN-γ: anti-viral factor, associated with the Th1-like profile [Schoenborn, 2007];
- 4-1BB (CD137), expressed on activated CD4+ and CD8+ T cells [Wölfl, 2008];
- IL-13: associated with the Th2-like profile [Bao, 2015];
- IL-17: associated with the Th17-like profile [Korn, 2009].

After staining with the markers above, the cellular samples are analyzed by flow-cytometry allowing to determine the frequency of CD4+ and/or CD8+ T cells expressing the marker(s) of interest per million of total CD4+ and/or CD8+ T cells.

10.3. Appendix 3: AEs and SAEs: Definitions and procedures for recording, evaluating, follow-up, and reporting

10.3.1. Definition of AE

**AE definition**
• An AE is any untoward medical occurrence in a clinical study participant, temporally associated with the use of a study intervention, whether or not considered related to the study intervention.<br>• NOTE: An AE can therefore be any unfavorable and unintended sign (including an abnormal laboratory finding), symptom, or disease (new or exacerbated) temporally associated with the use of a study intervention.
**Events Meeting the AE Definition**
• Any abnormal laboratory test results (hematology, clinical chemistry, or urinalysis) or other safety assessments (e.g., ECG, radiological scans, vital signs measurements), including those that worsen from baseline, considered clinically significant in the medical and scientific judgment of the investigator (i.e., not related to progression of underlying disease).<br>• Exacerbation of a chronic or intermittent pre-existing condition including either an increase in frequency and/or intensity of the condition.<br>• New condition detected or diagnosed after study intervention administration even though it may have been present before the start of the study.<br>• Signs, symptoms, or the clinical sequelae of a suspected intervention- intervention interaction.<br>• Signs, symptoms, or the clinical sequelae of a suspected overdose of either study intervention or a concomitant medication. Overdose per se will not be reported as an AE/SAE unless it is an intentional overdose taken with possible suicidal/self-harming intent. Such overdoses should be reported regardless of sequelae.<br>• Events that occur as a result of protocol-mandated procedures (i.e., invasive procedures, modification of participant's previous therapeutic regimen).
**Events NOT Meeting the AE Definition**
• Any clinically significant abnormal laboratory findings or other abnormal safety assessments that are associated with the underlying disease, unless judged by the investigator to be more severe than expected for the participant's condition.<br>• Medical or surgical procedure (e.g., endoscopy, appendectomy): the condition that leads to the procedure is the AE.<br>• Situations in which an untoward medical occurrence did not occur (social and/or convenience admission to a hospital, admission for routine examination.).

• Anticipated day-to-day fluctuations of pre-existing disease(s) or condition(s) present or detected at the start of the study that do not worsen. Pre-existing diseases will be recorded in the medical history section of the eCRF.<br>• Hospitalization for elective treatment of a pre-existing condition (known or diagnosed before signing the informed consent) that did not worsen from baseline.

10.3.2. Definition of SAE

An SAE is defined as any untoward medical occurrence that, at any dose, meets one or more of the criteria listed:
• Results in death
• Is life-threatening<br>The term 'life-threatening' in the definition of 'serious' refers to an event in which the participant was at risk of death at the time of the event. It does not refer to an event, which hypothetically might have caused death, if it were more severe.
• Requires inpatient hospitalization or prolongation of existing hospitalization<br>– In general, hospitalization signifies that the participant has been admitted (usually involving at least an overnight stay) at the hospital or emergency ward for observation and/or treatment that would not have been appropriate in the physician’s office or outpatient setting. Complications that occur during hospitalization are AE. If a complication prolongs hospitalization or fulfills any other serious criteria, the event is serious. When in doubt as to whether “hospitalization” occurred or was necessary, the AE should be considered serious.<br>– Hospitalization for elective treatment of a pre-existing condition that did not worsen from baseline is not considered an AE.
• Results in persistent or significant disability/incapacity<br>– The term disability means a substantial disruption of a person’s ability to conduct normal life functions.<br>– This definition is not intended to include experiences of relatively minor medical significance such as uncomplicated headache, nausea, vomiting, diarrhea, influenza, and accidental trauma (e.g., sprained ankle) that may interfere with or prevent everyday life functions but do not constitute a substantial disruption.
• Is a congenital anomaly/birth defect in the offspring of a study participant
• Abnormal pregnancy outcomes (e.g., spontaneous abortion, fetal death, stillbirth, congenital anomalies, ectopic pregnancy)

• Is a suspected transmission of any infectious agent via an authorized medicinal product
• Other situations:<br>– Possible Hy's Law case: ALT ≥ 3x ULN AND total bilirubin ≥ 2x ULN (>35% direct bilirubin) or INR >1.5 must be reported as SAE<br>– Medical or scientific judgment should be exercised by the investigator in deciding whether SAE reporting is appropriate in other situations such as significant medical events that may jeopardize the participant or may require medical or surgical intervention to prevent one of the other outcomes listed in the above definition. These events should usually be considered serious.<br>Examples of such events include invasive or malignant cancers, intensive treatment for allergic bronchospasm, blood dyscrasias, convulsions, or development of intervention dependency or intervention abuse.

10.3.3. Solicited events

- **Definition of solicited event**

Solicited events are predefined events (administration site events and systemic events) for which the participant/participant's parent(s)/LAR(s) is specifically questioned, and which are noted by the participant/participant's parent(s)/LAR(s) in their diary.

Table 22 Solicited administration site events

Pain at administration site
Redness at administration site
Swelling at administration site

Table 23 Solicited systemic events

Fever
Myalgia (muscle pain)
Arthralgia (joint pain)
Headache
Fatigue (tiredness)

Note: Participants/participants' parent(s)/LAR(s) will be instructed to measure and record the oral/axillary temperature in the evening, using a sponsor provided device. If additional temperature measurements are taken at other times of the day, participants/participants' parent(s)/LAR(s) will be instructed to record the highest temperature in the diary card.

10.3.4. Unsolicited AE

• **Definition of unsolicited AE**
An unsolicited AE is an AE that was either not included in the list of solicited events or could be included in the list of solicited events but with an onset outside the specified period of follow-up for solicited events. Unsolicited AEs must have been communicated by participants/participant's parent(s)/LAR(s) who has signed the informed consent. Unsolicited AEs include both serious and nonserious AEs.<br>• Potential unsolicited AEs may be medically attended (i.e., symptoms or illnesses requiring a hospitalization, emergency room visit, or visit to/by a healthcare provider). The participants/participant's parent(s)/LAR(s) will be instructed to contact the site as soon as possible to report medically attended event(s), as well as any events that, though not medically attended, are of participant's/participant's parent(s)/LAR(s) concern. Detailed information about reported unsolicited AEs will be collected by qualified site personnel and documented in the participant's records.<br>• Unsolicited AEs that are not medically attended nor perceived as a concern by the participant/participant's parent(s)/LAR(s) will be collected during an interview with the participant/participant's parent(s)/LAR(s) and by review of available medical records at the next visit.

10.3.5. Recording, assessment and follow-up of AE, SAE, AESIs and pregnancies

10.3.5.1. AE and SAE recording

- When an AE/SAE occurs, it is the responsibility of the investigator to review all documentation (e.g., hospital progress notes, laboratory reports, and diagnostics reports) related to the event.
- The investigator will then record all relevant AE/SAE information.
- It is not acceptable for the investigator to send photocopies of the participant's medical records to GSK in lieu of completion of the eCRF/required form.
- There may be instances when copies of medical records for certain cases are requested by GSK. In this case, all participant identifiers, with the exception of the participant number, will be redacted on the copies of the medical records before submission to GSK.
- The investigator will attempt to establish a diagnosis of the event based on signs, symptoms, and/or other clinical information. Whenever possible, the diagnosis (not the individual signs/symptoms) will be documented as the AE/SAE.
- A pDiary will be used in this study to capture solicited administration site or systemic events. The pDiary will be distributed to all participants at Visit 1. The participant should be trained on how and when to complete the pDiary. If a

participant is unable or not willing to complete the pDiary him/herself, he/she may be helped by a caregiver (refer to Definition of Terms for the definition of caregiver).

- Anyone who measures administration site or systemic events and who will record the event in the pDiary, e.g., the study caregiver, should have received a caregiver information letter explaining the role of the caregiver prior to completing the pDiary. This training must be documented in the participant's source record.
- For each solicited and unsolicited AE the participant experiences, the participant/participant's parent(s)/LAR(s) will be asked if they/the minor participant received medical attention (defined as unscheduled visit to or from medical personnel for any reason, including emergency room visits). This information will be recorded in the participant's pDiary or in the participant's diary (for solicited AEs) and in the participant's eCRF as part of normal AE reporting (for unsolicited AEs). Medical attention received for SAEs/AESIs will have to be reported using the normal AE reporting process in the eCRF.
- If any individual other than the participant/participant's parent(s)/LAR(s) is making entries in the pDiary, their identity must be documented in the participant's source record.
- Note: pDiary may be completed by a minor participant under the supervision of the participant's parent(s)/LAR(s) provided the minor is capable of assessing and reporting the information to be recorded on pDiary. The ultimate accountability for completion of the pDiary remains with the participant's parent(s)/LAR(s). The investigator should discuss this accountability with the participant's parent(s)/LAR(s).
- The investigator or delegate should verify the reported information during a discussion with the minor participant preferably in the presence of their parent(s)/LAR(s).
- Collect and verify completed pDiary during discussions with the participant on Visit 3 (all groups) and 4 (IC 2-dose group), respectively.
- Any unreturned pDiary will be sought from the participant/participant's parent(s)/LAR(s) through telephone call(s) or any other convenient procedure.
- The investigator or delegate will transcribe the required information into the eCRF in English.

10.3.5.2. Assessment of intensity

The investigator will make an assessment of intensity for each AE, AESI and SAE reported during the study and assign it to one of the following categories:

- **Mild:**
  A type of AE that is usually transient and may require only minimal treatment or therapeutic intervention. The event does not generally interfere with usual activities of daily living.

- **Moderate:**
  A type of AE that is usually alleviated with additional specific therapeutic intervention. The event interferes with usual activities of daily living, causing discomfort but poses no significant or permanent risk of harm to the research participant.
- **Severe:**
  A type of AE that interrupts usual activities of daily living, or significantly affects clinical status, or may require intensive therapeutic intervention.
- The intensity of the following solicited AEs will be assessed as described:

Table 24 Intensity scales for solicited events in participants ≥18 YoA (IC group) and ≥50 YoA (HA group)

Event	Intensity grade	Parameter
Pain at administration site	0	None
	1	Mild: Any pain neither interfering with nor preventing normal everyday activities
	2	Moderate: Painful when limb is moved and interferes with everyday activities
	3	Severe: Significant pain at rest. Prevents normal everyday activities
Redness at administration site	See Table 25	Greatest surface diameter in mm
Swelling at administration site	See Table 25	Greatest surface diameter in mm
Temperature*	See Table 25	Temperature in °C/°F
Headache	0	None**
	1	Mild: Headache that is easily tolerated
	2	Moderate: Headache that interferes with normal activity
	3	Severe: Headache that prevents normal activity
Fatigue (tiredness)	0	None**
	1	Mild: Fatigue that is easily tolerated
	2	Moderate: Fatigue that interferes with normal activity
	3	Severe: Fatigue that prevents normal activity
Myalgia (muscle pain)	0	None**
	1	Mild: Myalgia present but does not interfere with activity
	2	Moderate: Myalgia that interferes with normal activity
	3	Severe: Myalgia that prevents normal activity
Arthralgia (joint pain)	0	None**
	1	Mild: Arthralgia present but does not interfere with activity
	2	Moderate: Arthralgia that interferes with normal activity
	3	Severe: Arthralgia that prevents normal activity

* Refer to the SoA (Section 1.3) for the definition of fever and the preferred location for temperature measurement.
**For participants already experiencing some of the solicited systemic events, 'None' corresponds to 'similar to baseline' and only discomfort above baseline is to be reported as ≥1.

The maximum intensity of administration site redness/swelling, and fever will be scored at GSK as follows:

Table 25 Intensity scales of administration site redness/swelling, and fever

Intensity grade	Redness/Swelling	Fever
0	≤20 mm	<38.0°C (100.4°F)

1	>20 - ≤50 mm	≥38.0°C (100.4°F) - ≤38.5°C (101.3°F)
2	>50 - ≤100 mm	>38.5°C (101.3°F) - ≤39.0°C (102.2°F)
3	>100 mm	>39.0°C (102.2°F)

The investigator will assess the maximum intensity that occurred over the duration of the event for all unsolicited AEs (including SAEs) recorded during the study. The assessment will be based on the investigator's clinical judgment.

An AE that is assessed as Grade 3 (severe) should not be confused with an SAE. Grade 3 is a category used for rating the intensity of an event; and both AEs and SAEs can be assessed as Grade 3. An event is defined as 'serious' when it meets 1 of the predefined outcomes as described in the Section 10.3.2.

10.3.5.3. Assessment of causality

- The investigator is obligated to assess the relationship between study intervention and each occurrence of each AE/SAE. The investigator will use clinical judgment to determine the relationship.
- A reasonable possibility of a relationship conveys that there are facts, evidence, and/or arguments to suggest a causal relationship, rather than a relationship cannot be ruled out.
- Alternative causes, such as underlying disease(s), concomitant therapy, and other risk factors, as well as the temporal relationship of the event to study intervention administration, will be considered and investigated.
- For causality assessment, the investigator will also consult the IB and/or product information, for marketed products.
- The investigator must review and provide an assessment of causality for each AE/SAE and document this in the medical notes There may be situations in which an SAE has occurred and the investigator has minimal information to include in the initial report to GSK. However, it is very important that the investigator always make an assessment of causality for every event before the initial transmission of the SAE data to GSK.
- The investigator may change their opinion of causality in light of follow-up information and send an SAE follow-up report with the updated causality assessment.
- The causality assessment is one of the criteria used when determining regulatory reporting requirements.

10.3.5.4. Assessment of outcomes

The investigator will assess the outcome of all serious and nonserious unsolicited AEs recorded during the study as:

- Recovered/resolved
- Recovering/resolving
- Not recovered/not resolved
- Recovered with sequelae/resolved with sequelae
- Fatal (SAEs only).

10.3.5.5. Follow-up of AEs, SAEs, AESIs and pregnancies

- The investigator is obligated to perform or arrange for the conduct of supplemental measurements and/or evaluations as medically indicated or as requested by GSK to elucidate the nature and/or causality of the AE or SAE as fully as possible. This may include additional laboratory tests or investigations, histopathological examinations, or consultation with other health care professionals.
- New or updated information will be recorded in the originally submitted documents.
- The investigator will submit any updated SAE data to GSK within 24 hours of receipt of the information.
- After the initial AE/SAE/AESI/pregnancy or any other event of interest, the investigator is required to proactively follow each participant at subsequent visits/contacts. All SAEs will be followed until the event is resolved, stabilized, otherwise explained, or the participant is lost to follow-up.
- Other nonserious AEs must be followed until end of study or until the participant is lost to follow-up.

Follow-up during the study

AEs/AESIs documented at a previous visit/contact and defined as not recovered/not resolved or recovering/resolving will be reviewed at subsequent visits/contacts until end of study.

If a participant dies during their participation in the study or during a recognized follow-up period, GSK will be provided with any available postmortem findings, including histopathology.

Follow-up of pregnancies

Pregnant participants will be followed to determine the outcome of the pregnancy. At the end of the pregnancy, whether full-term or premature, information on the status of the mother and child will be forwarded to GSK using the electronic pregnancy report and the

AE Report if applicable. Generally, the follow-up period does not need to be longer than 6 to 8 weeks after the estimated date of delivery.

Regardless of the reporting period for SAEs in this study, if the pregnancy outcome is an SAE, it should always be reported as such.

Furthermore, the investigator must report any SAE occurring as a result of a post-study pregnancy that is considered by the investigator to be reasonably related to the study intervention, to GSK as described in the Section 10.3.5.7.

10.3.5.6. Updating of SAE, AESI and pregnancy information after removal of write access to the participant's eCRF

When additional SAE, AESI or pregnancy information is received after write access to the participant's eCRF is removed, new or updated information should be recorded on the appropriate paper report, with all changes signed and dated by the investigator. The updated report should be sent to the Study contact for reporting SAEs (refer to Section 8.4.7).

10.3.5.7. Reporting of SAEs, AESIs and pregnancies

SAE Reporting to GSK via an Electronic Data Collection Tool

- The primary mechanism for reporting an SAE to GSK will be the electronic data collection tool.
- If the electronic system is unavailable, then the site will use the paper SAE data collection tool (see next section) to report the event within 24 hours.
- The site will enter the SAE data into the electronic system as soon as it becomes available.
- After the study is completed at a given site, the electronic data collection tool will be taken offline to prevent the entry of new data or changes to existing data.
- If a site receives a report of a new SAE from a study participant or receives updated data on a previously reported SAE after the electronic data collection tool has been taken offline, then the site can report this information on a paper SAE form (see next section).
- If the site during the course of the study or post-study becomes aware of any serious, nonserious AEs, pregnancy exposure, related to any GSK non-IMP they will report these events to GSK or to the concerned competent authority via the national spontaneous reporting system. These will be classified as spontaneous ICSRs.
- Contacts for SAE reporting can be found in Section 8.4.7.

SAE Reporting to GSK via Paper Data Collection Tool

- Email/fax transmission of the SAE paper data collection tool is the preferred method to transmit this information.
- In rare circumstances and in the absence of email/fax equipment, notification by telephone is acceptable with a copy of the SAE data collection tool sent by overnight mail or courier service.
- Initial notification via telephone does not replace the need for the investigator to complete and sign the SAE data collection tool within the designated reporting timeframes.
- Contacts for SAE reporting can be found in Section 8.4.7.

10.4. Appendix 4: Guidance on Contraception, Women not considered as WOCBP and collection of pregnancy information

This section covers wording on the definition of what we consider to be a woman of childbearing potential as well as guidance on what is considered as adequate contraception.

10.4.1. Definitions

10.4.1.1. Woman of childbearing potential (WOCBP)

A woman is considered WOCBP (fertile) from the time of menarche until becoming postmenopausal unless permanently sterile (see below).

Note: Menarche is the first onset of menses in a young female. Menarche is normally preceded by several changes associated with puberty including breast development and pubic hair growth.

10.4.1.2. Woman of Nonchildbearing potential (WONCBP)

Women in the following categories are considered WONCBP:

- Premenopausal female permanently sterile due to one of the following procedures:
  - Documented hysterectomy
  - Documented bilateral salpingectomy
  - Documented bilateral oophorectomy

  For permanently sterile individuals due to an alternate medical cause other than the above, (e.g., Mullerian agenesis, androgen insensitivity, gonadal dysgenesis), investigator discretion should be applied to determining study entry. If reproductive status is questionable, additional evaluation should be considered.

Note: Documentation can come from the site personnel's review of the participant's medical records, medical examination, or medical history interview.

- Postmenopausal female

A postmenopausal state is defined as no menses for 12 months without an alternative medical cause.

- A high FSH level in the postmenopausal range may be used to confirm a postmenopausal state in women not using hormonal contraception or HRT. However, in the absence of 12 months of amenorrhea, confirmation with more than one FSH measurement is required.
- Females on HRT and whose menopausal status is in doubt must discontinue HRT to allow confirmation of postmenopausal status before study enrollment.

10.4.2. Contraception guidance

10.4.2.1. Woman of childbearing potential (WOCBP)

Female participants of childbearing potential are eligible to participate if they agree to use a highly effective contraceptive method consistently and correctly according to the methods listed in GSK's list of highly effective contraceptive methods (Table 26).

Table 26 Highly effective contraceptive methods

Highly Effective Contraceptive Methods That Are User Dependent [a]<br>*Failure rate of <1% per year when used consistently and correctly*
Combined (estrogen- and progestogen-containing) hormonal contraception associated with inhibition of ovulation<br>• Oral[b]<br>• Intravaginal<br>• Transdermal
Progestogen-only hormonal contraception associated with inhibition of ovulation<br>• Injectable<br>• Oral[b] (only if allowed by local regulations or if part of standard medical practice in the country)
Highly Effective Methods That Are User Independent
• Implantable progestogen-only hormonal contraception associated with inhibition of ovulation<br>• Intrauterine device (IUD)<br>• Intrauterine hormone-releasing system (IUS)<br>• Bilateral tubal occlusion/ligation
Vasectomized partner<br>*(A vasectomized partner is a highly effective contraception method provided that the partner is the sole male sexual partner of the WOCBP and the absence of sperm has been confirmed. If not, an additional highly effective method of contraception should be used.)*
Male partner sterilization prior to the female participant's entry into the study, and this male is the sole partner for that participant,<br>*(The information on the male sterility can come from the site personnel's review of the participant's medical records; medical examination and/or semen analysis, or medical history interview provided by her or her partner)*
Sexual abstinence<br>(*Sexual abstinence is considered a highly effective method only if defined as refraining from heterosexual intercourse during the entire period of risk associated with the study drug. The reliability of sexual abstinence needs to be evaluated in relation to the duration of the study and the preferred and usual lifestyle of the participant).*

[a] Typical use failure rates may differ from those when used consistently and correctly. Use should be consistent with local regulations regarding the use of contraceptive methods for subjects in clinical studies.

[b] For WOCBP taking hormonal contraception, an additional barrier contraception method is recommended if they are concomitantly taking drugs that interact and reduce effectiveness of hormonal contraception. The investigator should check the list of drugs that could reduce hormonal contraceptive effectiveness (Potent drug enzyme inducers).

10.5. Appendix 5: Grading scales in scope of the SOT population

10.5.1. Chronic kidney disease (CKD) classification in RTx patients

Stages of CKD quantify the severity of the disease. CKD is classified into 5 stages [Levey, 2005].

- Stage 1: Normal GFR (≥90 mL/min/1.73 m^2) plus either persistent albuminuria or known structural or hereditary renal disease
- Stage 2: GFR 60 to 89 mL/min/1.73 m^2
- Stage 3: 30 to 59 mL/min/1.73 m^2
- Stage 4: GFR 15 to 29 mL/min/1.73 m^2
- Stage 5: GFR < 15 mL/min/1.73 m^2

GFR (in mL/min/1.73 m^2) in CKD can be estimated by the CKD-EPI creatinine equation: 141 × (serum creatinine) -1.209× 0.993 age. The result is multiplied by 1.018 if the patient is female, and by 1.159 if the patient is African American. For female African Americans, the result is multiplied by 1.018 × 1.159 (1.1799). Alternatively, GFR can be estimated using timed (most commonly 24 hours) urine creatinine clearance using measured serum and urine creatinine; this equation tends to overestimate GFR by 10 to 20%. It is used when serum creatinine assessment may not be as accurate (e.g., in patients who are sedentary, very obese, or very thin). Serum cystatin C is an alternative endogenous GFR marker used as a confirmatory test in people with nonrenal factors affecting serum creatinine level (e.g., extremely high, or low muscle mass, exogenous creatine intake, amputations or neuromuscular diseases, and high protein or exclusively plant-based diets). GFR is calculated using CKD-EPI cystatin C equation.

The CKD-EPI formula is more accurate than the MDRD and Cockcroft-Gault formulas, particularly for patients with a GFR near normal values. The CKD-EPI equation yields fewer falsely positive results indicating chronic kidney disease and predicts outcome better than the other formulas.

10.5.1.1. Revised equations for eGFR from serum creatinine in Japan

In Japanese population other equations might be used [Matsuo, 2009]. The literature reports that the new Japanese coefficient for the modified IDMS–MDRD Study equation and the new Japanese equation are more accurate for the Japanese population than the previously reported equations. If such equations are used to calculate eGFR they should be provided to the sponsor prior to the study start (see Section 8.1.7.1).

IDMS–MDRD Study equation with new Japanese coefficient:

- **3-variable Japanese equation:**
  eGFR = 194 x $SCr^{-1.094}$ x $Age^{-0.287}$ (x 0.739 if female)
- **5-variable Japanese equation:**
  eGFR = 142 x $SCr^{-0.923}$ x $Age^{-0.185}$ x $Alb^{0.414}$ x $SUN^{-0.233}$ (x 0.772 if female)

Alb = Albumin; eGFR = estimated glomerular filtration rate; IDMS = isotope dilution mass spectrometry; MDRD = Modification of Diet in Renal Disease; SCr = serum creatinine; SUN = serum urea nitrogen

Although the 5-variable Japanese equation estimates GFR more accurately than other equations, SUN and albumin are not routinely measured in Japan.

Because the new 3-variable Japanese equation provided reasonably accurate eGFRs, it was recommended to use the new 3-variable Japanese equation.

10.5.2. Proteinuria/albuminuria categories in CKD in RTx patients

Adapted from [Chapter 1: Definition and classification of CKD, 2013].

Table 27 Proteinuria/albuminuria categories in CKD

Category	ACR (approximate equivalent)		Terms
	mg/mmol	mg/g	
A1	<30	<30	Normal to mild increase
A2	30-300	30-300	Moderately increased *
A3	>300	>300	Severely increased**

ACR = albumin-to-creatinine ratio

Table 28 Relationship among categories for albuminuria and proteinuria

Measure	Category		
	Normal to mildly increased (A1)	Moderately increased (A2)	Severely increased (A3)
ACR			
mg/mmol	<3	3-30	>30
mg/g	<30	30-300	>300
PCR			
g/mmol	<15	15-50	>50
mg/g	<150	150-500	>500
Protein reagent strip	Negative to trace	Trace to +	+ or greater

ACR = albumin-to-creatinine ratio; PCR = protein-to-creatinine ratio.
Albuminuria and proteinuria can be measured using excretion rates in timed urine collections, ratio of concentrations to creatinine in spot urine samples, and using reagent strips in spot urine samples.
The conversions are rounded for pragmatic reasons. (For an exact conversion from mg/g of creatinine to mg/mmol of creatinine, multiply by 0.113) Creatinine excretion varies with age, sex, race and diet; therefore, the relationship among these categories is approximate only. The relationship between urine reagent strip results and other measures depends on urine concentration.

10.5.3. Staging of bronchoscopy – acute cellular rejection (ACR) in LTx patients

Classification of ACR is according to the 2007 Working Formulation of the International Society for Heart and Lung Transplantation (ISHLT) [Roden, 2017].

Table 29 Classification of ACR

Acute Rejection		Small Airways Inflammation -Lymphocytic Bronchiolitis	
**ISHLT Grade**	**Definition**	**ISHLT Grade**	**Definition**
A0	None	B0	None
A1	Minimal	B1R	Low grade
A2	Mild	B2R	High grade
A3	Moderate	BX	Ungradable
A4	Severe		

10.5.4. Grading of bronchoscopy – antibody-mediated rejection (AMR) in LTx patients

Staging of Clinical AMR is as proposed by the 2016 ISHLT Consensus Report [Roden, 2017].

Table 30 Classification of AMR

	AMR Stage		
**Criteria**	**Definite**	**Probable**	**Possible**
Donor-specific antibodies	+	2 of the 3 criteria are present	1 of the 3 criteria is present
Histology suggestive of AMR	+		
C4d deposition	+		

10.5.5. CLAD staging in LTx patients

CLAD is classified into 5 stages. Once CLAD is diagnosed, staging is performed according to the decline in FEV1, compared with baseline. The date of onset of CLAD is defined as the date at which the first value of FEV1 ≤80% of baseline was recorded when subsequent values taken at least 3 weeks (and for definite CLAD up to 3 months) apart also fell below the threshold. The same principle holds for each stage [Verleden, 2019].

Table 31 Stages of CLAD

Stage	Spirometry
CLAD 0	Current FEV1 >80% FEV1 compared to baseline
CLAD 1	Current FEV1 >65–80% FEV1 compared to baseline
CLAD 2	Current FEV1 >50–65% FEV1 compared to baseline
CLAD 3	Current FEV1 >35–50% FEV1 compared to baseline
CLAD 4	Current FEV1 ≤35% FEV1 compared to baseline

10.6. Appendix 6: Country-specific requirements

10.6.1. Germany

Explanatory statement concerning Gender Distribution (Article 7, paragraph 2 (12) of the German GCP ORDER).

For this study, there is no intention to conduct specific analyses investigating the relationship between gender and the safety, immunogenicity and efficacy of the investigational RSVPreF3 OA vaccine. Recruitment will include both males and females. To not expose pregnant women and their fetuses/children to an investigational vaccine, females enrolled in this study will either be of nonchildbearing potential (i.e., hysterectomy, bilateral oophorectomy, bilateral salpingectomy or post-menopause), or if she is of childbearing potential, she must have a negative pregnancy test and use appropriate methods of contraception for the study duration (refer to the study protocol, Section 5.1 “Inclusion criteria” and Section 5.2 “Exclusion criteria”). Women who are pregnant, planning to become pregnant or breastfeeding are excluded from this study.

Remote Source Data Verification during exceptional situations in Germany

Frequent instream monitoring of safety data by the central study team at GSK is required for this study. Instream review of study data items and processes should be considered during exceptional situations/circumstances, such as with pandemics like COVID-19, focusing on key data points, patient assessments and processes that are critical to ensure the rights, safety and well-being of study participants and the integrity of the study and data. Prior to any rSDV activity a written agreement by the investigator will be obtained. The agreement includes the extent and the method of rSDV activities. Monitoring Plan and Study-Specific Risk Register will be updated to include rSDV activities and CRAs will be guided for the conduct of rSDV.

Option 1 Transfer of redacted Source Documentation

Process for transfer and review of redacted source documentation provided by the site:

- The CRA instructs study site on the source data needed for the remote SDV activities.
- The CRA instructs site staff they must pseudonymize the requested documentation, do a quality check that anonymized (redacted) areas cannot be read, and then delivers the documentation to the CRA in an encrypted form of communication (the site should have a documented process).
- The minimum requirements regarding quality of the copies will be agreed with the site upfront:
  - For the scanning of paper documents resolution will be a minimum of 300 dpi.
  - For the scanning of photographs and images resolution will be 600 dpi minimum.
  - Color scanners must be able to produce copies that match the original.
  - A4 format as final size without loss of information.
  - Documents will be saved as PDF.

- In order to maintain quality standards, a captured image will not be subjected to non- uniform scaling (i.e., sizing) or re-sampling to a lower resolution.

- Redacted source document scans will be sent to the CRA via email using 1 of the following secure options:
  - TLS connection:

  TLS connections are intended to support significant mail flow between GSK and external partners in a secure manner.

  - GSK Secure

  In cases where only a handful of users are communicating or the volume of emails is low, the use of GSK secure, the GSK ad-hoc message encryption solution is recommended.

  - Password protected PDF attachment

  A password protected scan (PDF) will be attached to an email. The password to open the attachment will be send in a separate email.

- The CRA may use the secure email website to assess whether the sites email address is secure (i.e., encrypted).

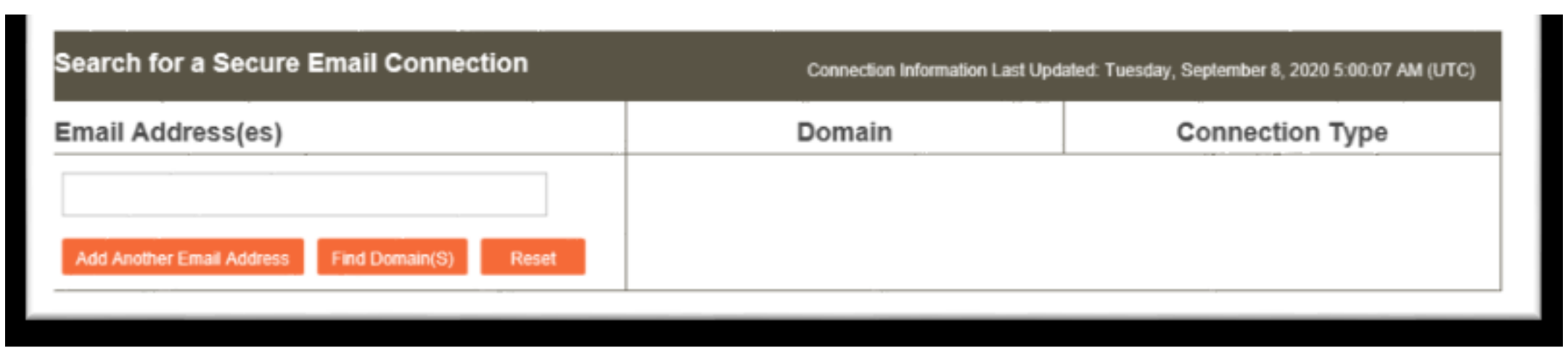

- Prior to starting remote SDV the CRA ensures that the provided documents are complete and does not contain any personal information.
  - In case the CRA detects any personal information that has not been redacted, the CRA informs the study site and deletes the files (incl. the Recycle bin).
  - A Data Breach must be reported Data Breach Web Report Form.
- Use of an external PC screen is recommended. The CRA will not generate any copies from the source data received.
- Source data verification/review will be conducted according to the process outlined in the GSK Monitoring SOP.
- After completion of SDV activities, the CRA deletes all copies/images of participant data received from the site. This includes the deletion of the recycle bin and any temporary files.
- A statement confirming that all documents were destroyed will be provided by the CRA via email to the site.
- Details of what was monitored remotely will be documented in the appropriate section of the Monitoring Visit Report.

Option 2 – Review of Subject Source Documentation remotely

Process for use of Webcams, WebEx, MS Teams for viewing participant source remotely:

- The CRA ensures that the site personnel sharing information with GSK have authority to do so.
- Remote SDV activities will be performed exclusively by the assigned site monitor.
- Prior to conducting any remote SDV activities the CRA ensures that a written informed consent, covering the proposed SDV activities, has been signed by the study patient.
- For CRAs using GSK laptops, only use GSK approved video conferencing tools (e.g., MS Teams or GSK WebEx). Live image transmission is fully encrypted and protected for authorized user. By using these systems, it will be assured that data will be viewed only but not transmitted/stored.
- FSP/Local CRO CRAs not using GSK laptops, only MS Teams via Remote Access Application may be used for meetings between the CRA and the site. WebEx is not permitted from non-GSK laptops. Other tools like FaceTime, WhatsApp or Zoom are not permitted since they do not have sufficient encryption features. GSK does not have enterprise contract/privacy agreement with these providers.
- Prior to the remote monitoring visit, the CRA instructs study site on the specific data needed for the remote SDV.
- Source data verification will be conducted according to the process outlined in the Monitoring SOP.
- The use of a headset is required, do not use computer audio.
- The CRA does not capture screens or take pictures of screens to ensure we are not transferring content outside of clinical sites.
- WebEx or Teams do not store or have access to any data, GSK staff is not allowed to make or store any screenshots or save any data which has been shared.
- Details of what was monitored remotely will be documented in the appropriate section of the Monitoring Visit Report.
- In case of technical malfunctions or if the security of the transmission is no longer ensured, we will pause rSDV activities. GSK Issue Management Procedures will be initiated.

10.6.2. Japan

Regulatory and ethical considerations

The study will be conducted in accordance with "the Ministerial Ordinance on the Standards for the Conduct of Clinical Trials of Medicinal Products (MHW Notification No.28 dated 27 March 1997)" and Pharmaceuticals and Medical Devices Act.

The statement "To assume responsibility for the proper conduct of the study at this site." on the Protocol Investigator Agreement Page means the investigator's responsibility defined by Japanese GCP.

GSK will submit the clinical trial notification to the regulatory authorities in accordance with Pharmaceuticals and Medical Devices Act before conclusion of any contract for the conduct of the study with study sites.

Study period

Study Period is included in Exhibit 1.

Study administrative structure

Sponsor information is included in Exhibit 1. List of Medical Institutions and Investigators is included in Exhibit 2.

10.6.3. South Korea

Exclusion criteria for enrollment of study participant

As per local Regulation on Approval for Enforcement Regulation on the Safety of Pharmaceuticals, etc., Article 24 paragraph 2, a study participant cannot be enrolled in a trial if he or she has "participated as a subject in a clinical trial targeting healthy people within the last 6 months."

Therefore, in order to comply with this local requirement, investigators from Republic of Korea should carefully check, before enrolling each participant in the study, that they have not participated in any other clinical study targeting healthy people within the last 6 months prior to enrollment/first study intervention administration (Refer Section 5.2.3).

Participants from South Korea are required to be aged at least 19 years or greater for entry into the study.

10.7. Appendix 7: Protocol amendment history

The Protocol Amendment Summary of Changes Table for the current amendment is located directly before the TOC.

Amendment 1 GER, ITA, SPN-1

This amendment is considered substantial based on the criteria defined in EU Clinical Trial Regulation No 536/2014 of the European Parliament and the Council of the European Union because it significantly impacts the scientific value of the study.

Overall rationale for the Amendment:

Amendment 1 GER, ITA, SPN-1 is a country-specific protocol amendment created in response to Health Authority feedback to provide clarifications to the protocol, including those that impact participant enrollment and site conduct. A description and rationale for all changes is provided in the table below.

List of main changes in the protocol and their rationale:

Section # and title	Description of change	Brief rationale
Definition of terms	Added the definitions for Serious Adverse Reaction (SAR) and Suspected Unexpected Serious Adverse Reactions (SUSAR).	Response to health authority feedback to implement AE definitions according to EU-CTR.
2.1 Study rationale<br>2.2 Background<br>2.3 Benefit/risk assessment	Added market approval information for RSV OA vaccine across countries.	Response to health authority feedback to provide information on market authorization of the study vaccine.
4.3 Justification for dose	Revised the section to indicate that an approved formulation of the vaccine will be used in the study.	Conistency with market authorization of the study vaccine, as requested by health authority.
5.1.1 Inclusion criteria for all participants	Revised inclusion criteria for informed consent to specify that participant must be able to understand the informed consent.	Response to health authority feedback.
5.2.1 Medical conditions	Note added as a clarification for investigator's assessment of potential hypersensitivity of participant to vaccine components.	Response to health authority feedback to indicate Vaccine components that may ease investigator asessment for potential hypersensitivity reactions.
6.9 Prior and concomitant therapy	Text and table pertaining to prohibited medication and their washout period prior to vaccination has been added.	Response to health authority feedback.
8.3.4 Safety monitoring and committee	Modified/deleted text to imply that SOT patients are defined as 'special' but not 'vulnerable' population.	Response to health authority feedback that SOT patients cannot be defined as a 'vulnerable population'.
8.4 Adverse events (AEs), serious adverse events (SAEs), and other safety reporting	Added text pertaining to SUSAR reporting as per local authorities.	Response to health authority feedback to add that SUSARs will be reported to Eudravigilance database.
9.3.1 Primary endpoints/estimands analyses	Added text to imply that missing data will not be imputed.	Response to health authority feedback to add strategy for handling missing data.

Section # and title	Description of change	Brief rationale
Throughout protocol	Administrative and editorial changes were made to update sponsor signatory on cover page, align table numbers, formatting, and crossreferences.	To maintain consistency with template guidance.

11. REFERENCES

Arexvy Prescribing Information. AREXVY (Respiratory Syncytial Virus Vaccine, Adjuvanted) suspension for intramuscular injection, 2023. Accessed in October 2023 at https://gskpro.com/content/dam/global/hcpportal/en_US/Prescribing_Information/Arexvy/pdf/AREXVY.PDF

Arexvy Summary of Product Characteristics. Arexvy powder and suspension for suspension for injection, Respiratory Syncytial Virus (RSV) vaccine (recombinant, adjuvanted), 2023. Accessed in October 2023 at https://www.ema.europa.eu/en/documents/overview/arexvy-epar-medicine-overview_en.pdf

Bao K, Reinhardt RL. The differential expression of IL-4 and IL-13 and its impact on type-2 immunity. *Cytokine.* 2015;75(1):25-37. doi: 10.1016/j.cyto.2015.05.008.

Barbas CF, Crowe JE, Cababa D, et al. Human monoclonal Fab fragments derived from a combinatorial library bind to respiratory syncytial virus F glycoprotein and neutralize infectivity. *Proc Natl Acad Sci USA*. 1992;89(21):10164-8.

Bates JT, Keefer CJ, Slaughter JC, et al. Escape from neutralization by the respiratory syncytial virus-specific neutralizing monoclonal antibody palivizumab is driven by changes in on-rate of binding to the fusion protein. *Virology*. 2014;454-455:139-44. doi: 10.1016/j.virol.2014.02.010.

Borchers AT, Chang C, Gershwin ME, Gershwin LJ. Respiratory syncytial virus--a comprehensive review. *Clin Rev Allergy Immunol.* 2013;45(3):331-379.

Boyman O, Sprent J. The role of interleukin-2 during homeostasis and activation of the immune system. *Nat Rev Immunol.* 2012;12(3):180-90.

Chapter 1: Definition and classification of CKD. *Kidney Int Suppl* (2011). 2013;3(1):19-62. doi:10.1038/kisup.2012.64

Chattopadhyay PK, Yu J, Roederer M. A live-cell assay to detect antigen-specific CD4+ T cells with diverse cytokine profiles. *Nat Med.* 2005;11(10):1113-7.

Clopper C.J., Pearson E., The Use of Confidence or Fiducial Limits Illustrated in the case of the Binomial. *Biometrika.* 1934; 26: 404-13.

Dropulic LK, Lederman HM. Overview of Infections in the Immunocompromised Host. *Microbiol Spectr.* 2016;4(4).

Fishman JA, Rubin RH. Infection in organ-transplant recipients. *N Engl J Med.* 1998;338(24):1741-1751.

Frentsch M, Arbach O, Kirchhoff D, et al. Direct access to CD4+ T cells specific for defined antigens according to CD154 expression. *Nat Med.* 2005;11(10):1118-24.

Haralambieva IH, Ovsyannikova IG, Pankratz VS, et al. The genetic basis for interindividual immune response variation to measles vaccine: new understanding and new vaccine approaches. *Expert Rev Vaccines*. 2013;12(1):57–70.

Kollmann TR. Variation between populations in the innate immune response to vaccine adjuvants. *Front Immunol*. 2013;4:81. doi: 10.3389/fimmu.2013.00081.

Korn T, Bettelli E, Oukka M, et al. IL-17 and Th17 Cells. *Annu Rev Immunol.* 2009;27:485-517.

Levey AS, Eckardt KU, Tsukamoto Y, et al. Definition and classification of chronic kidney disease: a position statement from Kidney Disease: Improving Global Outcomes (KDIGO). *Kidney Int*. 2005;67(6):2089-100. doi:10.1111/j.1523-1755.2005.00365.x

Matsuo S, Imai E, Horio M, et al. Revised equations for estimated GFR from serum creatinine in Japan. *Am J Kidney Dis*. 2009;53(6):982-92. doi:10.1053/j.ajkd.2008.12.034

McDonald JU, Rigsby P, Dougall T, et al. Establishment of the first WHO International Standard for antiserum to Respiratory Syncytical Virus: Report of an International collaborative study. *Vaccine*. 2018;36(50):7641-7649. doi: 10.1016/j.vaccine.2018.10.087.

McDonald JU, Rigsby P, Dougall T, et al. Expansion of the 1st WHO international standard for antiserum to respiratory syncytial virus to include neutralisation titres against RSV subtype B: An international collaborative study. *Vaccine*. 2020;38(4):800-807. doi: 10.1016/j.vaccine.2019.10.095.

Moris P, Van Der Most R, Leroux-Roels I, et al. H5N1 influenza vaccine formulated with AS03 A induces strong cross-reactive and polyfunctional CD4 T-cell responses. *J Clin Immunol.* 2011; 31(3):443-54.

Neemann K, Freifeld A. Respiratory Syncytial Virus in hematopoietic stem cell transplantation and solid-organ transplantation. *Curr Infect Dis Rep*. 2015; 17(7):490. doi:10.1007/s11908-015-0490-9

Papi A, Ison MG, Langley JM, et al. Respiratory Syncytial Virus Prefusion F Protein Vaccine in Older Adults. *N Engl J Med*. 2023;388(7):595-608. doi:10.1056/NEJMoa2209604

Pérez-Losada M, Posada D, Arenas M, et al. Ethnic differences in the adaptation rate of HIV gp120 from a vaccine trial. *Retrovirology*. 2009;6:67.

Pochon C, Voigt S. Respiratory Virus Infections in Hematopoietic Cell Transplant Recipients. *Front Microbiol.* 2019;9:3294.

Reichenberger F, Dickenmann M, Binet I, et al. Diagnostic yield of bronchoalveolar lavage following renal transplantation. *Transpl Infect Dis.* 2001;3(1):2-7.

Roden AC, Aisner DL, Allen TC, et al. Diagnosis of Acute Cellular Rejection and Antibody-Mediated Rejection on Lung Transplant Biopsies: A Perspective From Members of the Pulmonary Pathology Society. *Arch Pathol Lab Med*. 2017;141(3):437-44. doi:10.5858/arpa.2016-0459-SA

Samten B, Thomas EK, Gong J, Barnes PF. Depressed CD40 ligand expression contributes to reduced gamma interferon production in human tuberculosis. *Infect Immun.* 2000;68(5):3002-6.

Schoenborn JR, Wilson CB. Regulation of interferon-gamma during innate and adaptive immune responses. *Adv Immunol.* 2007;96:41-101.

Sedger LM, McDermott MF. TNF and TNF-receptors: From mediators of cell death and inflammation to therapeutic giants - past, present and future. *Cytokine Growth Factor Rev.* 2014;25(4):453-72.

Smith L. New AHA Recommendations for Blood Pressure Measurement. *Am Fam Physician*. 2005;72(7):1391-8.

Stubbe M, Vanderheyde N, Goldman M, et al. Antigen-specific central memory CD4+ T lymphocytes produce multiple cytokines and proliferate in vivo in humans. *J Immunol.* 2006;177(11):8185-90.

Verleden GM, Glanville AR, Lease ED, et al. Chronic lung allograft dysfunction: Definition, diagnostic criteria, and approaches to treatment-A consensus report from the Pulmonary Council of the ISHLT. *J Heart Lung Transplant*. 2019;38(5):493-503. doi:10.1016/j.healun.2019.03.009

Vink P, Ramon Torrell JM, Sanchez Fructuoso A, et al. Immunogenicity and Safety of the Adjuvanted Recombinant Zoster Vaccine in Chronically Immunosuppressed Adults Following Renal Transplant: A Phase 3, Randomized Clinical Trial. *Clin Infect Dis.* 2020;70(2):181-90.

Wölfl M, Kuball J, Eyrich M, et al. Use of CD137 to study the full repertoire of CD8+ T cells without the need to know epitope specificities. *Cytometry A.* 2008;73(11):1043-9.

Signature Page for 219900 TMF-17016141 v1.0

Reason for signing: Approved	Name: PPD<br>Role: A<br>Date of signature: 11-Oct-2023 07:09:45 GMT+0000

Reason for signing: Approved	Name: PPD<br>Role: A<br>Date of signature: 11-Oct-2023 13:26:58 GMT+0000

Signature Page for TMF-17016141 v1.0
