# Supplementary material for: AS01E-adjuvanted RSV pre-F protein vaccine in adults with lung or kidney transplant: a phase 2b randomized trial
Source: Nat Commun. 2026 Nov 13;17:11678. doi: 10.1038/s41467-026-78584-9 (PMC13768339; doi:10.1038/s41467-026-78584-9)
Supplement: Supplementary file 2 [file 41467_2026_78584_MOESM2_ESM.pdf]

nature portfolio

Corresponding author(s): Veronica Hulstrøm

Last updated by author(s): 04/09/2026

Reporting Summary

Nature Portfolio wishes to improve the reproducibility of the work that we publish. This form provides structure for consistency and transparency in reporting. For further information on Nature Portfolio policies, see our Editorial Policies and the Editorial Policy Checklist.

Statistics

For all statistical analyses, confirm that the following items are present in the figure legend, table legend, main text, or Methods section.

n/a	Confirmed	
☐	☒	The exact sample size (n) for each experimental group/condition, given as a discrete number and unit of measurement
☐	☒	A statement on whether measurements were taken from distinct samples or whether the same sample was measured repeatedly
☐	☒	The statistical test(s) used AND whether they are one- or two-sided<br>*Only common tests should be described solely by name; describe more complex techniques in the Methods section.*
☐	☒	A description of all covariates tested
☐	☒	A description of any assumptions or corrections, such as tests of normality and adjustment for multiple comparisons
☐	☒	A full description of the statistical parameters including central tendency (e.g. means) or other basic estimates (e.g. regression coefficient) AND variation (e.g. standard deviation) or associated estimates of uncertainty (e.g. confidence intervals)
☒	☐	For null hypothesis testing, the test statistic (e.g. F, t, r) with confidence intervals, effect sizes, degrees of freedom and P value noted<br>*Give P values as exact values whenever suitable.*
☒	☐	For Bayesian analysis, information on the choice of priors and Markov chain Monte Carlo settings
☒	☐	For hierarchical and complex designs, identification of the appropriate level for tests and full reporting of outcomes
☒	☐	Estimates of effect sizes (e.g. Cohen's d, Pearson's r), indicating how they were calculated

Our web collection on statistics for biologists contains articles on many of the points above.

Software and code

Policy information about availability of computer code

Data collection	Study case report forms were collected using Veeva EDC software (Veeva Systems Inc, Pleasanton, CA, US). The EDC system went GoLive for this study under Veeva release 23R1.
Data analysis	All analyses were conducted using SAS Viya 3.5 software (SAS Institute Inc., Cary, NC, US).

For manuscripts utilizing custom algorithms or software that are central to the research but not yet described in published literature, software must be made available to editors and reviewers. We strongly encourage code deposition in a community repository (e.g. GitHub). See the Nature Portfolio guidelines for submitting code & software for further information.

Data

Policy information about availability of data

All manuscripts must include a data availability statement. This statement should provide the following information, where applicable:

- Accession codes, unique identifiers, or web links for publicly available datasets
- A description of any restrictions on data availability
- For clinical datasets or third party data, please ensure that the statement adheres to our policy

Not all participant-level data generated or analyzed during this study are publicly available because they contain information that could compromise participant privacy or consent. Where appropriate, data underlying the figures and tables are provided in the Source Data file; however, data rows have been randomized to minimize the risk of participant re-identification. Additional de-identified participant data generated and analyzed during this study can be requested via Vivli - Center for Global Clinical Research Data. Requests are reviewed by an independent panel within 90 calendar days of receipt of a complete proposal, and if

approved, researchers will be granted access to anonymized data in a secure environment after signing a Data Sharing Agreement (DSA). Additional guidance is available via https://www.gsk-studyregister.com/gsk-patient-level-data-sharing-july2025.pdf.

Research involving human participants, their data, or biological material

Policy information about studies with human participants or human data. See also policy information about sex, gender (identity/presentation), and sexual orientation and race, ethnicity and racism.

Reporting on sex and gender	Study recruitment included both males and females. Participant sex was collected and recorded as biological sex, as determined and documented by the investigator in the electronic case report form, in accordance with the study protocol. Sex was not assigned experimentally, nor was gender identity specifically collected as a separate variable in this study. The distribution of sexes of enrolled participants is reported in Table 1. The study did not intend to conduct specific analyses investigating the relationship between sex and the safety and immunogenicity of the vaccine. In line with the study design and sample size considerations, results are presented in aggregate. Formal sex-based comparative analyses were therefore not conducted, as there would be limited ability to draw meaningful or robust conclusions.
Reporting on race, ethnicity, or other socially relevant groupings	Race and ethnicity data were collected to assess and monitor the diversity of the trial participants, and to determine if the trial participants are representative of the impacted population. To minimize the risk of participant identification, only race categories including a minimum of 11 participants are described in the publication; other race categories were pooled (Table 1).<br>Race and ethnicity were self-reported. The study did not control for race or ethnicity as potential confounding variables in its analyses.
Population characteristics	The study enrolled two populations: immunocompromised individuals, specifically lung or kidney transplant recipients aged ≥18 years who were at least one-year post-transplant and received maintenance therapy for prevention of allograft rejection, and non-immunocompromised adults ≥50 years, who served as controls. Detailed eligibility criteria are provided in the Supplement, and participant characteristics are provided in Table 1.
Recruitment	Participants were recruited at 48 sites across 8 countries. Immunocompromised participants (renal and lung solid organ transplant recipients) were recruited through clinical sites and transplant centers, where eligible individuals were identified by investigators based on the protocol inclusion and exclusion criteria. Non-immunocompromised participants were recruited from the general population at participating sites. All participants provided written informed consent prior to enrollment.<br>As participation required consent and willingness to comply with study procedures, there is potential for self-selection bias, whereby individuals who are more health-conscious, motivated, or engaged with healthcare systems may have been more likely to participate. In addition, the study design may introduce other potential sources of bias. The non-immunocompromised control group was not randomized and received only 1 dose of the vaccine, however, the inclusion and exclusion criteria were aligned to those used in the pivotal vaccine efficacy study. Recruitment through specialized transplant centers may also result in a population with closer clinical monitoring and differing baseline health status compared to the broader transplant population.<br>These factors are not expected to affect immunogenicity and safety assessments overall.
Ethics oversight	1. Metro North Health Human Research Ethics Committee, Herston, Australia<br>2. University of British Columbia Clinical Research Ethics Board, Vancouver, Canada<br>3. University of Alberta Human Research Ethics Board, Edmonton, Canada<br>4. Advarra, Aurora, Canada<br>5. University Health Network Research Ethics Board, Toronto, Canada<br>6. Western Health Sciences Research Ethics Board, London, Canada<br>7. Ethikkommission der Universität Duisburg-Essen, Langen, Germany<br>8. CET Interaziendale AOU Maggiore della Carità di Novara, Novara, Italy<br>9. Nagoya Daini Red Cross Hospital Institutional Review Board, Nagoya, Japan<br>10. Tokyo Medical University Hachioji Medical Center Institutional Review Board, Tokyo, Japan<br>11. The Central Institutional Review Board for the Fujita Health University Hospitals, Toyoake, Japan<br>12. Hyogo Prefectural Nishinomiya Hospital Institutional Review Board, Nishinomiya, Japan<br>13. Fukuoka University Hospital Institutional Review Board, Fukuoka, Japan<br>14. Okayama University Hospital Institutional Review Board, Okayama, Japan<br>15. Medical Corporation Shinanokai Shinanosaka Clinic Institutional Review Board, Tokyo, Japan<br>16. Japanese Red Cross Kumamoto Hospital Institutional Review Board, Kumamoto, Japan<br>17. Seoul National University Hospital Institutional Review Board, Seoul, Republic of Korea<br>18. Yonsei University Health System, Severance Hospital, Institutional Review Board, Seoul, Republic of Korea<br>19. Asan Medical Center Institutional Review Board, Seoul, Republic of Korea<br>20. CEIm Hospital Clínico San Carlos, Madrid, Spain<br>21. WCG IRB, Princeton, United States<br>22. University of Kentucky Office of Research Integrity, Lexington, United States<br>23. Baylor Scott & White Research Institute, Dallas, United States<br>24. Columbia University Institutional Review Board, New York, United States<br>25. University of Minnesota Institutional Review Board, Minneapolis, United States

Note that full information on the approval of the study protocol must also be provided in the manuscript.

Field-specific reporting

Please select the one below that is the best fit for your research. If you are not sure, read the appropriate sections before making your selection.

- [x] Life sciences
- [ ] Behavioural & social sciences
- [ ] Ecological, evolutionary & environmental sciences

For a reference copy of the document with all sections, see nature.com/documents/nr-reporting-summary-flat.pdf

Life sciences study design

All studies must disclose on these points even when the disclosure is negative.

Sample size

The descriptive nature of the study and prior immunogenicity results with adjuvanted RSVPreF3 informed the overall sample size. A power calculation was performed during study design to inform sample size determination and to ensure that meaningful differences in immune responses for the primary objective could be detected. The primary study objective (i.e., comparing the response post-dose 2 to post-dose 1 in the 2-dose transplant group) was specified with 100 evaluable participants. Analyzing a paired t-test with 1-sided alpha of 2.5% and a standard deviation of differences of 0.5 (inflated from 0.4 to account for the immunocompromising conditions), the detectable fold-increases were 1.39 with 80% power and 1.46 with 90% power. With a standard deviation of differences of 0.4, the detectable fold-increases were 1.30 with 80% power and 1.35 with 90% power. These calculations were intended to support study design, rather than to define formal success thresholds.

Data exclusions

Participants were excluded from the per-protocol set for humoral immunogenicity when they did not receive the study intervention administration as per protocol, when pre- and post-dose immunogenicity results were not available, when they did not comply with blood draw intervals, when they had intercurrent conditions that may interfere with immunogenicity, or when they received prohibited medication or vaccination (according to the prespecified analysis plan). No participants were excluded from the safety analyses.

Replication

Double programming was performed for primary and major secondary endpoints as a QC check and results were confirmed.

Randomization

The randomization strategy in the transplant recipients in two dosing groups in a 1:1 ratio employed a permuted block randomization method, with stratification by transplant type and cell-mediated immunogenicity subset, using a GSK Randomization and Trial Supply Management (RTSM) system. The RTSM system managed recruitment caps for the transplant types to maintain balance.

Blinding

This was an open-label study; all participants received at least one dose of the vaccine. For the 1-dose and 2-dose transplant groups, the primary descriptive immunogenicity endpoint is not affected by the open-label design.

Reporting for specific materials, systems and methods

We require information from authors about some types of materials, experimental systems and methods used in many studies. Here, indicate whether each material, system or method listed is relevant to your study. If you are not sure if a list item applies to your research, read the appropriate section before selecting a response.

Materials & experimental systems

n/a	Involved in the study	
	x	Antibodies
x		Eukaryotic cell lines
x		Palaeontology and archaeology
x		Animals and other organisms
	x	Clinical data
x		Dual use research of concern
x		Plants

Methods

n/a	Involved in the study	
x		ChIP-seq
	x	Flow cytometry
x		MRI-based neuroimaging

Antibodies

Antibodies used

CD3, fluorochrome BV605 (BioLegend, cat. no.: 317322, clone: OKT3)
CD4, fluorochrome BB700 (BD, cat. no.: 566392, clone: SK3)
CD8, fluorochrome APC-H7 (BD, cat. no.: 641400, clone: SK1)
IL-2, fluorochrome APC-R700 (Pharmingen, cat. no.: 565136, clone: MQ1-17H12)
TNFα, fluorochrome PeCy7 (Pharmingen, cat. no.: 557647, clone: Mab11)
IFNγ, fluorochrome FITC (Pharmingen, cat. no.: 554551, clone: 4S.B3)
IL-13, fluorochrome APC (BioLegend, cat. no.: 501907, clone: JES10-5A2)
IL-17, fluorochrome BV785 (BioLegend, cat. no.: 512338, clone: BL168)
CD40L, fluorochrome PE (Miltenyi Biotech, cat. no.: 130-113-613, clone: REA238)
4-1BB, fluorochrome BV421 (BioLegend, cat. no.: 309820, clone: 4B4-1)

Validation

Specificity of each antibody used for flow cytometry was confirmed during assay development through a Fluorescence Minus One experiment, ensuring the signal detected in the dedicated channel was only present when the antibody was present and that it did

not impact the fluorescence in the other channels. In addition, each antibody lot was titrated to ensure the use at optimal dilution, identified as providing the best signal-to-noise ratio and stain index.

Clinical data

Policy information about clinical studies

All manuscripts should comply with the ICMJE guidelines for publication of clinical research and a completed CONSORT checklist must be included with all submissions.

Clinical trial registration	NCT05921903
Study protocol	https://www.gsk-studyregister.com/en/trial-details/?id=219900
Data collection	This multicountry study was conducted across 48 sites (clinical sites and transplant centers) in Australia, Canada, Germany, Italy, Japan, Republic of Korea, Spain, and the United States from July 28, 2023, to May 16, 2025. Participant recruitment took place between 28 July 2023 and 22 March 2024.
Outcomes	The primary outcomes were RSV-A and RSV-B serum neutralizing titers, expressed as mean geometric increase post-dose 2 over post-dose 1 in kidney or lung solid organ transplant patients in the 2-dose transplant group.<br>Secondary outcomes included RSV-A and RSV-B serum neutralizing titers expressed as GMT at different timepoints in all groups, cell-mediated immune response (assessed as the frequency of RSVPreF3-specific CD4+ and/or CD8+ T cells expressing at least 2 activation markers, including at least one cytokine among CD40L, 4-1BB, IL-2, TNF-α, IFN-γ, IL-13, and IL-17) at different timepoints in a subset of participants from all groups, and safety (assessed as the incidence and severity of adverse events) in all groups.

Plants

Seed stocks	NA
Novel plant genotypes	NA
Authentication	NA

Flow Cytometry

Plots

Confirm that:

- [x] The axis labels state the marker and fluorochrome used (e.g. CD4-FITC).
- [x] The axis scales are clearly visible. Include numbers along axes only for bottom left plot of group (a 'group' is an analysis of identical markers).
- [x] All plots are contour plots with outliers or pseudocolor plots.
- [x] A numerical value for number of cells or percentage (with statistics) is provided.

Methodology

Sample preparation	Peripheral blood mononuclear cells (PBMC) were stimulated in vitro either with a pool of 15-mer peptides overlapping by 11 amino acids and spanning the RSVPreF3 protein sequence, or with medium alone as negative control (background condition). Culture medium contained anti-CD28/anti-CD49d costimulatory antibodies (Pharmingen, cat. no. 555725 and 555501, respectively). In peptide-stimulated wells, the final DMSO dilution was 1/1600. After an initial 2-hour incubation at 37°C, Brefeldin A (GolgiPlug, Pharmingen) was added to inhibit cytokine secretion for an additional 18 hours (total incubation time: 20 hours). Cells were subsequently harvested, stained for surface markers (CD4 and CD8) together with a viability dye (L&D Aqua, Invitrogen), and then fixed and permeabilized (CellFix/CellPerm, Pharmingen). Permeabilized cells were stained with labelled antibodies specific for CD3, CD40 ligand, 4-1BB, interleukin-2, tumor necrosis factor-α, interferon-ɣ, interleukin-13, and interleukin-17.
Instrument	Data acquisition was performed on BD flow cytometers (Fortessa X20, Symphony A3 or A5).
Software	Data analysis was performed with FlowJo v09 (BD).
Cell population abundance	There was no cell sorting applied to perform the intracellular cytokine staining assay, which was run on total PBMC. To ensure quality data, the PBMC had to contain at least 80% viable cells at thawing to be engaged in the assay.
Gating strategy	Events were sequentially gated as follows: (1) a time gate was created to ensure that the events were captured once the flow stream was stabilized (side scatter-area [SSC]-A versus time density plot); (2) a gate was made on singlets (doublets were

excluded [forward scatter-height [FSC]-H versus FSC-A plot]); (3) a gate was made on live cells (dead cells were excluded [live-dead dye parameter versus SSC-A plot]); (4) a lymphocyte gate was created based on size and granularity (FSC-height [FSC-H] [size] versus SSC-A [granularity] plot); (5) CD3+ T cells were gated (CD3 versus IFN-γ plot; as the CD3 marker expression is downregulated after stimulation, a polygon gate was used); (6) CD4+ (CD4+CD8-) and CD8+ (CD8+CD4-) T cells were then gated (CD4 versus CD8 plot); and finally (7) activation markers (SSC-A versus CD40L [CD154], IL-2, IL-13, IL17, TNF-α, IFN-γ and 4-1BB [CD137], respectively) were assessed in both CD4+CD8- T cells and CD8+CD4- T cells.

A Boolean gating analysis was used to identify all combinations of T cells expressing at least two activation markers, including at least one cytokine, which were summed to generate polypositive T-cell counts. These counts were subsequently expressed as frequencies of polypositive T cells per million CD4+ or CD8+ T cells. Polypositive T-cell frequencies obtained under background conditions were subtracted from those obtained under stimulated conditions, to calculate RSVPreF3-specific polypositive T-cell frequencies per million CD4+ or CD8+ T cells.

☒ Tick this box to confirm that a figure exemplifying the gating strategy is provided in the Supplementary Information.
