# Supplementary material for: AS01E-adjuvanted RSV pre-F protein vaccine in adults with lung or kidney transplant: a phase 2b randomized trial
Source: Nat Commun. 2026 Nov 13;17:11678. doi: 10.1038/s41467-026-78584-9 (PMC13768339; doi:10.1038/s41467-026-78584-9)
Supplement: Supplementary file 3 [file 41467_2026_78584_MOESM3_ESM.pdf]

nature portfolio

Peer Review File

AS01E-adjuvanted RSV pre-F protein vaccine in adults with lung or kidney transplant: a phase 2b randomized trial

Corresponding Author: Dr Veronica Hulstrom

This manuscript has been previously reviewed at another journal. This document only contains information relating to versions considered at Nature Communications.

This file contains all reviewer reports in order by version, followed by all author rebuttals in order by version.

Version 0:

Reviewer comments:

Reviewer #1

(Remarks to the Author)
This manuscript by Small and colleagues report data from a phase 2b RCT on the immunogenicity and safety of an AS01E – adjuvanted RSV preF protein vaccine among immunocompromised adults with lung or kidney transplants. These findings are of interest as there is limited data on RSV vaccines in this particular population group who are at higher risk of severe RSV disease and who generally respond poorer to vaccines. The authors have provided results on RSVA/B neutralizing antibodies as well as cell-mediated immunity in a small subset of participants. Reactogenicity and safety is also reported.

This is a well-written manuscript with interesting findings however requires some further clarifications and revisions before it can be considered for publication.

1. Comparisons with the non-randomized non-immunocompromised control group should be interpreted with caution. Was there a reason why this group was non-randomized? While this group provides an important comparator there may be several confounders not accounted for that impact on the results.
2. The non-immunocompromised group is stated as >50y of age while the immunocompromised groups were >18y of age. In Table 1, the age categories are 18-59y and >60y so this is a bit confusing. Ie 35.2% of the non-immunocompromised group were in the 18-59y but they would all be >50y – I think it would be helpful to also include the actual median age (and IQR) for these categories.
3. Reactogenicity and AEs appears slightly elevated in the non-immunocompromised group overall compared to both transplant groups – any thoughts on this?
4. Further to this, comparison of immunogenicity based on these age cutoffs are worthwhile. The authors note that this is limited by the small sample size but there are approximately 80 participants >60y across the groups which would allow for meaningful comparisons.
5. Supp Table 2 includes information on various co-morbidities across the 3 groups. For some categories e.g. metabolism and nutrition, vascular disorders, there are ~35% in the non-immunocompromised group. How does this impact on immunogenicity compared to those without these co-morbidities?
6. Can the authors provide information on the breakdown on recruitment by site? There were 48 sites across 8 countries. Were there any obvious differences or skewness to any particular site/country?
7. As the study was conducted over at least a 12m period for each participant, including at least 1 RSV season, were any RSV infections recorded, either by PCR/RAT testing or serology, especially between Visit 5 and Visit 6 (6-12m period)? This would be interesting to know and likely to impact immunogenicity data at these later timepoints. Line 155 states that there were no intercurrent conditions but what does this refer to? RSV infection or any other medical condition?
8. What was the lost-to-follow-up rate overall and by study group?
9. The MGI for RSVA and RSVB were lower than the expected rates for which a sample size with 80% power was determined. Can the authors comment on the interpretation of this result?
10. How did the immunogenicity compare between 2 dose recipients given between the 30-60 day interval. It is well recognised that immunogenicity is higher when given over a longer interval so this information would be interesting to know.
11. Please indicate the RSV neutralizing assay cutoffs on the relevant Figures. Why was ED60 chosen?
12. For CMI analyses, how was this subset determined? How does this cohort compare with the immunogenicity cohort?

Some more details are needed here
13. In general the details of the methods are scarce, especially for the CMI analysis. Some more details are needed, including representative gating strategies. In particular how the data was calculated in terms of per million PBMCs – it is more common to represent this as % of the parent population of interest.
14. It is not clear what the difference is between Extended data which appears in the main manuscript and Supplementary data. I think Extended data should go into the Supplementary section.
15. I could not find Extended data Figure 2 – only the Supplementary Tables 4 and 5.
16. The authors state that mycophenolate had no impact on CD4 T cell frequencies (although data is presented in actual numbers) – however it does appear that T cell levels were higher among MC group, compared to the lower antibody responses in the MC group. What is the explanation for this?

Reviewer #2

(Remarks to the Author)
The manuscript "Immunogenicity and safety of the AS01E-adjuvanted respiratory syncytial virus prefusion F protein vaccine in immunocompromised adults with lung or kidney transplant: a phase 2b randomized clinical trial" reports a phase I clinical trial involving 386 participants (261 transplant participants and 125 non-immunocompromised participants) that evaluated the safety and immunogenicity of the adjuvanted RSVPreF3. The results suggest that a two-dose regime was safe and immunogenic in immunocompromised adults. The study has shown novelty, is important to the related field, and is well presented in general. A few major and minor issues were identified, and suggestions for the authors' consideration are provided below.

Major:
1. Method/Result: This is a multicountry, multicentre trial, and there is potential centre-level and country level correlation in immunity profiles, baseline seropositivity levels and lab assay processing variabilities. As the authors did not mention country or site as randomisation factor, ignoring this may underestimate SE and overestimate precision. Could you please report the number of participants by country/site in each group (baseline characteristics). The authors are encouraged to include country as an additional covariate, and conduct a sensitivity analysis by country to check the consistency.

2. Method/Result: The authors used GMT ratios estimated from ANCOVA with baseline titer as covariates. The authors may wish to consider a mixed model that accounts for the correlation structure of repeated measurements within individuals over multiple timepoints, i.e., the Generalised linear mixed models (GLMM) or generalised estimating equations (GEE) to model the outcome measurements as a function of time, group, and their interaction as fixed effects, site and participant as random effect and adjust for baseline. This method allows missing data handling, different intervention schedules, and between-visit fold rises are also directly estimable.

Minor:
3. Method - Sample size justification: The Statistics section states that the primary objective will be addressed using an estimation approach without hypothesis testing. However, the sample size calculation in the supplementary material is hypothesis-based, using a paired t-test framework with 80% and 90% power targets. The authors may want either provide a precision-based sample size justification, or clarify that hypothesis testing is intended.

4. Method - Definition of cell-mediated subset Line 521-522. It is suggested to define this subset earlier (e.g., at population section). The term was first encountered in the randomisation section, which may confuse readers unfamiliar with this term.

5. Method - Figure 1 and Line 499-500: if the V5 is 180 -210 days since last dose and V6 is 350-380 days since last dose, the total study period for the 2-dose transplant would be 387-440 days (13-15 months), and 1-dose transplant and 1-dose non-immunocompromised group should share the same total study period (12 month). However at Line 499-500, it says "13–15 months for transplant recipients and 12 months for non-immunocompromised participants".

6. Method - Time window for V3. 30-60 days post first vaccination appears to be a relatively wide window compared to V4. Titres at day 30 and day 60 could be meaningfully different due to natural waning, and potentially introducing heterogeneity within this visit window. The authors are encouraged to provide a distribution of visit timing at V3 across groups, and potentially provide justification for this chosen window.

7. Result/Discussion: There is a gap between the wording in the result and the analysis conducted. For example, Line 233-241/Line 352-353, comparative claims are made: "GMT increased after the second dose". The GMT at V3 and V4 for MC participants had overlapping CI, so "GMTs further increased after the second dose" may not be a valid description. As the current analysis results are by-visit GMT and MGI compared to baseline, the claim of between-visit differences should be based on the between-visit fold rise with CI. In addition, "A second dose administered…, bringing them close to non-immunocompromised levels" would require between group GMR (CI) evidence to support this claim, and this is a relatively strong causal claim. This type of comparative wording should be double-checked throughout the manuscript.

8. Result - Table 2: As the N refers to number of exposed participants in each group, please explain the 2-dose transplant group post-dose 1 column shows N=128 for solicited events and N=130 for unsolicited AEs.

Open Access This Peer Review File is licensed under a Creative Commons Attribution 4.0 International License, which permits use, sharing, adaptation, distribution and reproduction in any medium or format, as long as you give appropriate credit to the original author(s) and the source, provide a link to the Creative Commons license, and indicate if changes were made.
In cases where reviewers are anonymous, credit should be given to 'Anonymous Referee' and the source.
The images or other third party material in this Peer Review File are included in the article’s Creative Commons license, unless indicated otherwise in a credit line to the material. If material is not included in the article’s Creative Commons license and your intended use is not permitted by statutory regulation or exceeds the permitted use, you will need to obtain permission directly from the copyright holder.
To view a copy of this license, visit https://creativecommons.org/licenses/by/4.0/

Point-by-point responses

We sincerely thank the reviewers for their careful evaluation of our manuscript and for their constructive comments. We have revised the manuscript accordingly and provided detailed, point-by-point responses below, describing how each comment has been addressed. All changes made to the manuscript are shown using tracked changes and are highlighted throughout the manuscript for ease of review. The line and page numbers cited in the responses correspond to those shown in "All markup" mode. We believe that these revisions have strengthened the manuscript and improved the overall quality of the work.

In addition, we would like to bring to the reviewers' attention that, during the revision of this manuscript, questions from a regulatory authority prompted additional subject-level assessments and investigator follow-up regarding a small number of medically attended adverse events (AEs) reported during the trial, for which hospitalization had been recorded in the healthcare encounter dataset. Based on the additional information obtained and reassessment against protocol-defined seriousness criteria, three vaccine-unrelated AEs were reclassified from non-serious AEs to serious AEs (SAEs), while the remaining AEs retained their original classification. These updates reflect a refinement of event classification rather than the identification of new safety findings. The study datasets, analyses, and the corresponding manuscript results and tables have been updated accordingly. Importantly, these changes did not affect the overall interpretation of the safety data or the study conclusions. The $AS01_E$-adjuvanted RSVPreF3 vaccine continued to demonstrate an acceptable reactogenicity and safety profile across all study groups, including the 1-dose and 2-dose transplant groups and the non-immunocompromised group of adults aged ≥50 years.

We also want to bring to your attention that we have now included a list of all Internal Review Boards/Ethics Committees in the Supplemental Material and in the Number Reporting Summary. We are currently ensuring we obtain all the necessary permissions for the disclosures should the manuscript be deemed acceptable for publication.

Reviewer #1:

This manuscript by Small and colleagues report data from a phase 2b RCT on the immunogenicity and safety of an AS01E – adjuvanted RSV preF protein vaccine among immunocompromised adults with lung or kidney transplants. These findings are of interest as there is limited data on RSV vaccines in this particular population group who are at higher risk of severe RSV disease and who generally respond poorer to vaccines. The authors have provided results on RSVA/B neutralizing antibodies as well as cell-mediated immunity in a small subset of participants. Reactogenicity and safety is also reported.

This is a well-written manuscript with interesting findings however requires some further clarifications and revisions before it can be considered for publication.

1. Comparisons with the non-randomized non-immunocompromised control group should be interpreted with caution. Was there a reason why this group was non-randomized? While this group provides an important comparator there may be several confounders not accounted for that impact on the results.

Response: We thank the reviewer for this important comment and agree that comparisons with the non-randomized non-immunocompromised control group should be interpreted with caution. We acknowledge that comparisons between immunocompromised and non-immunocompromised groups may be subject to confounding due to differences in baseline characteristics; therefore, these analyses are descriptive and exploratory in nature.

The non-immunocompromised control group was included as a contextual, exploratory benchmark for immunogenicity and safety rather than as a randomized comparator. As this group represents a fundamentally different population from the immunocompromised cohort, randomization across these populations was not appropriate for the study objectives. Instead, the non-immunocompromised control group enabled contemporaneous comparison under consistent study conditions, minimizing variability related to factors such as RSV seasonality and assay methodology.

This design is further supported by prior evidence in non-immunocompromised adults (Leroux-Roels et al, *J Infect Dis* 2023), in whom no additional immunogenic benefit was observed following a second dose administered two months after the first. This finding supported development of a single-dose primary regimen in that population and underscores the role of the single-dose non-immunocompromised control group in the present study as a contextual reference rather than as a comparator to assess dose effects.

We have added text to the Introduction (lines 96-99) describing this prior evidence demonstrating no additional immunogenic benefit from a second dose in non-immunocompromised adults. This clarification helps contextualize the role of the non-immunocompromised control group in the present study as a descriptive benchmark.

Reference: Leroux-Roels I, Davis MG, Steenackers K, et al. Safety and immunogenicity of a respiratory syncytial virus prefusion F (RSVPreF3) candidate vaccine in older adults: Phase 1/2 randomized clinical trial. *J Infect Dis*, 2023; 227(6): 761-72.

2. The non-immunocompromised group is stated as >50y of age while the immunocompromised groups were >18y of age. In Table 1, the age categories are 18-59y and >60y so this is a bit confusing. Ie 35.2% of the non-immunocompromised group were in the 18-59y but they would all be >50y – I think it would be helpful to also include the actual median age (and IQR) for these categories.

Response: To minimize confusion, we have revised the age categories in Table 1 to align with the study inclusion criteria: 18–49 years, 50–59 years, and ≥60 years for the transplant groups, and 50–59 years and ≥60 years for the non-immunocompromised group. The mean overall age per group has been replaced with the median age and interquartile range (IQR) in the first line of the table, as determined by post hoc analysis. In addition, the median age (and IQR) within each applicable age category has now been included to better reflect the age distribution within groups.

3. Reactogenicity and AEs appears slightly elevated in the non-immunocompromised group overall compared to both transplant groups – any thoughts on this?

Response: A slightly higher frequency of reactogenicity and adverse events can be noted in the non-immunocompromised group compared with the transplant groups. This may reflect, in part, a more robust innate and adaptive immune response in immunocompetent individuals, which commonly presents as increased reactogenicity. Differences in baseline characteristics between the non-immunocompromised and immunocompromised populations (including age distribution, co-morbidities, and concomitant medications such as immunosuppressants) may also contribute to the observed differences. However, given the descriptive nature of the study and the lack of formal statistical comparisons, we prefer not to include such speculative interpretations in the manuscript.

4. Further to this, comparison of immunogenicity based on these age cutoffs are worthwhile. The authors note that this is limited by the small sample size but there are approximately 80 participants >60y across the groups which would allow for meaningful comparisons.

Response: We thank the reviewer for this suggestion and agree that evaluation of immunogenicity by age group could be informative. In line with the study protocol, we explored immunogenicity descriptively across pre-defined age strata.

Overall, in both populations (transplant recipients and non-immunocompromised individuals), immune responses tended to be numerically higher in younger participants compared with older participants, particularly when comparing those aged ≥60 years with younger age groups. While these findings are directionally consistent with known age-related differences in immune responsiveness, the analyses remain descriptive. Despite the relatively high number of participants ≥60 years, the study was not powered for formal statistical comparisons between age strata, and potential confounding factors (e.g., underlying conditions, transplant status, and concomitant medications in immunocompromised participants) may also influence these observations.

As such, we consider that adding these data in the manuscript would not meaningfully enhance the interpretation of the results.

5. Supp Table 2 includes information on various co-morbidities across the 3 groups. For some categories e.g. metabolism and nutrition, vascular disorders, there are ~35% in the non-immunocompromised group. How does this impact on immunogenicity compared to those without these co-morbidities?

Response: Participants with stable chronic medical conditions were eligible for the study, reflective of the real-world population for whom the vaccine is intended/approved. In the current study, formal subgroup analyses assessing the impact of individual co-morbidities on immunogenicity were not pre-specified in the protocol or statistical analysis plan. We therefore acknowledge that the present study does not directly address the potential independent effect of specific co-morbidities on the vaccine-induced immune responses.

However, available evidence from previous studies with adjuvanted RSVPreF3 and similar vaccines suggests that baseline characteristics, including the presence or number of co-morbidities, do not meaningfully alter vaccine-induced immune responses. In a prior clinical study in 50–59-year-olds, immunogenicity was consistent in individuals without and those with stable chronic medical conditions leading to an increased risk of severe RSV disease (chronic pulmonary disease, chronic cardiovascular disease, diabetes, and chronic liver or renal disease) (Ferguson et al, *Clin Infect Dis* 2024; Ferguson et al, *Hum Vaccin Immunother* 2025). Similarly, in the pivotal phase 3 efficacy trial in ≥60-year-olds (NCT04886596), vaccine efficacy against RSV-associated disease remained high in participants with cardiorespiratory or endocrine/metabolic conditions (Feldman et al, *Clin Infect Dis* 2024; Papi et al, *ERJ Open Research* 2026). Post-vaccination neutralizing antibody titers in these participants were at least as high as those observed in participants without such conditions, suggesting that these co-morbidities do not impair vaccine-induced immune responses in older adults (Feldman et al, *Clin Infect Dis* 2024).

In this context, while some contribution of baseline co-morbidities to variability in immune responses cannot be excluded, we believe that the presence of these conditions in the non-immunocompromised group in the current study is unlikely to have materially affected the overall interpretation of immunogenicity results in this study.

We have now added text to the Introduction (lines 108-111) describing this prior evidence indicating no apparent impact of these conditions on the vaccine's immunogenicity and efficacy.

References:

Ferguson M, Schwarz TF, Núñez SA, et al. Noninferior immunogenicity and consistent safety of respiratory syncytial virus prefusion F protein vaccine in adults 50-59 years compared to ≥60 years of age. *Clin Infect Dis*, 2024; 79(4): 1074-84.

Ferguson M, Schwarz TF, Núñez SA, et al. Immune persistence and safety of the $AS01_E$-adjuvanted respiratory syncytial virus prefusion F protein-based vaccine in adults 50-59 years of age, including at-increased-risk adults: a randomized controlled trial. *Hum Vaccin Immunother*, 2025; 21(1): 2579335.

Feldman RG, Antonelli-Incalzi R, Steenackers K, et al. Respiratory syncytial virus prefusion F protein vaccine is efficacious in older adults with underlying medical conditions. *Clin Infect Dis*, 2024; 78(1): 202-9.

Papi A, Halpin DMG, Feldman RG, et al. Adjuvanted RSVPreF3 vaccine impact over 3 RSV seasons in older adults with comorbidities. *ERJ Open Research*, 2026; 00418-2026.

6. Can the authors provide information on the breakdown on recruitment by site? There were 48 sites across 8 countries. Were there any obvious differences or skewness to any particular site/country?

Response: Recruitment by country was broadly distributed, with the largest contributions from the United States (n=104) and Spain (n=82), followed by Japan (n=61), Canada (n=47), Italy (n=28), Australia (n=26), South Korea (n=24), and Germany (n=14).

Enrolment at the individual site level was not pre-specified as a factor in the statistical analysis plan and is therefore not included in the reported analyses. Similarly, site was not included as a covariate. We acknowledge that site-level effects can be relevant in multi-center trials; however, given the relatively small sample sizes per site, such analyses would be limited in interpretability.

The study was not designed or powered for country-specific comparisons, and the primary objective was to evaluate overall patterns in this specific population. To minimize variations in laboratory assay processing, all blood samples of the study were processed and analyzed at a single, centralized laboratory.

To improve transparency and allow readers to better assess the geographical distribution of the study population, we have included the number of participants by country to the baseline characteristics table (Table 1). We have also clarified in the Methods that all blood samples for immunogenicity were analyzed centrally (lines 620-621).

7. As the study was conducted over at least a 12m period for each participant, including at least 1 RSV season, were any RSV infections recorded, either by PCR/RAT testing or serology, especially between Visit 5 and Visit 6 (6-12m period)? This would be interesting to know and likely to impact immunogenicity data at these later timepoints. Line 155 states that there were no intercurrent conditions but what does this refer to? RSV infection or any other medical condition?

Response: We thank the reviewer for this question. The study was not designed or powered to evaluate RSV infection endpoints, and assessment of RSV incidence (including confirmation by PCR, rapid antigen testing, or serology) was not included among the protocol-defined objectives or endpoints. As such, systematic surveillance for RSV infection during the study period, including between Visit 5 and Visit 6, was not performed.

The investigators could however report RSV infections as adverse events. Based on adverse event reporting, one case of RSV infection was recorded during the study. Given the absence of systematic testing and the very low number of reported cases, it is not possible to assess the potential impact of intercurrent RSV infection on immunogenicity, particularly at later timepoints.

With respect to the statement in line 155 (now line 177), "no intercurrent conditions" refers to medical conditions that could alter the immune response to the study vaccine or modify the participant's baseline immune status (other than those inherent to the transplant population), as defined in the study protocol. This term is therefore broader than RSV infection and includes any condition with the potential to influence immunogenicity.

8. What was the lost-to-follow-up rate overall and by study group?

Response: Details on participant follow-up can be found in Figure 3, participant flow. Based on the study completion data, there were no participants lost to follow-up.

At the end of the study, a total of 9 participants (2.3%) were withdrawn, with reasons including adverse events or participant-related factors (e.g., relocation or burden of the procedure), but none were due to loss to follow-up. While a proportion of participants were not included in the per-protocol immunogenicity analyses due to pre-defined criteria (e.g., missed assessments or protocol deviations), this should not be interpreted as loss to follow-up.

9. The MGI for RSVA and RSVB were lower than the expected rates for which a sample size with 80% power was determined. Can the authors comment on the interpretation of this result?

Response: We thank the reviewer for this question. The study was primarily designed to characterize the immune responses in all study groups. Exploring potential differences between immunocompromised participants (after one or two doses of adjuvanted RSVPreF3) and non-immunocompromised participants was foreseen descriptively without pre-specified success criteria. Therefore, the sample size calculation was informative (with power of 80% and 90%) and based on several hypothetical mean geometric increase (MGI) values. No formal statistical comparisons were intended or performed. Consequently, the observed MGI values should be interpreted descriptively, within the context of the study's exploratory objectives, rather than as an indication of a failure to meet a pre-specified statistical power target.

We have now clarified the description of the sample size calculation in the Supplement (section Sample size determination, page 33) to better emphasize the informative nature of the calculations and the absence of formal success criteria.

10. How did the immunogenicity compare between 2 dose recipients given between the 30-60 day interval. It is well recognised that immunogenicity is higher when given over a longer interval so this information would be interesting to know.

Response: We thank the reviewer for this insightful question and agree that the 30–60-day window at Visit 3 may have introduced variability in immunogenicity outcomes. Although the study protocol allowed the second dose to be administered within this broader window, the study was not designed or powered to formally compare immunogenicity across different dosing intervals.

To explore the potential impact of this variability in Visit 3, we conducted a post hoc descriptive analysis among 2-dose transplant recipients, examining the relationship between the neutralizing titers measured at Visit 4 (approximately 1 month post-dose 2) and Visit 5 (approximately 6 months post-dose 2) and the interval between dose 1 and dose 2. This exploratory analysis did not suggest a meaningful association between the dosing interval within the protocol-defined 30–60-day window and the observed immune responses. Given that the study was not designed nor powered for such

c

o

m

RSV-A:

n

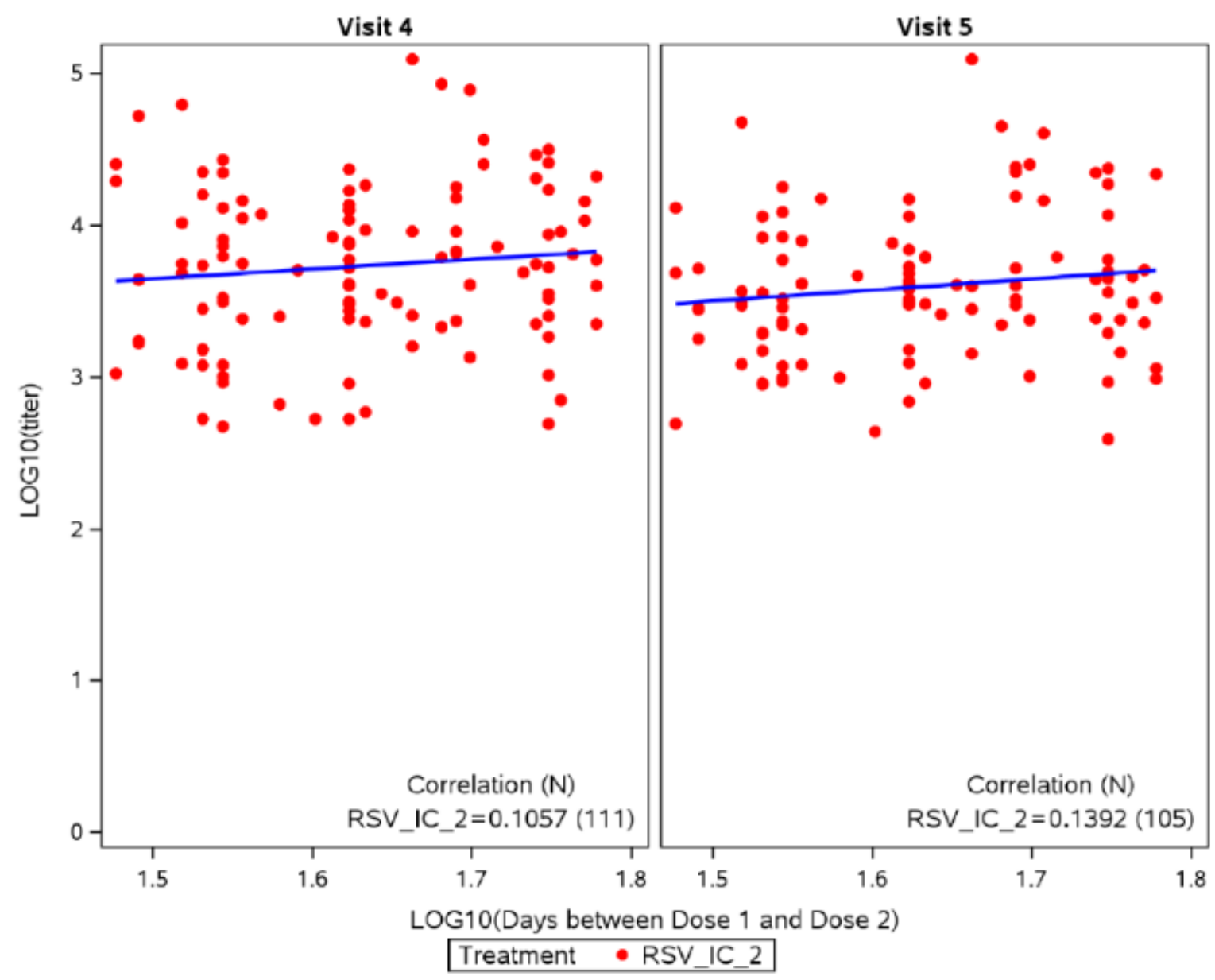

RSV_IC_2 = IC patients of >= 18 YOA receiving 2 doses of RSVPreF3 OA investigational vaccine at Visit 1 (Day 1) and Visit 3 (Visit 1 + 30-60 days)

N = number of participants with available results

d

RSV-B:

n

g

s

s

h

o

u

l

d

b

e

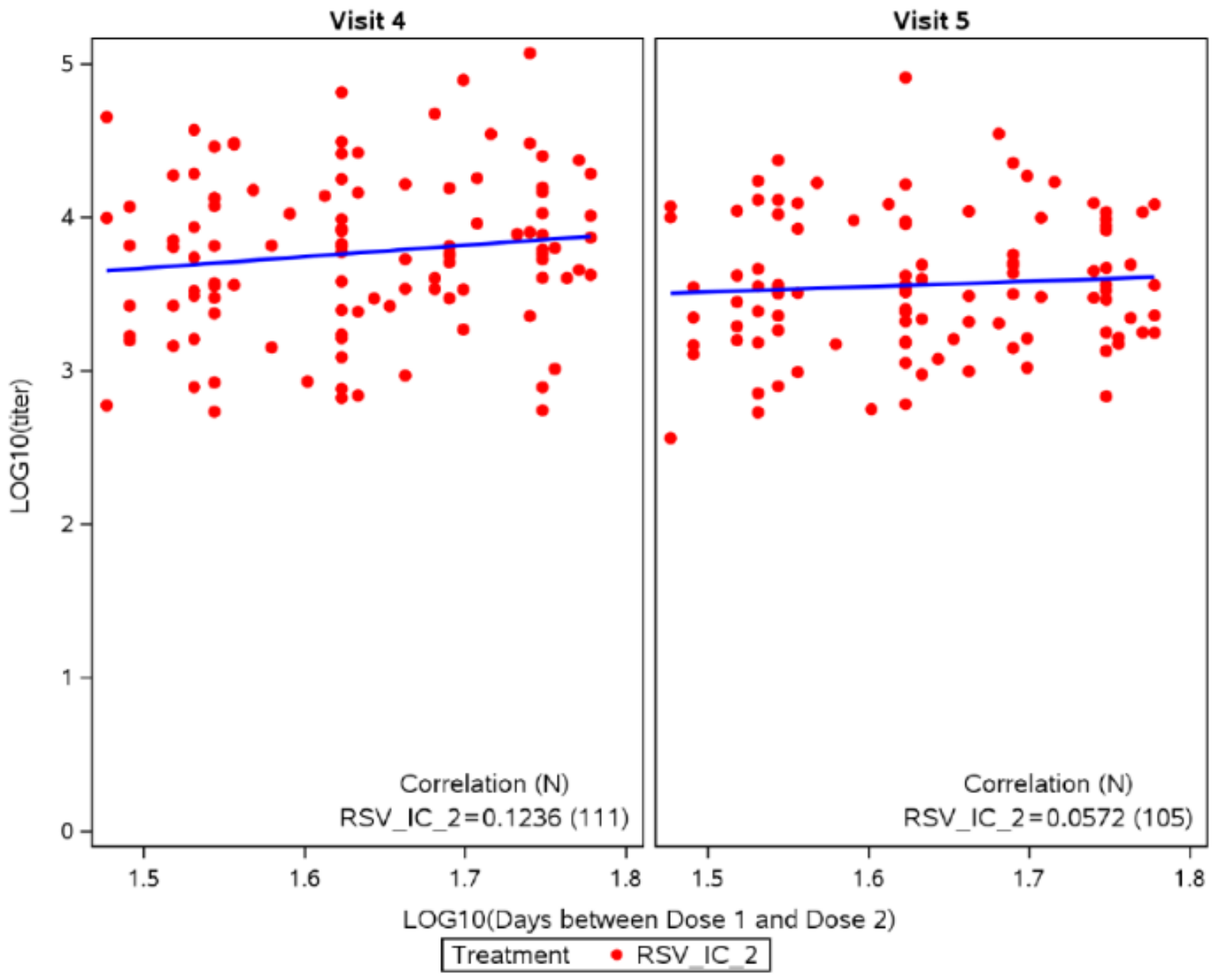

RSV_IC_2 = IC patients of >= 18 YOA receiving 2 doses of RSVPreF3 OA investigational vaccine at Visit 1 (Day 1) and Visit 3 (Visit 1 + 30-60 days)
N = number of participants with available results

11. Please indicate the RSV neutralizing assay cutoffs on the relevant Figures. Why was ED60 chosen?

Response: The lower limits of quantification of the serum neutralization assay are now included in the figure footnote (18 ED60 for RSV-A and 30 ED60 for RSV-B).

The ED60 unit aligns with previous clinical trials with adjuvanted RSVPreF3. At the start of the RSV vaccine program, the ED60 unit (i.e., a serum dilution able to neutralize 60% of the viral input) rather than ED50 was selected, based on previous research reporting the use of ED60 for titer calculation (Barbas 3rd et al, *Proc Natl Acad Sci USA* 1992; Yoder et al, *J Med Virol* 2004; Zielinska et al, *Virol J* 2005; Bates et al, *Virology* 2014), and the first reference serum available for RSV neutralization, which was titrated in ED60 (Yang et al, *Biologicals* 2007). In addition, the 60% neutralization criterion is more stringent than the 50% criterion. This helps to increase assay specificity and limits background signals.

<u>References:</u>

Barbas CF 3rd, Crowe JE Jr, Cababa D, et al. Human monoclonal Fab fragments derived from a combinatorial library bind to respiratory syncytial virus F glycoprotein and neutralize infectivity. *Proc Natl Acad Sci USA*, 1992; 89(21): 10164-8.

Yoder SM, Zhu Y, Ikizler MR, et al. Role of complement in neutralization of respiratory syncytial virus. *J Med Virol*, 2004; 72(4): 688-94.

Zielinska E, Liu D, Wu HY, et al. Development of an improved microneutralization assay for respiratory syncytial virus by automated plaque counting using imaging analysis. *Virol J*, 2005; 2:84.

Bates JT, Keefer CJ, Slaughter JC, et al. Escape from neutralization by the respiratory syncytial virus-specific neutralizing monoclonal antibody palivizumab is driven by changes in on-rate of binding to the fusion protein. *Virology*, 2014; 454-455: 139-44.

Yang DP, Zielinska E, Quiroz J, et al. Preparation of a respiratory syncytial virus human reference serum for use in the quantitation of neutralization antibody. *Biologicals*, 2007; 35(3): 183-7.

12. For CMI analyses, how was this subset determined? How does this cohort compare with the immunogenicity cohort? Some more details are needed here

Response: As specified in the protocol, the cell-mediated immunity subset was not randomly selected but was enrolled at selected study sites. Investigators at these sites allocated, where feasible at Visit 1, the first participants in each study group to the cell-mediated immunogenicity subset until the pre-defined target sample size (approximately 38 participants per group) was reached. This pragmatic approach was implemented to accommodate the logistical and technical requirements associated with cell-mediated immunogenicity assays, which could only be performed at designated sites. The cell-mediated immunogenicity subset is therefore a convenience sample rather than a randomized or stratified subset of the full study population. However, participants in the cell-mediated immunogenicity subset were enrolled contemporaneously with the overall study population and followed the same eligibility criteria and study procedures.

In terms of comparability, the cell-mediated immunogenicity subset was broadly similar to the overall immunogenicity cohort (per-protocol set for humoral immunogenicity) with respect to key baseline characteristics, although it represents a smaller sample size. No formal statistical comparisons between the cell-mediated immunogenicity subset and the overall cohort were pre-specified or performed. Given the method of selection and limited sample size, results from the cell-mediated immunogenicity analyses are considered descriptive and exploratory.

We have clarified in the manuscript the method of cell-mediated immunogenicity subset selection (lines 150-152 and 580-585), and the limitations regarding comparability and interpretation due to its non-random nature (lines 513-515).

13. In general the details of the methods are scarce, especially for the CMI analysis. Some more details are needed, including representative gating strategies. In particular how the data was calculated in terms of per million PBMCs – it is more common to represent this as % of the parent population of interest.

Response: We have expanded the Methods section to provide more details on the intracellular cytokine staining procedure and the downstream flow cytometry analysis (lines 625-652). We have also added a more detailed description of the flow cytometry gating strategy, together with a representative gating example, in the Supplementary Materials (Supplementary Figure 2).

Specifically, CMI results were quantified and reported using Boolean gating analysis to identify T cells expressing combinations of activation markers/cytokines, after which all combinations of T cells expressing at least two activation markers, including at least one cytokine, were summed to generate polypositive T-cell counts. These counts were subsequently expressed as frequencies of polypositive T cells per million CD4+ or CD8+ T cells. Polypositive T-cell frequencies obtained under background

conditions were subtracted from those obtained under stimulated conditions, to calculate RSVPreF3-specific polypositive T-cell frequencies per million CD4+ or CD8+ T cells.

This established approach has been used throughout the adjuvanted RSVPreF3 vaccine program. It was selected to normalize responses to the relevant T-cells subset, rather than per million peripheral blood mononuclear cells.

14. It is not clear what the difference is between Extended data which appears in the main manuscript and Supplementary data. I think Extended data should go into the Supplementary section.

Response: The manuscript was initially prepared and submitted to *Nature Medicine*, where the use of Extended Data figures and tables is standard. Following editorial transfer to *Nature Communications*, we have now revised the manuscript to align with its formatting requirements.

15. I could not find Extended data Figure 2 – only the Supplementary Tables 4 and 5.

Response: This was the last figure in the main manuscript; it is now revised according to new formatting guidelines and can be found as Figure 6 (provided separately, figure legend on page 58 in the current version).

16. The authors state that mycophenolate had no impact on CD4 T cell frequencies (although data is presented in actual numbers) – however it does appear that T cell levels were higher among MC group, compared to the lower antibody responses in the MC group. What is the explanation for this?

Response: We thank the reviewer for this observation. It is important to note that the cell-mediated immunogenicity analyses were conducted in a limited subset of participants, and results were expressed as frequencies (RSVPreF3-specific CD4+ T cells per million CD4+ T cells) rather than absolute functional capacity. Please also note the large interquartile ranges that overlap between the subgroups at each visit. The observed numerical differences in median frequencies may therefore reflect variability due to small sample size or differences in baseline characteristics rather than a true biological enhancement of T-cell responses in the mycophenolate group.

In line with this observation and other reviewers' comments, the wording in the manuscript has been revised to reflect the descriptive nature of the analyses in the description of CMI results (lines 293-307).

Reviewer #2:

The manuscript “Immunogenicity and safety of the AS01E-adjuvanted respiratory syncytial virus prefusion F protein vaccine in immunocompromised adults with lung or kidney transplant: a phase 2b randomized clinical trial” reports a phase I clinical trial involving 386 participants (261 transplant participants and 125 non-immunocompromised participants) that evaluated the safety and immunogenicity of the adjuvanted RSVPreF3. The results suggest that a two-dose regime was safe and immunogenic in immunocompromised adults. The study has shown novelty, is important to the related field, and is well presented in general. A few major and minor issues were identified, and suggestions for the authors’ consideration are provided below.

Major:

1. Method/Result: This is a multicountry, multicentre trial, and there is potential centre-level and country level correlation in immunity profiles, baseline seropositivity levels and lab assay processing variabilities. As the authors did not mention country or site as randomisation factor, ignoring this may underestimate SE and overestimate precision. Could you please report the number of participants by country/site in each group (baseline characteristics). The authors are encouraged to include country as an additional covariate, and conduct a sensitivity analysis by country to check the consistency.

Response: We thank the reviewer for this comment. We acknowledge that country and site were not considered as covariates in the analysis. However, it is important to reiterate that our study is primarily descriptive, aiming to characterize immunity profiles in this specific immunocompromised population. Our focus is on presenting observed patterns and distributions.

Nevertheless, to enhance transparency and provide further context for the observed data, we have included the number of participants by country to the baseline characteristics table (Table 1). This will allow readers to better understand the geographical distribution of our study population, with a fairly limited number of participants in some countries.

While a sensitivity analysis by country is a valuable suggestion for inferential studies, conducting such an analysis to check consistency across countries for purely descriptive outcomes would shift the focus from the overall observed patterns in this very specific population, which is our primary aim. The study was not powered for country-specific comparisons. We believe the proposed table of participant distribution by country will adequately address the potential for variability in a descriptive context. Importantly, to minimize variations in laboratory assay processing, all immunogenicity blood samples of the study were analyzed at a single, centralized laboratory. We have also clarified this in the Methods (lines 620-621).

2. Method/Result: The authors used GMT ratios estimated from ANCOVA with baseline titer as covariates. The authors may wish to consider a mixed model that accounts for the correlation structure of repeated measurements within individuals over multiple timepoints, i.e., the Generalised linear mixed models (GLMM) or generalised estimating equations (GEE) to model the outcome measurements as a function of time, group, and their interaction as fixed effects, site and participant as random effect and adjust for baseline. This method allows missing data handling, different intervention schedules, and between-visit fold rises are also directly estimable.

Response: We appreciate the suggestion to consider more advanced statistical models such as Generalized Linear Mixed Models (GLMM) or Generalized Estimating Equations (GEE) to account for repeated measurements and correlation structures. These methods are indeed powerful, particularly for analyzing longitudinal data from interventional studies where the goal is to model outcomes as a function of time, and to handle missing data or varying intervention schedules.

However, in line with the descriptive nature of our study, the use of ANCOVA to estimate GMT ratios was primarily for adjusting for baseline titers when presenting observed immunity profiles at the peak response (1 month post last vaccination), rather than for drawing causal inferences about specific group differences or intervention effects over time.

Our primary aim was to describe the overall immunity landscape, and the chosen methods were deemed appropriate for this purpose, providing clear and interpretable descriptive statistics. While GLMM/GEE could offer a more sophisticated view of within-individual changes, this level of inferential modeling was beyond what we intended to convey in this descriptive report. We sought to provide a clear snapshot of the observed data, and the proposed models, while robust, align more closely with inferential questions typically asked in comparative studies.

Minor:

3. Method - Sample size justification: The Statistics section states that the primary objective will be addressed using an estimation approach without hypothesis testing. However, the sample size calculation in the supplementary material is hypothesis-based, using a paired t-test framework with 80% and 90% power targets. The authors may want either provide a precision-based sample size justification, or clarify that hypothesis testing is intended.

Response: The study was primarily designed to characterize the immune responses in all study groups. Exploring potential differences between immunocompromised participants (after one or two doses of RSV vaccine) and non-immunocompromised participants was foreseen descriptively without pre-specified success criteria. Therefore, the sample size calculation was informative (with power of 80% and 90%) and based on several hypothetical MGI values. No formal statistical comparisons were intended or performed.

Consequently, the observed MGI values should be interpreted descriptively, within the context of the study's exploratory objectives, rather than as an indication of a failure to meet a pre-specified statistical power target.

We have now clarified the description of the sample size calculation in the Supplement (section Sample size determination, page 33) to better emphasize the informative nature of the calculations and the absence of formal success criteria.

4. Method - Definition of cell-mediated subset Line 521-522. It is suggested to define this subset earlier (e.g., at population section). The term was first encountered in the randomisation section, which may confuse readers unfamiliar with this term.

Response: We thank the reviewer for this helpful suggestion. In the revised manuscript, we have moved and expanded the definition of the cell-mediated immunogenicity subset to the study participants section in the Results (lines 150-152), as well as the study design section in the Methods (lines 580-585), where it is now introduced earlier and described in more detail. This ensures that readers are familiar with the concept before it is referenced in later sections, including the randomization section.

5. Method - Figure 1 and Line 499-500: if the V5 is 180 -210 days since last dose and V6 is 350-380 days since last dose, the total study period for the 2-dose transplant would be 387-440 days (13-15 months), and 1-dose transplant and 1-dose non-immunocompromised group should share the same total study period (12 month). However at Line 499-500, it says "13–15 months for transplant recipients and 12 months for non-immunocompromised participants".

Response: We thank the reviewer for highlighting this point. The difference in study duration indeed arises from how follow-up is defined relative to the last vaccine dose, which varies between groups.

For both the 1-dose transplant group and the non-immunocompromised group, Visit 6 occurs at 350–380 days after a single dose administered at Day 1, corresponding to an overall study duration of approximately 12 months. In contrast, for the 2-dose transplant group, Visit 6 occurs at 350–380 days after the second dose, which itself is administered 30–60 days after the first dose. This results in an overall study duration of approximately 13–15 months from the first dose.

We acknowledge that this distinction was not correctly phrased in the original manuscript. We have revised the text (lines 590-593) to explicitly clarify that the longer duration applies only to the 2-dose transplant group, while the 1-dose transplant and non-immunocompromised groups have a similar follow-up duration of approximately 12 months.

6. Method - Time window for V3. 30-60 days post first vaccination appears to be a relatively wide window compared to V4. Titres at day 30 and day 60 could be meaningfully different due to natural waning, and potentially introducing heterogeneity within this visit window. The authors are encouraged to provide a distribution of visit timing at V3 across groups, and potentially provide justification for this chosen window.

Response: We agree that the 30–60-day window at Visit 3 may introduce variability in immunogenicity.

To explore the potential impact of this variability, we conducted post hoc descriptive analyses of immune responses at Visit 3 when stratifying participants according to the interval between Visit 1 and Visit 3 (30–40, 41–50, and 51–60 days following the first vaccination). In all study groups, a trend toward higher neutralizing antibody responses with longer intervals was observed; however, confidence intervals of the different time intervals overlapped, and because these observations compared different, non-randomized subgroups rather than repeated measurements in the same subjects, interpretation is limited, and causal conclusions cannot be made.

Visit 3 blood draw timing	1-dose transplant N=123		2-dose transplant N=114		Non-immunocompromised N=117	
	n	GMT (95% CI)	n	GMT (95% CI)	n	GMT (95% CI)
**RSV-A neutralizing titers**						
30–40 days	53	3387.2 (2419.4–4742.0)	42	2776.3 (1810.3–4257.8)	72	6706.2 (5570.5–8073.5)
41–50 days	46	4370.1 (3037.1–6288.1)	42	5363.2 (3438.6–8365.2)	33	6867.1 (5333.3–8842.1)
51–60 days	24	4816.6 (2999.0–7735.8)	30	5200.4 (3289.4–8221.8)	12	8077.7 (4709.9–13853.7)
**RSV-B neutralizing titers**						
30–40 days	53	4408.6 (2932.5–6627.8)	42	3401.9 (2267.5–5103.7)	72	9136.3 (7432.9–11230.1)
41–50 days	46	5505.0 (3736.1–8111.5)	42	4669.8 (2999.8–7269.7)	33	8948.7 (6570.7–12187.2)
51–60 days	24	5506.4 (3056.2–9921.0)	30	8302.0 (5042.6–13668.2)	12	9559.1 (5438.1–16803.0)

The 30–60-day window between visits was selected to allow operational flexibility across multiple countries and sites, while ensuring assessment of peak or near-peak immune responses following the first dose and accommodating scheduling for the second dose in the 2-dose group. This window is consistent with early-phase immunogenicity studies where some flexibility is required without materially affecting overall interpretation.

Overall, while some variability in peak titers due to Visit 3 timing cannot be excluded, the exploratory analysis suggests that the impact on study conclusions is limited.

7. Result/Discussion: There is a gap between the wording in the result and the analysis conducted. For example, Line 233-241/Line 352-353, comparative claims are made: “GMT increased after the second dose”. The GMT at V3 and V4 for MC participants had overlapping CI, so “GMTs further increased after the second dose” may not be a valid description. As the current analysis results are by-visit GMT and MGI compared to baseline, the claim of between-visit differences should be based on the between-visit fold rise with CI. In addition, “A second dose administered…, bringing them close to non-immunocompromised levels” would require between group GMR (CI) evidence to support this claim, and this is a relatively strong causal claim. This type of comparative wording should be double-checked throughout the manuscript.

Response: We agree that some of the wording in the manuscript could be interpreted as implying formal statistical comparisons that were not performed. We have revised the manuscript throughout to ensure consistency between the descriptive nature of the analyses and the language used. Specifically, statements such as “GMTs increased after the second dose” have been revised to more accurately reflect the analysis framework, using wording such as “numerically higher GMTs were observed”.

For between-group comparisons, we have revised statements such as “bringing them close to non-immunocompromised levels” to more precise, data-supported language based on adjusted GMT ratios (GMRs) and their confidence intervals. For example, at Visit 4, the adjusted GMRs (non-immunocompromised vs 2-dose transplant) were 1.02 (95% CI: 0.80, 1.30) for RSV-A and 1.01 (0.80, 1.26) for RSV-B, indicating comparable responses rather than implying a causal effect.

In subgroup analyses (e.g., mycophenolate), as well as cell-mediated immunogenicity descriptions, similar revisions have been made to avoid overinterpretation of differences based solely on overlapping confidence intervals.

Overall, we have ensured that all comparative statements are either explicitly supported by the corresponding statistical analyses or clearly described as descriptive (e.g., “numerically higher/lower”), and we have removed or softened language that may imply unsupported causal inference.

8. Result - Table 2: As the N refers to number of exposed participants in each group, please explain the 2-dose transplant group post-dose 1 column shows N=128 for solicited events and N=130 for unsolicited AEs.

Response: For solicited events, the denominator (N=128) reflects the number of exposed participants with available reactogenicity data, which were recorded via self-completed diary cards during the 7-day reporting period. Two participants did not have evaluable diary card data and were therefore not included in the denominator for solicited events. For unsolicited adverse events, the denominator (N=130) includes all exposed participants, as these events were recorded by the investigator.

We have further clarified the reason for this difference in the table footnote (now Table 4).
